# Genetically engineered distal airway stem cell transplantation protects mice from pulmonary infection

**DOI:** 10.15252/emmm.201810233

**Published:** 2019-11-29

**Authors:** Yue‐qing Zhou, Yun Shi, Ling Yang, Yu‐fen Sun, Yu‐fei Han, Zi‐xian Zhao, Yu‐jia Wang, Ying Liu, Yu Ma, Ting Zhang, Tao Ren, Tina P Dale, Nicholas R Forsyth, Fa‐guang Jin, Jie‐ming Qu, Wei Zuo, Jin‐fu Xu

**Affiliations:** ^1^ Department of Respiratory and Critical Care Medicine Clinical Translation Research Center Shanghai Pulmonary Hospital Tongji University School of Medicine Shanghai China; ^2^ Shanghai East Hospital Tongji University School of Medicine Shanghai China; ^3^ Department of Respiratory and Critical Care Medicine Tangdu Hospital Fourth Military Medical University of PLA Xi'an China; ^4^ Regend Therapeutics Co. Ltd Zhejiang China; ^5^ Guy Hilton Research Center School of Pharmacy and Bioengineering Keele University Staffordshire UK; ^6^ Ruijin Hospital Shanghai Jiaotong University School of Medicine Shanghai China; ^7^ Institute of Respiratory Diseases Shanghai Jiaotong University School of Medicine Shanghai China; ^8^ Guangzhou Institute of Respiratory Disease The First Affiliated Hospital of Guangzhou Medical University Guangzhou China; ^9^ Ningxia Medical University Yinchuan China

**Keywords:** antimicrobial peptide, distal airway stem cells, pulmonary infection, transplantation, Immunology, Respiratory System

## Abstract

Severe pulmonary infection is a major threat to human health accompanied by substantial medical costs, prolonged inpatient requirements, and high mortality rates. New antimicrobial therapeutic strategies are urgently required to address the emergence of antibiotic resistance and persistent bacterial infections. In this study, we show that the constitutive expression of a native antimicrobial peptide LL‐37 in transgenic mice aids in clearing *Pseudomonas aeruginosa* (PAO1), a major pathogen of clinical pulmonary infection. Orthotopic transplantation of adult mouse distal airway stem cells (DASCs), genetically engineered to express LL‐37, into injured mouse lung foci enabled large‐scale incorporation of cells and long‐term release of the host defense peptide, protecting the mice from bacterial pneumonia and hypoxemia. Further, correlates of DASCs in adult humans were isolated, expanded, and genetically engineered to demonstrate successful construction of an anti‐infective artificial lung. Together, our stem cell‐based gene delivery therapeutic platform proposes a new strategy for addressing recurrent pulmonary infections with future translational opportunities.

## Graphical abstract

Using adult distal airway stem cells (DASCs) as a natural vehicle to deliver the antimicrobial peptide LL‐37 into damaged lung, this study reports a technology to restore epithelium barrier and pulmonary innate immunity, which could have applications for the treatment of infectious lung diseases.

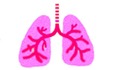

## Introduction

Respiratory infection is amongst the leading causes of human death. These include lower respiratory tract infections by Gram‐negative pathogens such as *Pseudomonas aeruginosa* which constitute the main reason for hospital‐associated infections and are associated with high morbidity and mortality rates in hospitals. These continue to pose a therapeutic challenge due to the rapid development of resistance to standard antibiotic regimes during treatments. In the case of the opportunistic pathogen *P. aeruginosa*, broad antibiotic resistance has been observed, and 18–25% of clinical isolates demonstrated multidrug resistance (Souli *et al*, 2008). Unfortunately, relapse or re‐infection is a frequent occurrence in patients with pulmonary infections. To this end, the development of new therapeutic strategies is needed to combat pulmonary bacterial infections.

Antimicrobial peptides (AMPs) are substances produced by animals, bacteria, and plants that are regarded as naturally occurring broad‐spectrum antibiotics. As an essential part of innate immunity, AMPs possess the ability to kill invading pathogens including bacteria, fungi, virus, and parasites (Zasloff, 2002; Fjell *et al*, 2011; Kovach *et al*, 2012). The peptide hCAP‐18/LL‐37 (LL‐37) is the only human cathelicidin (CAMP) identified so far. The LL‐37 peptide is cleaved from hCAP‐18 by proteinase, which enables it to have a broad range of bactericidal activity against both Gram‐negative and Gram‐positive organisms, including *P. aeruginosa*. LL‐37 has roles in multiple host defense processes by directly targeting microbial biofilm and activating innate immune cell function (Scott *et al*, 2002; Overhage *et al*, 2008; Yu *et al*, 2010; Bandurska *et al*, 2015). In the inflamed human lung, LL‐37 was reported to be highly expressed and had potent anti‐infective and anti‐inflammatory potential (Nijnik & Hancock, 2009; Currie *et al*, 2016), suggesting that the LL‐37 peptide can be used as an alternative medicine to conventional antibiotics for treating pulmonary infection. However, the degradation of the LL‐37 peptide *in vivo* due to bacterial proteases may limit its clinical application (Vandamme *et al*, 2012). Furthermore, as a peptide with potential off‐target toxicity, LL‐37 requires topical delivery to infected foci, rather than systemically, with local concentration control (Johansson *et al*, 1998; Heilborn *et al*, 2005). Therefore, the development of a system to achieve local, long‐term LL‐37 release may help combat pulmonary infection.

Viral systems have been utilized for exogenous gene expression; however, clinical applicability for this approach has multiple drawbacks including virus‐induced tissue toxicity and inflammation post‐virus infection, oncogenic risks and genotoxicity, and the off‐target effects of the viral vector (i.e., liver). Here, we introduce a novel platform combining conventional viral‐based gene engineering with intrapulmonary stem cell transplantation. We and others previously demonstrated that distal airway stem cells (DASCs) derived from p63^+^ lineage negative progenitors are the major regenerative cells following large‐scale lung damage (Kumar *et al*, 2011; Vaughan *et al*, 2015; Zuo *et al*, 2015; Yang *et al*, 2018). DASCs have the capacity to rapidly restore epithelial barriers *in vivo* and differentiate into functional alveolar cells with accompanied Notch signaling (Vaughan *et al*, 2015; Xi *et al*, 2017). The feasibility for large‐scale *in vitro* expansion and remarkable lung engraftment after transplantation (Zuo *et al*, 2015; Imai‐Matsushima *et al*, 2018) make DASCs ideal candidates for cell therapy and gene engineering.

In the current study, via a novel transgenic rodent model, we show that constitutive expression of human LL‐37 peptide, in the lung, enhances the pulmonary host defense system. Introduction of LL‐37 into mouse DASCs enable delivery of the antimicrobial peptide specifically into injured foci without distribution to other healthy lung regions, and endow the lung with enhanced bacterial clearance ability. An anti‐infective human bioengineered lung is also constructed by engrafting LL‐37‐overexpressing human DASCs into decellularized lung scaffolds. Taken together, we demonstrate that genetically engineered DASCs can efficiently and specifically deliver the antimicrobial peptide LL‐37 *in vivo* and protect the lung against pathogen infection.

## Results

### Constitutive LL‐37 expression clears pulmonary infection *in vivo*

**Figure 1:**
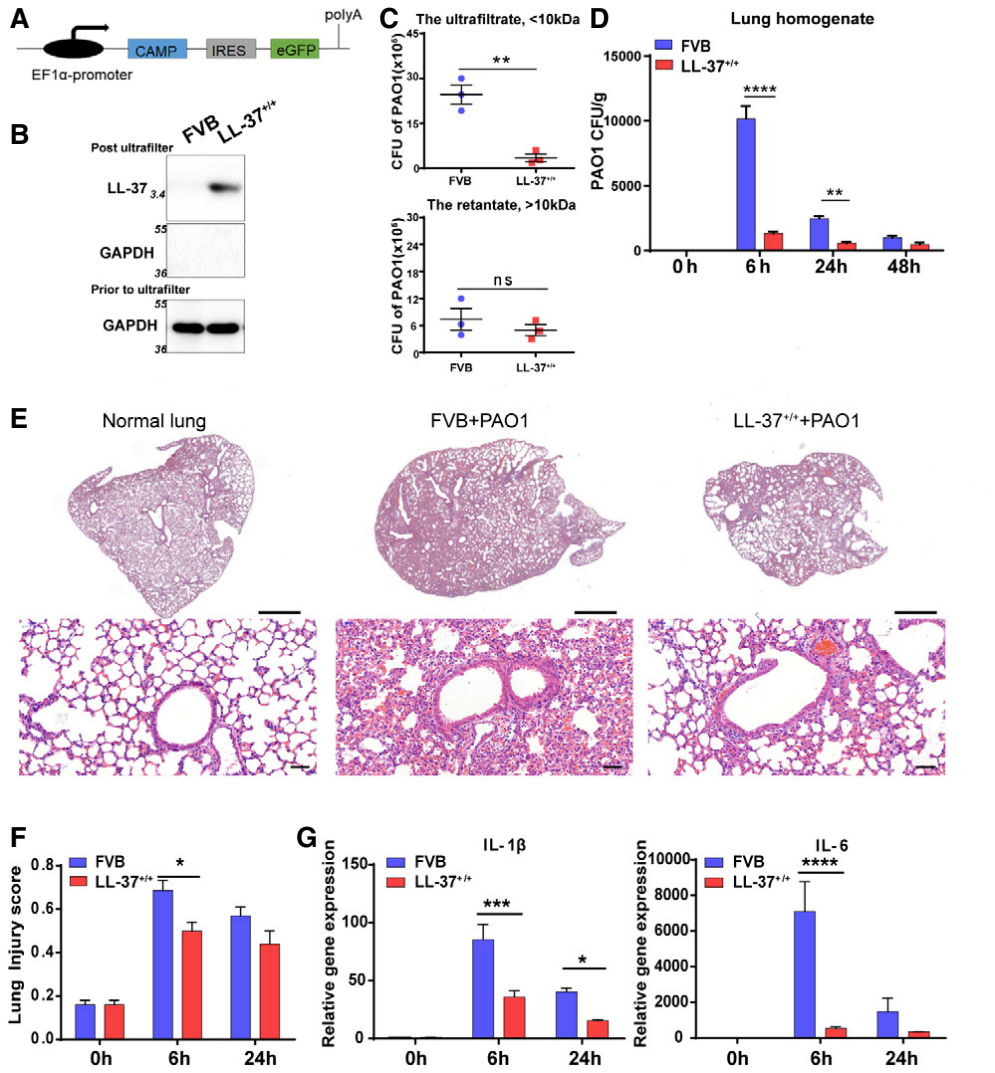
Constitutive expression of LL‐37 protected mouse lung from bacterial infection AThe schematic of human LL‐37(CAMP) transgenic mouse strain.BLL‐37(4‐kD) detection by Western blotting. Prior to loading, samples were centrifuged through 10‐kD ultrafiltration membranes and an equal amount of ultrafiltrate (19 μg/lane) was subjected to immunoblotting. High‐molecular‐weight proteins (GAPDH) were not detected in ultrafiltrate.CThe CFU of PAO1 was measured by culturing in ultrafiltrate (upper panel) or retentate (lower panel) of mouse BALF samples from indicated mice. Initial additions of PAO1 were 1 × 10^3^ CFU. Co‐culture duration, 6 h. *n* = 3. Error bars, SEM.DThe bacterial CFU (per gram) in lungs of indicated mice with and without PAO1 infection (5 × 10^6^ CFU). *n* = 3. Error bars, SEM.ERepresentative histological sections of indicated lungs with PAO1 infection (5 × 10^6^ CFU) for 6 h. H&E staining. Scale bar, 1,000 μm (upper panel) and 50 μm (lower panel).FHistopathological injury score of indicated mouse lungs with PAO1 infection (5 × 10^6^ CFU) based on blinded expert judgment. *n* = 3. Error bars, SEM.GGene expression level of IL‐1β and IL‐6 of indicated mouse lung with PAO1 infection (5 × 10^6^ CFU). *n* ≥ 3. Error bars, SEM.Data information: Statistics for graphs: unpaired two‐tailed *t*‐test (C) and two‐way ANOVA followed by Sidak's test (D, F, G). *P < 0.05; **P < 0.01; ***P < 0.001; ****P < 0.0001.Source data are available online for this figure.

Clinical observations have indicated the elevation of LL‐37 expression in lung disease exacerbation (Schaller‐Bals *et al*, 2002; Pouwels *et al*, 2015), suggesting that pulmonary infection and inflammation could activate the LL‐37‐based protective mechanism. Here, to understand whether the elevated expression of LL‐37 *in vivo* is beneficial, we constructed a novel transgenic mouse strain that constitutively expressed the human hCAP‐18 gene driven by the EF1a promoter (Fig 1A). The expressed 18‐kD hCAP‐18 precursor required additional processing to produce 4‐kD LL‐37 and acquired biological functionality. To detect LL‐37 expression, we collected the lysate from wild‐type FVB and LL‐37 transgenic mice and concentrated low‐molecular‐weight proteins by passing through a 10‐kD centrifugal filter device. The expression of 4‐kD LL‐37, but not larger proteins (i.e., GAPDH), was detected in the low‐molecular‐weight ultrafiltrate in the transgenic mice (Fig 1B).

Bronchoalveolar lavage fluid (BALF) ultrafiltrate from LL‐37 transgenic mice demonstrated a moderately enhanced bacteria inhibitory function than wild‐type FVB BALF when used to culture PAO1 *in vitro*. In contrast, high‐molecular‐weight retentates displayed little difference in their bacterial inhibitory effect (Fig 1C). We also challenged mouse lung with equal amounts of PAO1 (5 × 10^6^ CFU of PAO1) and analyzed the lung homogenate at different time points after infection. The results showed that the LL‐37 transgenic mice had significantly enhanced bacterial clearance compared to wild‐type FVB mice, leaving less residual infection (Fig 1D). Histological analysis indicated that LL‐37 transgenic mice lung tissue displayed alleviated alveolar tissue damage at 6 h after PAO1 infection, potentially due to reduced bacterial burden (Fig 1D–F). Furthermore, the mRNA levels of major pro‐inflammatory cytokines including IL‐6 and IL‐1β decreased in the lungs of LL‐37 transgenic mice (Fig 1G).

**Figure 2:**
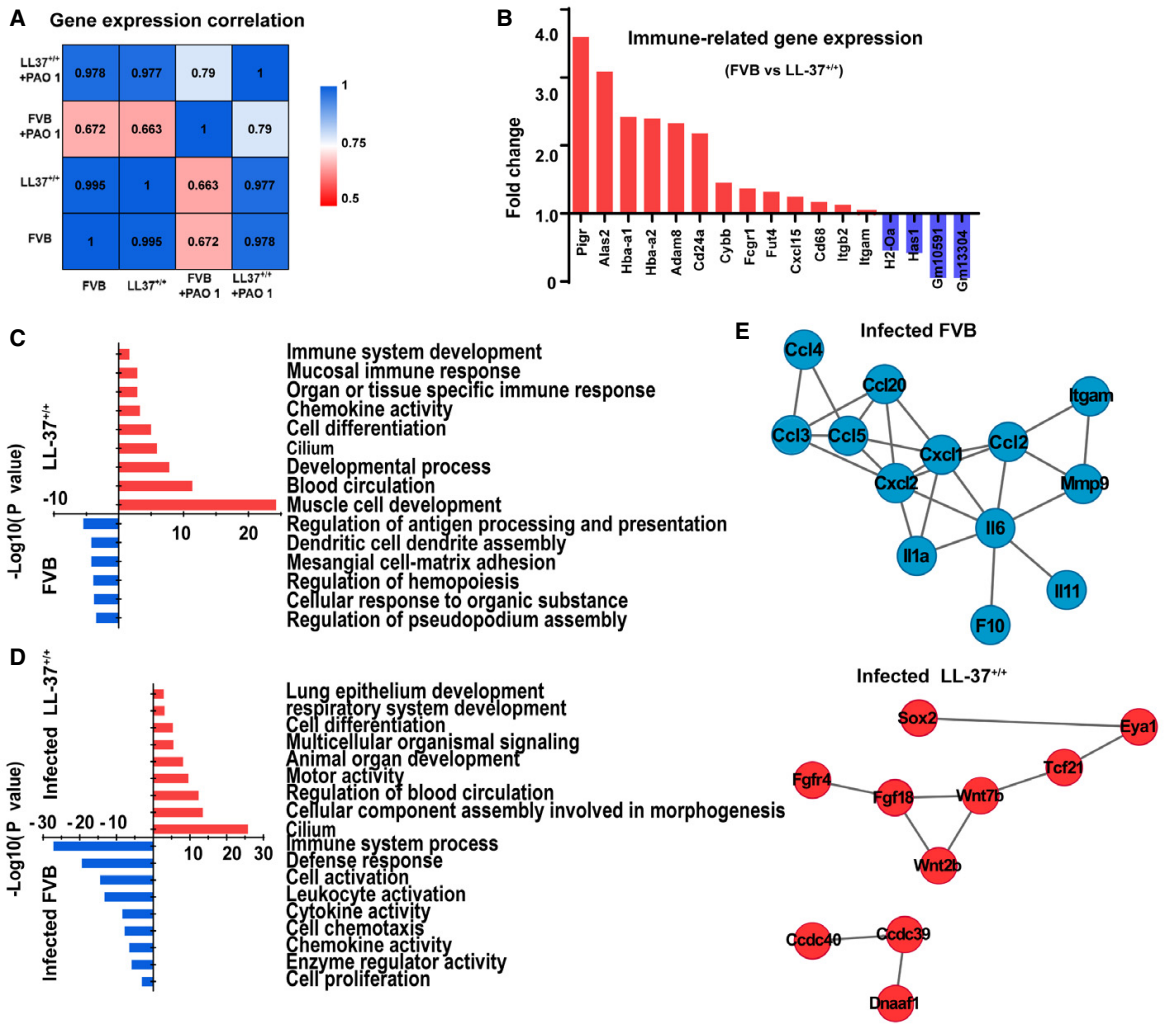
The altered transcriptomic profiles of transgenic mouse lungs before and after PAO1 infection AHeatmap showing transcriptome profile correlation values of indicated lung tissue samples before and after PAO1 infection.BHistogram of selected differentially expressed genes of LL‐37^+/+^ mouse lung versus wild‐type FVB mouse lung prior to infection. Blue bars indicated genes upregulated in wild‐type FVB mouse lungs, while red bars indicated genes upregulated in LL‐37^+/+^ mouse lungs.C, DEnriched Gene Ontology classes of uninfected (C) and PAO1‐infected (D) lungs. Red bar, GO class of upregulated gene in LL‐37^+/+^ mice. Blue bar, GO class of upregulated gene in wild‐type FVB mouse lung. GO terms were ranked by the enrichment *P*‐value.EProtein–protein interaction network of selected genes with high expression level in PAO1‐infected wild‐type lung (blue) and PAO1‐infected LL‐37^+/+^ lung (red), respectively. Source data are available online for this figure.

To further characterize the LL‐37 transgenic mouse lung before and after bacterial infection, we performed RNA‐Seq on lung tissues to analyze their whole transcriptomic profiles. As expected, FVB lungs had distinct transcriptomic profiles before and after PAO1 infection (PCC = 0.672). In contrast, LL‐37^+/+^ lungs shared highly similar whole transcriptomic profiles before and after PAO1 infection (PCC = 0.977), suggesting that the LL‐37^+/+^ lungs were protected from PAO1 challenge‐induced alterations (Fig 2A). Interestingly, we found that overexpression of LL‐37 gave rise to upregulation of multiple immune response‐related genes even prior to infection (Fig 2B). Further analysis on gene ontology revealed that LL‐37 expression enhanced normal mice development, including muscle, and blood circulation, and augmented mucosal immune response and organ‐specific immune responses (Fig 2C). This finding indicated that LL‐37 could stimulate lung immunity to protect infection, which was consistent with previous reports on other cathelicidin or cathelicidin‐related peptides (Kovach *et al*, 2012; Beaumont *et al*, 2014).

Next, we analyzed the lung transcriptomic profiles after PAO1 infection. The infected FVB mouse lungs were characterized by elevated infection and immune‐related processes, such as “defense response” and “leukocyte activation”; in contrast, the infected LL‐37^+/+^ lungs were characterized by lung tissue homeostasis/development‐related processes, such as “lung epithelium development,” “respiratory system development,” and “cilium” (Fig 2D and Table EV1). Protein–protein interaction network analysis of overexpressed genes identified an inflammation‐related molecular network in infected WT lungs, and in contrast, a lung development‐related molecular network in infected LL‐37^+/+^ lungs (Fig 2E). These data indicate that constitutive LL‐37 expression in mouse lung can protect the lung from bacterial infection and inflammation.

### Genetically engineered mDASCs express functional LL‐37 peptide

**Figure 3:**
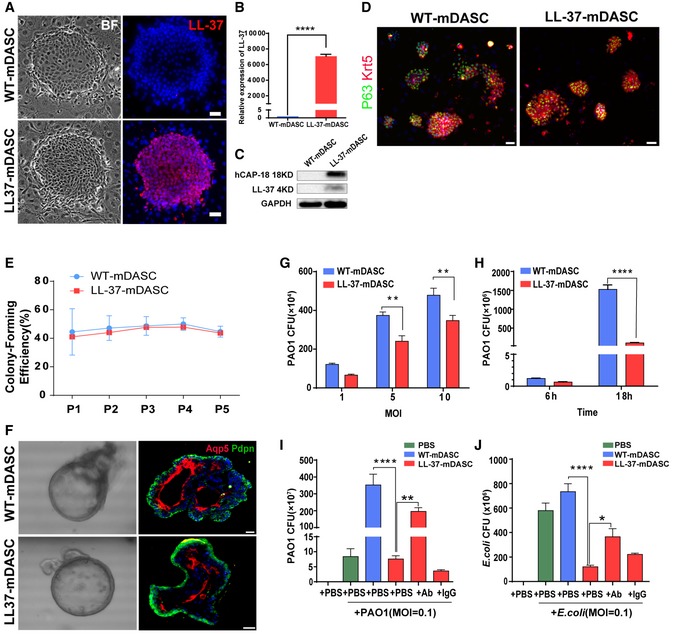
Engineered mDASCs possessed normal stem cell properties and enhanced antimicrobial potency A–CDetection of LL‐37 expression in the engineered mDASCs by immunofluorescence (A), real‐time quantitative PCR (B), and Western blot (C). Scale bar, 50 μm. BF, bright field. *n* = 10. Error bars, SEM.DAnti‐Krt5 (red) and anti‐P63 (green) immunostaining of WT‐ and LL‐37‐mDASC colonies. Scale bar, 70 μm.EStem cell colony‐forming efficiency of WT‐ and LL‐37‐mDASCs during five serial passages. *n* = 6. Error bars, SD.FRepresentative 3D organoid culture of mDASCs with expression of type I alveolar cell markers (Aqp5 and Pdpn). Left panels, bright‐field imaging of 3D organoids. Right panels, immunofluorescence of organoid sections. Scale bar, 20 μm.GCo‐culture of bacteria with DASCs shows antimicrobial effects in dose‐dependent manner. Initial additions of PAO1 were 0.1 × , 0.5 × and 1 × 10^4 ^CFU, respectively. Co‐culture duration, 6 h. *n* = 4. Error bars, SEM. MOI, multiplicity of infection.HCo‐culture of bacteria with DASCs shows antimicrobial effects in time‐dependent manner. Initial concentration of PAO1 was 1 × 10^4 ^CFU. MOI = 1. *n* = 3. Error bars, SEM.I, JPreincubation of cells with anti‐LL‐37 antibody, but not IgG control, significantly reduced anti‐PAO1 (I) and anti‐*Escherichia coli* (J) effects of LL‐37‐mDASCs. Initial dose of bacteria was 10^3 ^CFU. Co‐culture duration, 18 h. *n* = 4 in (I) and *n* = 3 in (J). Error bars, SEM.Data information: Statistics for graphs: unpaired two‐tailed *t*‐test (B), two‐way ANOVA followed by Sidak's test (G, H) and one‐way ANOVA followed by Tukey's test (I, J). *P < 0.05; **P < 0.01; ****P < 0.0001.Source data are available online for this figure.

**Figure EV1:**
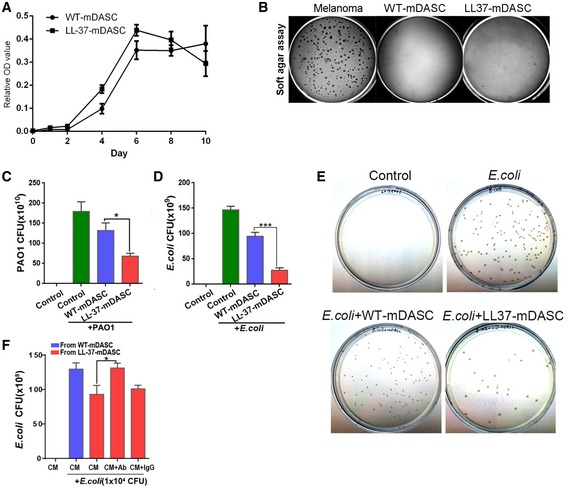
The antimicrobial effect of LL‐37‐mDASCs *in vitro* ACell growth curve of WT‐ and LL‐37‐mDASCs was measured by MTT assay. *n* = 3–5. Error bars, SD.BSoft agar assay of WT‐ and LL‐37‐mDASCs. Mouse melanoma cell line was included as a positive control.CHistogram showed that LL‐37‐mDASCs conditioned medium (CM) had potent growth inhibitory effect on PAO1. Initial addition of PAO1 was 1 × 10^4^ CFU. *n* = 5. Error bars, SEM.DHistogram shows that LL‐37‐mDASCs CM had potent growth inhibitory effect on *Escherichia coli*. Initial addition of *E. coli* was 1 × 10^4^ CFU. *n* = 3. Error bars, SEM.EClone formation unit assay of *E. coli* following incubation with indicated cellular CM.FPreincubation of CM with anti‐LL‐37 antibody, but not mouse IgG, reduced the antimicrobial effect of LL‐37‐mDASCs against *E. coli*. *n* = 3. Error bars, SEM.Data information: Statistics for graphs: one‐way ANOVA followed by Tukey's test. *P < 0.05; ***P < 0.001.

LL‐37 aids bacterial clearance in the mouse lung. We next determined if this peptide could be used in combination with DASCs to protect damaged lung from infection. P63^+^/Krt5^+^ DASCs (mDASCs) were isolated from normal adult mouse lung and expanded on 3T3 feeder cells as stem cell clones. The LL‐37 gene was introduced into mDASCs by lentiviral transduction. Constitutive LL‐37 expression was detected at both RNA and protein levels in LL‐37‐mDASCs but not in their wild‐type counterpart (Fig 3A–C). WT‐mDASCs and LL‐37‐mDASCs expressed similar levels of stem cell markers P63 and Krt5 (Fig 3D). mDASCs, irrespective of types, were able to be passaged indefinitely in our system. Elevated LL‐37 expression was previously reported to affect cell viability and proliferation (Heilborn *et al*, 2005), while in this study we did not detect significant differences in either proliferation rate (Fig EV1A) or clonogenic ability (Fig 3E). In a three‐dimensional organoid culture system, both mDASC cell lines formed alveolar‐like sphere structures consisting of differentiated cells expressing AQP5 and PDPN, type I alveolar cell markers (Fig 3F). To confirm that genetically modified mDASCs were not tumorigenic, we assessed the anchorage‐independent growth potential of these cells. mDASCs after LL‐37 lentiviral transduction were unable to grow in soft agar medium, while mouse melanoma cells (B16) exhibited robust colony‐forming efficiency under identical conditions (Fig EV1B). This indicated the successful generation of a LL‐37‐expressing mDASC cell line with unaltered self‐renewal and differentiation properties.

To test whether functional LL‐37 peptide could be produced and secreted by LL‐37‐mDASCs, we assessed the bacterial clearance ability of their culture‐conditioned medium (CM). PAO1 growth was significantly inhibited by the CM from LL‐37‐mDASCs but not from WT‐mDASCs (Fig EV1C). In a series of cell/bacteria co‐culture assays, LL‐37‐mDASCs, when compared to WT‐mDASCs, showed impaired bacterial growth of PAO1 at different infection doses and time points, although substantial bacterial proliferation was observed under both conditions (Fig 3G–I). A similar bacterial growth inhibitory effect of LL‐37‐mDASCs was also detected with Gram‐negative pathogen, *Escherichia coli* (Figs 3J and EV1D and E). To confirm that the inhibitory effect of engineered cells was attributed to LL‐37 peptide production, we used anti‐LL‐37 antibody to neutralize the secreted peptide. Compared with IgG control, anti‐LL‐37 antibody compromised the inhibitory effect of LL‐37‐mDASCs (Fig 3I and J) and their cellular CM (Fig EV1F). Collectively, the above data demonstrate successful engineering of LL‐37‐mDASCs with normal stem cell properties and antimicrobial functions.

### Regeneration of LL‐37‐Lung by transplantation of genetically engineered mDASCs

**Figure 4:**
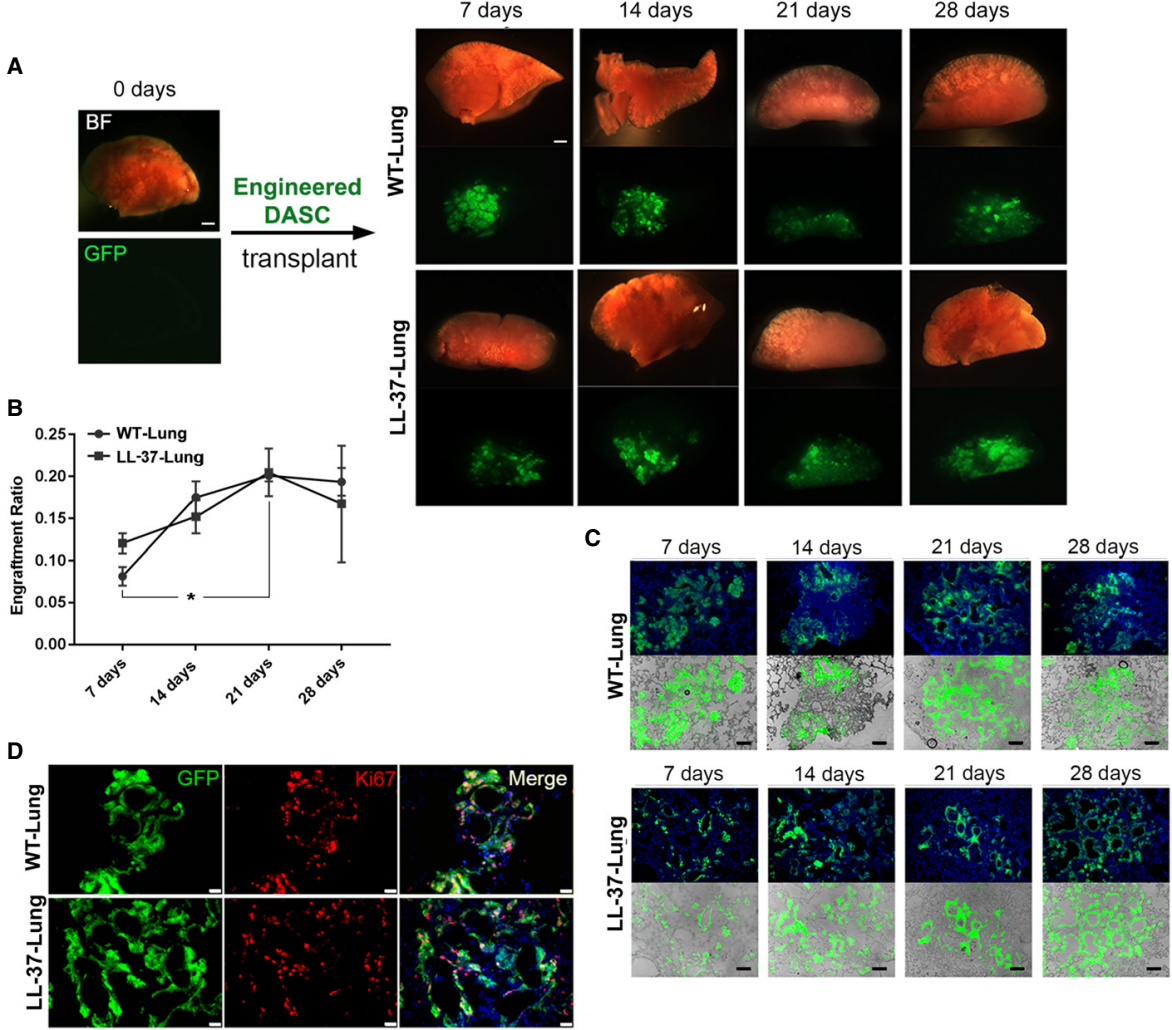
Lung engraftment of WT‐ and LL‐37‐mDASCs after orthotopic transplantation ABright‐field and direct fluorescence images of mouse lungs following transplantation of 1 × 10^6^ GFP‐labeled WT‐mDASCs (WT‐Lung) or LL‐37‐mDASCs (LL‐37‐Lung) on indicated days. Scale bar, 1,000 μm.BEngraftment ratio of indicated cells in mouse lungs on indicated days. *n* = 3. Error bars, SEM. *P = 0.0474. Statistics: two‐way RM ANOVA followed by Tukey's test.CMorphology of engrafted GFP‐labeled cells in lung parenchyma by direct fluorescence. Blue color indicates nucleus DAPI staining. Scale bar, 200 μm.DAnti‐Ki67 immunofluorescence of engrafted GFP‐labeled WT‐ and LL‐37‐mDASCs in lung parenchyma 21 days after transplantation. Scale bar, 50 μm. Source data are available online for this figure.

**Figure EV2:**
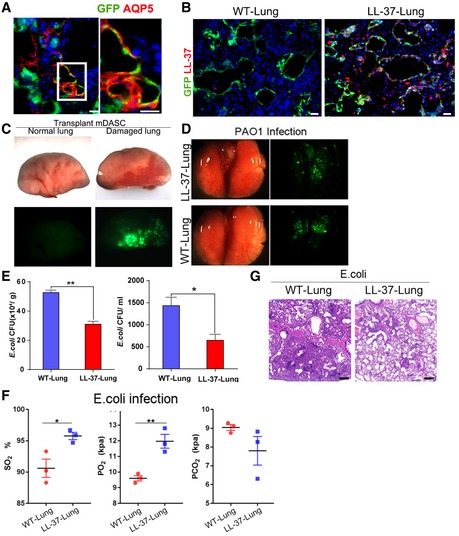
The antimicrobial effect of LL‐37‐Lung ALeft, immunostaining of engrafted LL‐37‐mDASCs cells with anti‐GFP and anti‐AQP5 (type I alveolar cell marker) antibodies; right, amplification of inset in upper panel indicated regenerated alveolar structure. Scale bar, 30 μm.BDistribution of engrafted GFP‐labeled cells in lung parenchyma by immunostaining 21 days after transplantation. WT‐Lung, WT‐mDASCs engrafted; LL‐37‐Lung, LL‐37‐mDASCs engrafted. Scale bar, 40 μm.CDirect fluorescence image of lungs from normal mouse or bleomycin injured mouse 7 days after transplantation of 1 × 10^6^ GFP‐labeled mDASCs.DDirect fluorescence image under stereomicroscope showing mouse lung transplanted with 1 × 10^6^ GFP‐labeled WT‐ and LL‐37‐mDASCs followed by PAO1 infection.EIntratracheal instillation of equal amounts of *E. coli* (5 × 10^6 ^CFU per mouse) into WT‐Lung (WT‐mDASCs engrafted) and LL‐37‐Lung (LL‐37‐mDASCs engrafted) followed by bacterial CFU analysis in whole lung homogenates (left panel) and BALF (right panel) 2 days after infection. *n* = 3. Error bars, SEM.FArterial blood gas analysis of mice with WT‐Lung and LL‐37‐Lung following *E. coli* infection 2 days after transplantation. *n* = 3. Error bars, SEM.GH&E staining showing histology of WT‐Lung and LL‐37‐Lung with *E. coli* infection after 2 days of cell transplantation. Scale bar, 200 μm.Data information: Statistics for graphs: unpaired two‐tailed *t*‐test. *P < 0.05; **P < 0.01.

To investigate the potential therapeutic effect of engineered DASCs *in vivo*, we transplanted the LL‐37‐mDASCs into the damaged lung of syngeneic animals. The chemotherapeutic drug bleomycin was intratracheally instilled into the mouse lung to induce acute pulmonary inflammation and alveolar tissue damage. Seven days after bleomycin administration, 10^6^ GFP‐labeled WT‐mDASCs or LL‐37‐mDASCs were intratracheally delivered into injured mouse lungs. We named the lungs with WT‐mDASCs or LL‐37‐mDASCs engraftment as WT‐Lung or LL‐37‐Lung, respectively. The lung tissues were harvested for analysis on different days after transplantation. Substantial incorporation of mDASCs into mouse lung was detected without observing significant rejection of cells (Fig 4A). An equal engraft ratio was observed for the two cell types, which both peaked around 21 days after transplantation and remained stable thereafter (Fig 4B). Tissue sectioning and analysis showed a broad distribution of stem cells and their progeny in mouse lung parenchyma with a substantial proportion differentiated into air sac‐like structures with type I alveolar cell marker AQP5 co‐staining (Figs 4C and EV2A). A population of transplanted engineered cells continued to express the proliferation marker Ki67 up to 28 days after transplantation (Fig 4D), suggesting their high viability and extended contribution to the tissue regeneration process.

**Figure 5:**
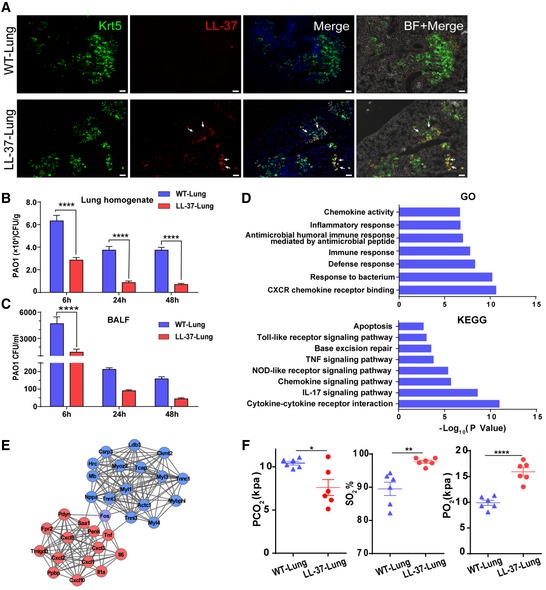
LL‐37‐expressing lung had enhanced host defense ability ADistribution of engrafted GFP‐labeled cells in lung parenchyma by immunofluorescence 7 days after transplantation. WT‐Lung, WT‐mDASCs engrafted; LL‐37‐Lung, LL‐37‐mDASCs engrafted. Scale bar, 200 μm. Arrows show the representative cells with overlapping fluorescence of GFP and LL‐37.BIntratracheal instillation of equal amount of PAO1(5 × 10^6^ CFU per mouse) into WT‐Lung (WT‐mDASCs engrafted) and LL‐37‐Lung (LL‐37‐mDASCs engrafted) followed by bacterial CFU analysis in whole lung homogenates 6, 24, and 48 h after infection. *n* = 3. Error bars, SEM.CIntratracheal instillation of equal amount of PAO1 into WT‐Lung and LL‐37‐Lung followed by bacterial CFU analysis in BALF 6, 24, and 48 h after infection. *n* = 3. Error bars, SEM.DDownregulated gene in LL‐37‐Lung enriched in Gene Ontology and KEGG pathways.EProtein–protein interaction network of selected genes with high expression level in WT‐Lung (red) and LL‐37‐Lung (blue), respectively.FArterial blood gas analysis of mice with WT‐Lung and LL‐37‐Lung following PAO1 infection. *n* = 6. Error bars, SEM.Data information: Statistics for graphs: two‐way ANOVA followed by Sidak's test (B, C) and unpaired two‐tailed *t*‐test (F). *P < 0.05; **P < 0.01; ****P < 0.0001.Source data are available online for this figure.

For the LL‐37‐mDASC group, constitutive LL‐37 expression was specifically detected in the lung area with GFP^+^ stem cell incorporation (Figs 5A and EV2B). Importantly, there was no incorporation of GFP^+^ stem cells in the uninjured lung (Fig EV2C), indicating specific targeting of LL‐37 toward injured but not healthy lung tissue.

### Regenerated LL‐37‐Lung has enhanced bacterial clearance ability

Previously, the capacity of LL‐37 to enhance pulmonary bacterial clearance 6 and 24 h post‐PAO1 infection has been demonstrated (Beaumont *et al*, 2014). To assess whether the LL‐37‐Lung had enhanced host defense post‐bacterial infection, WT‐Lung and LL‐37‐Lung were challenged with intratracheal delivery of PAO1 (Fig EV2D). The PAO1 load levels in mouse lung were quantified by bacterial culture 6, 24, and 48 h after infection. LL‐37‐Lung had significantly less PAO1 load in the whole lung homogenate (Fig 5B) as well as in BALF (Fig 5C). A similar effect was observed when the lungs were challenged with *E. coli* (Fig EV2E) 48 h after infection. Correspondingly, differentially expressed genes measured by RNA‐Seq between infected WT‐Lungs and LL‐37‐Lungs indicated that LL‐37 expression alleviated the inflammation reaction and downregulated the related pro‐inflammatory pathways (Fig 5D and E). Consequently, 2 days after infection, the mice with LL‐37‐Lung demonstrated healthier pulmonary function, as shown by the higher O_2_ partial pressure and O_2_ saturation, yet lower CO_2_ partial pressure in arterial blood (Figs 5F and EV2F). Altogether, the data above showed that the LL‐37‐expressing mDASC engraftment could protect mouse lung from bacterial infection and improve the pulmonary function of infected recipients.

**Figure 6:**
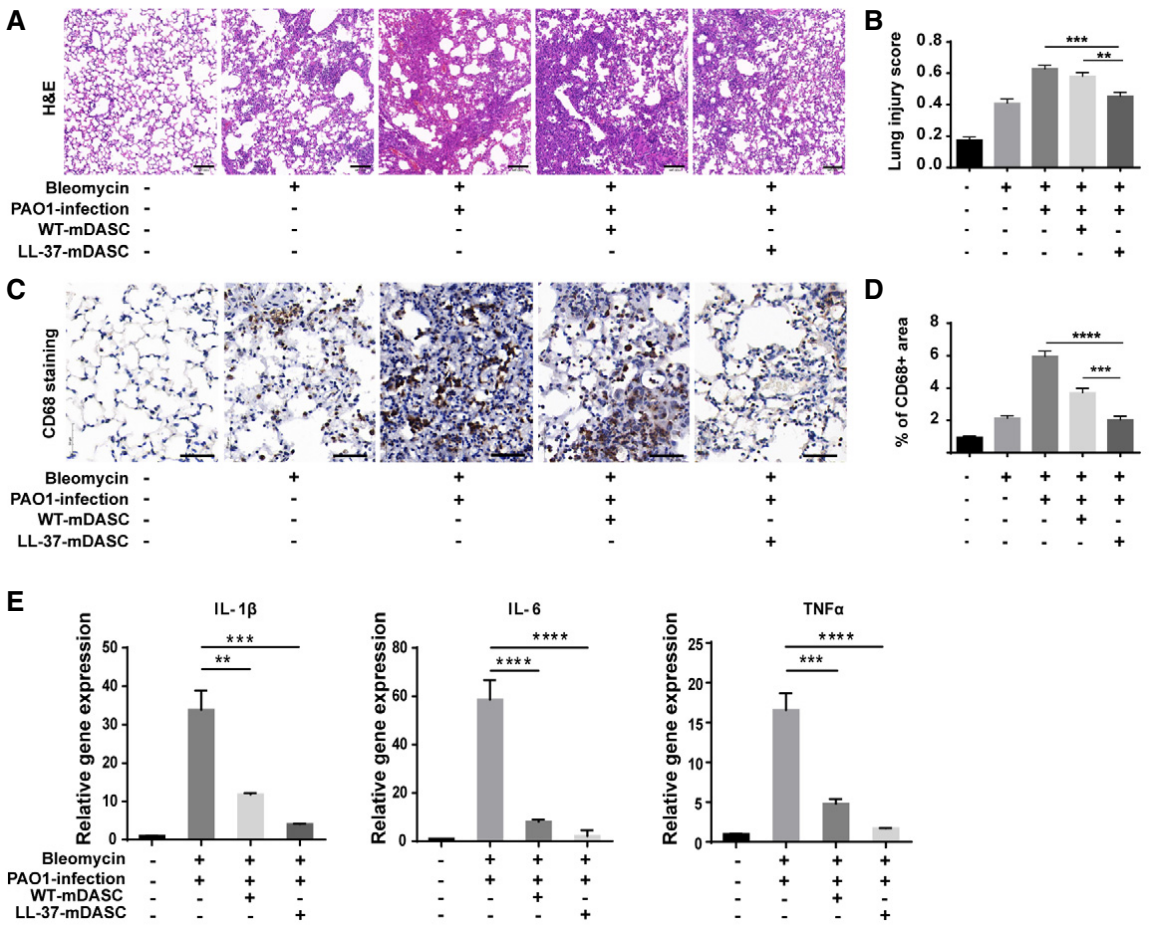
LL‐37‐mDASCs transplantation protected infected mouse from pulmonary inflammation AH&E staining showing histology of injured lung infected by PAO1 with WT‐mDASCs or LL‐37‐mDASCs transplantation. Scale bar, 100 μm.BHistopathological examination according to the lung injury scoring system based on blinded expert judgment. *n* = 5 mice per group. Error bars, SEM.CCD68 immunochemistry (brown) in infected lung with WT‐mDASCs or LL‐37‐mDASCs transplantation. Scale bar, 50 μm.DQuantification of brown‐stained (CD68^+^) area by Image J software. *n* = 5. Error bars, SEM.EGene expression level of indicated pro‐inflammatory cytokines of lung infected by PAO1 with WT‐mDASCs or LL‐37‐mDASCs transplantation. *n* = 3. Error bars, SEM.Data information: Statistics for graphs: one‐way ANOVA followed by Tukey's test. **P < 0.01; ***P < 0.001; ****P < 0.0001.Source data are available online for this figure.

Two days after infection, we investigated the effects of LL‐37‐mDASCs on histopathological changes in lung injury (Fig 6A and B for PAO1 infection and Fig EV2G for *E. coli* infection). Intratracheal bleomycin administration caused acute alveolar damage and inflammation aggravated by bacterial infection. However, the destructive changes to the lung were significantly attenuated by LL‐37‐mDASCs and lung injury scores were significantly decreased, compared to the WT‐mDASC treated mice. Levels of CD68 protein, a marker for macrophages, were also examined by immunohistochemistry (Fig 6C and D). The data showed an increase of CD68 expression in lung tissues following bleomycin + PAO1 treatment, which was significantly alleviated by treatment with LL‐37‐mDASCs. Real time qPCR analysis of major pro‐inflammatory cytokines including IL‐1β, TNF‐α, and IL‐6 showed a less severe inflammatory response in the LL‐37‐Lung after infection (Fig 6E).

### LL‐37‐expressing human DASC engraftment protects bioengineered artificial lung from bacterial infection

Following on from our demonstration that DASCs can be successfully engrafted into native lung, we next assessed engraftment into a bioengineered artificial lung. Bioengineered artificial lungs are a highly promising alternative organ source for clinical lung transplant surgery, given that suitable, autologous source seed cells are used to reconstruct functional epithelium and avoid immune rejection (Petersen *et al*, 2010). Furthermore, infection before and after organ transplant surgery is a major hindrance to successful organ transplants, and development of an anti‐infective bioengineered organ may provide a functional alternative.

**Figure 7:**
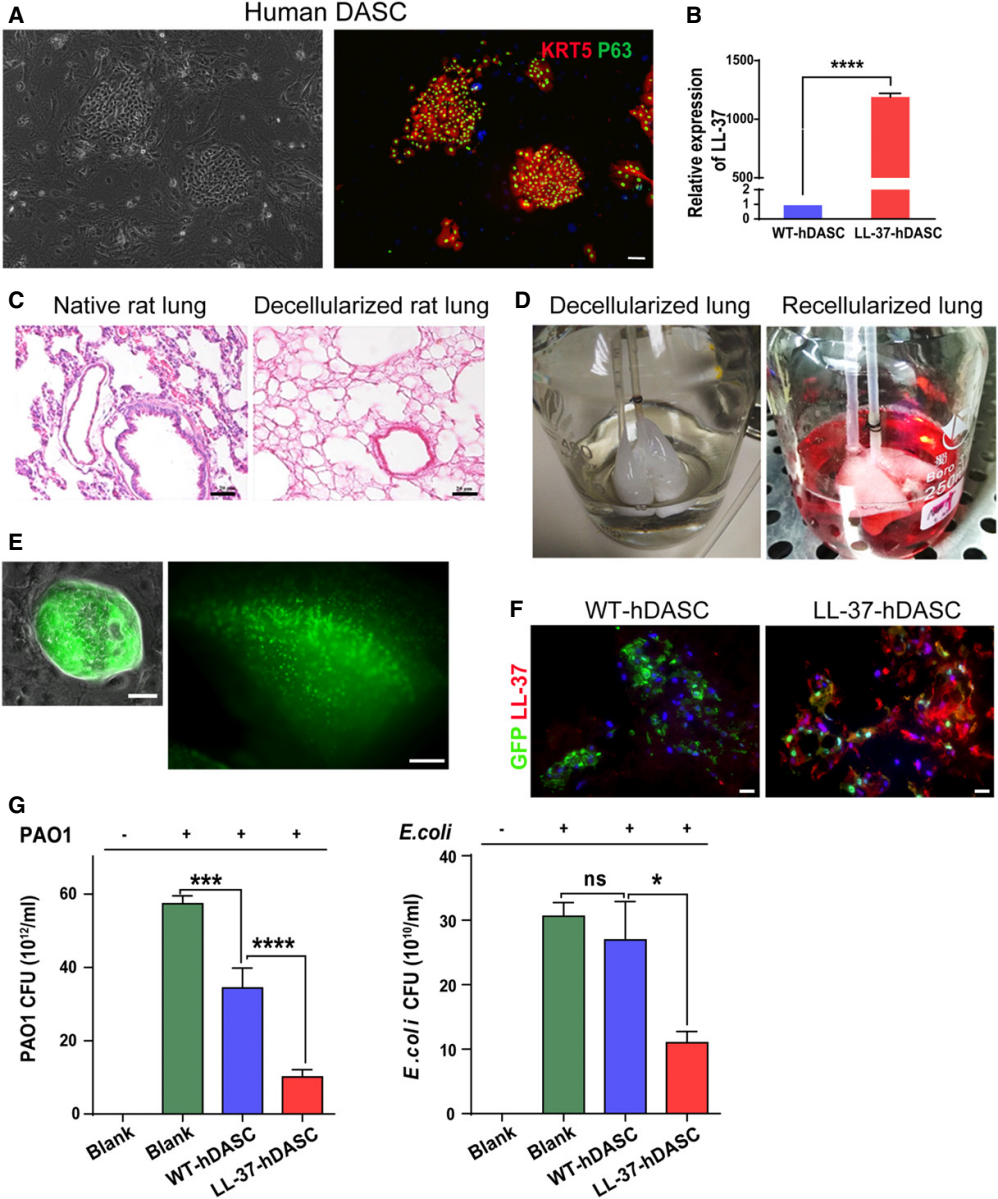
Lung scaffold recellularized by LL‐37 engineered human DASCs has enhanced anti‐bacterial ability AAnti‐Krt5 (red) and anti‐P63 (green) immunofluorescence of isolated hDASCs. Scale bar, 90 μm.BThe mRNA expression levels of LL‐37 were measured by real‐time qPCR for WT‐ and LL‐37‐hDASCs. *n* = 5. Error bars, SEM.CH&E staining of native rat lung tissue and decellularized scaffold showed the absence of nuclei following decellularization. Scale bar, 40 μm.D
*Ex vivo* biomimetic culture of LL‐37‐hDASCs recellularized lungs with constant media perfusion.EDirect fluorescence image showing GFP‐labeled LL‐37‐hDASCs before (left) and 7 days after (right) being engrafted onto the decellularized scaffold. Scale bar, 50 μm (right panel) and 2,000 μm (right panel).FImmunofluorescence of recellularized lung with indicated antibodies. Blue color indicates nuclear DAPI staining. Scale bar, 50 μm.GThe recellularized lung by LL‐37‐hDASCs displayed growth inhibitory effect on PAO1 and *Escherichia coli*. Initial dose of bacteria was 2 × 10^4 ^CFU. Culture duration, 24 h. *n* = 6. Error bars, SEM.Data information: Statistics for graphs: unpaired two‐tailed *t*‐test (B) and one‐way ANOVA followed by Tukey's test (G). *P < 0.05; ***P < 0.001; ****P < 0.0001.Source data are available online for this figure.

**Figure EV3:**
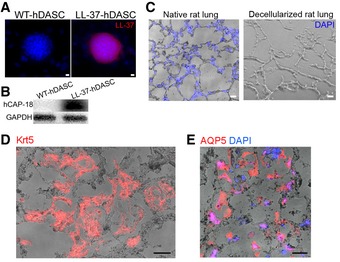
Lung scaffold recellularized by engineered human DASCs A, BThe expression of LL‐37 in engineered human DASC (hDASCs) was detected by immunostaining (A) and Western blot (B). Scale bar, 20 μm.CCryosections of native and decellularized rat lung with nuclei counterstain. Blue color indicates nucleus DAPI staining Scale bar, 50 μm.DImmunofluorescence staining showed that major cells preserved KRT5 + hDASCs phenotype (red). Scale bar, 50 μm.EImmunofluorescence staining showed that a few grafted cells of elongated shape had AQP5 (type I alveolar cell marker) expression. Scale bar, 50 μm.

To test the feasibility of artificial lung construction with human DASCs (hDASCs), we isolated adult hDASCs from the sixth‐order airway of bronchiectasis patients by bronchoscopic brushing followed by large‐scale cell expansion on irradiated 3T3‐J2 feeder cells (Hackett *et al*, 2011; Ma *et al*, 2018; Fig 7A). The LL‐37 expression level in wild‐type hDASCs was barely detectable. A lentiviral system was used to stably overexpress LL‐37 up to a thousand‐fold (Figs 7B, and EV3A and B). We expanded the engineered hDASCs as homogeneous, immature clones to indefinite numbers *in vitro* and achieved approximately 100 million cells in < 3 weeks.

To construct bioengineered artificial lungs, we harvested rat lungs and decellularized them by airway perfusion, as previously reported (Gilpin *et al*, 2016). Complete removal of cellular components and proper preservation of extracellular matrix were demonstrated by morphologic and cell nucleus analyses (Figs 7C and EV3C). GFP‐labeled WT‐ and LL‐37‐hDASCs were then delivered into the lung through the trachea by gravity to repopulate the scaffold and maintained the recellularized lung in *ex vivo* biomimetic culture for 7 days (Fig 7D). Large‐scale cellular engraftment was detected by direct GFP fluorescence observation (Fig 7E). Immunofluorescence staining showed that most cells preserved the KRT5^+^ hDASC phenotype (Fig EV3D). However, we found that a few grafted cells acquired an elongated shape with AQP5 marker expression, suggesting the gradual maturation of these stem cells into type I alveolar cells (Fig EV3E).

Substantial LL‐37 expression was detectable in LL‐37‐hDASC recellularized lungs (Fig 7F). Successfully recellularized lungs were co‐cultured with PAO1 or *E. coli* to examine their inhibiting growth ability. The results showed, compared to wild‐type ones, that the LL‐37‐hDASC recellularized lungs had improved bacterial clearance ability (Fig 7G). Collectively, the above work proposes a novel genetic and stem cell‐based therapeutic strategy for the treatment of recurrent, antibiotic‐resistant pulmonary infections.

## Discussion

Here, we have demonstrated that constitutive expression of LL‐37 peptide can protect the lung from bacterial infection, and transplantation of LL‐37‐expressing DASCs can be used to regenerate lung with enhanced host defense capability. Considering that respiratory infection has become the leading reason for human death and the number of antibiotic‐resistant cases is constantly increasing (Theuretzbacher & Toney, 2006), the concept of host defense augmentation is an attractive approach with great clinical potential (Finlay & Hancock, 2004; Spellberg *et al*, 2004; Easton *et al*, 2009). LL‐37 plays a key role in inhibiting the formation of *P. aeruginosa* biofilm at lower concentrations and directly degrades biofilms at higher concentrations (Dean *et al*, 2011). Moreover, the anti‐infection mechanism of LL‐37 *in vivo* is likely to be more complex than simple direct microbicidal effects. Systematic administration of LL‐37 protects mice from pulmonary bacterial infection (Beaumont *et al*, 2014), and transfer of LL‐37 restored bacterial killing in a cystic fibrosis xenograft model (Bals *et al*, 1999a). Systemic expression of LL‐37/hCAP‐18 after intravenous injection also resulted in improved survival rates following intravenous injection of lipopolysaccharide with galactosamine or *E. coli* (Bals *et al*, 1999b).

Wild‐type mice challenged with *P. aeruginosa*, pro‐inflammatory cytokines, and chemokines, including Il‐6, Il‐1, and Cxcl1, were significantly recruited. Yet, infection and inflammation of LL‐37^+/+^ mice were reduced, as confirmed by transcriptomic analysis. Both Wnt and FGF signals, recognized as key factors in pulmonary development and regeneration (Shu *et al*, 2005; Zacharias *et al*, 2018), were also activated in infected LL‐37^+/+^ mice. We used DASCs as a vehicle for LL‐37 antimicrobial peptide to achieve improved delivery. The incorporated DASCs itself could rapidly establish the epithelium barrier, which seems to have protective effects on pulmonary infection, while LL‐37‐DASCs have even stronger protective effects than WT‐DASC. As DASCs will not incorporate into healthy pulmonary tissue or other tissues or organs, our current strategy ensured the localized distribution of LL‐37‐DASCs to injured lung foci, where they can concentrate to combat local pulmonary infection with little or no side‐effects on other healthy tissue or organs. Meanwhile, LL‐37‐DASCs avoid limitations such as fast degradation following direct application of synthetic LL‐37. At the same time, we have shown that overexpression of LL‐37 impacts neither the viability of host cells nor the general health of LL‐37 transgenic mice. All the above data show the evidence that LL‐37‐DASCs provide a promising therapeutic exploration for lung infection. However, the potential risk associated with longer‐term release of LL‐37 *in vivo* needs to be fully investigated as well.

We also used LL‐37‐hDASCs to recellularize lung scaffolds and successfully established an anti‐infective bioengineered lung. It should be noted that the bioengineered lung built in the current work is only conceptual and could not contribute to recipient pulmonary function, even if transplanted, due to lack of proper vasculature. However, it demonstrated an important proof of concept, namely, that patient‐specific DASCs could be isolated and genetically engineered to build artificial organs with demanding biological functions.

In addition to DASCs, many other distinct populations of adult lung stem/progenitor cells have been reported to exert regenerative functions under different circumstances (Barkauskas *et al*, 2013; Leeman *et al*, 2014; Nabhan *et al*, 2018). Among them, only DASCs were tested in the current study, as such cells were easily expanded, genetically manipulated, and transplanted into the damaged lung. We did observe a fraction of transplanted LL‐37‐DASCs differentiating into mature alveolar cells *in vivo*. However, even undifferentiated, the engrafted LL‐37‐DASCs could rapidly establish an epithelial barrier to prevent the spread of bacteria and inflammation while maintaining the local antimicrobial peptide concentration. Once the bacteria were gradually cleared by LL‐37 and other immune factors, the improved microenvironment could further accelerate LL‐37‐DASC maturation process. We anticipate that similar genetic engineering strategies can also be applied to other lung stem/progenitor cell types—including but not limited to type II alveolar cells—that might exert synergistic effects on lung repair following transplantation.

In clinical practice, genetically engineered cells are being recognized as powerful “living drugs” for treating many previously uncured diseases. For example, substantial advances have been made in the field of T‐cell‐mediated cancer therapy. Based on this technique, T cells are genetically modified by viral vector transduction that either alter T‐cell receptor (TCR) specificity or introduce antibody‐like recognition into chimeric antigen receptors (CARs) (Sadelain *et al*, 2017). The successful application of genetically engineered T cells is encouraging the field to develop more gene‐ and cell‐based therapies. As LL‐37 peptide has already demonstrated safety and efficacy in human infectious disease treatment (Gronberg *et al*, 2014) and human DASC transplantation was also previously tested in a pilot clinical trial (Ma *et al*, 2018), we think their combination could lead to a promising practical therapy with great clinical potential for those life‐threatening lung infections.

## Materials and Methods

### Animals and bacteria

Female C57/B6 mice and wild‐type FVB mice (6–8 weeks) weighing 16–18 g were purchased from Shanghai SLAC Laboratory Animal Co., Ltd. (China). LL‐37^+/+^ mice (Fvb background) were produced by Cyagen Biosciences Inc. (China). Male Sprague‐Dawley rats, weighing 180–220 g each were purchased from Shanghai SLAC Laboratory Animal Co., Ltd. (China). All mice were housed in specific pathogen‐free conditions within an animal care facility (Center of Laboratory Animal, Tongji University, Shanghai, China) until euthanization. All animals were cared for in accordance with NIH guidelines, and all animal experiments were performed under the guidance of, and with approval from, the Institutional Animal Care and Use Committee of Tongji University. *Pseudomonas aeruginosa* (ATCC‐BAA‐47; strain HER‐1018/PAO1) and *E. coli* strain DH5‐α (ATCC‐98489) were used in these experiments.

### Generation of LL‐37^+/+^ transgenic mice

All animal experiments were performed according to guidelines approved by the Tongji University Association for Laboratory Animal Science. To understand whether the expression of LL‐37 is beneficial *in vivo*, we constructed a transgenic mouse that expressed the human CAMP gene. To begin with, we constructed the LL‐37‐expressing plasmid. Full‐length human CAMP cDNA (NCBI: NM_004345.4) was inserted into an IRES vector with a GFP expression sequence under a constitutively active EF‐1α promoter. Next, fertilized eggs obtained from FVB background mice were injected with the pIRES human LL‐37 expression plasmid described above and then implanted into FVB females to generate LL‐37 founders. Then LL‐37^+/+^ mice were identified using genotyping PCR for LL‐37 gene expression, which was performed on tails that were obtained from mice aged 3–4 weeks old. PCR was performed using the following primers: LL‐37 F: 5′‐AGCAGTCACCAGAGGATTGT‐3′ and LL‐37 R: 5′‐GGCACACACTAGGACTCTGT‐3′. The PCR product produced by LL‐37^+/+^ mice was 135 bp long. LL‐37 protein expression was analyzed using Western blotting analysis. PCR‐negative littermates and wild‐type (FVB) mice were used as controls.

### Tissue histology and immunohistochemistry

For mouse lung histology analysis, mice were euthanized and the diaphragm was carefully cut to open without touching the lung. Lung was inflated with 3.7% formaldehyde (Sigma, USA) using a 30G needle through trachea. Then the lung was dissected and fixed in 3.7% formaldehyde at 4°C overnight before paraffin section or cryosection.

For cryosection, the fixed lung was settled by 30% sucrose before embedding into the Tissue‐Tek O.C.T compound (Sakura, Japan), solidified on dry ice, and cut using cryotome (Leica Microsystems, Germany) into 5‐ to 10‐μm thickness. For paraffin section, the lung was dehydrated by gradient ethanol in an automatic tissue processer and then embedded into paraffin blocks. The blocks were cut into 5‐ to 7‐μm thickness by using microtome (Leica Microsystems, Germany) at distinct planes. The sections were placed on polylysine coated glass slides and stored at room temperature until further use. Hematoxylin and eosin (H&E) staining was performed following standard protocol. The lung injury score was calculated as previously described (Matute‐Bello *et al*, 2011). For immunostainings, sections were deparaffinized, rehydrated, and blocked by 0.3% H_2_O_2_ for 30 min. Antigen retrieval was performed by treating the slides in citrate buffer in a microwave oven for 10 min. The slides were incubated for 1 h with normal goat serum, and then incubated in a moist chamber with anti‐CD68 (ab125212, Abcam) antibody at 4°C overnight. After a complete wash in phosphate‐buffered saline (PBS), the tissues were incubated in biotin‐labeled goat anti‐mouse antibody for 30 min and rinsed with PBS, and incubated with avidin–biotin–peroxidase complex for 30 min at 37°C. The signal was detected using diaminobenzidine (DAB). Brown‐stained area (CD68^+^ cells in the monocyte lineage) was separately quantified by Image J version 1.52a.

### Immunofluorescence

For immunofluorescence staining, cells or tissues were fixed by 3.7% formaldehyde, and then incubated with 0.2% Triton X‐100 to improving the cell permeability for 10 min. Paraffin‐ or cryo‐embedded tissues were sectioned and subjected to antigen retrieval in citrate buffer (pH 6.0, Sigma, USA) in microwave oven for 20 min before staining. Normal donkey serum at 10% concentration (Jackson Immuno Research) was used to block the non‐specific antigen. Primary antibodies used in this work include DASC markers: KRT5 (1:200, EP1601Y, Thermo and ab128190, Abcam); P63 (deltaN,1:200, 4A4, Abcam); pneumocyte markers like AQP5 (1:200, EPR3747, Abcam); and others like GFP (1:200, B‐2, Santa Cruz), GFP (1:1,000, ab5450, Abcam), GFP (1:1,000, ab290, Abcam), and LL‐37/cathelicidin (1:200, ab69484 and ab80895, Abcam). Alexa Fluor‐conjugated Donkey 488/594 (1:200, Life Technologies, USA) was used as secondary antibodies. After counterstaining with DAPI (Roche, USA), samples were treated with 0.1% Sudan Black (Sigma, USA) for 1–2 min to remove autofluorescence and then mounted with VECTASHIELD^®^ Mounting medium (Vector labs, USA). Stained slides were stored at 4°C in the dark and images were taken using fluorescence microscope (Nikon 80i and Eclipse Ti, Nikon, Japan).

### RNA sequencing analysis and bioinformatics

For RNA sequencing, lungs were dissected in ice‐cold phosphate‐buffered saline and lungs from FVB/LL‐37^+/+^ or GFP^+^ lobe from WT‐Lung/LL‐37‐Lung were dedicated for RNA isolation. Total RNA was extracted using TRIzol Reagent (Invitrogen, LifeTechnologies, USA) following the manufacturer's instructions, and then treated with DNase I (Invitrogen, Life Technologies, USA). The RNA integrity was verified using an Agilent 2100 BioAnalyzer (Agilent, USA). The cDNA library construction and sequencing were performed using BGISEQ‐500 platform. High‐quality reads were aligned to the mouse reference genome (UCSCmm10) using Bowtie2 (v2.2.5). The expression levels for each of the genes were normalized to fragments per kilobase of exon model per million mapped reads (FPKM) using RNA‐Seq by Expectation Maximization (RSEM) (v1.2.12), and the common genes among samples were displayed by Venn Charts. NOISeq method was used to screen differentially expressed genes. Cluster and java Treeview were used to perform clustering analysis of the DEGs. The threshold for significant differential expression was based on the Poisson distribution (Fold Change ≥ 2.00 and FDR ≤ 0.001). Using the cor function in R, we calculated the Pearson correlation coefficient (PCC) between each of the two samples. Gene Ontology (GO) enrichment analysis of differentially expressed genes was implemented by the phyper function in R. With the KEGG annotation result, we classified DEGs according to official classification, and we also performed pathway functional enrichment using phyper, a function of R. We used DIAMOND (Buchfink *et al*, 2015) to map the DEGs onto the STRING (von Mering *et al*, 2005) database to obtain the interaction between DEG‐encoded proteins using homology with known proteins. For the entire interaction result, we provided an input file that could be imported directly into Cytoscape for network analysis.

### Isolation and culture of mouse and human DASCs

Mouse DASC cells were isolated and cultured as previously described (Zuo *et al*, 2015). Briefly, lung tissue was collected from adult mice and immersed in cold wash buffer (F12 medium, 1%Pen/Strep, 5% FBS). The main bronchi were removed from the lungs and the lobes were cut with sterile surgical blade into small pieces and digested with dissociation buffer (F12/DMEM, 1 mg/ml protease, 0.005% trypsin, and 10 ng/ml DNaseI) over night with gentle rocking. Dissociated cells were passed through 70‐μm nylon mesh and then washed twice with cold F12 medium.

To isolate human DASCs, bronchoscopy procedure for lung sampling was performed by board‐certified respiratory physicians using a flexible fiber‐optic bronchoscope (Olympus, Japan). Before bronchoscopy, oropharyngeal and laryngeal anesthesia was obtained by administration of 2 ml of nebulized 4% lidocaine, followed by 1 ml of 2% topical lidocaine sprayed into the patient's oral and nasal cavities. After the bronchoscope was advanced through the vocal cords, 2 ml of 2% lidocaine solution was instilled into the trachea and both main bronchi through the working channel of the bronchoscope. Then a disposable 2‐mm brush was advanced through the working channel of the bronchoscope and used to collect airway epithelial cells by gently gliding the brush back and forth two times in 4–6 order bronchi in the right or left lobe. To isolate the human DASCs, the brush with samples were cut with scissors into 1‐cm pieces. After removing sputum, the brush pieces were directly digested with dissociation buffer described above. Specimens were incubated at 37°C for an hour with gentle rocking. Dissociated cells were passed through 70‐μm nylon mesh and then washed twice with cold F12 medium. All the human tissues were obtained following clinical SOP under patient's consent and approved by Shanghai East Hospital Ethics Committee (Shanghai, China). Informed consent was obtained from all subjects and all the experiments conformed to the principles set out in the WMA Declaration of Helsinki and the Department of Health and Human Services Belmont Report.

Mouse or human cells were then plated onto feeder cells in DASC culture medium including DMEM/F12, 10% FBS (Hyclone, Australia), Pen/Strep, amphotericin, and growth factor cocktail as previously described. Under 7.5% CO_2_ culture condition, the DASC colonies emerged 3–5 days after plating, and were digested with 0.25% trypsin‐EDTA (Gibco, USA) for 3–5 min for passaging. Typically, DASCs are passaged every 5–7 days and split at 1:7 ratio.

For anchorage‐independent growth assay, 1 × 10^5^ cells were seeded in 1 ml of a 0.375% upper agar (Sigma) layer on 0.5% under agar layer in the DMEM supplemented with 10% FBS. Cultures were usually maintained for 14 days, and then gels were stained by crystal violet‐methanol solution (Solarbio).

To express LL‐37 in stem cells, full‐length human CAMP cDNA was cloned from human genome into pHIV‐EGFP plasmid vector. Then pHIV‐CAMP‐EGFP was used to generate lentiviral particles in 293T cells, which was transduced into DASC in combination of 10 μg/ml polybrene. pHIV‐EGFP lentivirus was transduced for control purpose. The cell viability of cells was assessed by MTT Cell Proliferation and Cytotoxicity Assay Kit.

### *In vitro* antimicrobial assay

Assessment of direct inhibition of bacterial growth by LL‐37‐DASC or its conditioned medium (CM), which had been incubated with cells for 24 h, was done by counting CFU. Cells or CM in 96‐well plates (approx. 1 × 10^4^ cells per well) within culture medium without antibiotics and FBS were pre‐incubated with 1 μg/ml anti‐LL‐37 antibody (HM2070, Hycult biotech) or mouse isotype antibody control (B30010M, Abmart) for 2 h, and then co‐cultured with *P. aeruginosa* or *E. coli* in a humidified CO_2_ incubator. Aliquots of culture medium were taken from each well and serially diluted with sterile PBS. Then they were brought onto a solid medium and evenly spread over the LB‐agar surface. After incubation, the number of colonies present on the agar surface was counted, and the number of CFU in the sample was then calculated. For BALF antimicrobial assay, 0.5 ml BALF/per mouse was collected using standard protocol and then processed by Ultra‐4 10K Centrifugal Filter Device (Amicon, Millipore) at 4,000 × *g* for 60 min at 4°C to collect the ultrafiltrate and retentate. About 1 ml of ultrafiltrate or retentate was diluted with equal volume of LB medium and used for 6‐h bacterial suspension culture, followed by overnight agar culture and CFU counting.

### 3D organoid culture of DASC

Three‐dimensuional organoid culture of mouse DASCs was performed on Matrigel matrix (Corning, USA) as previously described. Cells were first cultured in culture medium for 2 days and then transferred to serum‐free DMEM/F12 medium supplemented with FGF10 (50 ng/ml, Peprotech, USA), Transferrin (5 μg/ml, Peprotech, USA), HGF (20 ng/ml, Peprotech, USA), and 5% BSA for 5 days to induce distal lung differentiation. After sphere formation and differentiation, the organoids were harvested, embedded in Tissue‐Tek O.C.T. Compound (Sakura, USA), and frozen in dry ice and ethanol mixture. For analysis, 5‐μm sections were obtained and subjected to immunofluorescence for the indicated mature pulmonary markers, followed by counterstaining with DAPI.

### DASC transplantation

Mouse lung was injured by intratracheally instilling with 3 U/kg body weight of bleomycin (Selleckchem, USA) 7 days prior to transplantation. Then mice were anesthetized by isoflurane and rested on a stand. One million GFP‐labeled DASCs were suspended in 50 μl of PBS and used for transplantation of each mouse. Intratracheal aspiration was performed by injecting the cells into trachea via mouth. Bright‐field and direct fluorescence images of the transplanted lung were acquired under the fluorescence stereomicroscope (MVX10, Olympus, Japan). Engraftment ratio was then calculated by the area ratio of GFP fluorescence in the lung tissue.

### PAO1 and *Escherichia coli* infection mouse model

*Pseudomonas aeruginosa* (PAO1, ATCC 9027) and the *E. coli* strain DH5‐α were used to model lung infection in mice as previously described (Torres *et al*, 2018). Bacterial concentrations were validated by plating on LB‐agar and counting colony‐forming units (CFU). Before each experiment, the bacterial samples were washed twice and resuspended in PBS. Mice were anesthetized with isoflurane and PAO1 or *E. coli* (5 × 10^6^ CFUs) were intratracheally instilled into mouse lung. To evaluate whether LL‐37‐mDASCs show enhanced host defense *in vivo*, mice were treated with 3 U/kg body weight of bleomycin (Selleckchem, USA) 7 days in advance and then they were instilled with bacteria and mDASCs (1 × 10^6^) in a 30 μl total volume. Intratracheal aspiration was performed by instilling the bacteria and cells into the trachea via mouth, which was described in previous publications (Shi *et al*, 2019). Two days post‐infection, mice were sacrificed, and the lung samples and BALFs were collected for analysis. To collect BALF, a 20‐gauge catheter was placed into the mouse trachea through which 1 ml of cold PBS was flushed back and forth three times. Aliquots of BALF were diluted and cultured on LB‐agar plate for 16 h at 37°C before CFU counting. For lung homogenate harvest, the pulmonary lobes with the stem cell incorporation were separated under the stereomicroscope and then homogenized under sterile conditions. Aliquots of homogenate were diluted and cultured on LB‐agar plate for 16 h at 37°C for CFU counting.

### Arterial blood gas measurement

Mice were anesthetized and the blood samples were drawn from the carotid aorta into polypropylene syringes containing 60 IU of dry, electrolyte‐balanced heparin (PICO70; Radiometer Medical, Copenhagen, Denmark). Partial oxygen pressure (pO2), partial carbon dioxide pressure (pCO2), and oxygen saturation (sO2) were measured by using ABL90 Flex Blood Gas Analyzer (Radiometer Medical).

### Decellularization and recellularization of the rat lung

Rat lungs were harvested from male Sprague‐Dawley rats and decellularized by perfusion of 0.1% SDS solution and 1% TritonX‐100 solution through the trachea at 15 rpm, followed by washing. For recellularization, GFP‐labeled WT‐hDASCs and LL‐37‐hDASCs (1 × 10^6^) were delivered to rat lung scaffold, respectively, in 10 ml of media by gravity. Constant media perfusion of serum‐free DMEM/F12 medium supplemented with FGF10 (50 ng/ml; Peprotech, USA), Transferrin (5 μg/ml; Peprotech, USA), HGF (20 ng/ml; Peprotech, USA), 2% Matrigel, and 5% BSA through the pulmonary artery was maintained at 4 ml/min and changed daily. Recellularized lungs were maintained in culture for 7 days. For antimicrobial assay, recellularized lung lobes were dissected and co‐cultured with PAO1 or *E. coli* (2 × 10^4^ CFU) in 24‐well plates and incubated at 37°C for 24 h, while bacteria were cultured with medium in the control group. Then aliquots of culture medium were diluted and plated on LB‐agar plates for bacterial colony number counting.

### Real‐time qPCR

RNA was isolated from cells or tissue, using the RNeasy mini kit with DNase digestion according to the manufacturer's instructions (Qiagen). RNA, the quality of which was assessed with SimpliNano (GE Healthcare), was reverse‐transcribed into cDNA with PrimeScript™ RT Master Mix (TaKaRa). The Q‐PCR was performed on an ABI 7500 real‐time PCR system (Applied Biosystems) under following conditions: 95°C for 2 min, then 40 cycles of 95°C for 10 s, and 60°C for 40 s. The relative expression level of genes was calculated using the 2^−ΔΔ*C*t^ method.

Primers: hCAP18/LL‐37: F 5′‐CACAGCAGTCACCAGAGGATTG‐3′, R 5′‐GGCCTGGTTGAGGGTCACT‐3′; Il‐1β: F 5′‐CAACCAACAAGTGATATTCTCCATG‐3′, R 5′‐GATCCACACTCTCCAGCTGCA‐3′; Il‐6: F 5′‐GAGGATACCACTCCCAACAGACC‐3′, R 5′‐AAGTGCATCATCGTTGTTCATACA‐3′; TNF‐α: F 5′‐CATCTTCTCAAAATTCGAGTGACAA‐3′; R 5′‐TGGGAGTAGACAAGGTACAACCC‐3′; β‐actin: F 5′‐TACCACCATGTACCCAGGCA‐3′, R 5′‐CTCAGGAGGAGCAATGATCTTGAT‐3′; GAPDH: F 5′‐CGGAGTCAACGGATTTGGTCGTAT‐3′, R 5′‐AGCCTTCTCCATGGTGGTGAAGAC‐3′.

### Western blotting

Cells or tissues were washed with cold PBS and lysed in RIPA buffer (CST) containing protease inhibitors cocktail (Roche) followed by standard Western blotting procedure. To detect the 4‐kD LL‐37 expression, tissue lysates were centrifuged through Ultra‐4 10K Centrifugal Filter Device (Amicon, Millipore) at 4,000 × *g* for 60 min at 4°C to collect ultrafiltrate whose mass was less than 10‐kD. After measuring protein concentration, samples were loaded and separated on 4–20% precast polyacrylamide gels, and then transferred to PVDF membranes (Roche) at 100 V for 10 min. Membranes were blocked with 5% no‐fat powdered milk, and then incubated with primary antibodies overnight, followed by secondary antibodies. The specific signals were detected by Immobilon Western Chemiluminescent HRP Substrate (Millipore) and Tanon image system. The following antibodies were used: LL‐37/cathelicidin (Santa Cruz, D‐5), GAPDH (ab8245, Abcam), and HRP‐conjugated anti‐mouse IgG(H+L) as secondary antibody (800151, Vazyme).

### Statistics

All statistical analyses were performed using GraphPad Prism 7 software. A significance threshold was set at *P* < 0.05. All experiments were performed in three independent triplicates at least. *P*‐values are provided in Appendix Table S1. *P < 0.05; **P < 0.01; ***P < 0.001; ****P < 0.0001.

## Author contributions

JFX, WZ, and JMQ designed the study; JFX and WZ supervised the study; YQZ, YS (Yun Shi), LY, YFS (Yufen Sun), and YFH performed experiments; YJW, YL, YM, and FGJ assisted with the experiments; TZ, TR, and ZXZ contributed new reagents or analytic tools; WZ, JFX, YS, YQZ, and LY drafted the manuscript; JFX, WZ, JMQ, TPD, and NRF provided critical discussion and edited the paper.

## Conflict of interest

The authors declare that they have no conflict of interest.

**The paper explained**

**Problem**

Recurrent lung infections lead to chronic inflammation and additional lung tissue damage. Patients’ recovery cannot always be achieved with antibiotics. Therefore, new therapeutic strategies are needed to fight pulmonary bacterial infections and subsequent lung injuries.

**Results**

Using a novel transgenic rodent model, we showed that specific expression of LL‐37 in mouse distal airway stem cells (DASCs) allowed antimicrobial properties to be exerted in the injured foci without affecting other healthy lung regions. Transplantation of LL‐37‐expressing DASCs regenerated alveolar tissue, and enhanced the lung bacterial clearance ability. Blood–air exchange function was increased. DASCs from human patients were isolated, expanded, and further engrafted into decellularized lung scaffold to generate antimicrobial artificial lung.

**Impact**

This study reports the combination of LL‐37 antimicrobial peptide and stem cell transplantation to reconstitute the epithelium barrier and increase the host defense system. In China, DASC transplantation has been tested in clinical trials with promising results. We propose to combine it with LL‐37 gene engineering in the context of lung injury diseases complicated by recurrent pulmonary infections.

## Supporting information



Appendix

Expanded View Figures PDF

Table EV1

Review Process File

Source Data for Figure 1

Source Data for Figure 2

Source Data for Figure 3

Source Data for Figure 4

Source Data for Figure 5

Source Data for Figure 6

Source Data for Figure 7

## Data Availability

The RNA‐Seq data are available at the Sequence Read Archive (SRA) https://www.ncbi.nlm.nih.gov/sra (Accesion no. PRJNA559606 and PRJNA559986).
