# Supplementary material for: Genetically engineered distal airway stem cell transplantation protects mice from pulmonary infection
Source: EMBO Mol Med. 2019 Nov 29;12(1):e10233. doi: 10.15252/emmm.201810233 (PMC6949487; doi:10.15252/emmm.201810233)
Supplement: Supplementary file 1 — Appendix [file EMMM-12-e10233-s001.pdf]

Genetically engineered distal airway stem cell transplantation protects mice from pulmonary infection

Yue-qing Zhou, Yun Shi, Ling Yang, Yu-fen Sun, Yu-fei Han, Zi-xian Zhao, Yu-jia Wang, Ying Liu, Yu Ma, Ting Zhang, Tao Ren, Tina P. Dale, Nicholas R. Forsyth, Fa-guang Jin, Jie-ming Qu, Wei Zuo, Jin-fu Xu

Appendix

Appendix Table S1

Appendix Table S1: Statistics.

Figure	p-Value	n
Fig 1C	0.0033(Top pannel)；0.4231(Down pannel)	3
Fig 1D	>0.9999(0h);<0.0001(6h); 0.0094(24h);	3
Fig 1F	> 0.9999(0h); 0.0204(6h);	3
Fig 1G	IL-1β: 0.0002(6h); 0.0356(24h)<br>IL-6: < 0.0001(6h);	n≥3
Fig 3B	<0.0001	10
Fig 3E	P0-P5: 0.9978; 0.9990; > 0.9999; > 0.9999; > 0.9999	6
Fig 3G	0.3038(MOI=1); 0.0023(MOI=5); 0.0028(MOI=10)	4
Fig 3H	< 0.0001(18h)	3
Fig 3I	< 0.0001 (column C-D)； 0.0018 (columnD-E)	4
Fig 3J	< 0.0001 (column C-D)； 0.0252 (columnD-E)	3
Fig 4B	0.0474(WT-lung, 7d vs 21d)	3
Fig 5B	<0.0001(6h); <0.0001(24h);<0.0001(48h)	3
Fig 5C	<0.0001(6h)	3
Fig 5F	0.0415(PCO2); 0.0027(SO2); 0.0006(PO2)	6
Fig 6B	0.0002(column C-E)；0.0064(column D-E)	5
Fig 6D	<0.0001(column C-E)；0.0004(column D-E)	5
Fig 6E	IL-6: < 0.0001 (column B-C); < 0.0001(column B-D)<br>IL-1b: 0.0013 (column B-C) ; 0.0002(column B-D)<br>TNF-a: 0.0003 (column B-C) ; < 0.0001(column B-D)	3
Fig 7B	<0.0001	5
Fig 7G	PAO1: 0.0002(column B-C) ; <0.0001(columnC-D)<br>E.coli: 0.8607(column B-C)； 0.0131(columnC-D)	6
Fig EV1C	0.0206(column C-D)	5
Fig EV1D	0.0003(column C-D)	3
Fig EV1F	0.0443(column C-D)	3
Fig EV2E	0.0011(Lung); 0.0287(BALF);	3
Fig EV2F	0.0075(PpO2); 0.0301(So2); 0.1859(PCO2)	3
