# Supplementary material for: Genetically engineered distal airway stem cell transplantation protects mice from pulmonary infection
Source: EMBO Mol Med. 2019 Nov 29;12(1):e10233. doi: 10.15252/emmm.201810233 (PMC6949487; doi:10.15252/emmm.201810233)
Supplement: Supplementary file 2 — Expanded View Figures PDF [file EMMM-12-e10233-s002.pdf]

Expanded View Figures

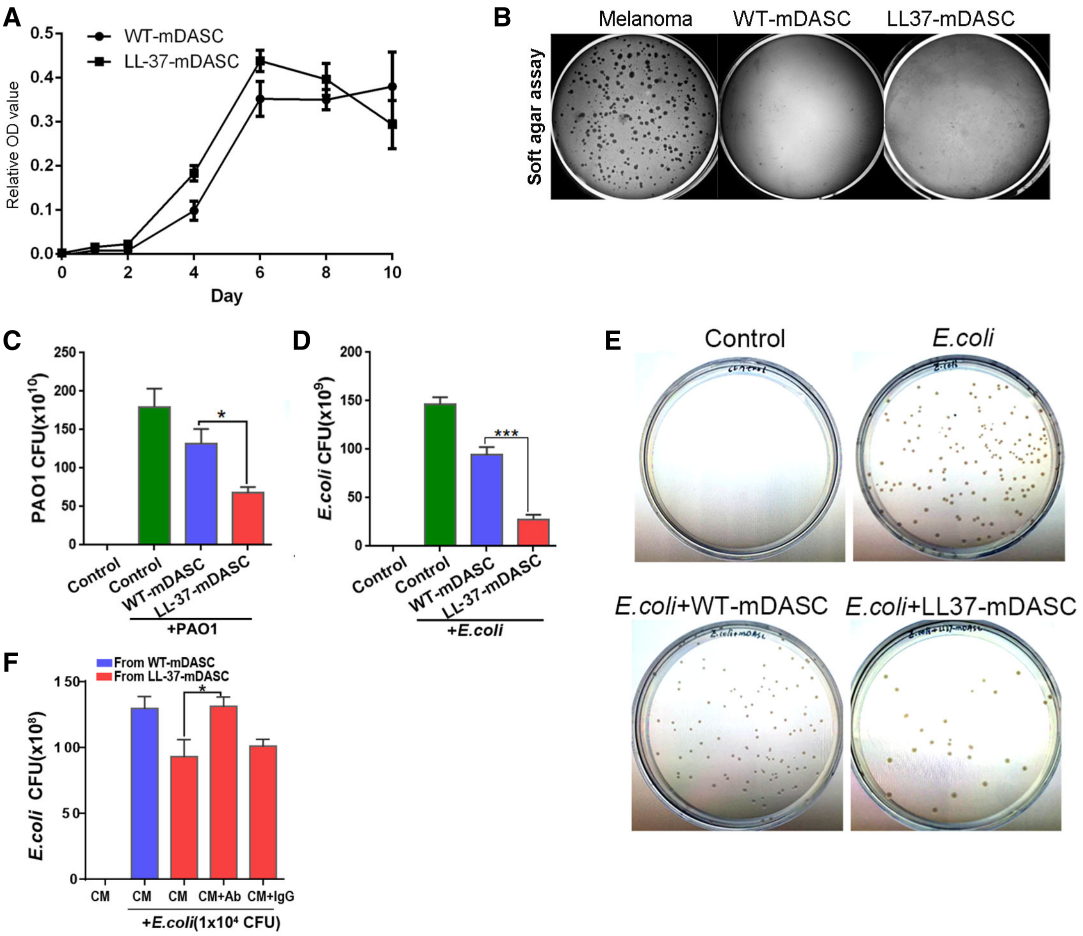

Figure EV1. The antimicrobial effect of LL-37-mDASCs *in vitro*.

A Cell growth curve of WT- and LL-37-mDASCs was measured by MTT assay. n = 3–5. Error bars, SD.

B Soft agar assay of WT- and LL-37-mDASCs. Mouse melanoma cell line was included as a positive control.

C Histogram showed that LL-37-mDASCs conditioned medium (CM) had potent growth inhibitory effect on PAO1. Initial addition of PAO1 was 1×10^4 CFU. n = 5. Error bars, SEM.

D Histogram shows that LL-37-mDASCs CM had potent growth inhibitory effect on *Escherichia coli*. Initial addition of *E. coli* was 1×10^4 CFU. n = 3. Error bars, SEM.

E Clone formation unit assay of *E. coli* following incubation with indicated cellular CM.

F Preincubation of CM with anti-LL-37 antibody, but not mouse IgG, reduced the antimicrobial effect of LL-37-mDASCs against *E. coli*. n = 3. Error bars, SEM.

Data information: Statistics for graphs: one-way ANOVA followed by Tukey's test. *$P < 0.05$; ***$P < 0.001$.

© 2019 The Authors

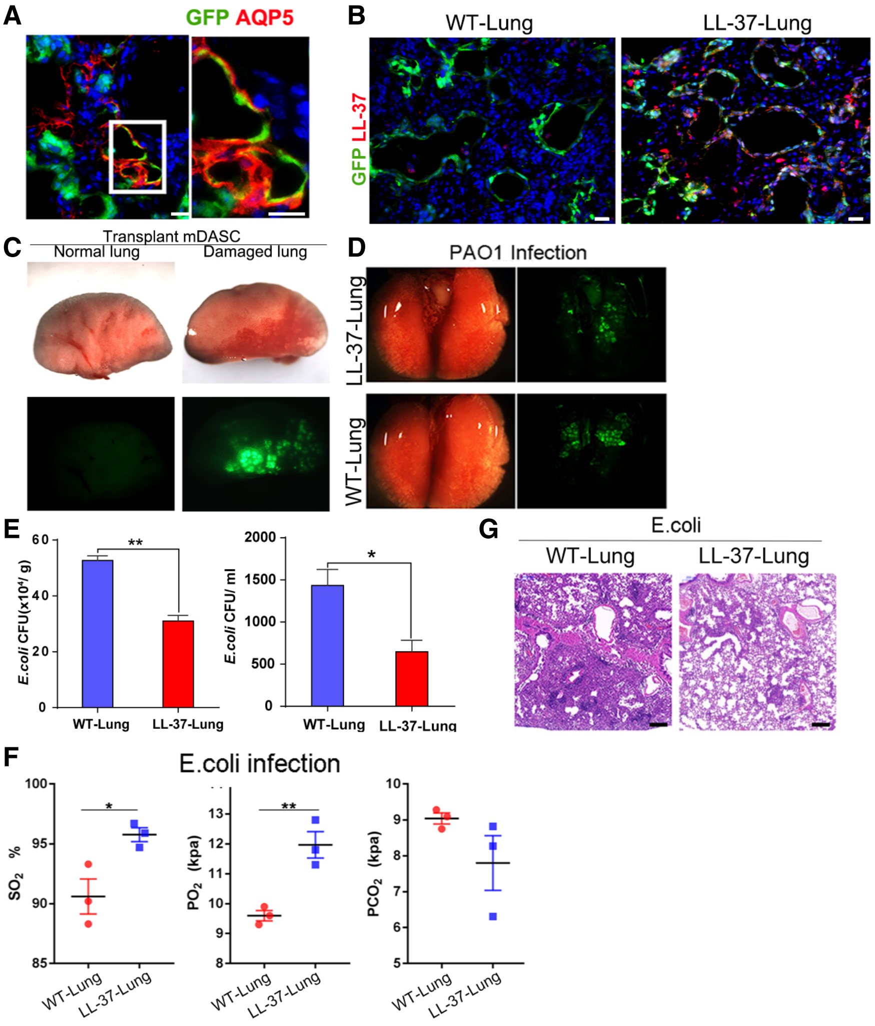

Figure EV2. The antimicrobial effect of LL-37-Lung.

A Left, immunostaining of engrafted LL-37-mDASCs cells with anti-GFP and anti-AQP5 (type I alveolar cell marker) antibodies; right, amplification of inset in upper panel indicated regenerated alveolar structure. Scale bar, 30 μm.

B Distribution of engrafted GFP-labeled cells in lung parenchyma by immunostaining 21 days after transplantation. WT-Lung, WT-mDASCs engrafted; LL-37-Lung, LL-37-mDASCs engrafted. Scale bar, 40 μm.

C Direct fluorescence image of lungs from normal mouse or bleomycin injured mouse 7 days after transplantation of 1×10^6 GFP-labeled mDASCs.

D Direct fluorescence image under stereomicroscope showing mouse lung transplanted with 1×10^6 GFP-labeled WT- and LL-37-mDASCs followed by PAO1 infection.

E Intratracheal instillation of equal amounts of *E. coli* (5×10^6 CFU per mouse) into WT-Lung (WT-mDASCs engrafted) and LL-37-Lung (LL-37-mDASCs engrafted) followed by bacterial CFU analysis in whole lung homogenates (left panel) and BALF (right panel) 2 days after infection. n = 3. Error bars, SEM.

F Arterial blood gas analysis of mice with WT-Lung and LL-37-Lung following *E. coli* infection 2 days after transplantation. n = 3. Error bars, SEM.

G H&E staining showing histology of WT-Lung and LL-37-Lung with *E. coli* infection after 2 days of cell transplantation. Scale bar, 200 μm.

Data information: Statistics for graphs: unpaired two-tailed *t*-test. $^*P < 0.05$; $^{**}P < 0.01$.

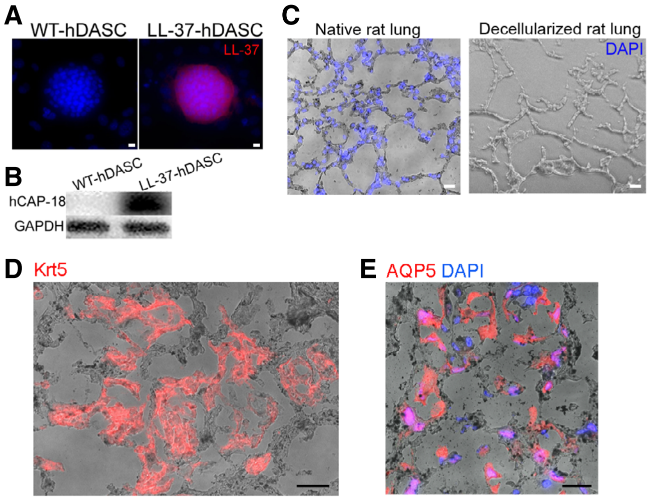

Figure EV3. Lung scaffold recellularized by engineered human DASCs.

A, B The expression of LL-37 in engineered human DASC (hDASCs) was detected by immunostaining (A) and Western blot (B). Scale bar, 20 μm.

C Cryosections of native and decellularized rat lung with nuclei counterstain. Blue color indicates nucleus DAPI staining Scale bar, 50 μm.

D Immunofluorescence staining showed that major cells preserved KRT5 + hDASCs phenotype (red). Scale bar, 50 μm.

E Immunofluorescence staining showed that a few grafted cells of elongated shape had AQP5 (type I alveolar cell marker) expression. Scale bar, 50 μm.

© 2019 The Authors
