# Supplementary figures and images for: Genetically engineered distal airway stem cell transplantation protects mice from pulmonary infection
Source: EMBO Mol Med. 2019 Nov 29;12(1):e10233. doi: 10.15252/emmm.201810233 (PMC6949487; doi:10.15252/emmm.201810233)

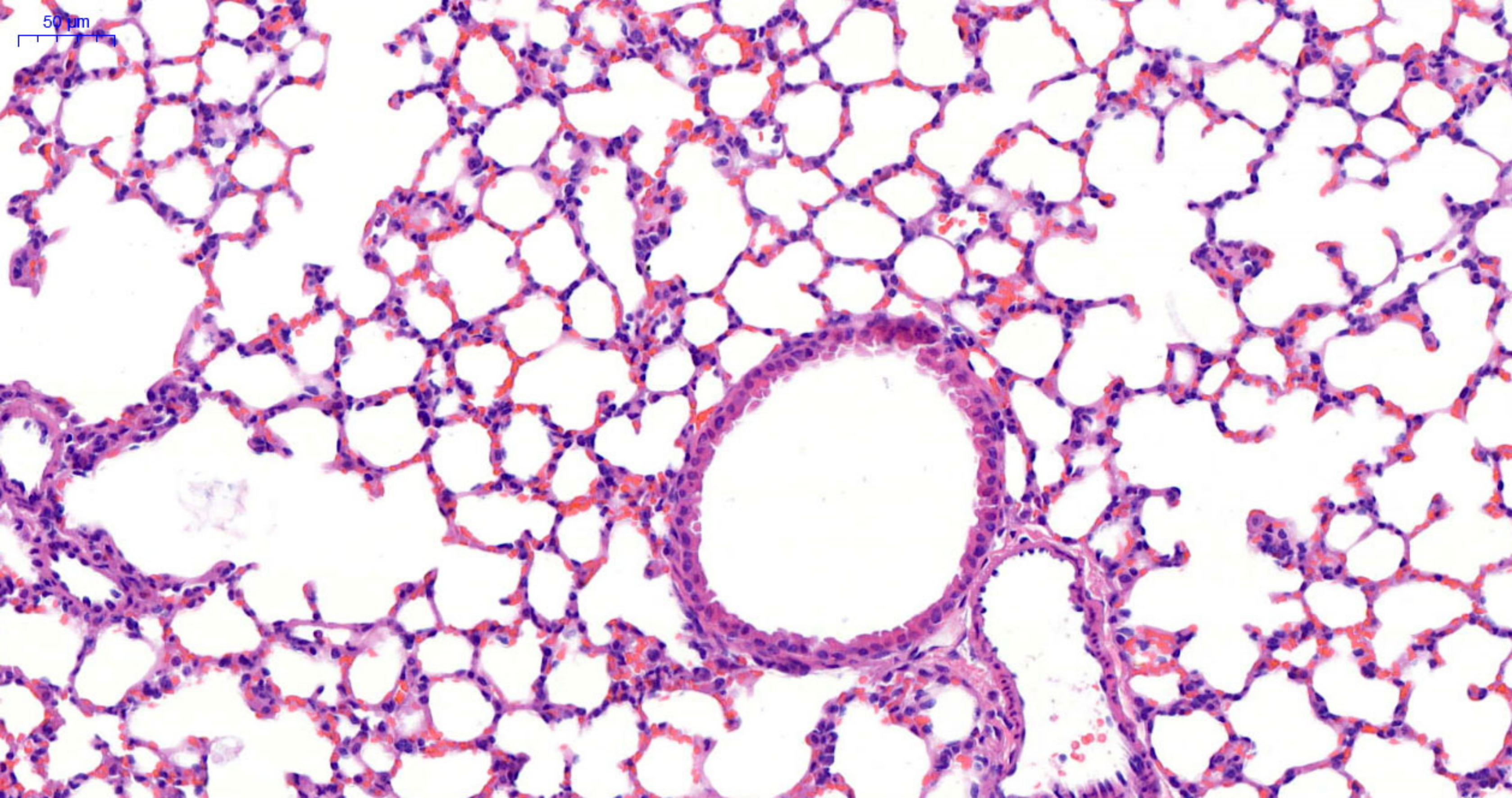

1000  $\mu\text{m}$

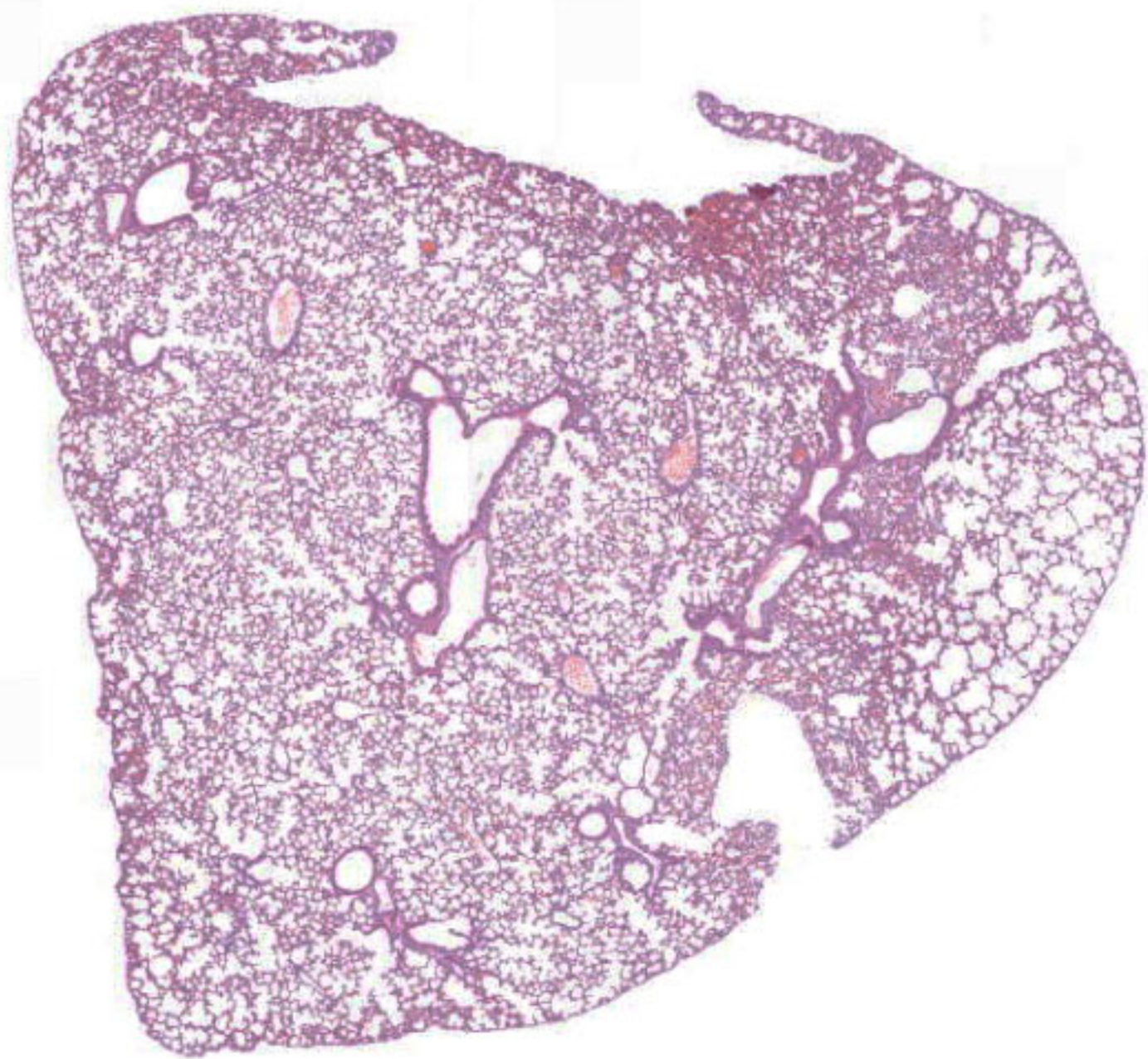

Supplement: Supplementary file 5 — Source Data for Figure 1 [file EMMM-12-e10233-s004.zip › Figure_1E_control.pdf]

1000  $\mu\text{m}$

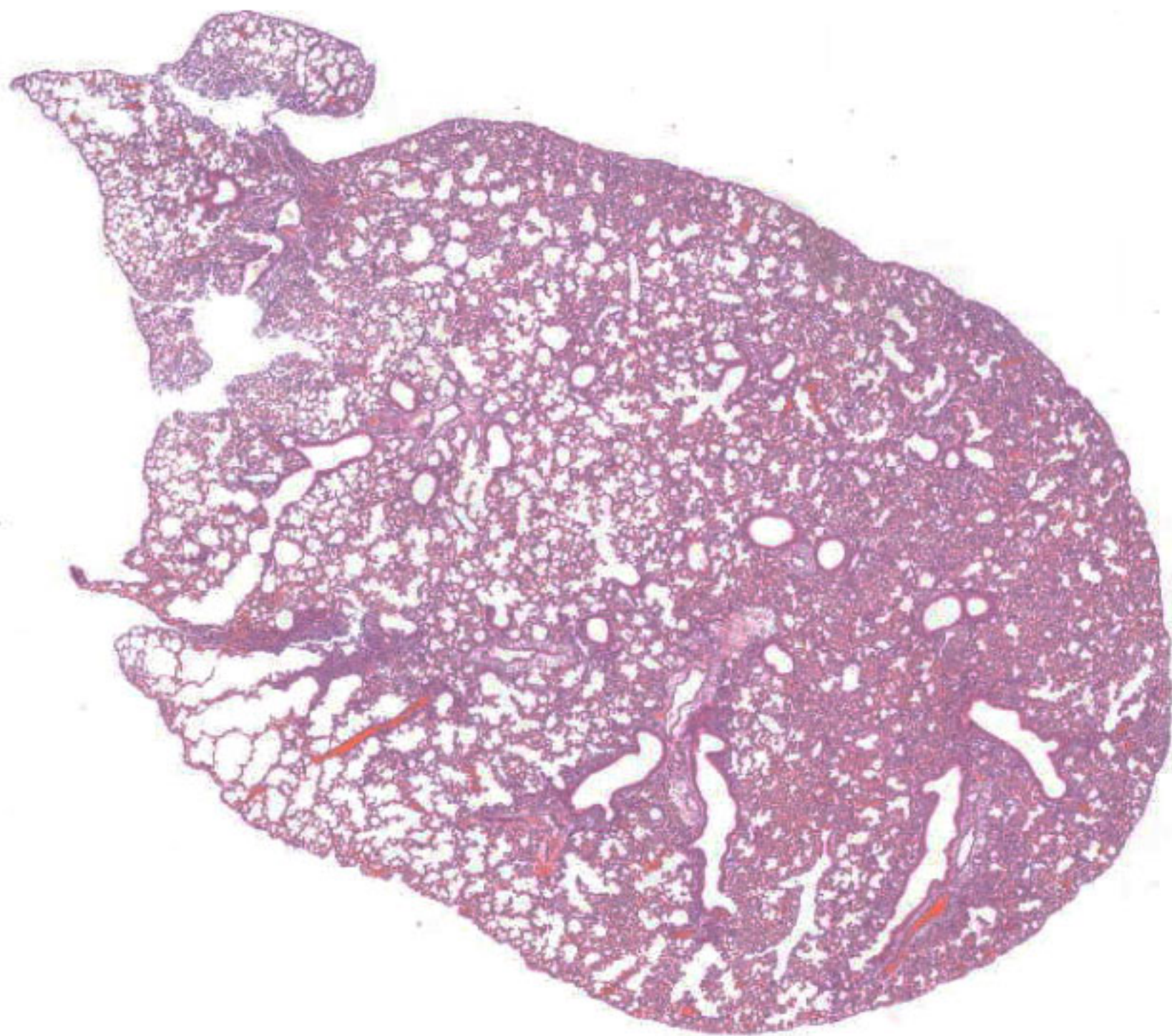

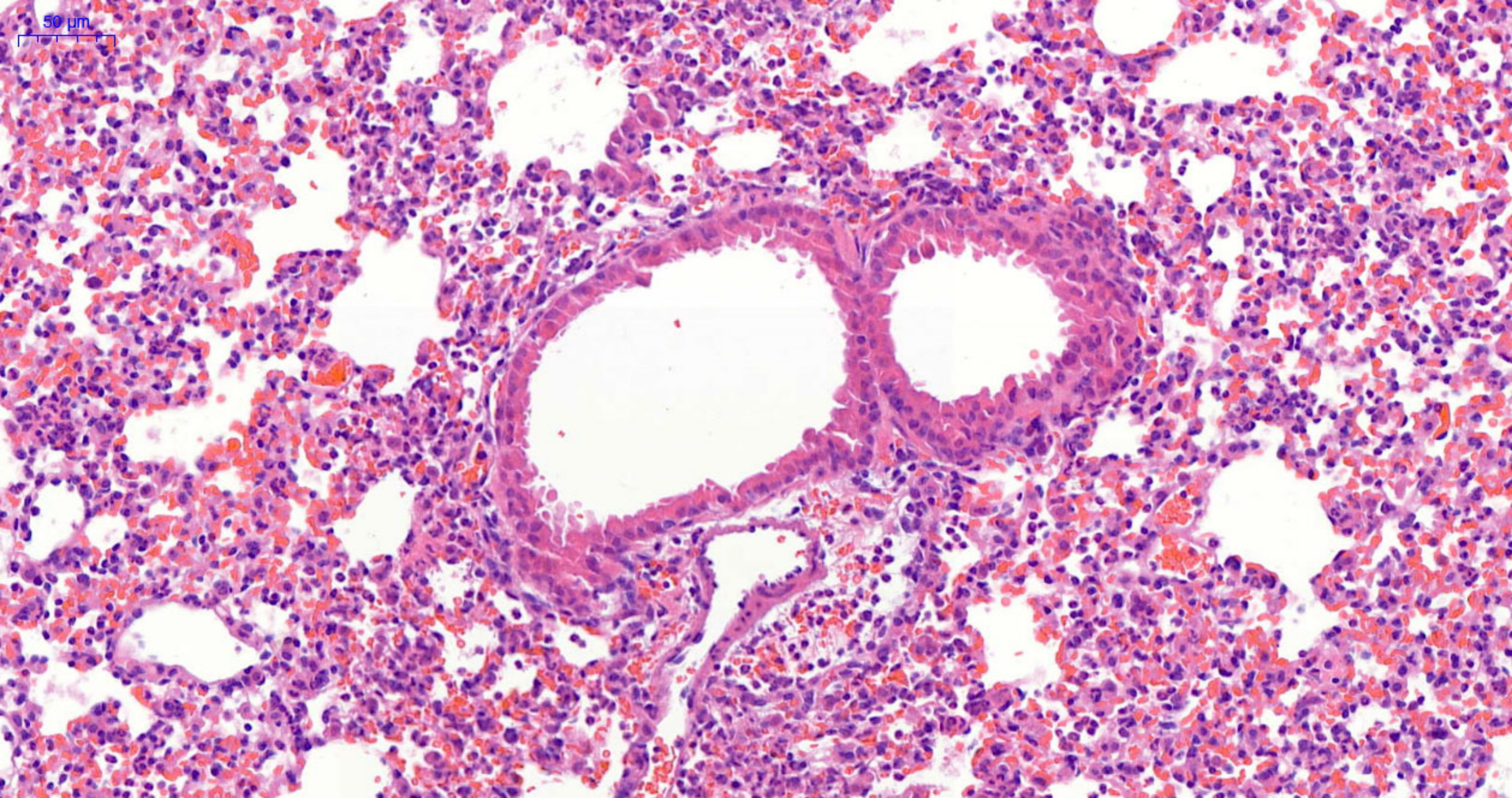

50  $\mu$ m

Supplement: Supplementary file 5 — Source Data for Figure 1 [file EMMM-12-e10233-s004.zip › Figure_1E_fvb.pdf]

1000  $\mu\text{m}$

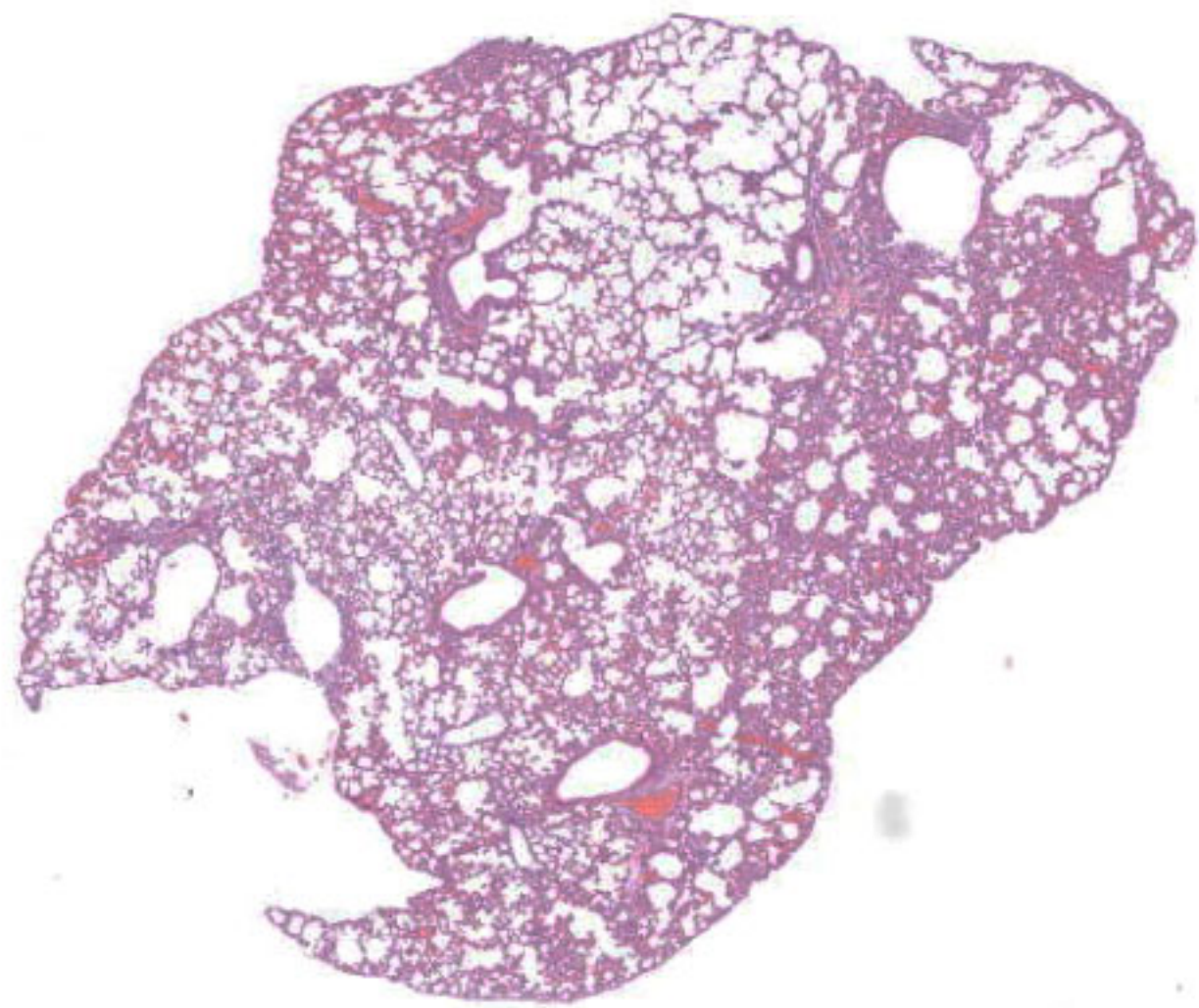

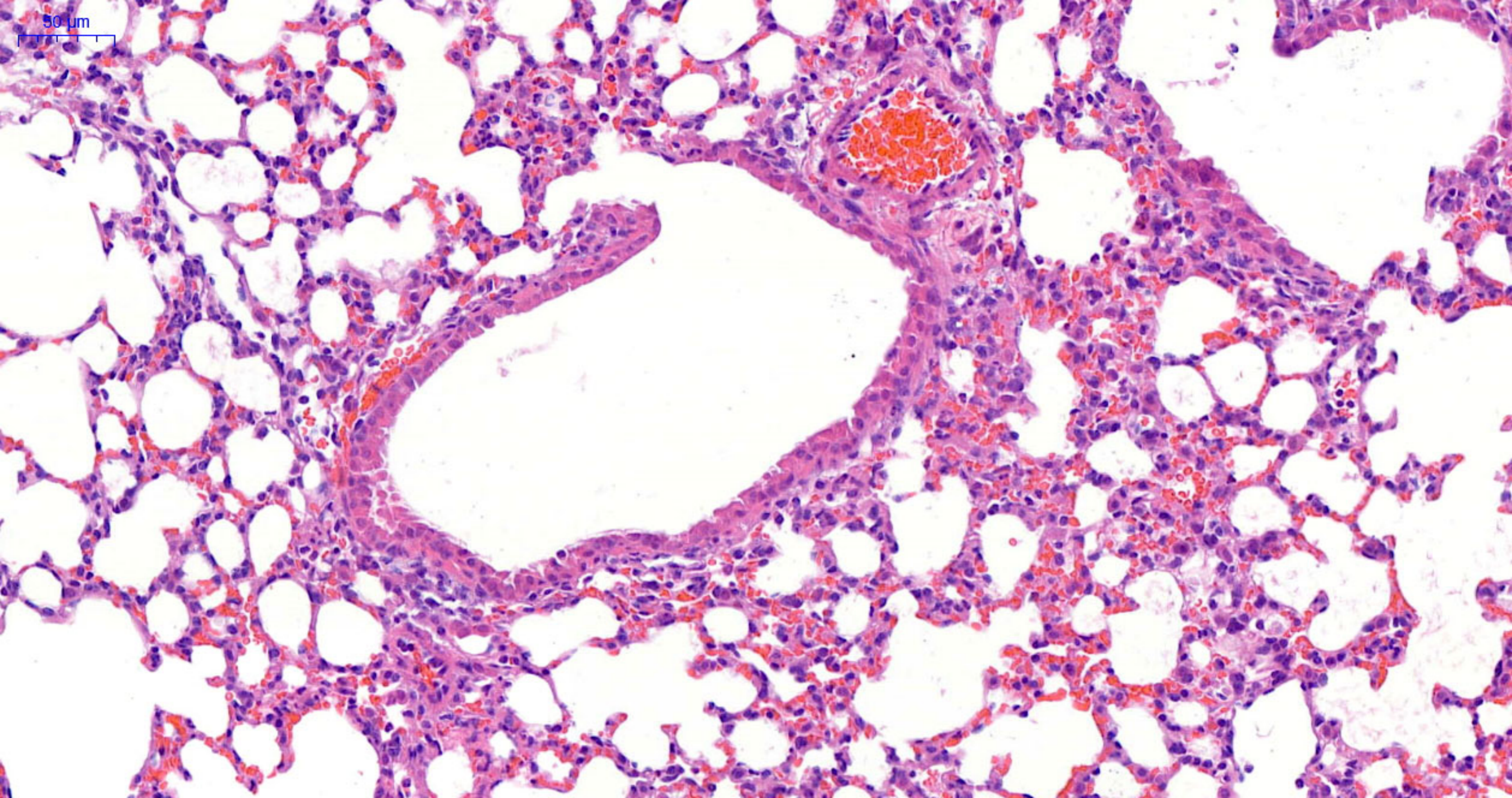

50  $\mu$ m

Supplement: Supplementary file 5 — Source Data for Figure 1 [file EMMM-12-e10233-s004.zip › Figure_1E_ll-37.pdf]

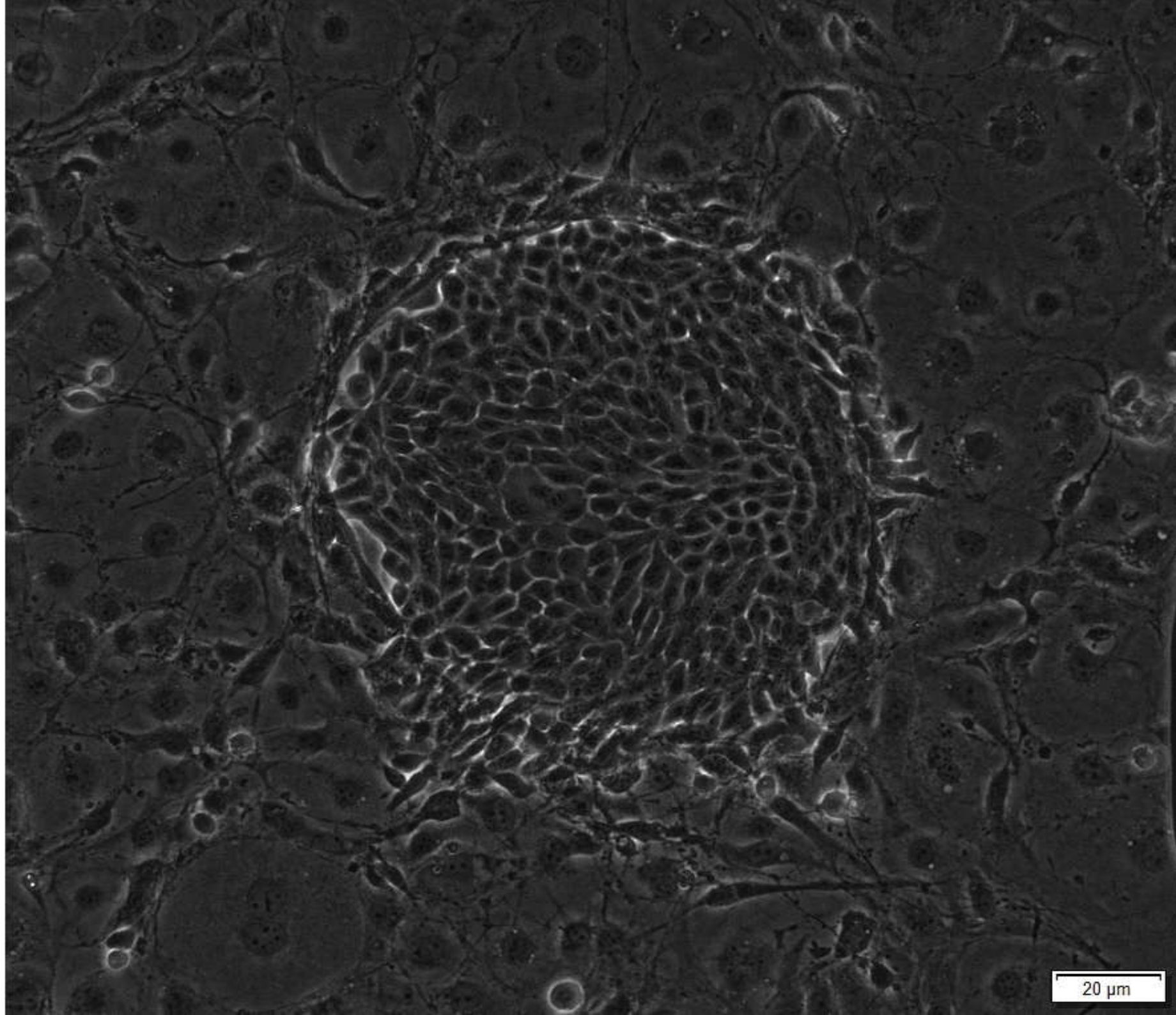

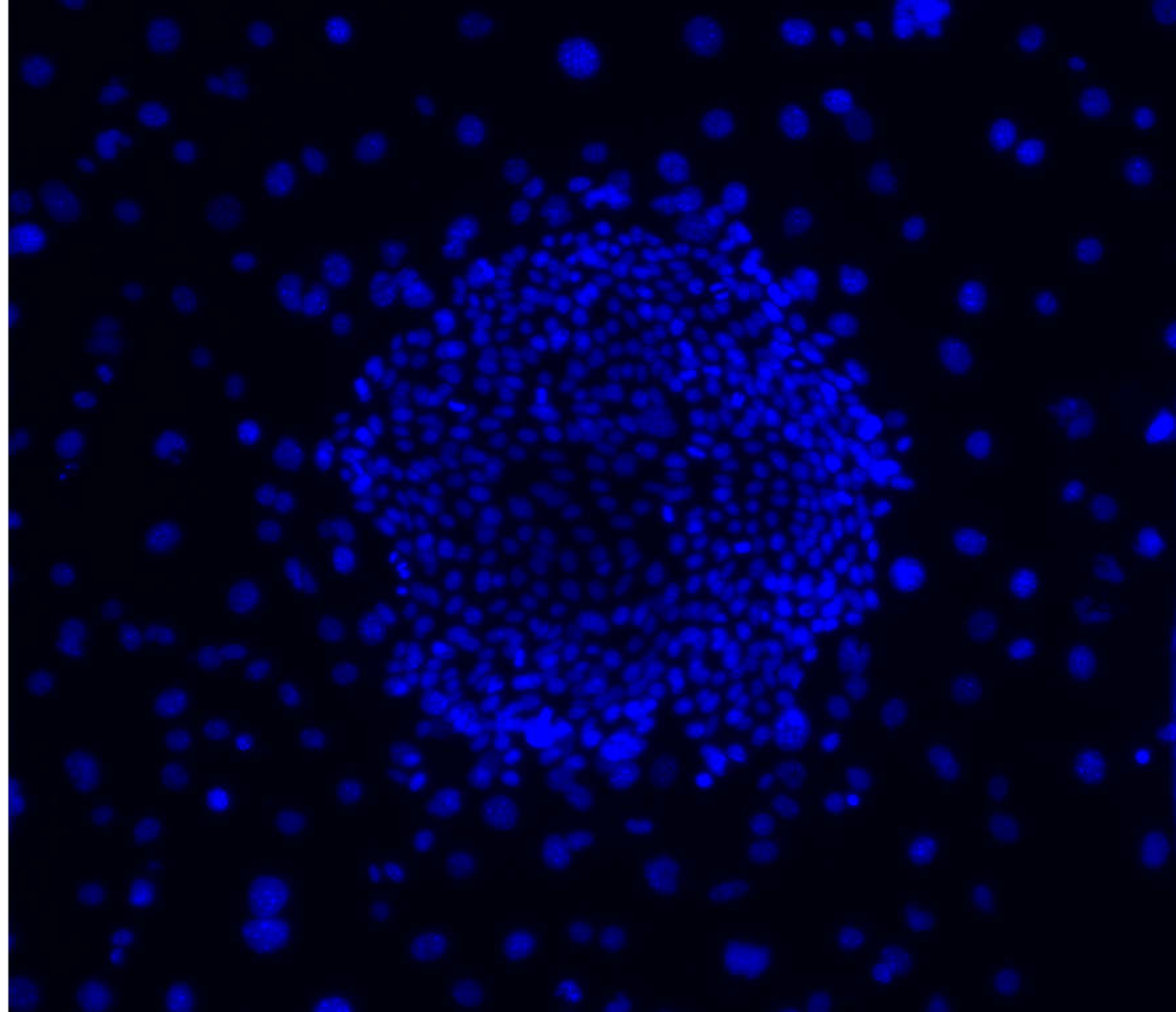

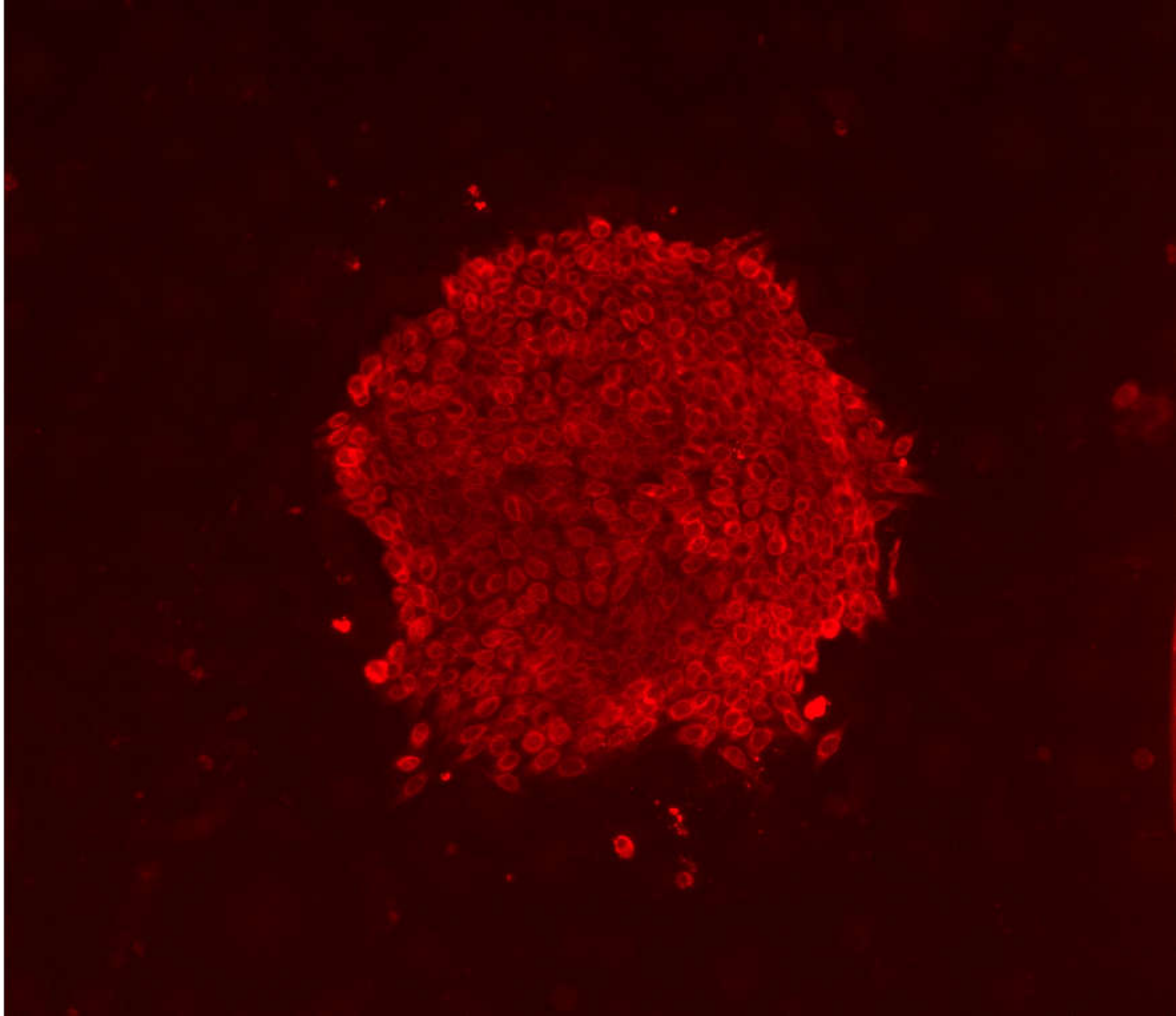

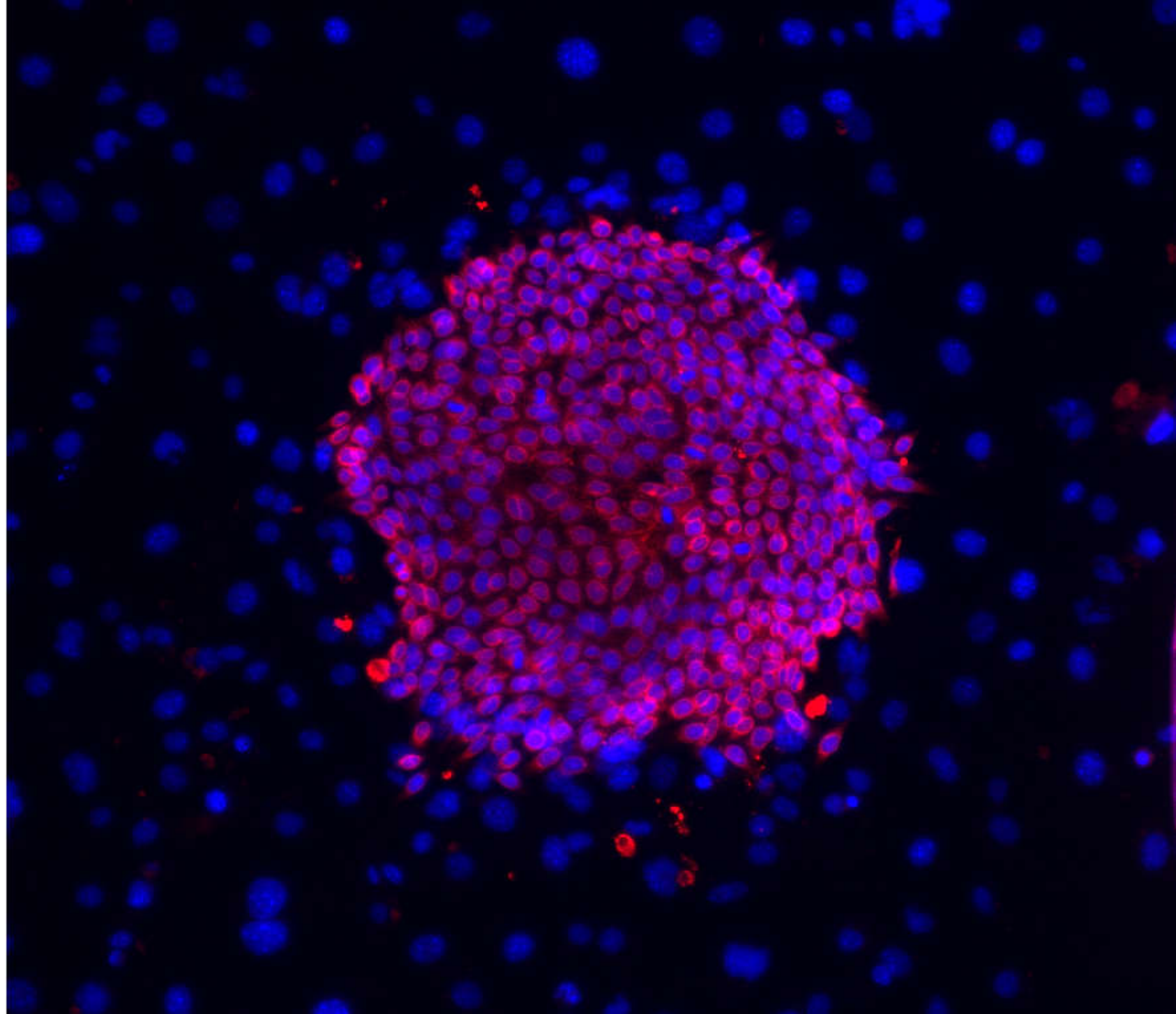

Supplement: Supplementary file 7 — Source Data for Figure 3 [file EMMM-12-e10233-s006.zip › Figure_3A_LL-37_mDASC.pdf]

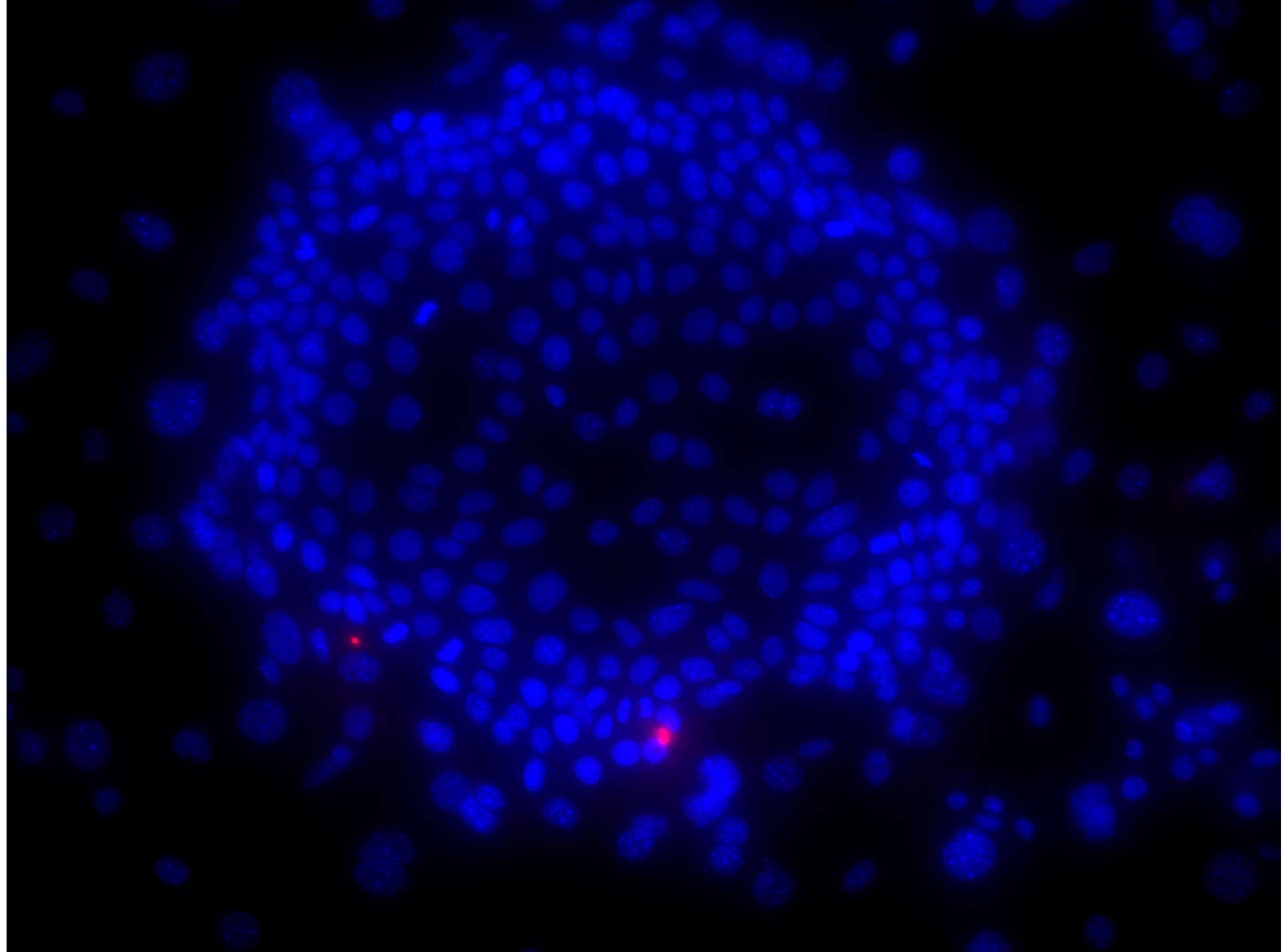

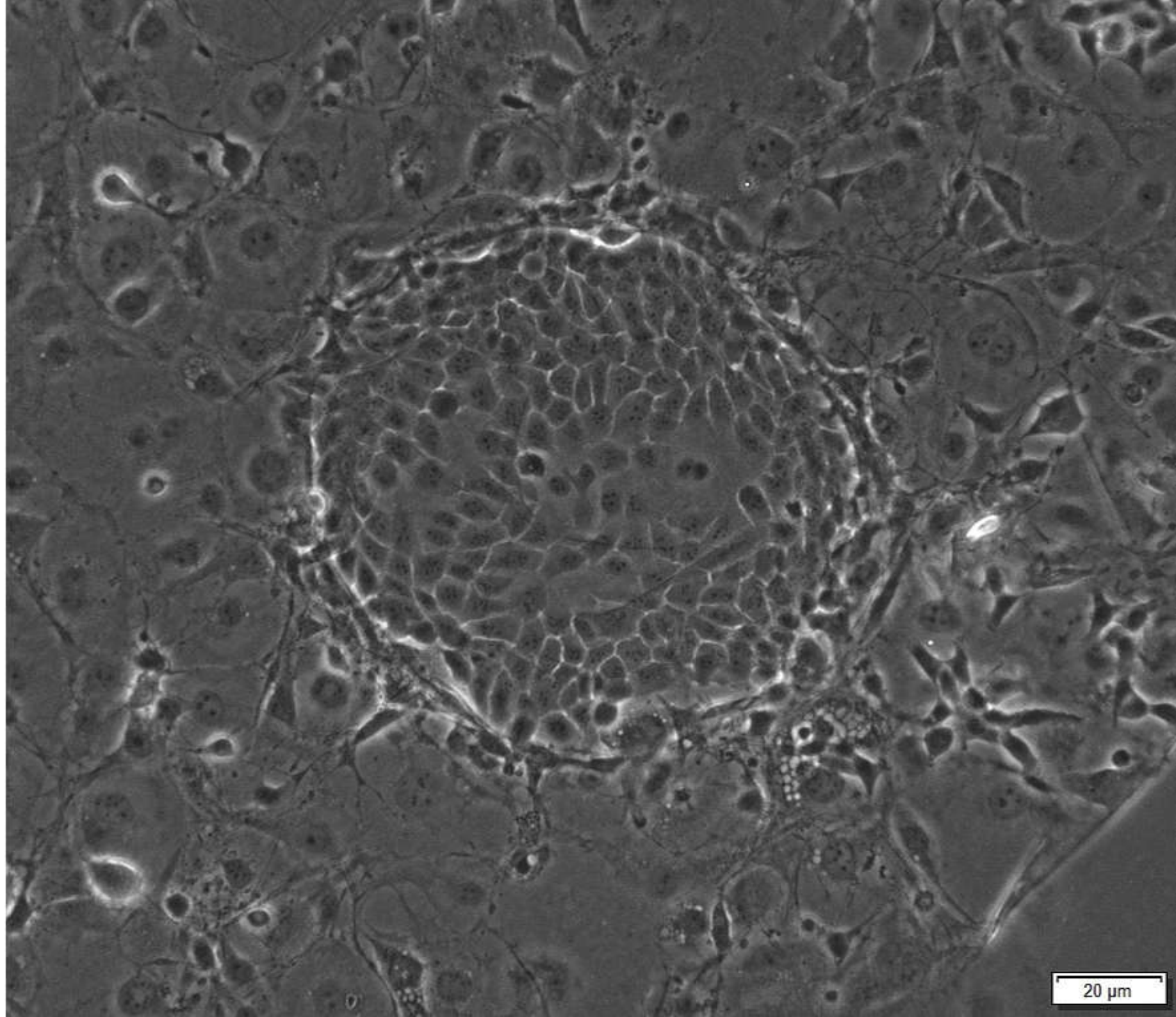

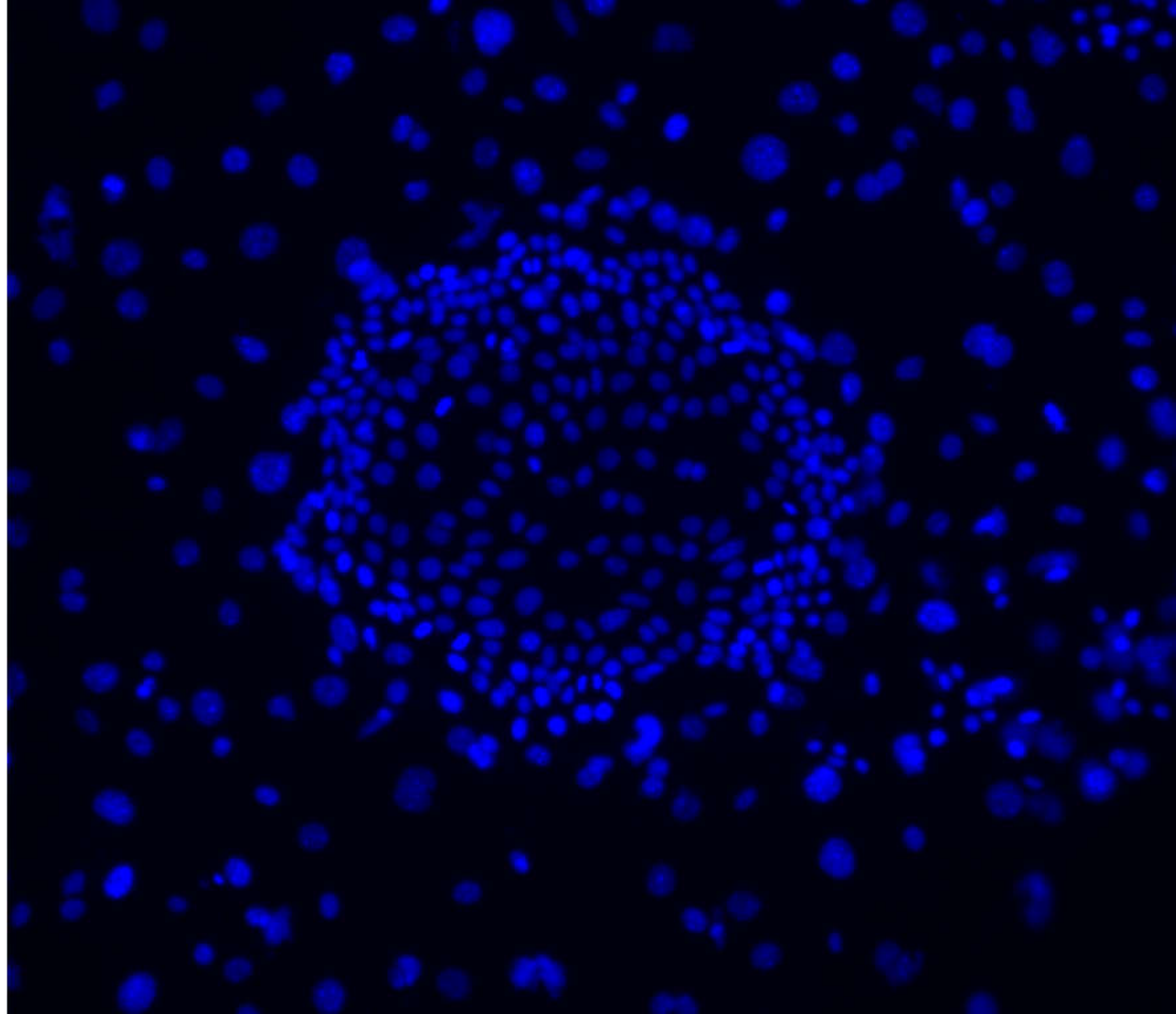

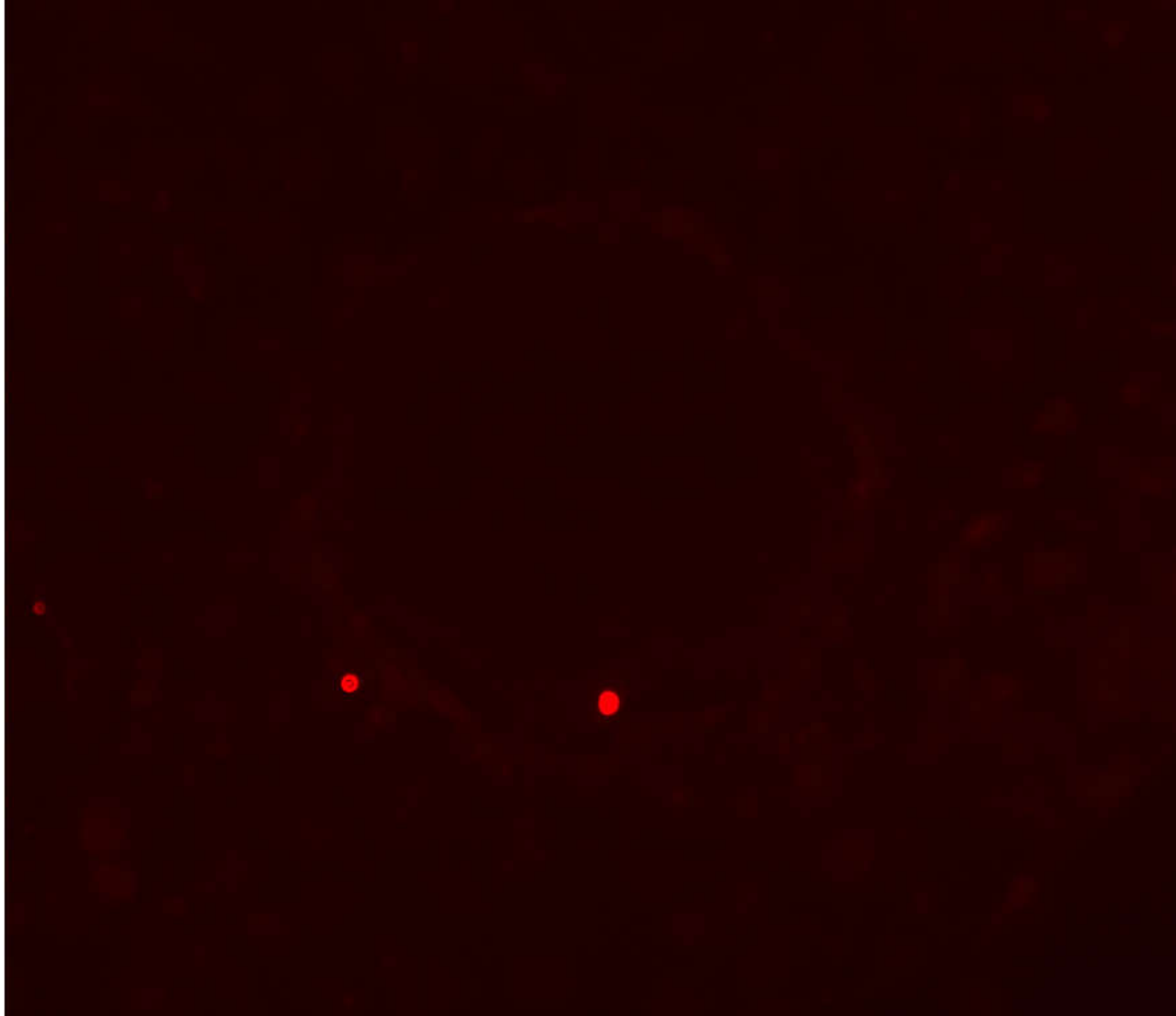

Supplement: Supplementary file 7 — Source Data for Figure 3 [file EMMM-12-e10233-s006.zip › Figure_3A_WT_mDASC.pdf]

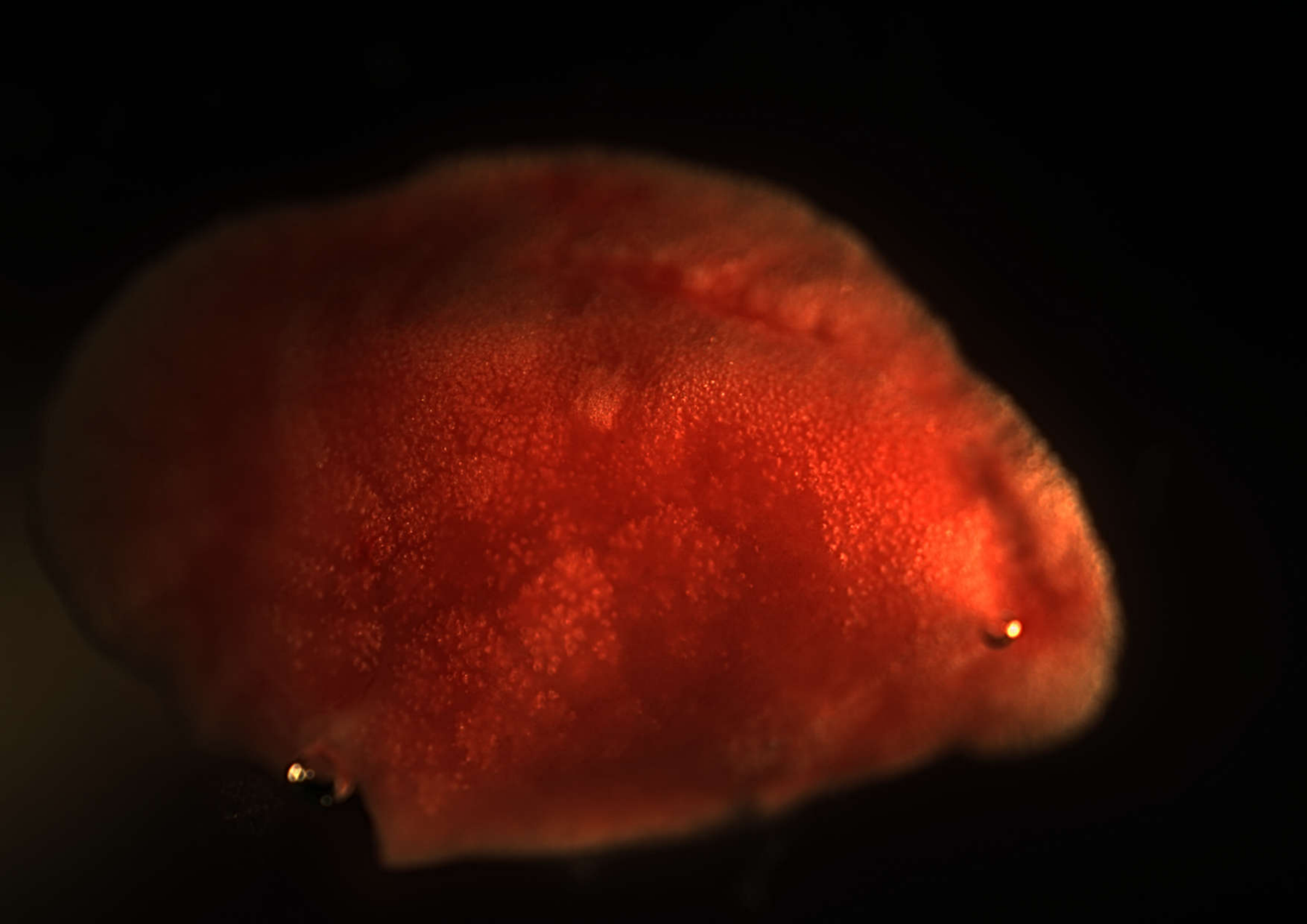

Supplement: Supplementary file 8 — Source Data for Figure 4 [file EMMM-12-e10233-s007.zip › Figure_4A_0_dpdf.pdf]

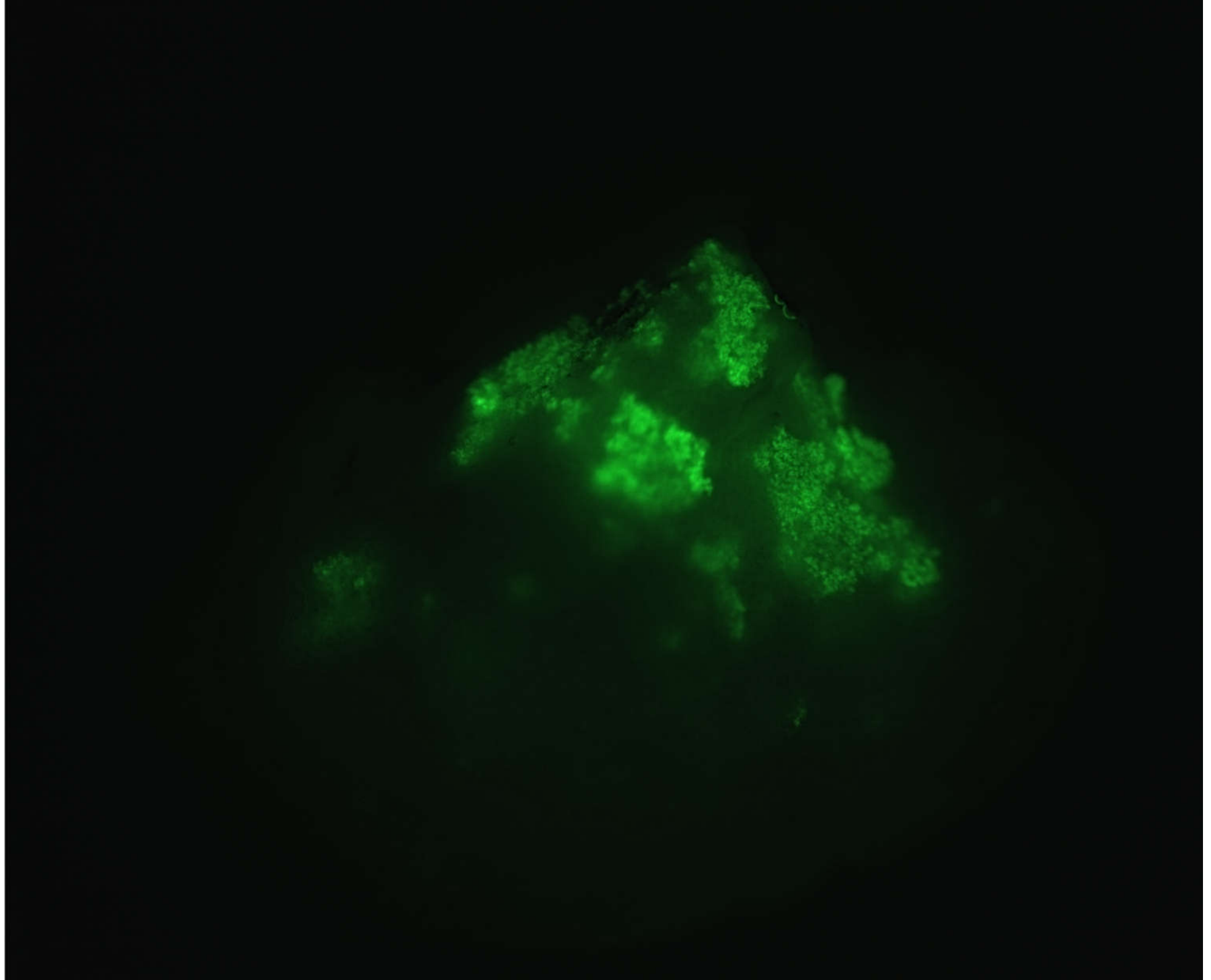

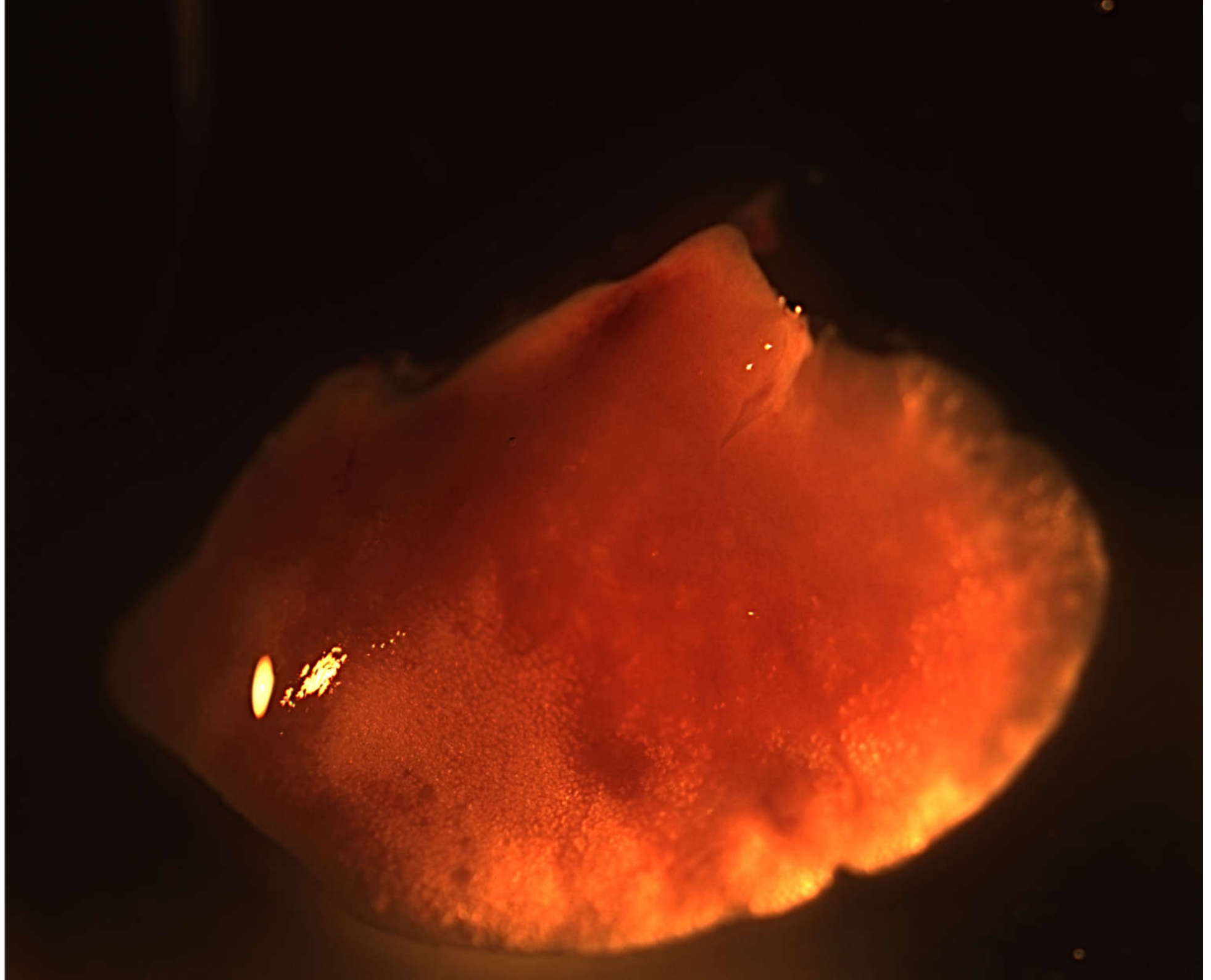

Supplement: Supplementary file 8 — Source Data for Figure 4 [file EMMM-12-e10233-s007.zip › Figure_4A_LL-37-lung_14days.pdf]

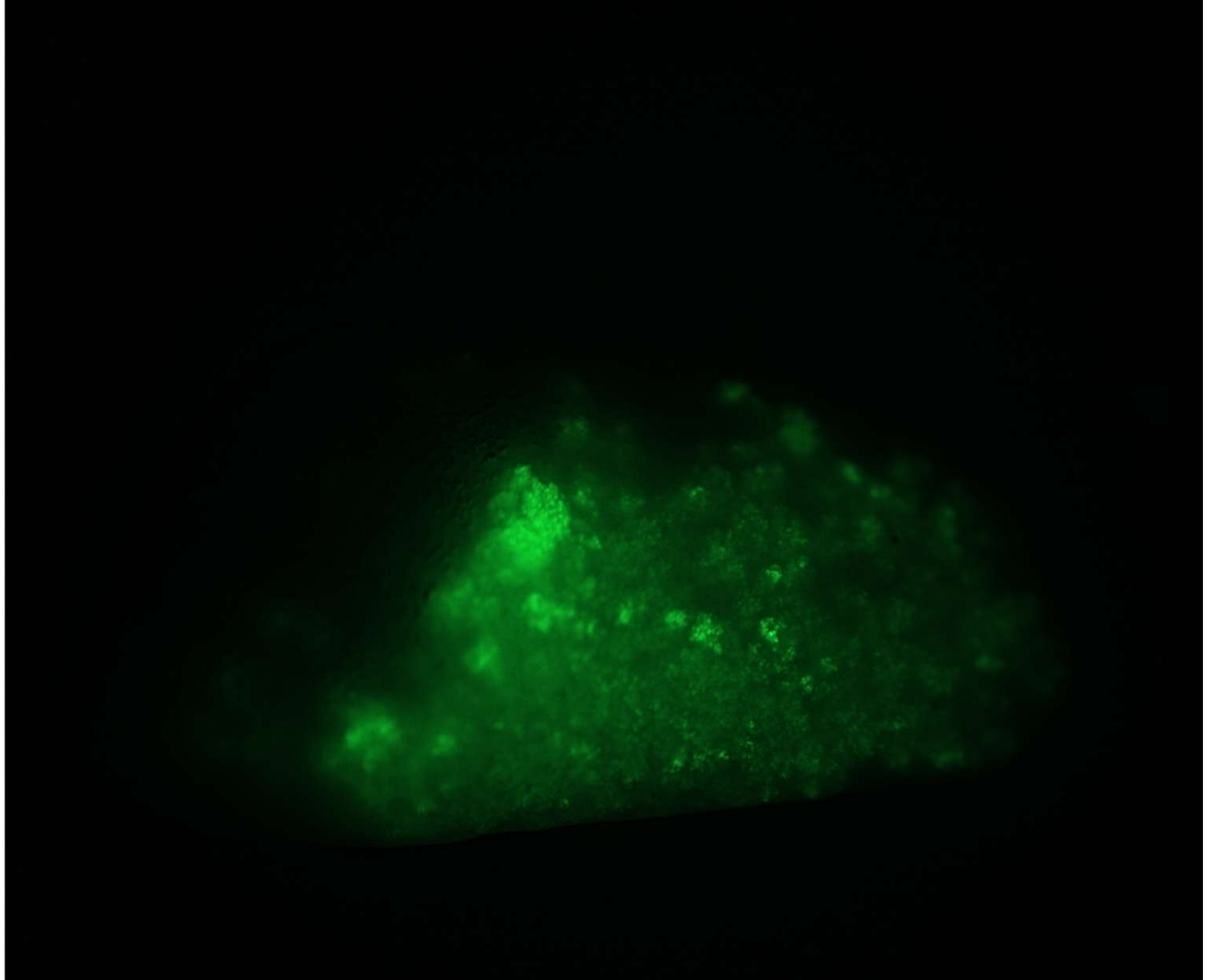

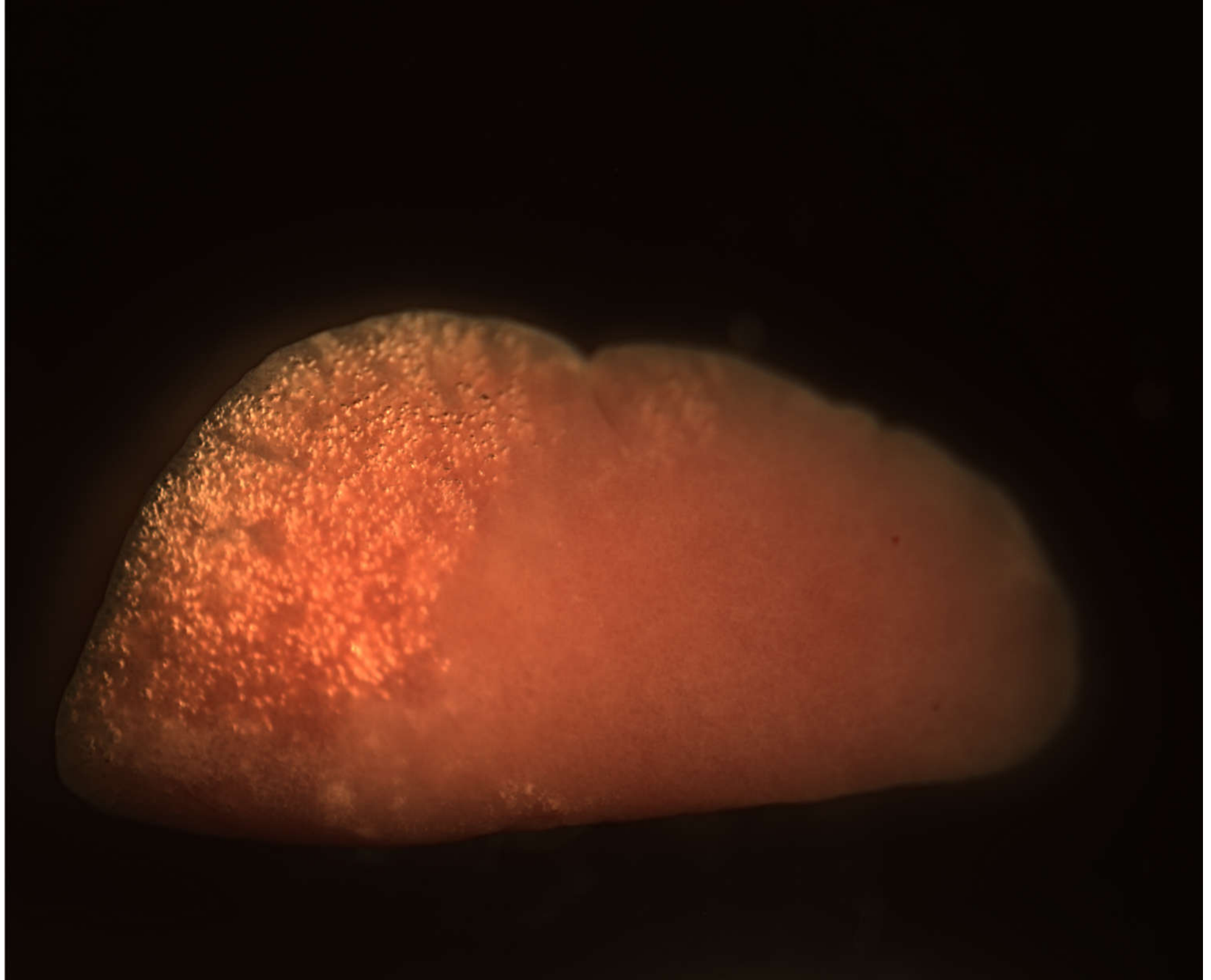

Supplement: Supplementary file 8 — Source Data for Figure 4 [file EMMM-12-e10233-s007.zip › Figure_4A_LL-37-lung_21days.pdf]

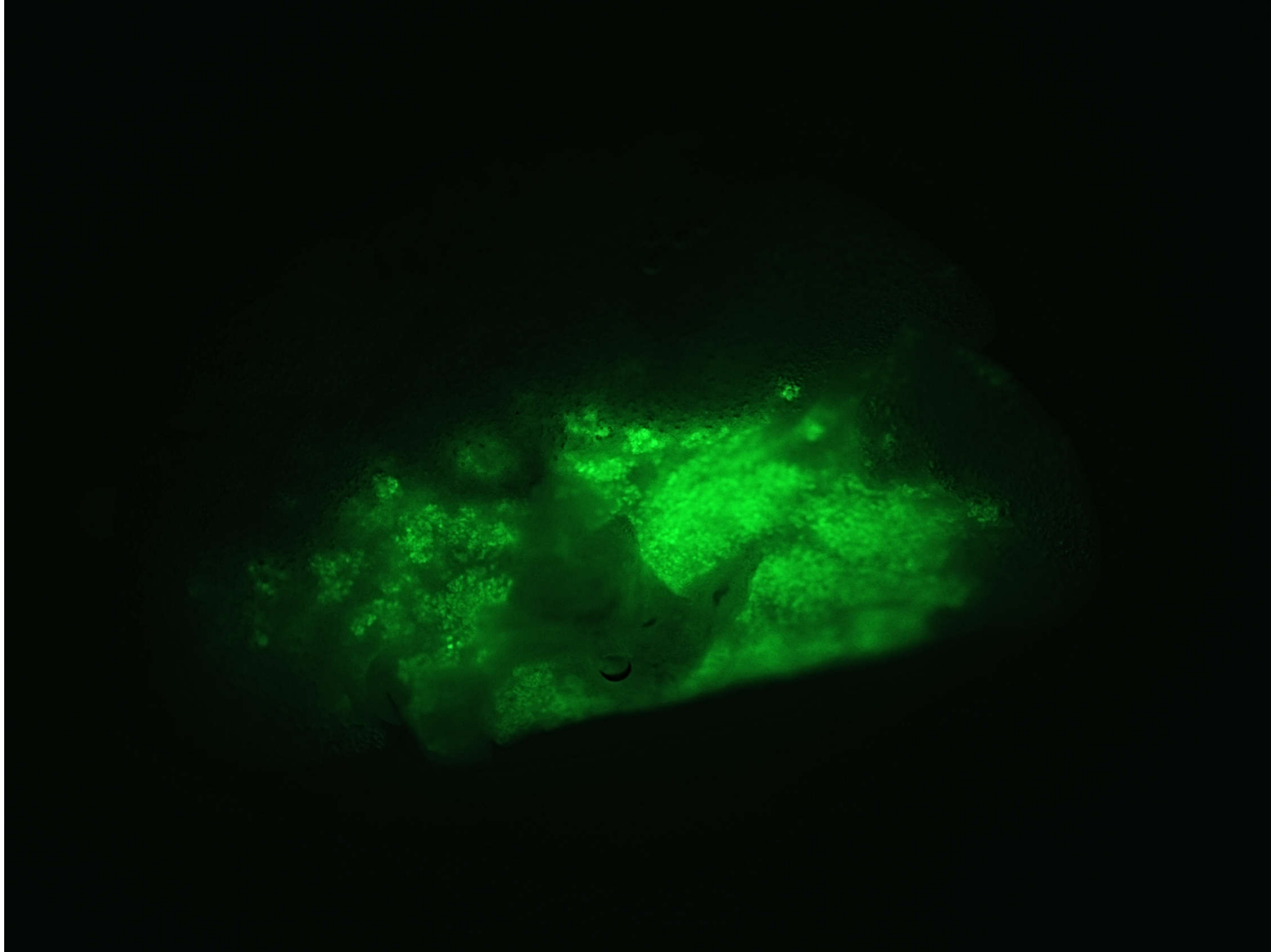

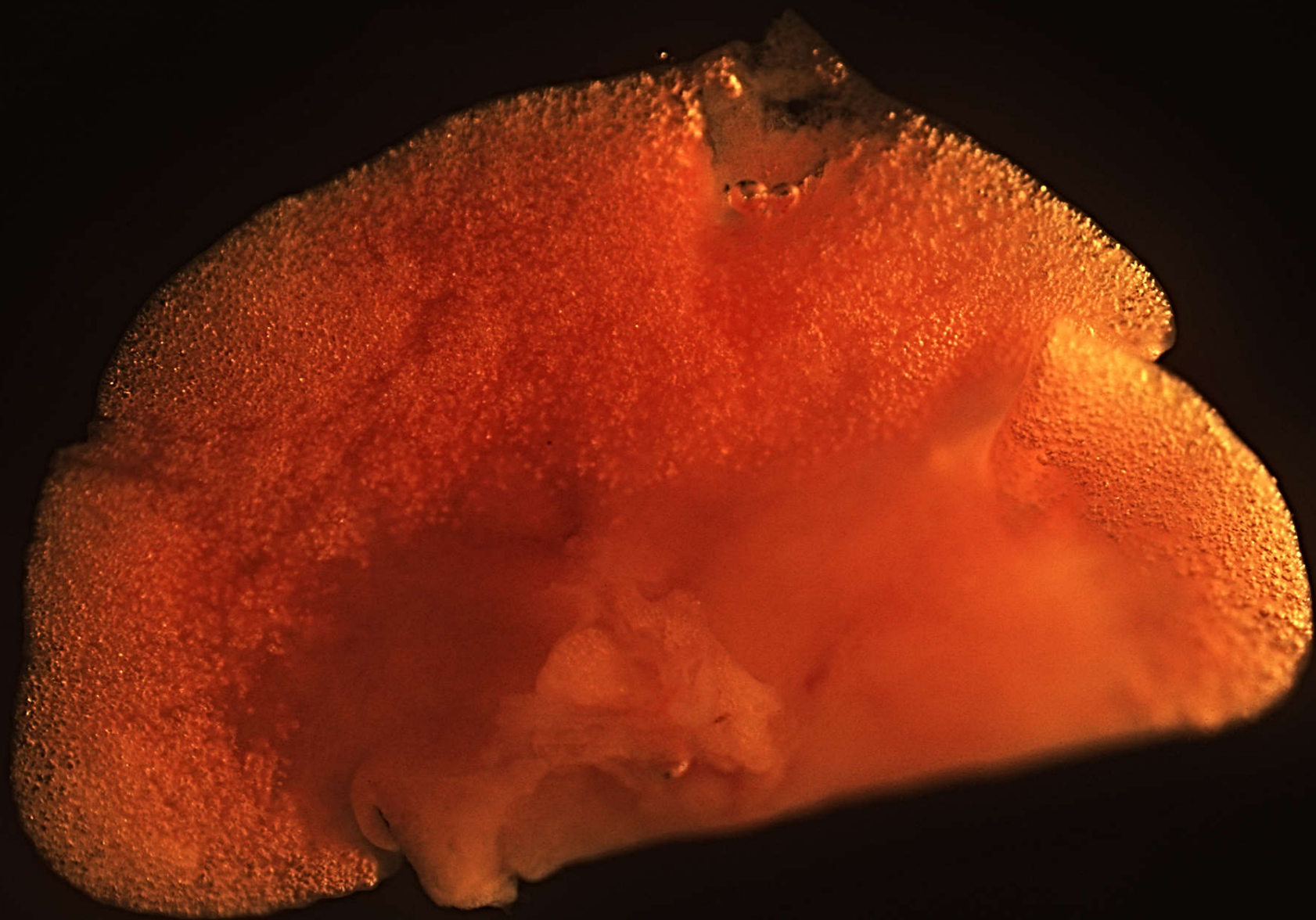

Supplement: Supplementary file 8 — Source Data for Figure 4 [file EMMM-12-e10233-s007.zip › Figure_4A_LL-37-lung_28days.pdf]

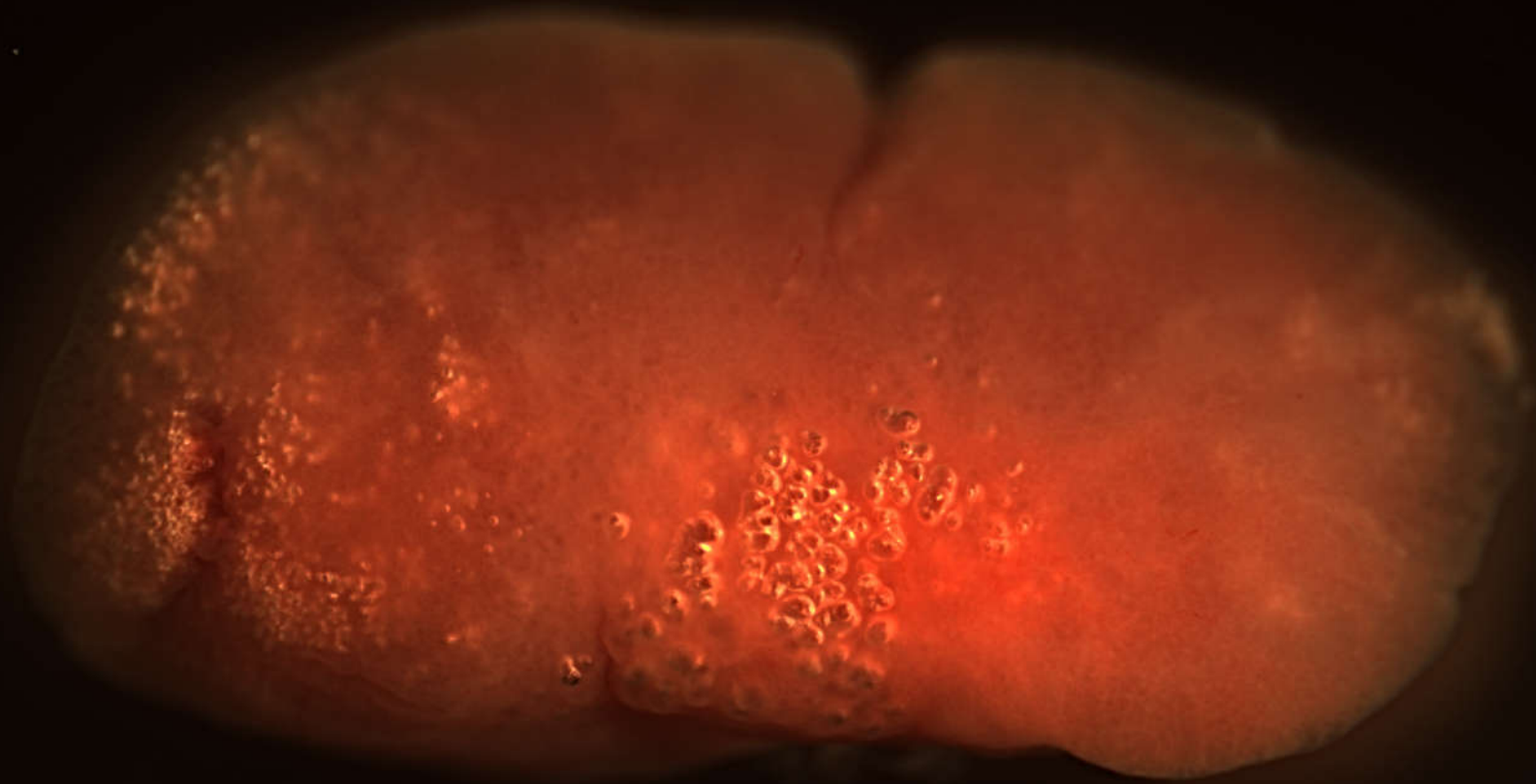

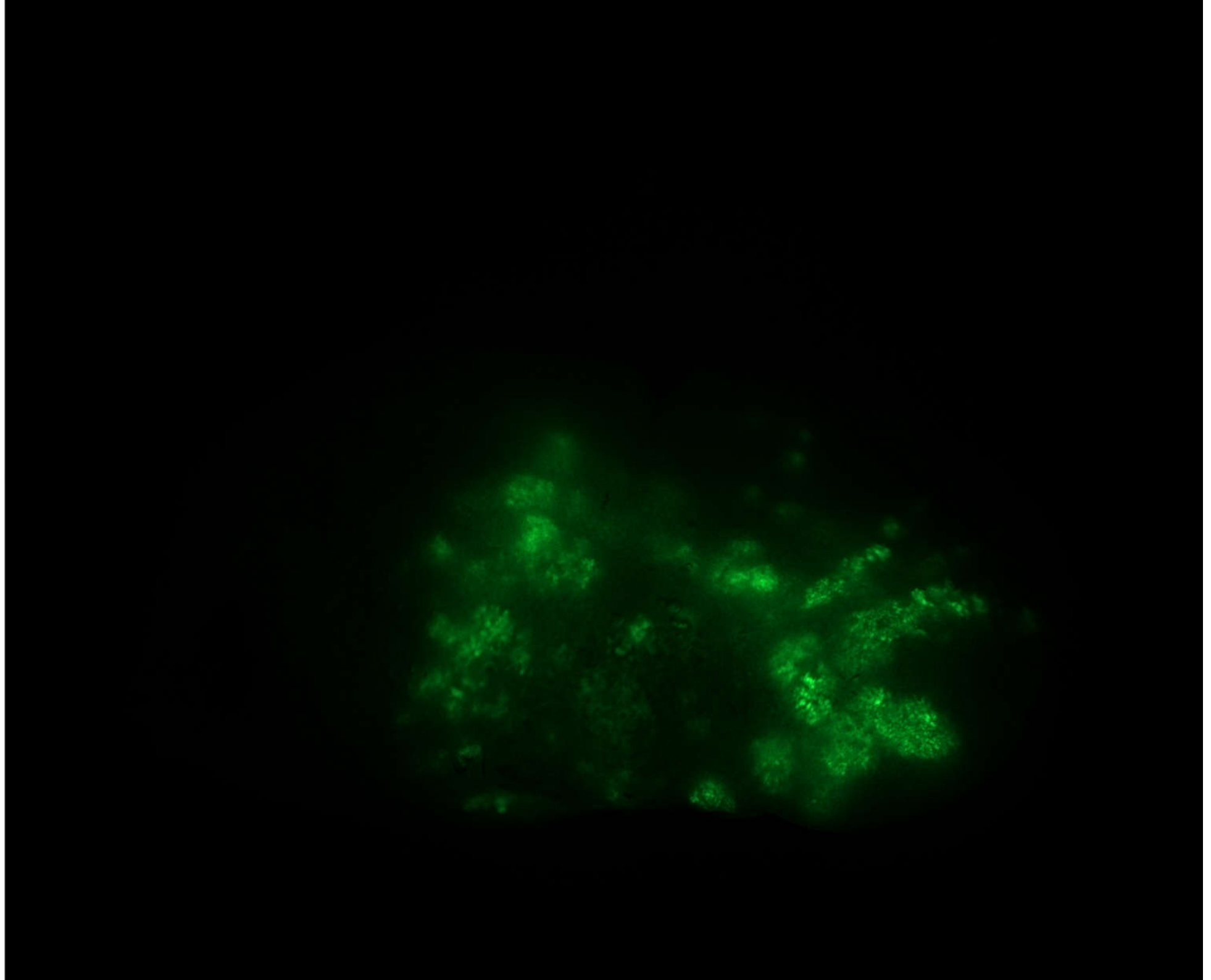

Supplement: Supplementary file 8 — Source Data for Figure 4 [file EMMM-12-e10233-s007.zip › Figure_4A_LL-37-lung_7days.pdf]

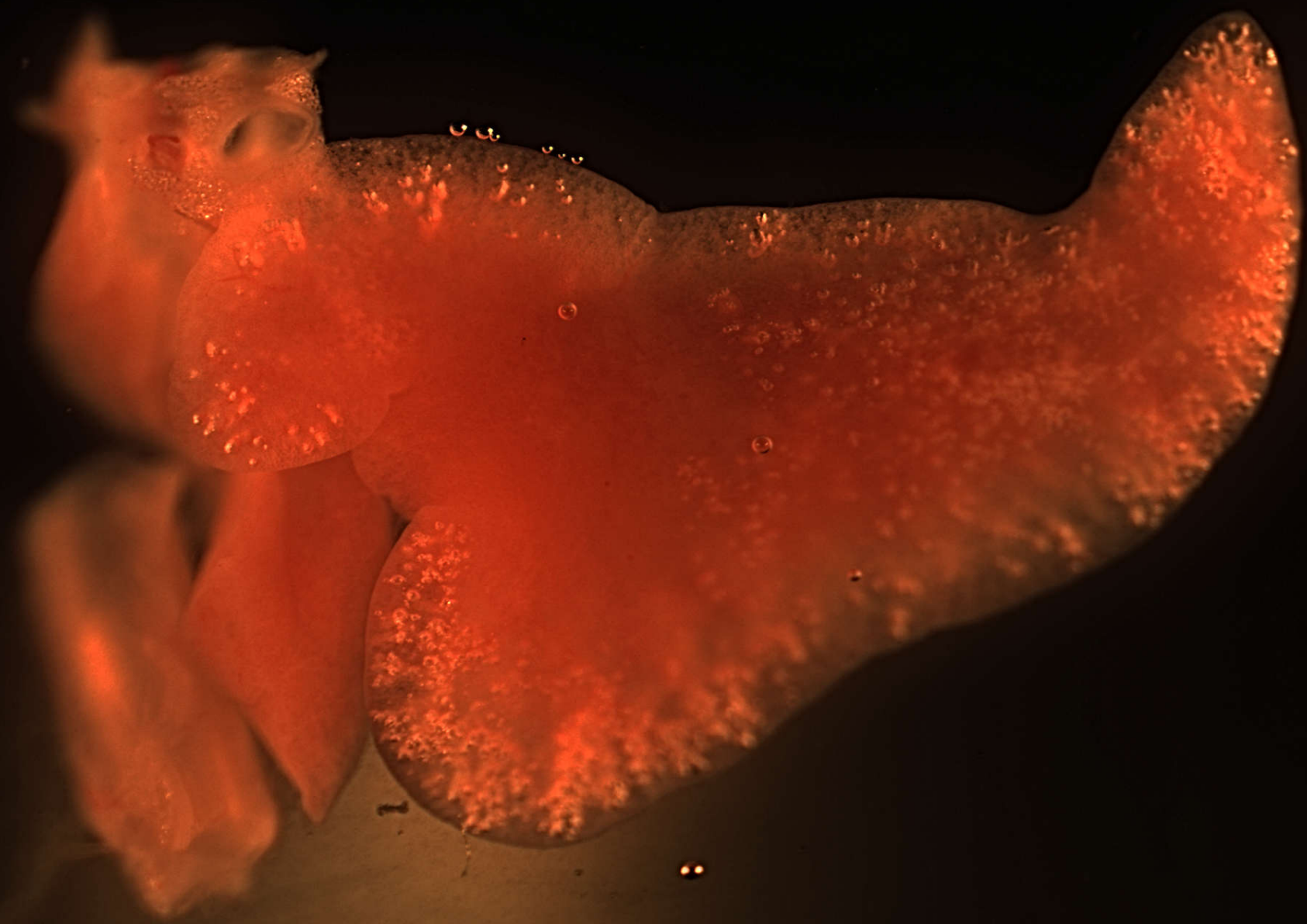

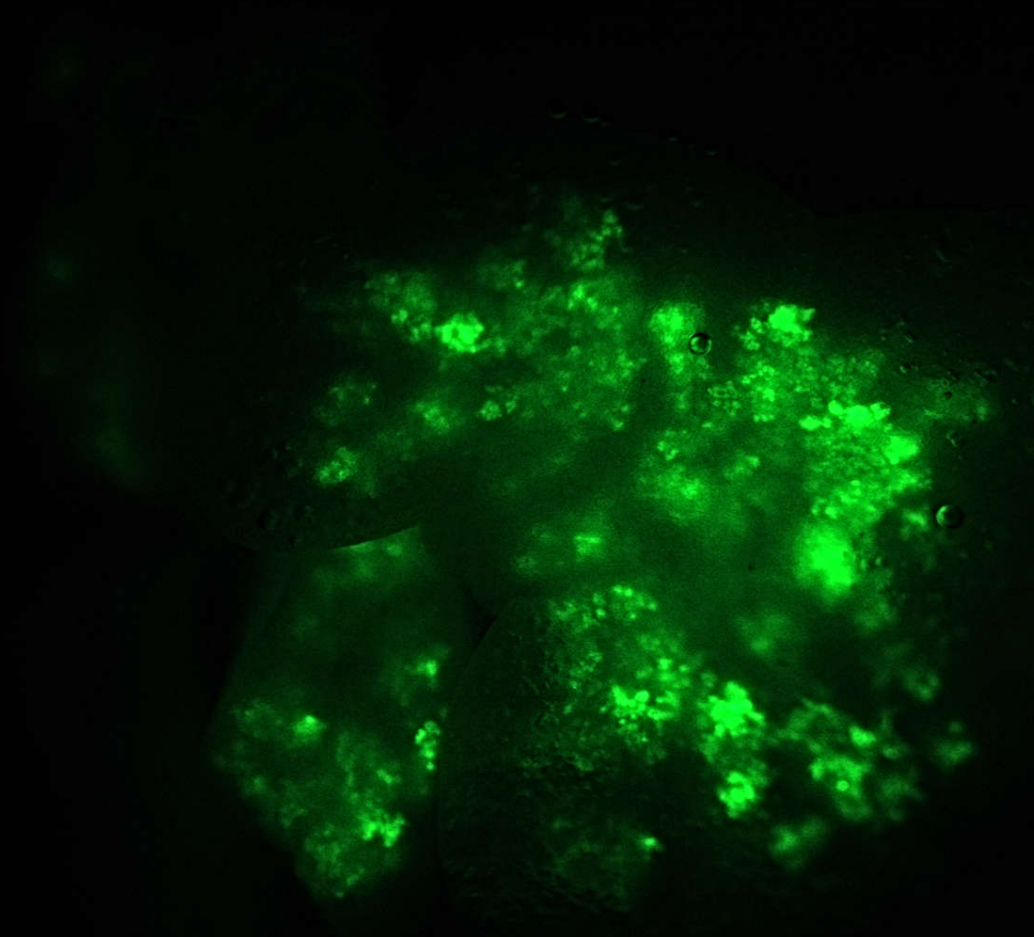

Supplement: Supplementary file 8 — Source Data for Figure 4 [file EMMM-12-e10233-s007.zip › Figure_4A_WT-lung_14days.pdf]

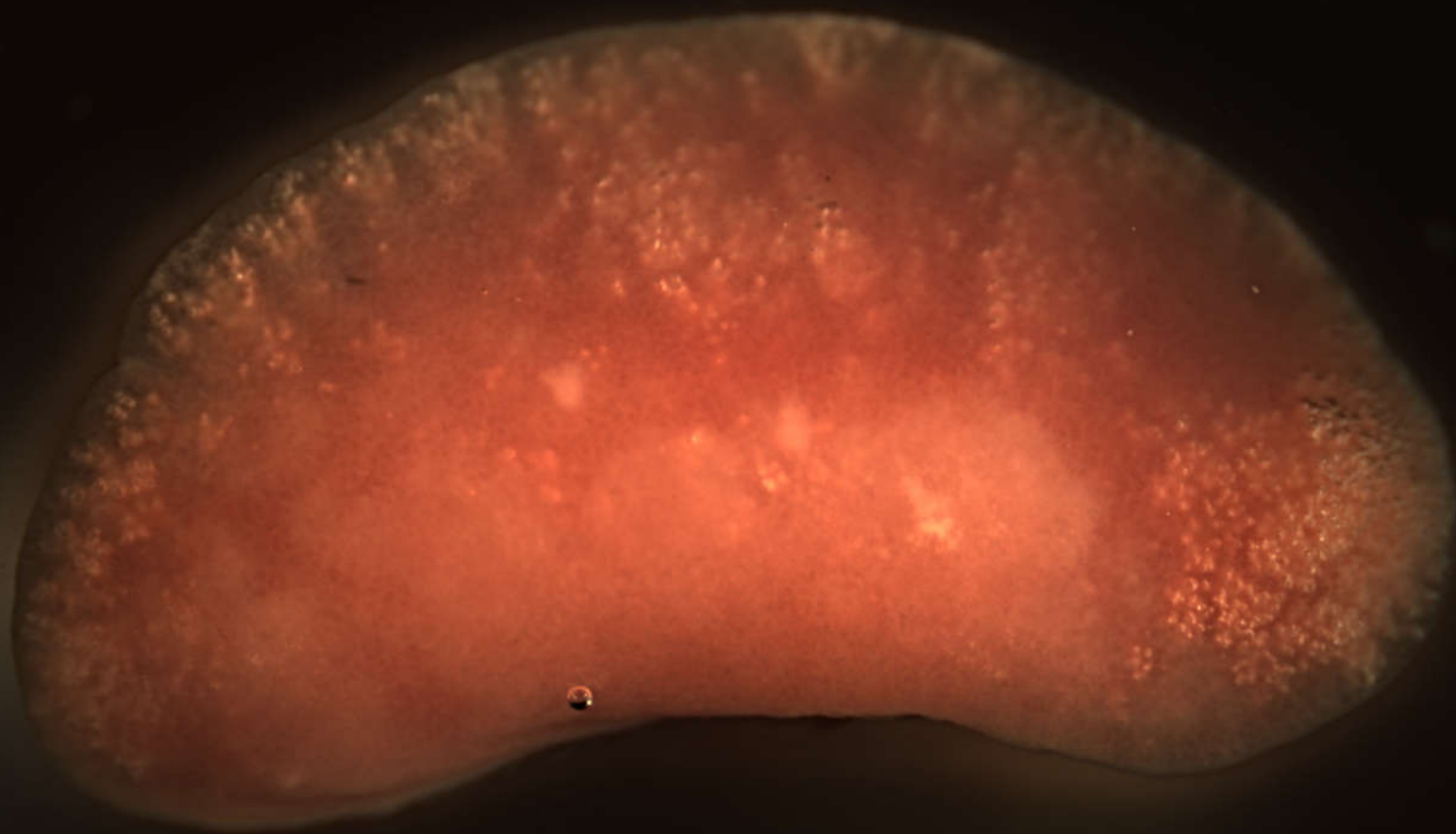

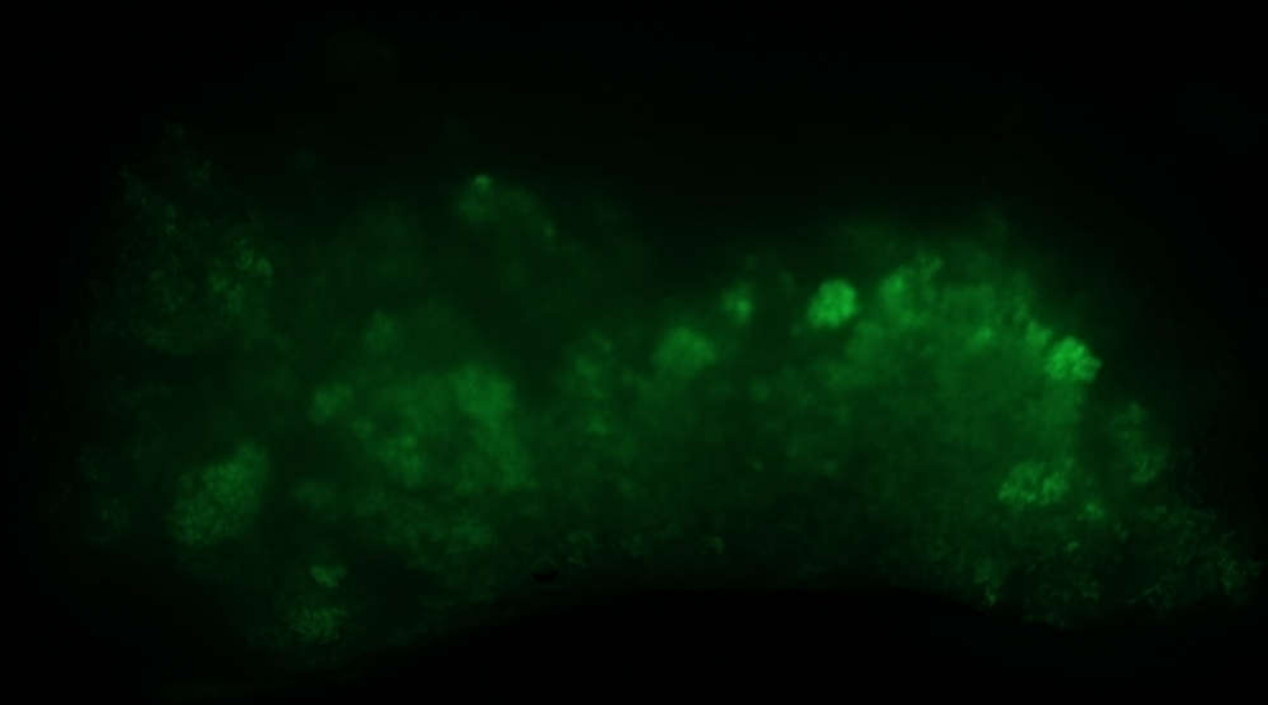

Supplement: Supplementary file 8 — Source Data for Figure 4 [file EMMM-12-e10233-s007.zip › Figure_4A_WT-lung_21days.pdf]

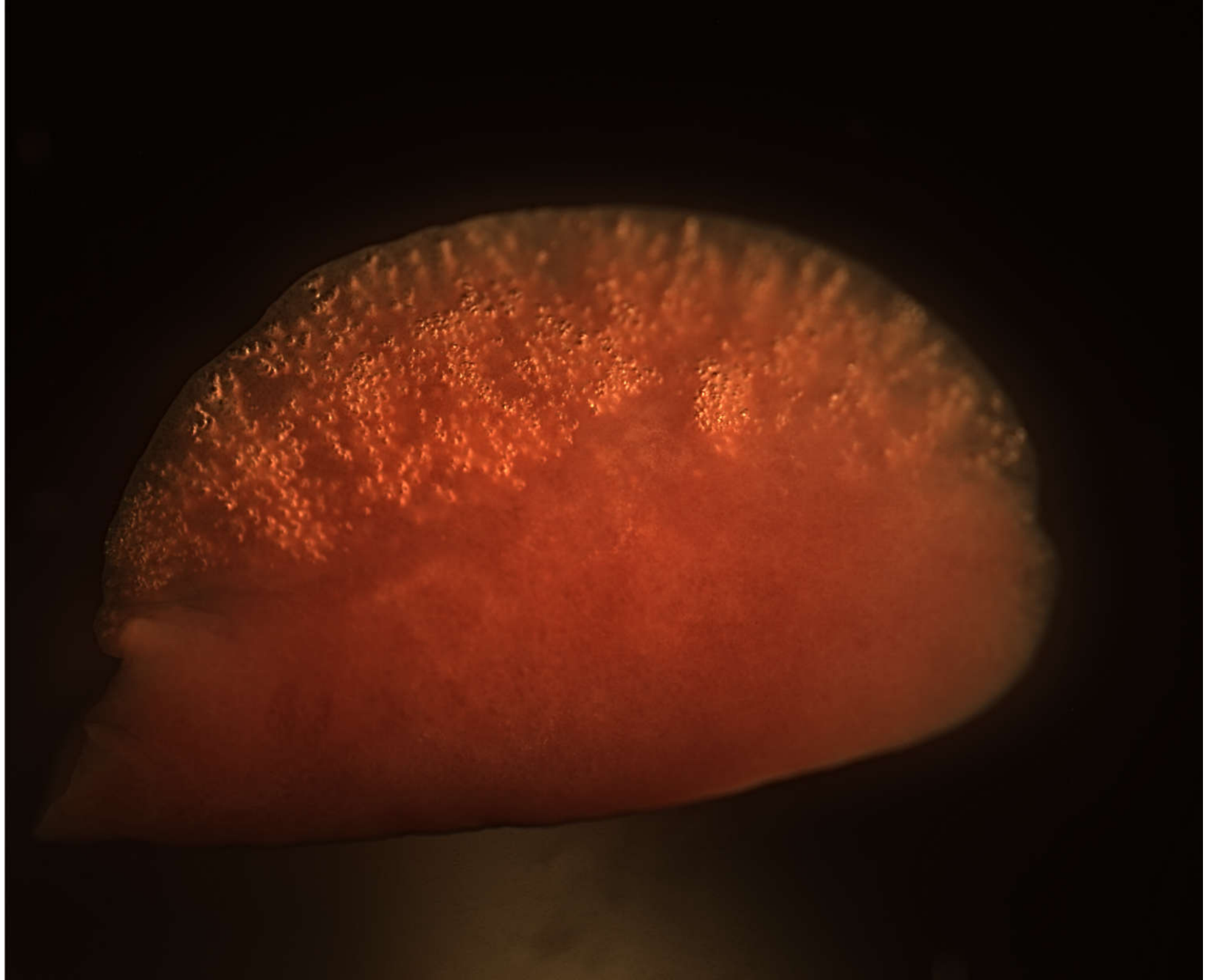

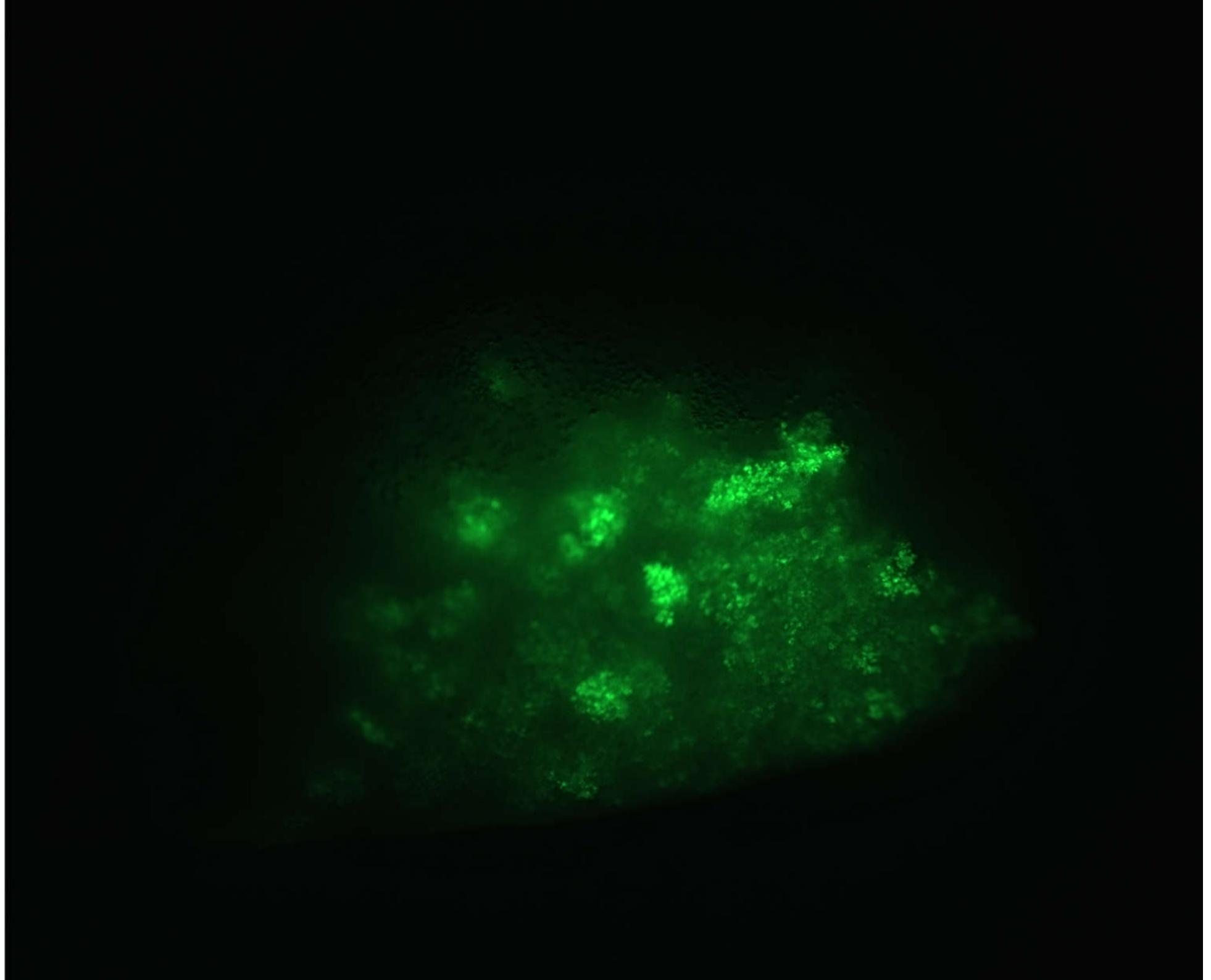

Supplement: Supplementary file 8 — Source Data for Figure 4 [file EMMM-12-e10233-s007.zip › Figure_4A_WT-lung_28days.pdf]

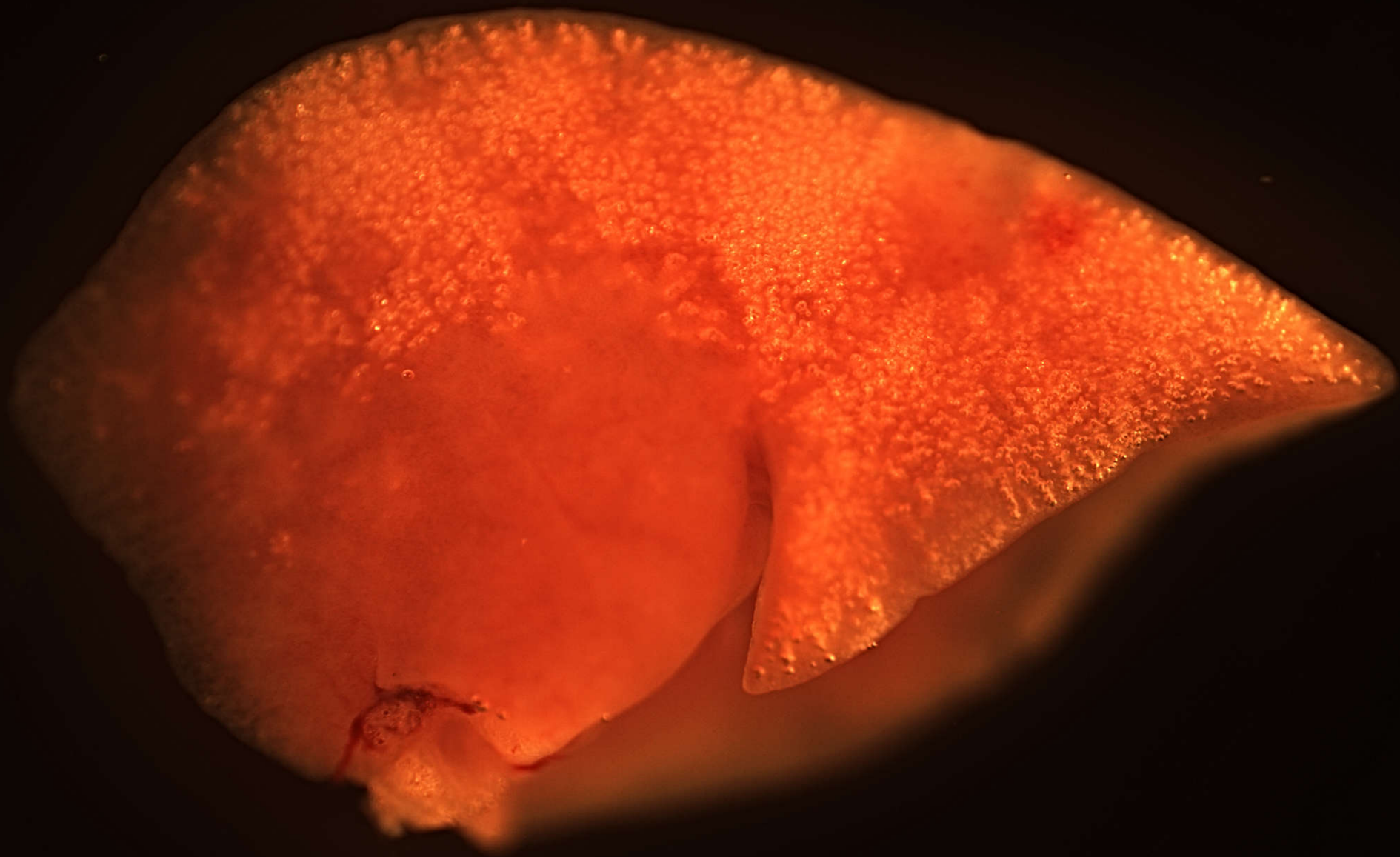

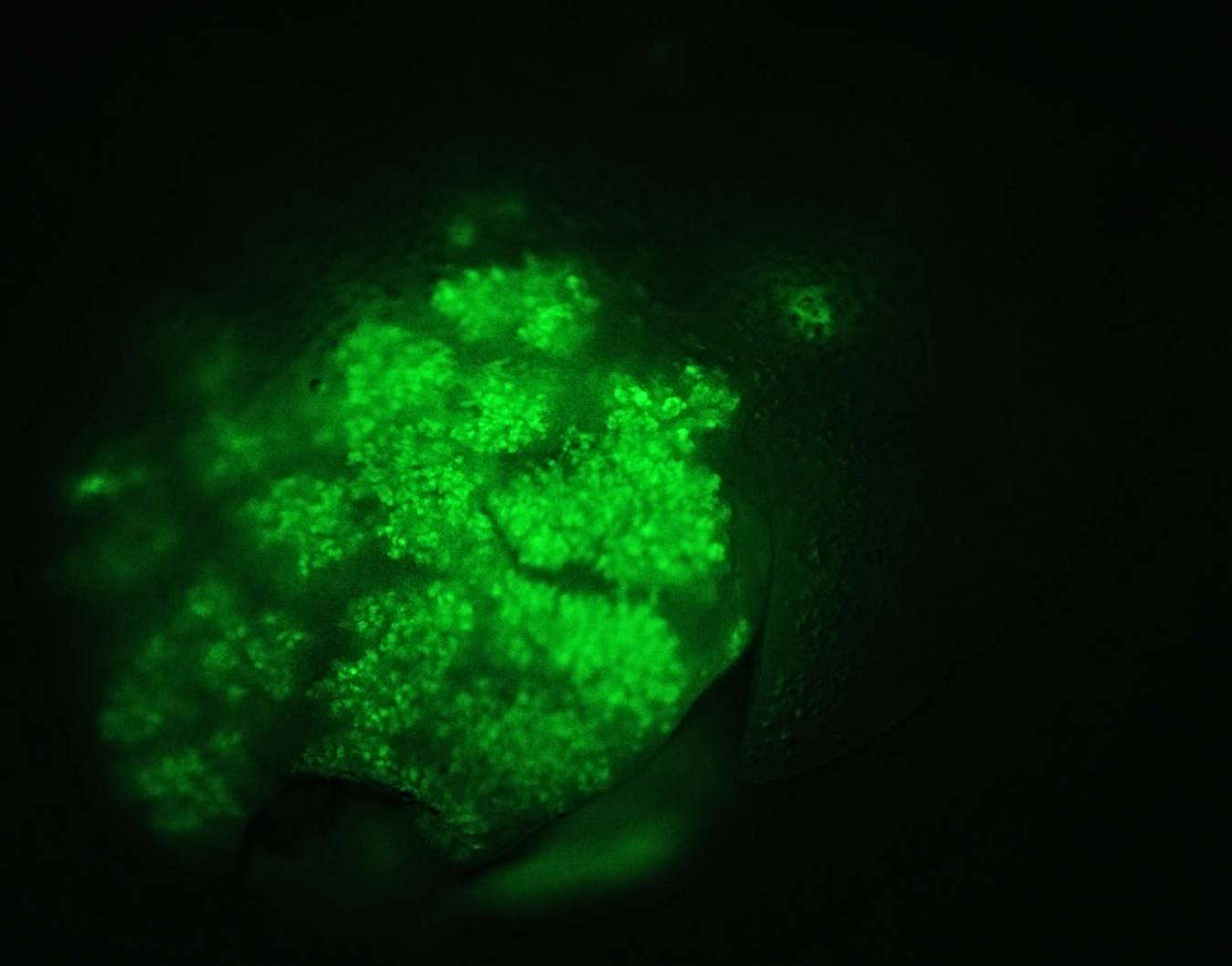

Supplement: Supplementary file 8 — Source Data for Figure 4 [file EMMM-12-e10233-s007.zip › Figure_4A_WT-lung_7days.pdf]

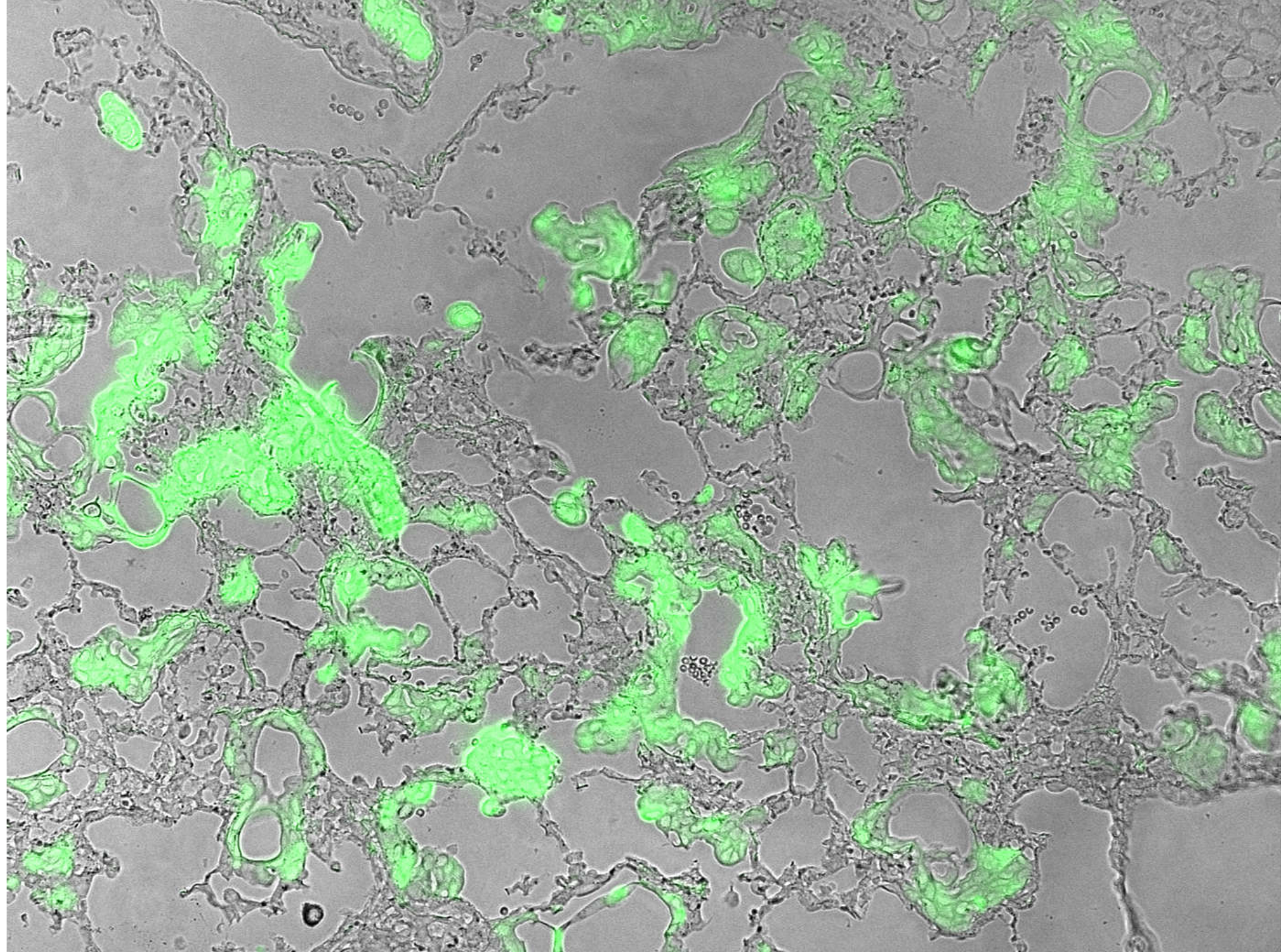

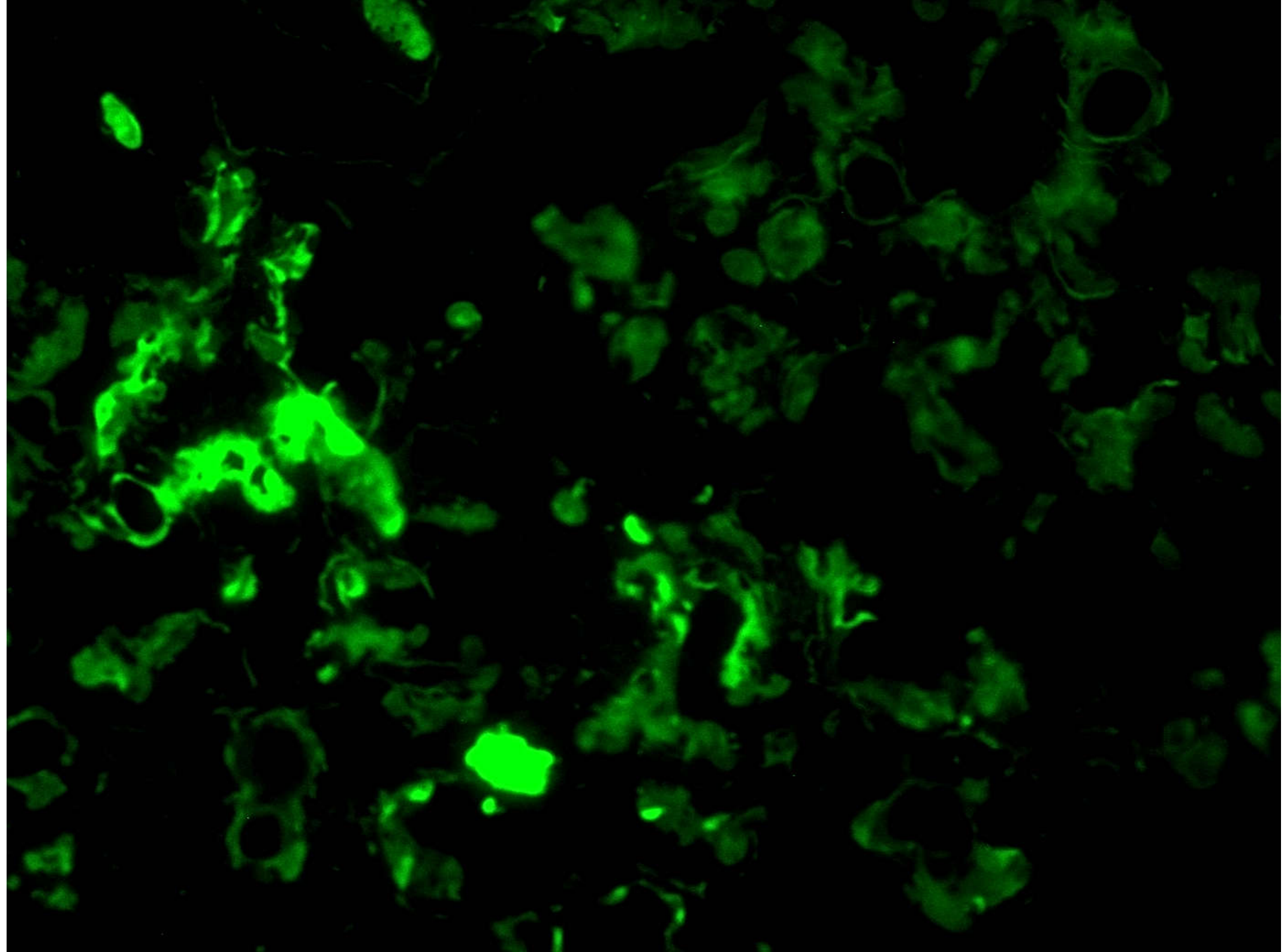

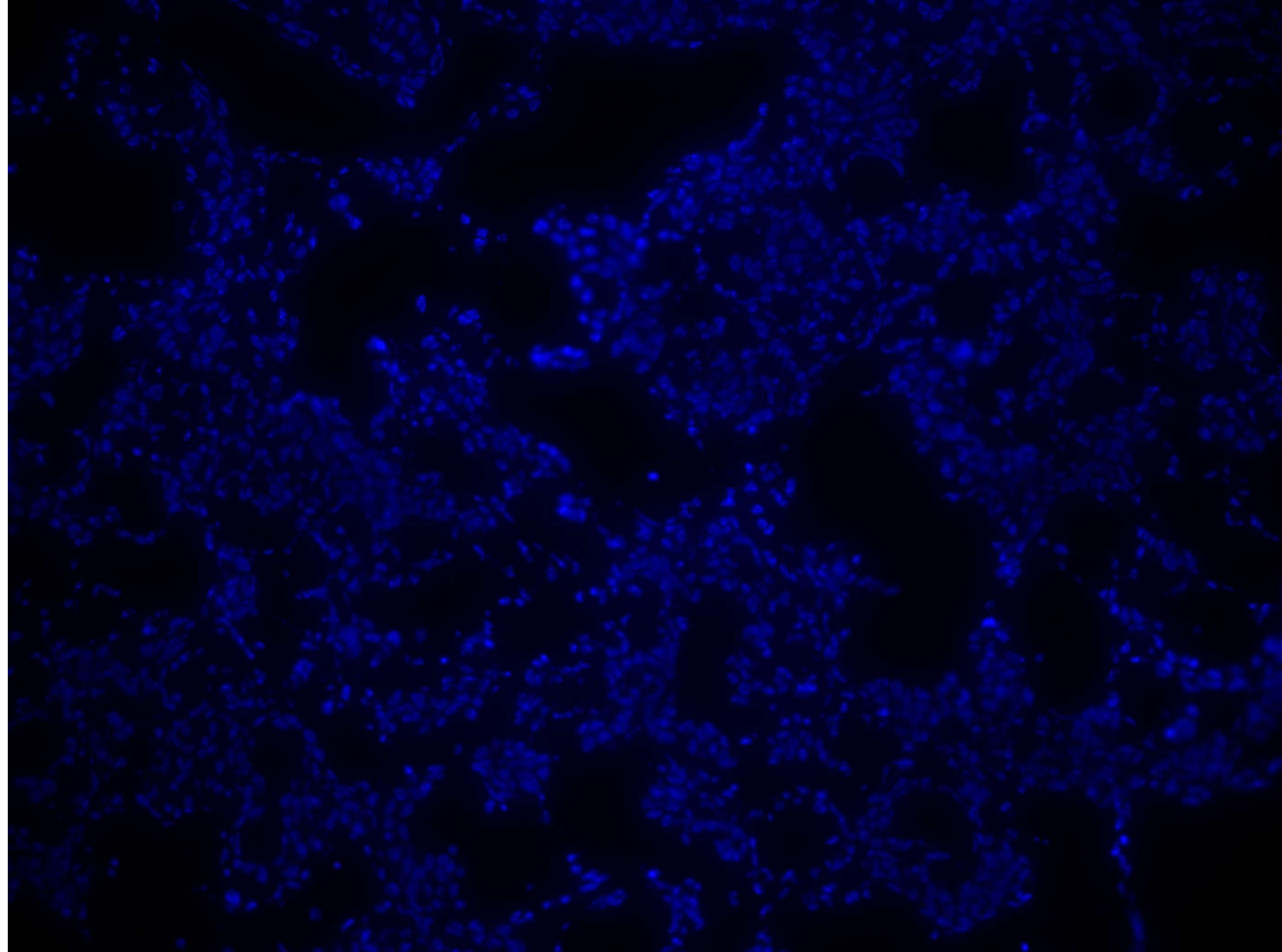

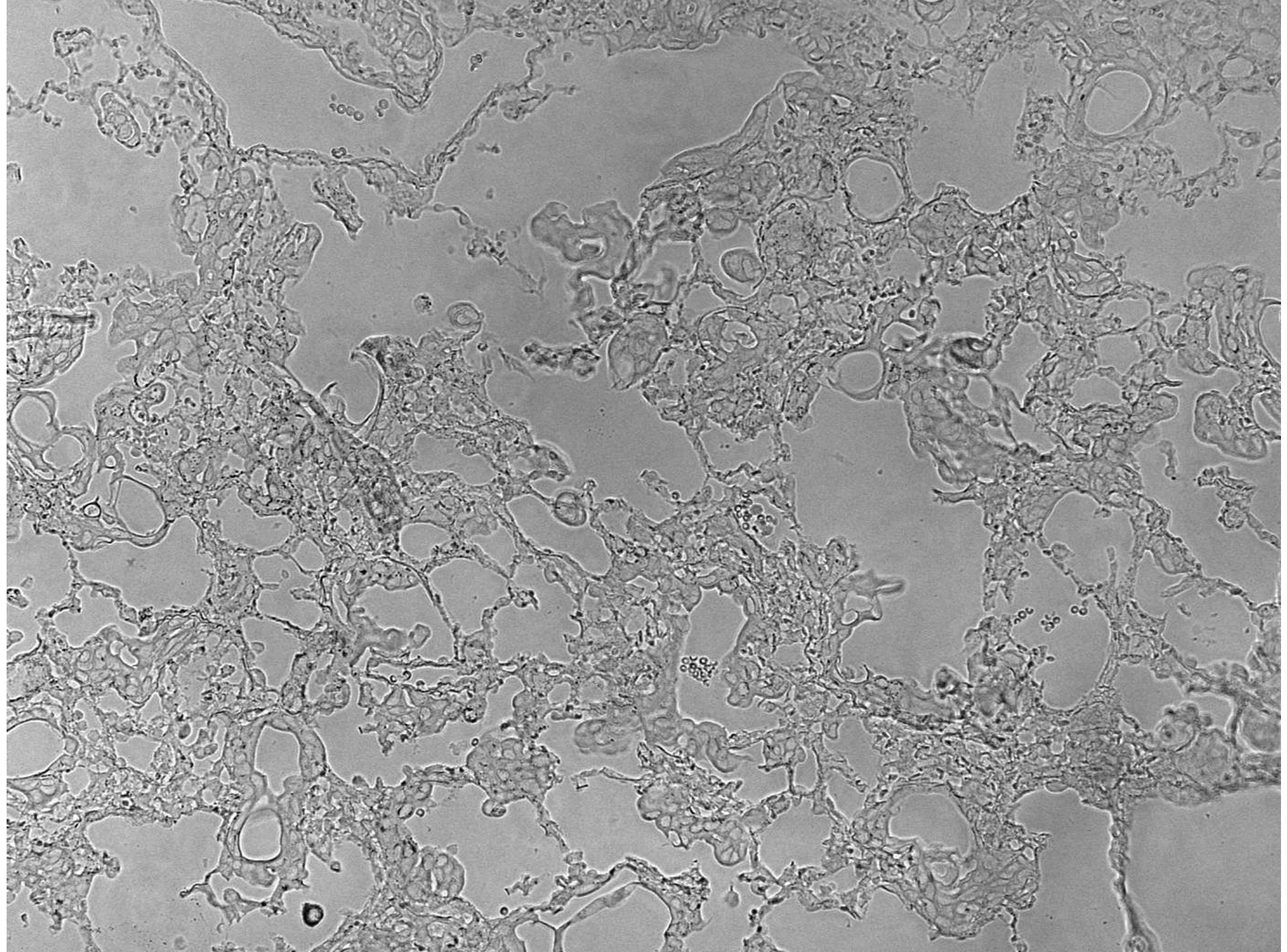

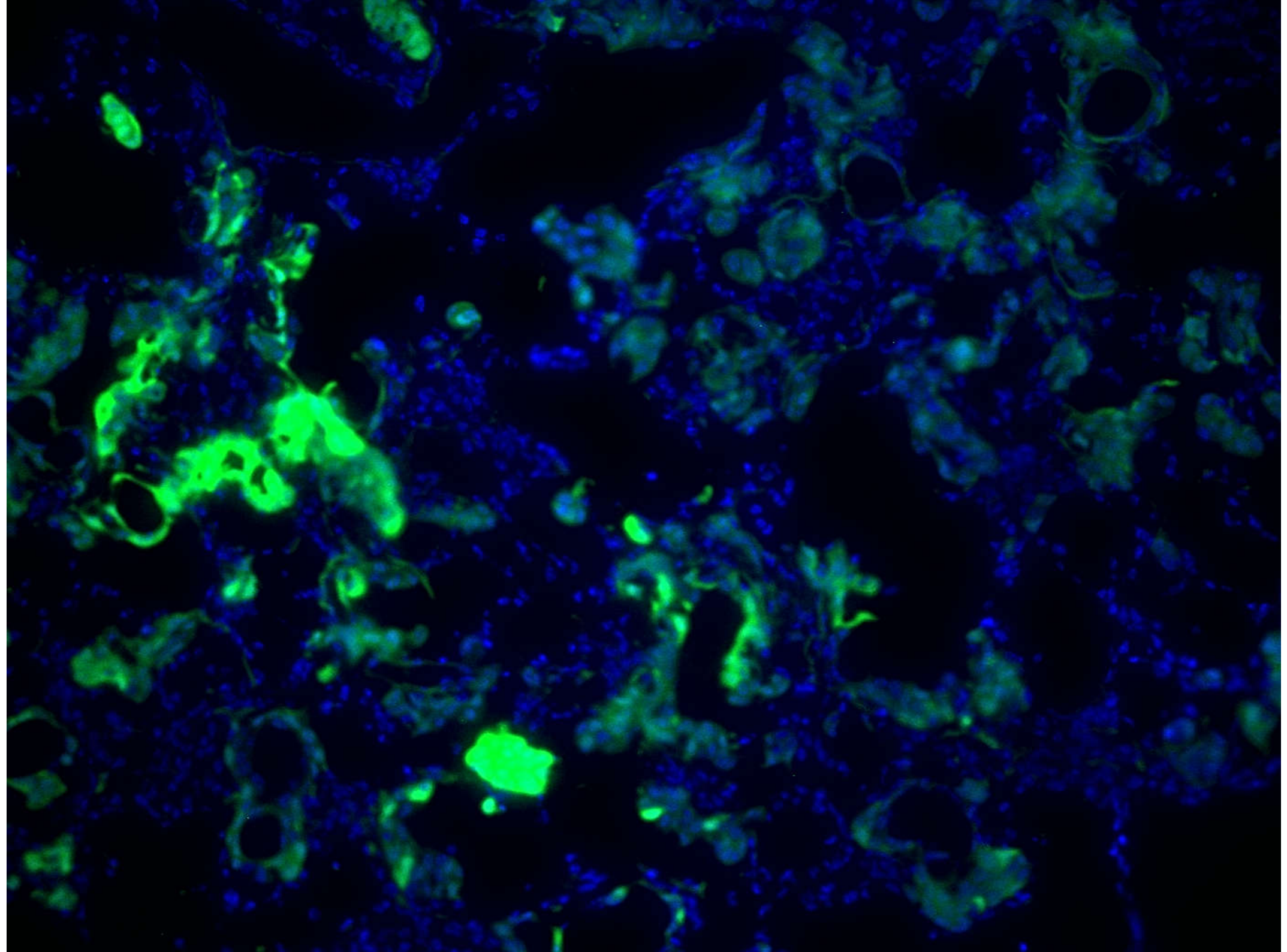

Supplement: Supplementary file 8 — Source Data for Figure 4 [file EMMM-12-e10233-s007.zip › Figure_4C_LL-37-lung_14days.pdf]

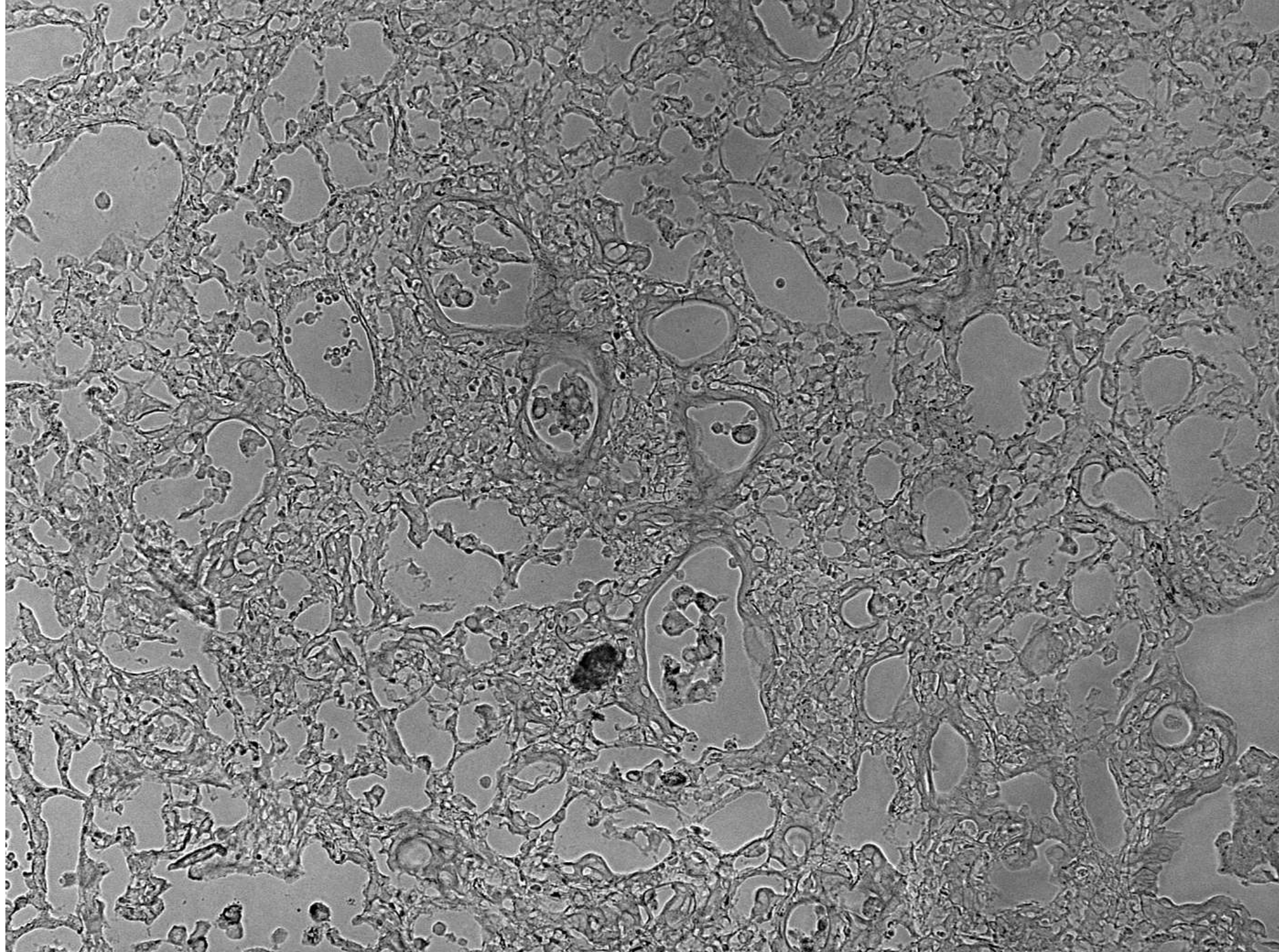

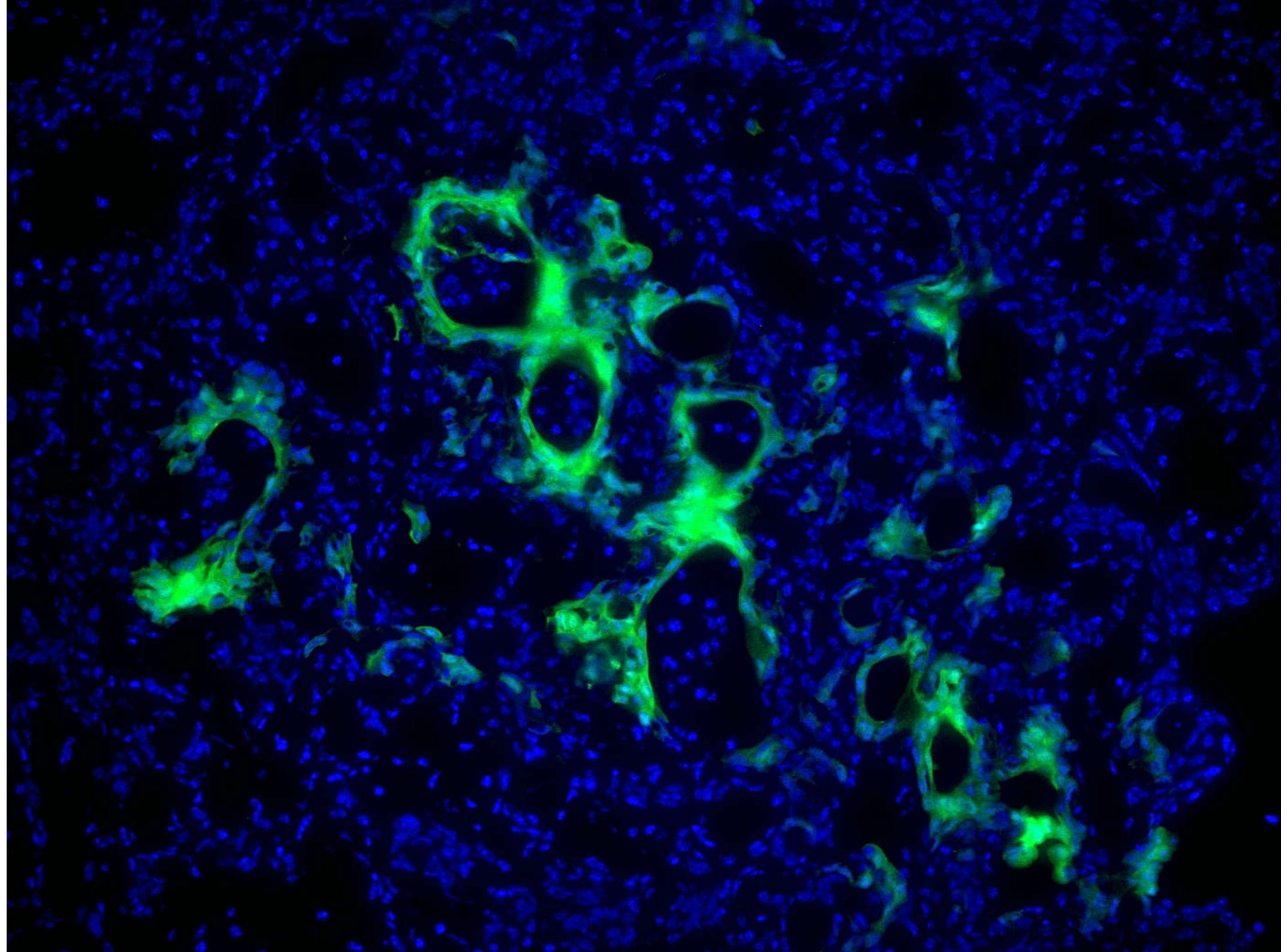

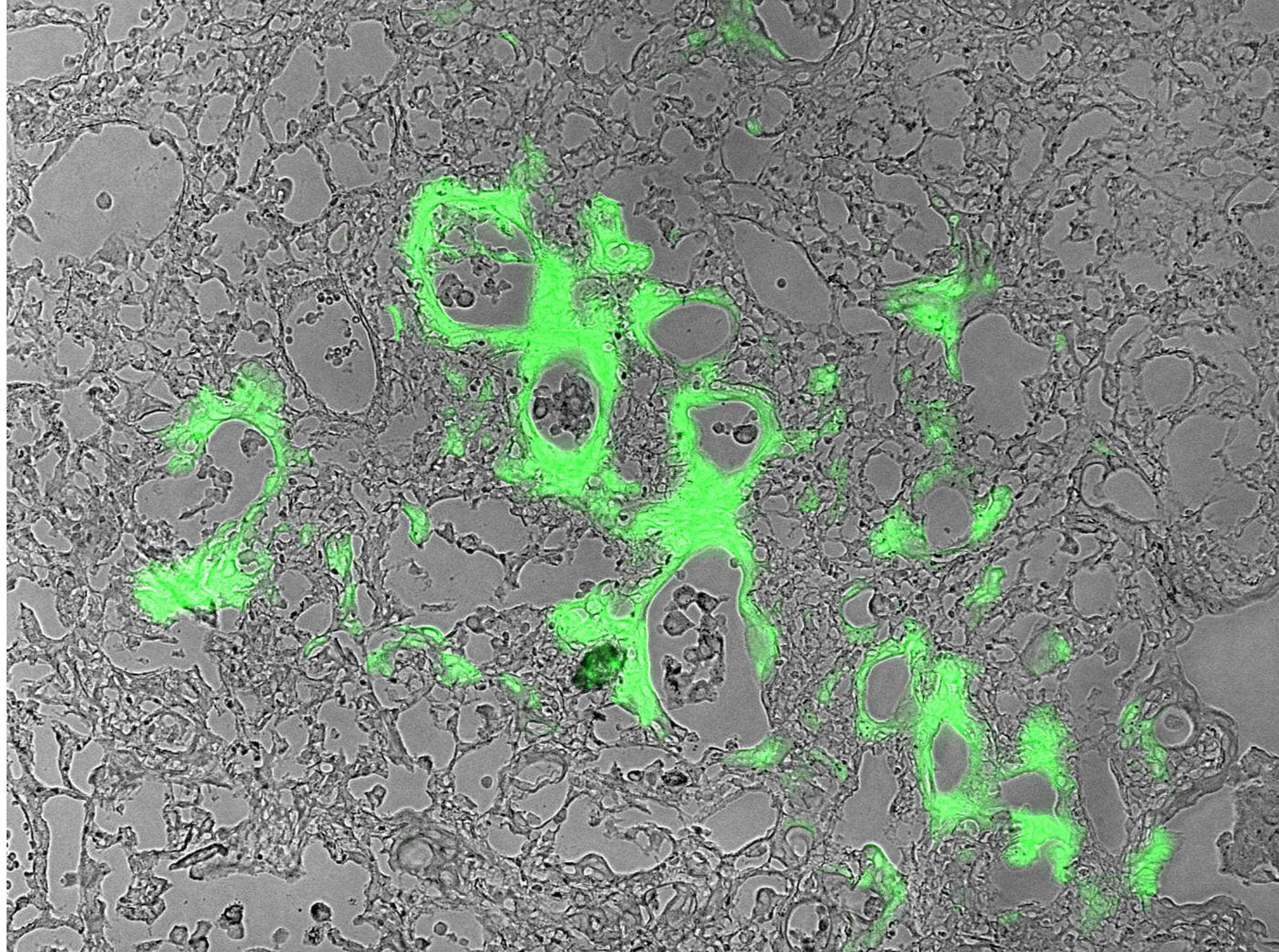

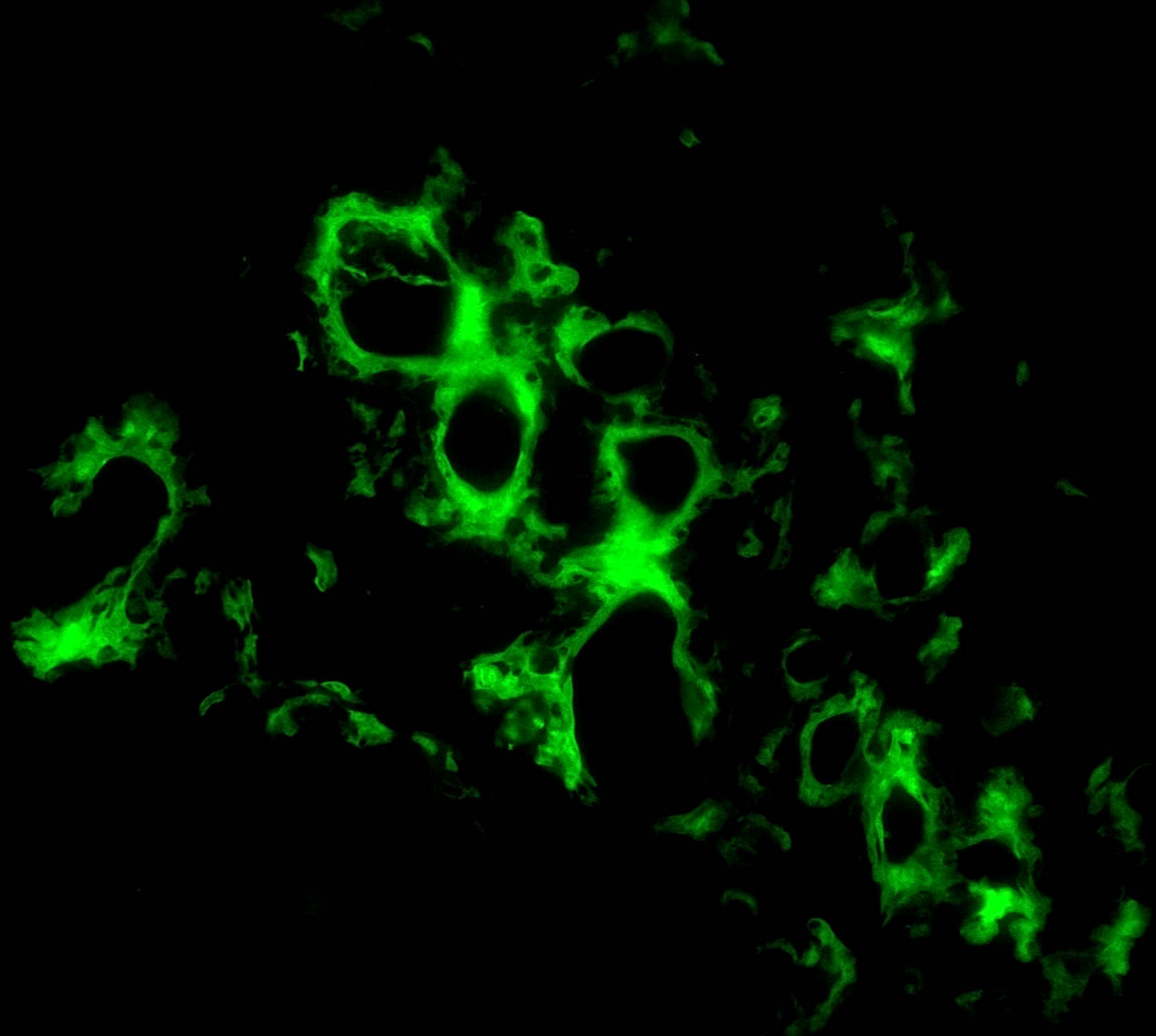

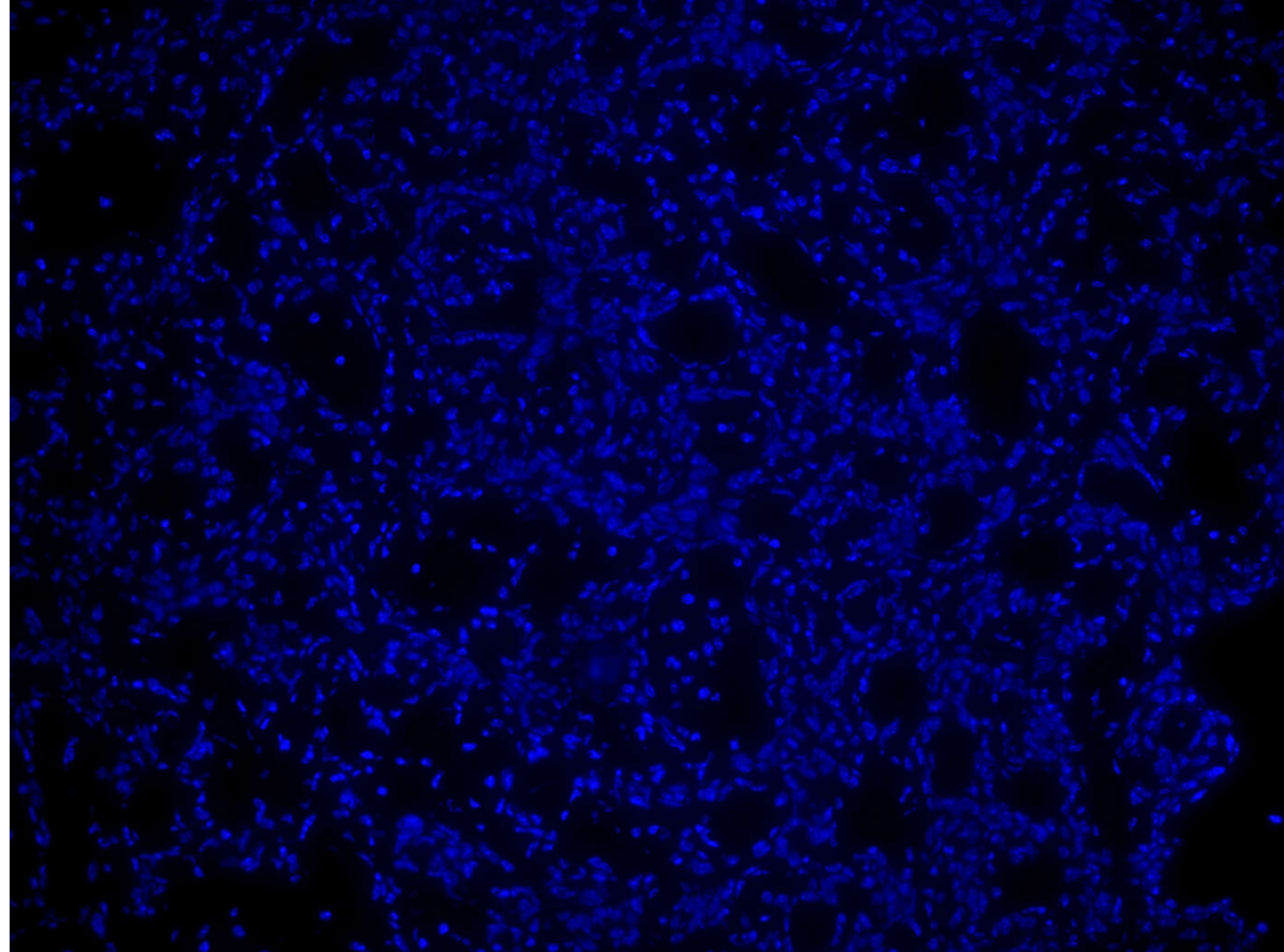

Supplement: Supplementary file 8 — Source Data for Figure 4 [file EMMM-12-e10233-s007.zip › Figure_4C_LL-37-lung_21days.pdf]

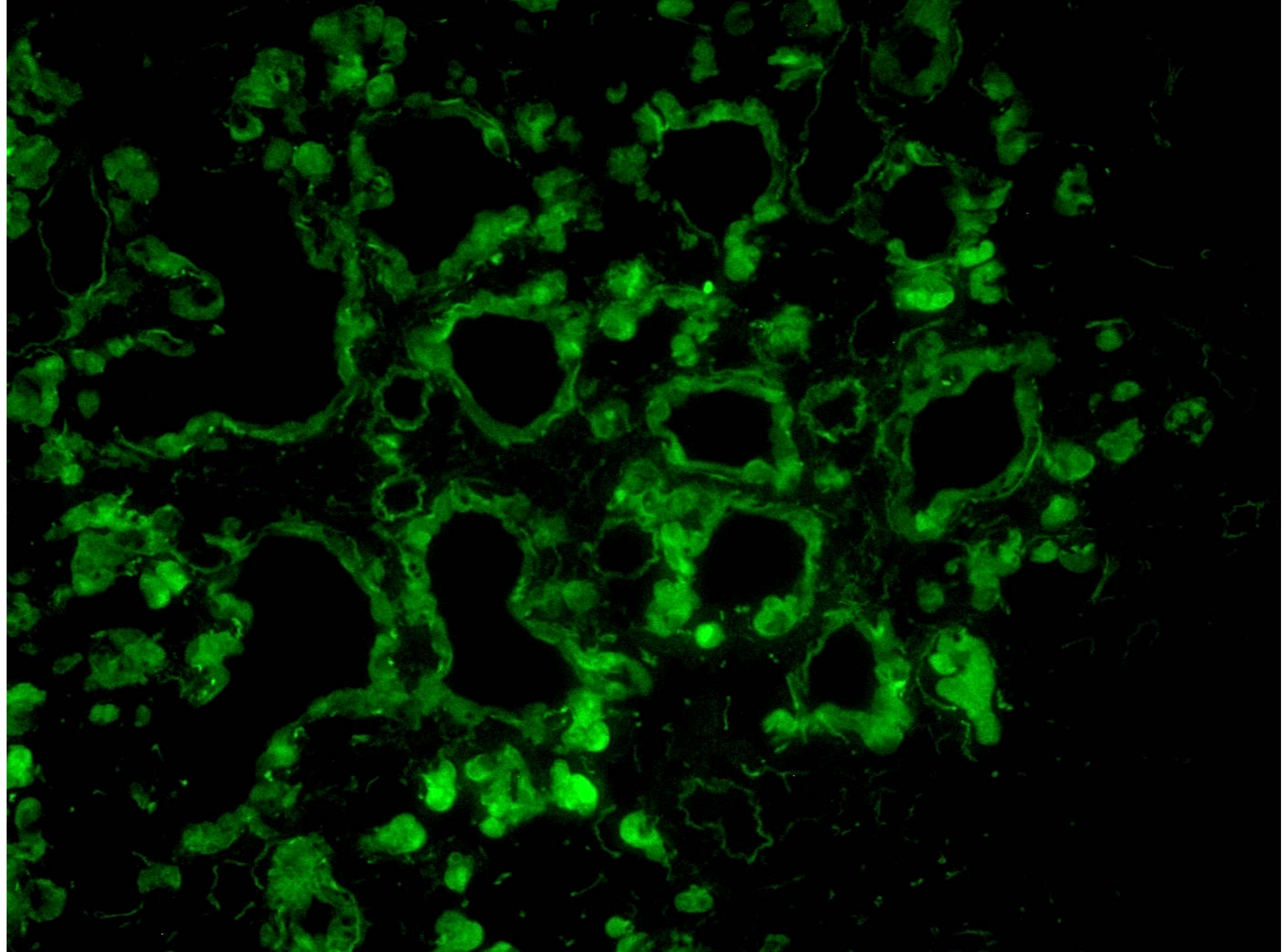

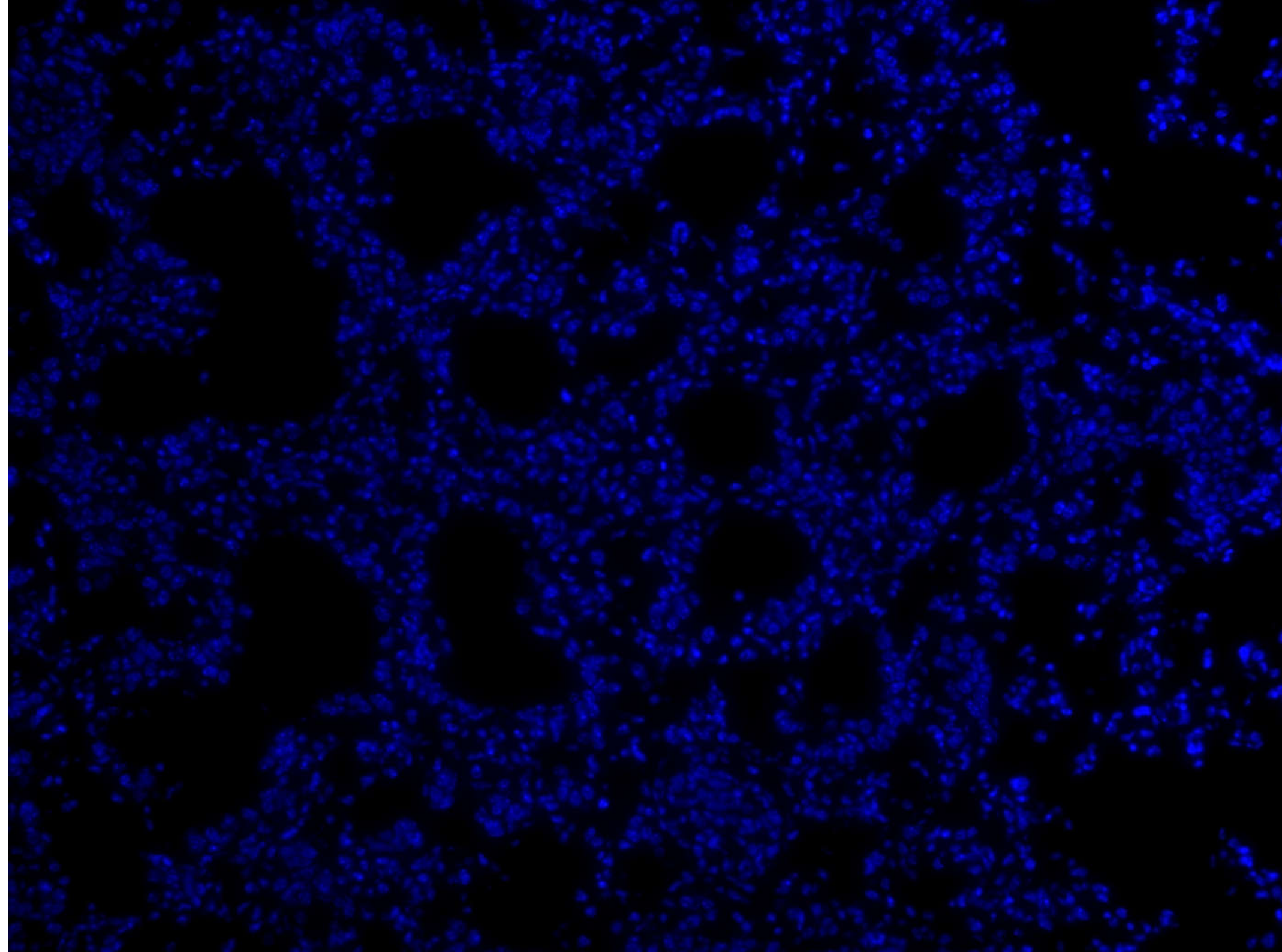

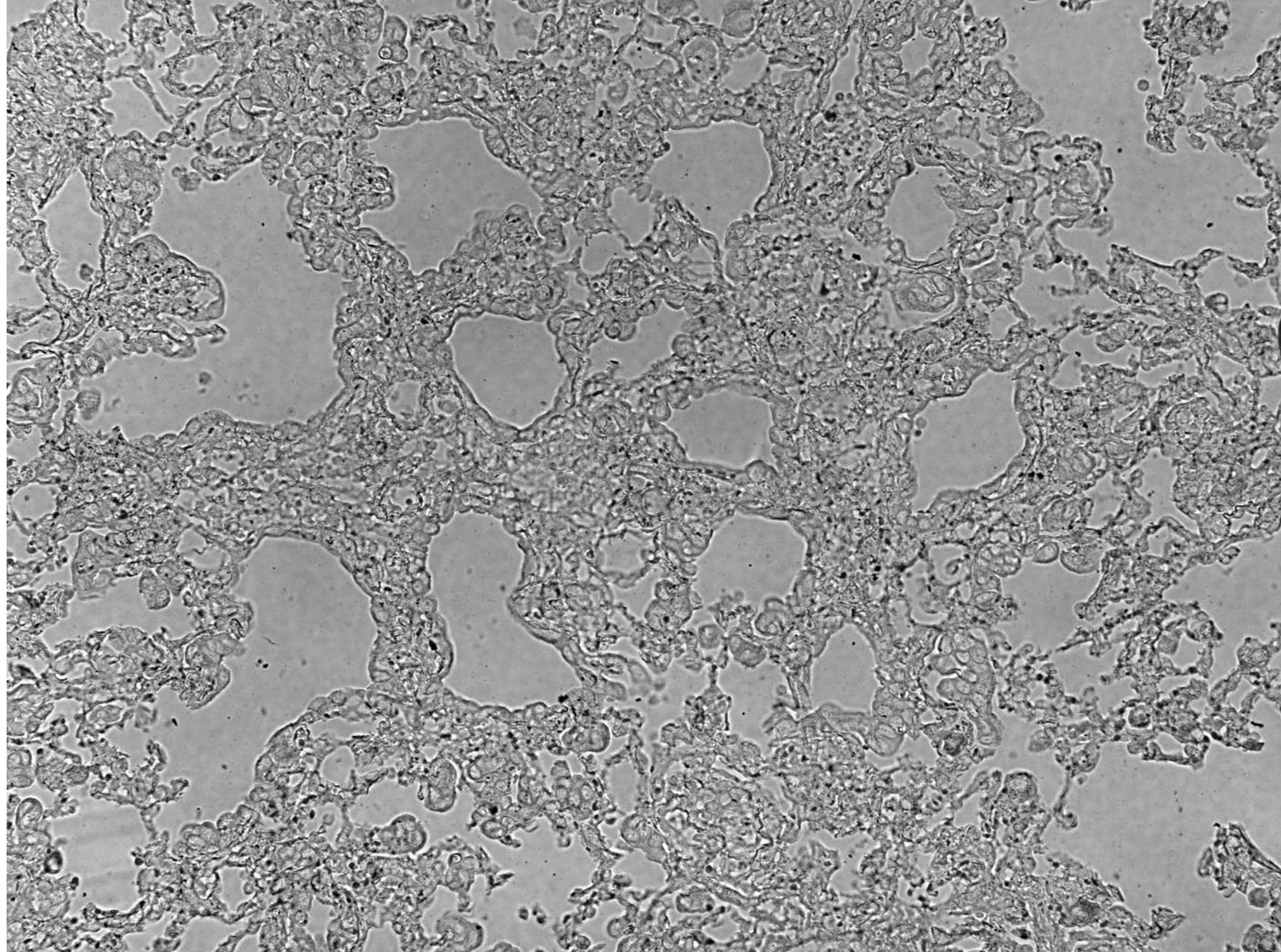

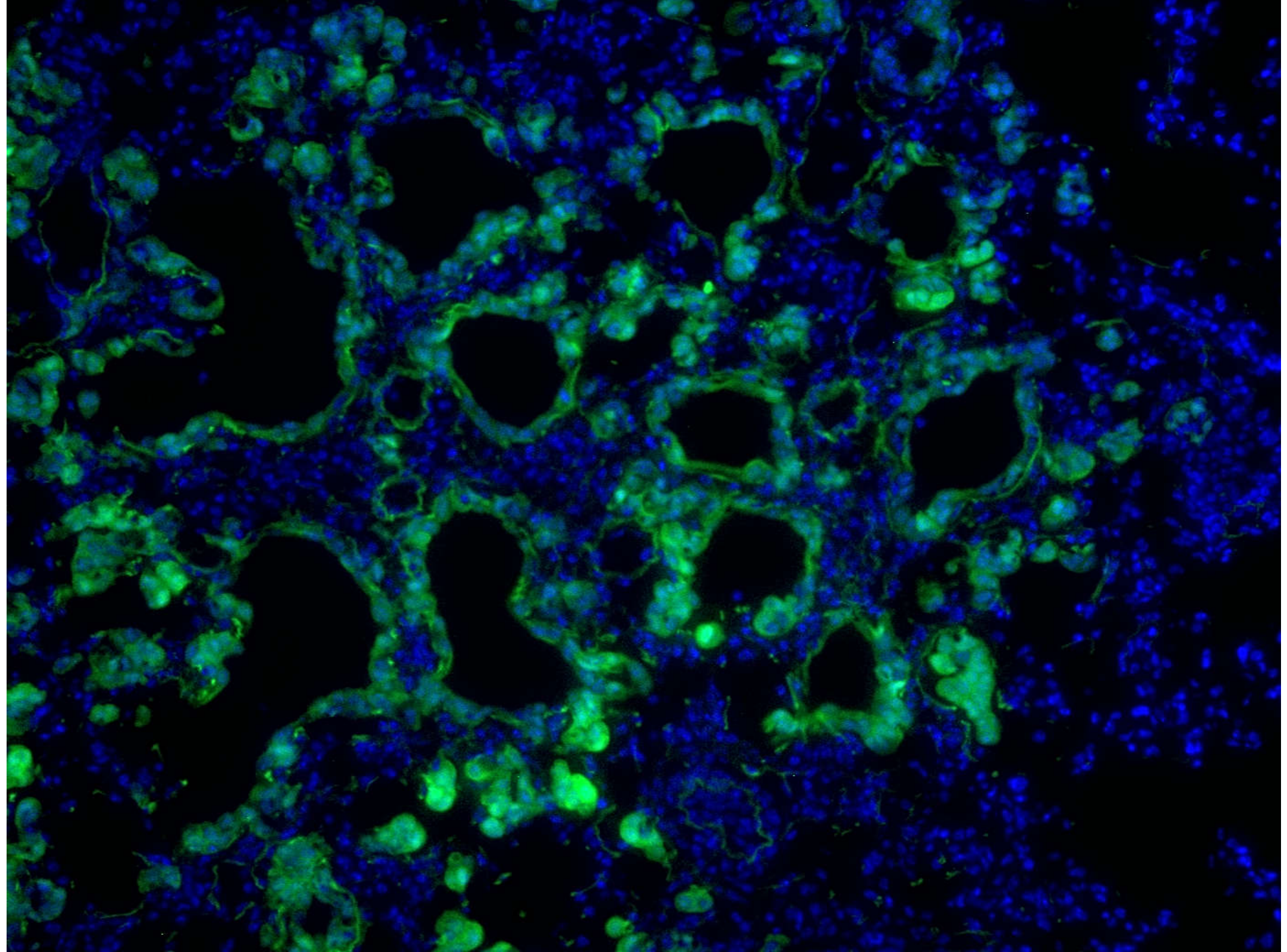

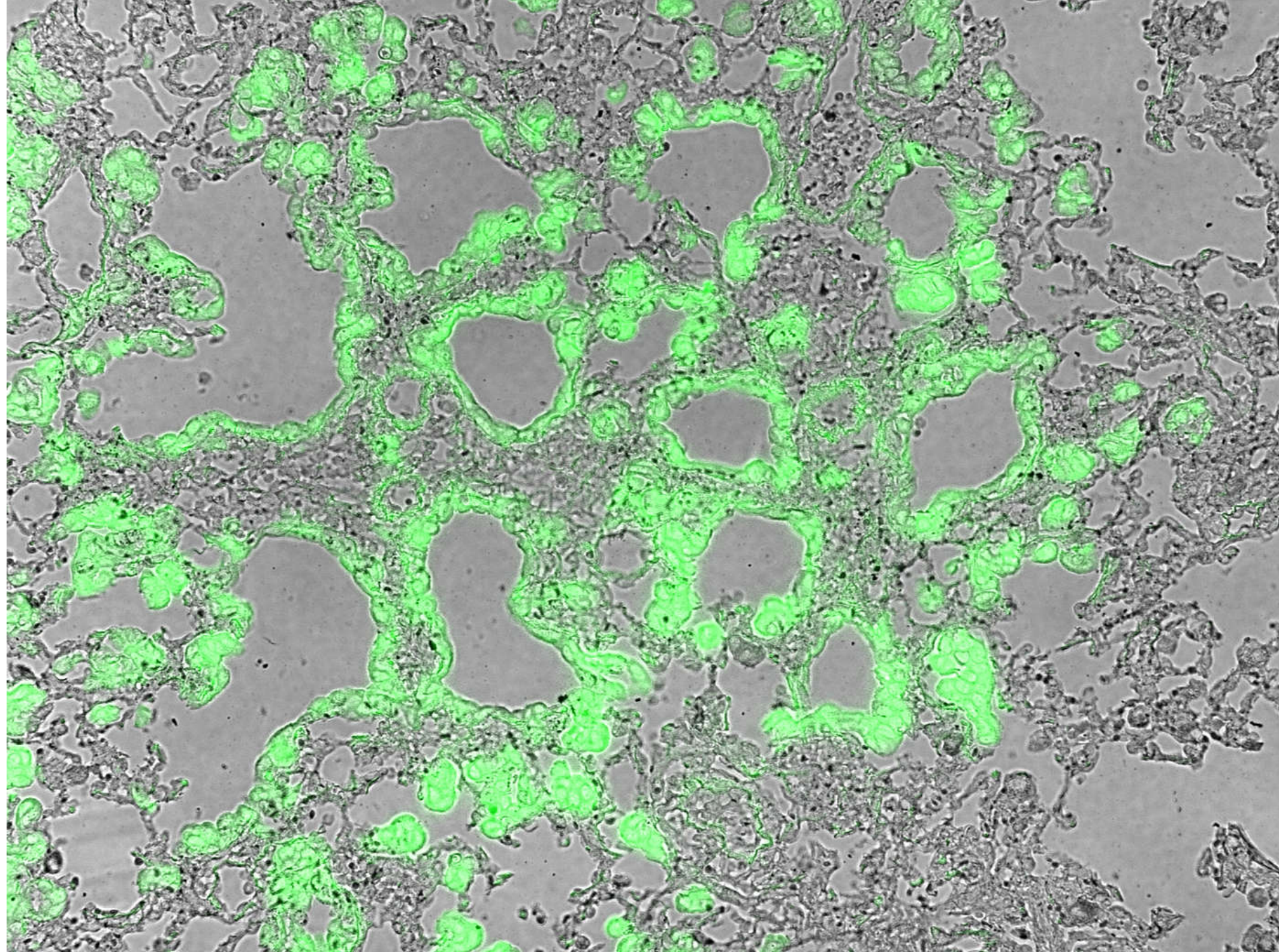

Supplement: Supplementary file 8 — Source Data for Figure 4 [file EMMM-12-e10233-s007.zip › Figure_4C_LL-37-lung_28days.pdf]

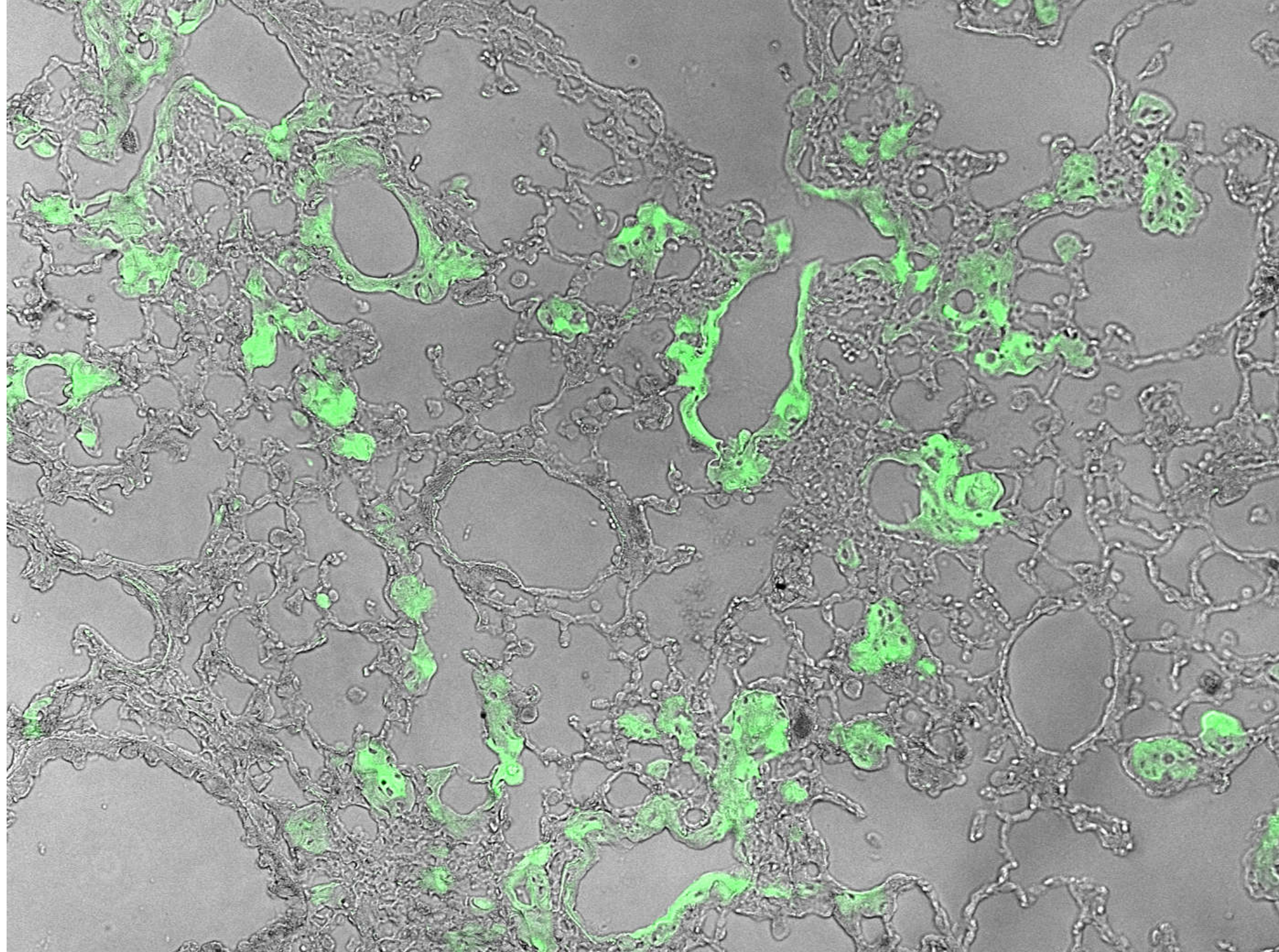

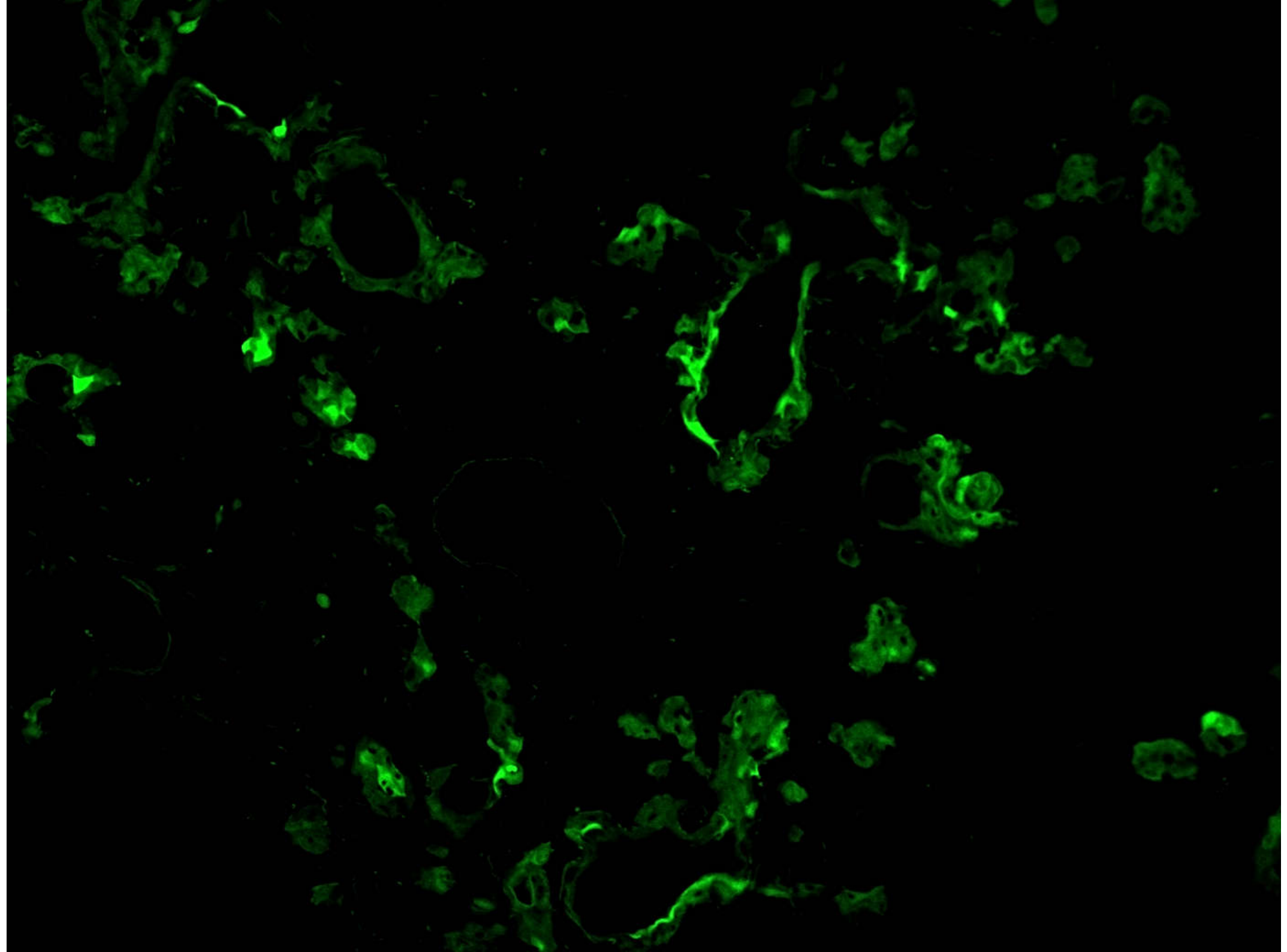

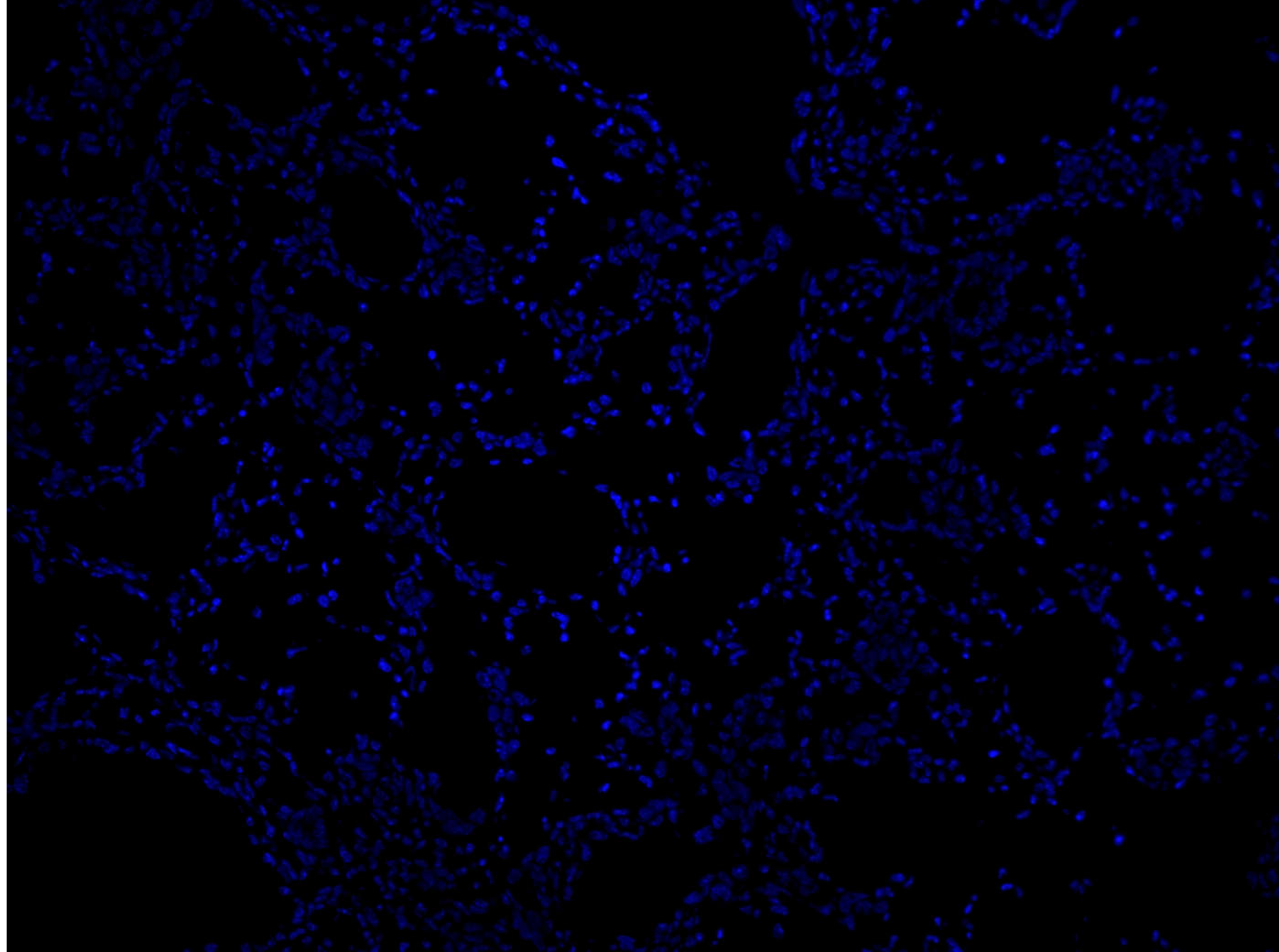

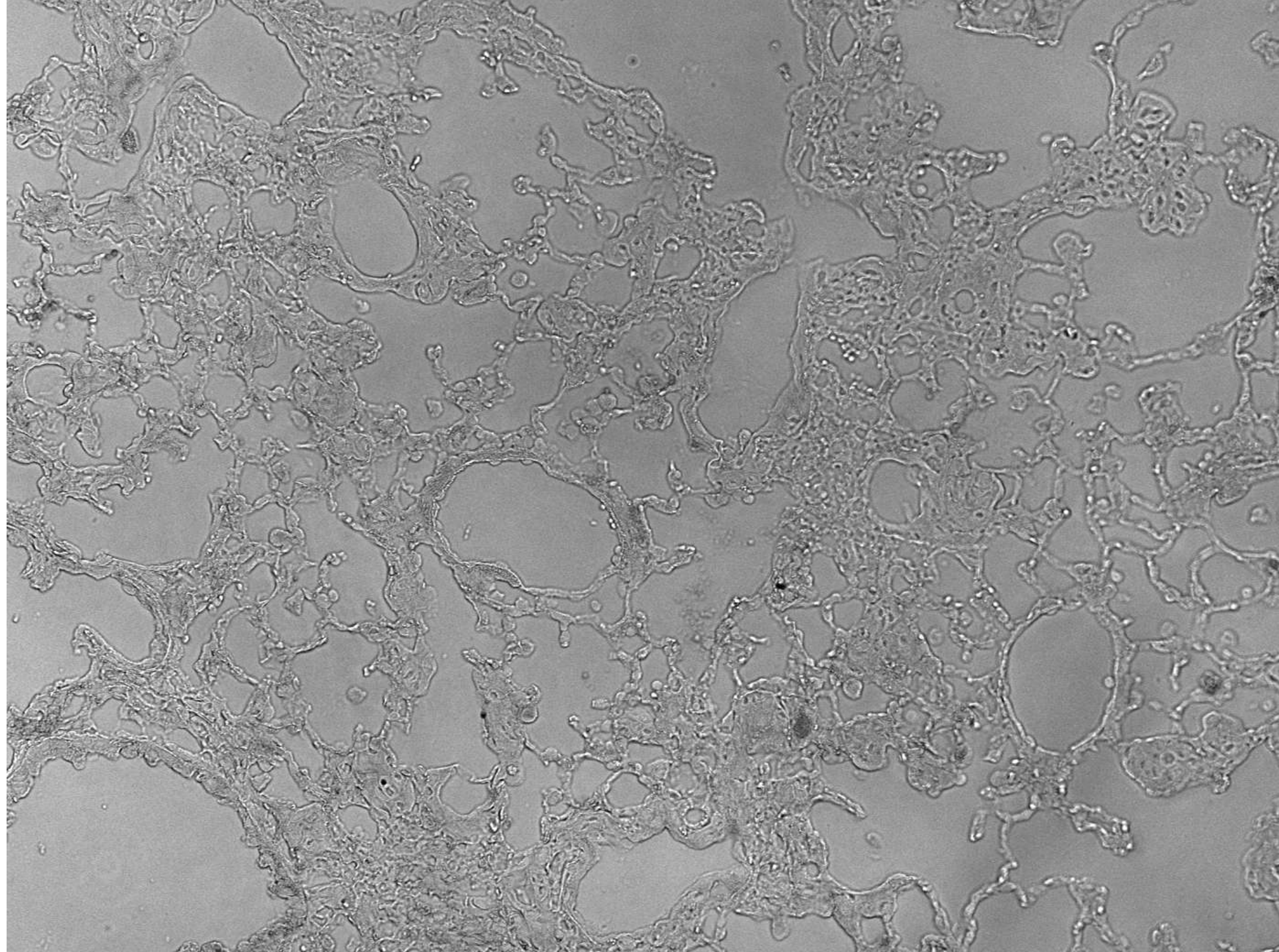

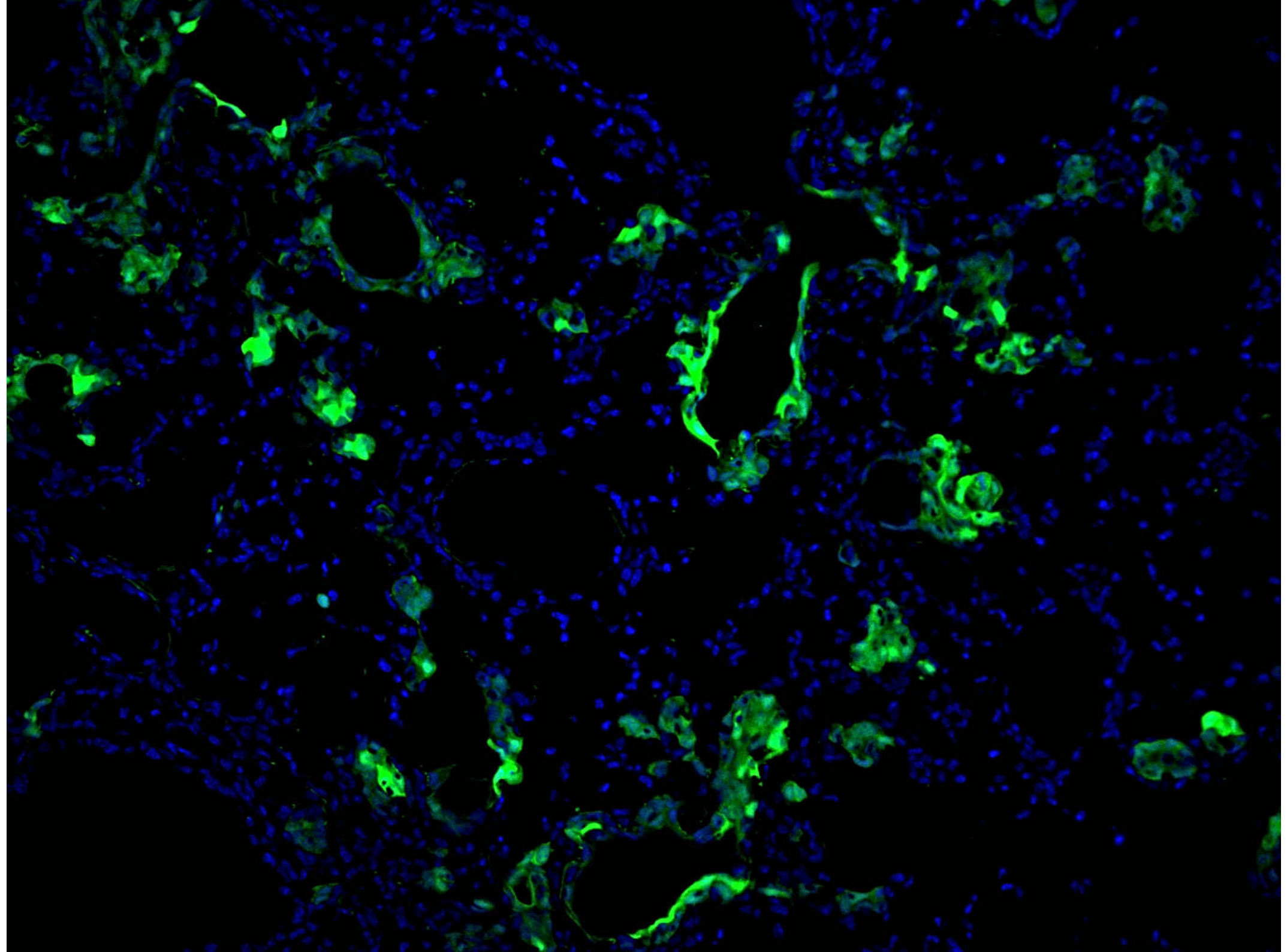

Supplement: Supplementary file 8 — Source Data for Figure 4 [file EMMM-12-e10233-s007.zip › Figure_4C_LL-37-lung_7days.pdf]

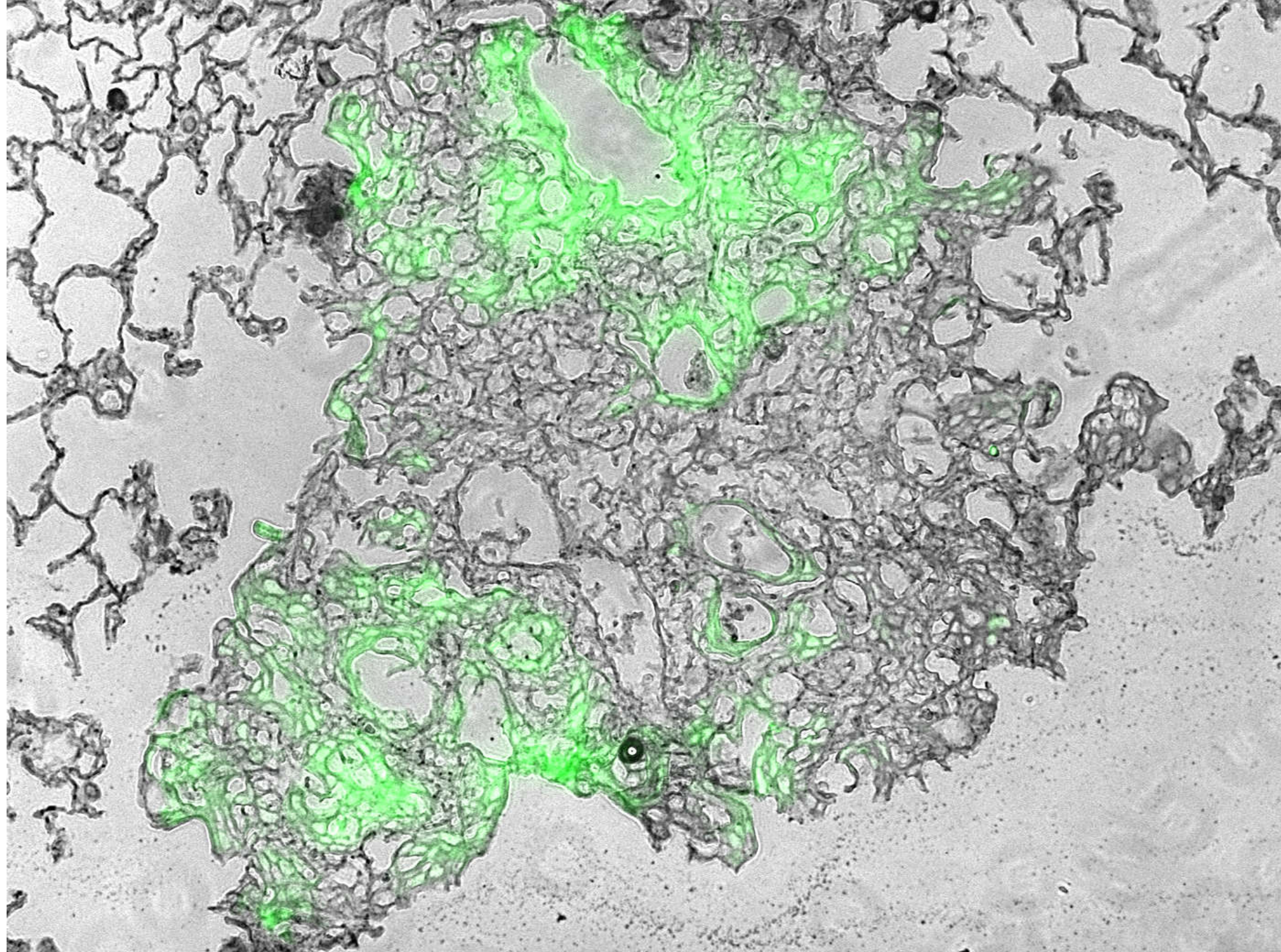

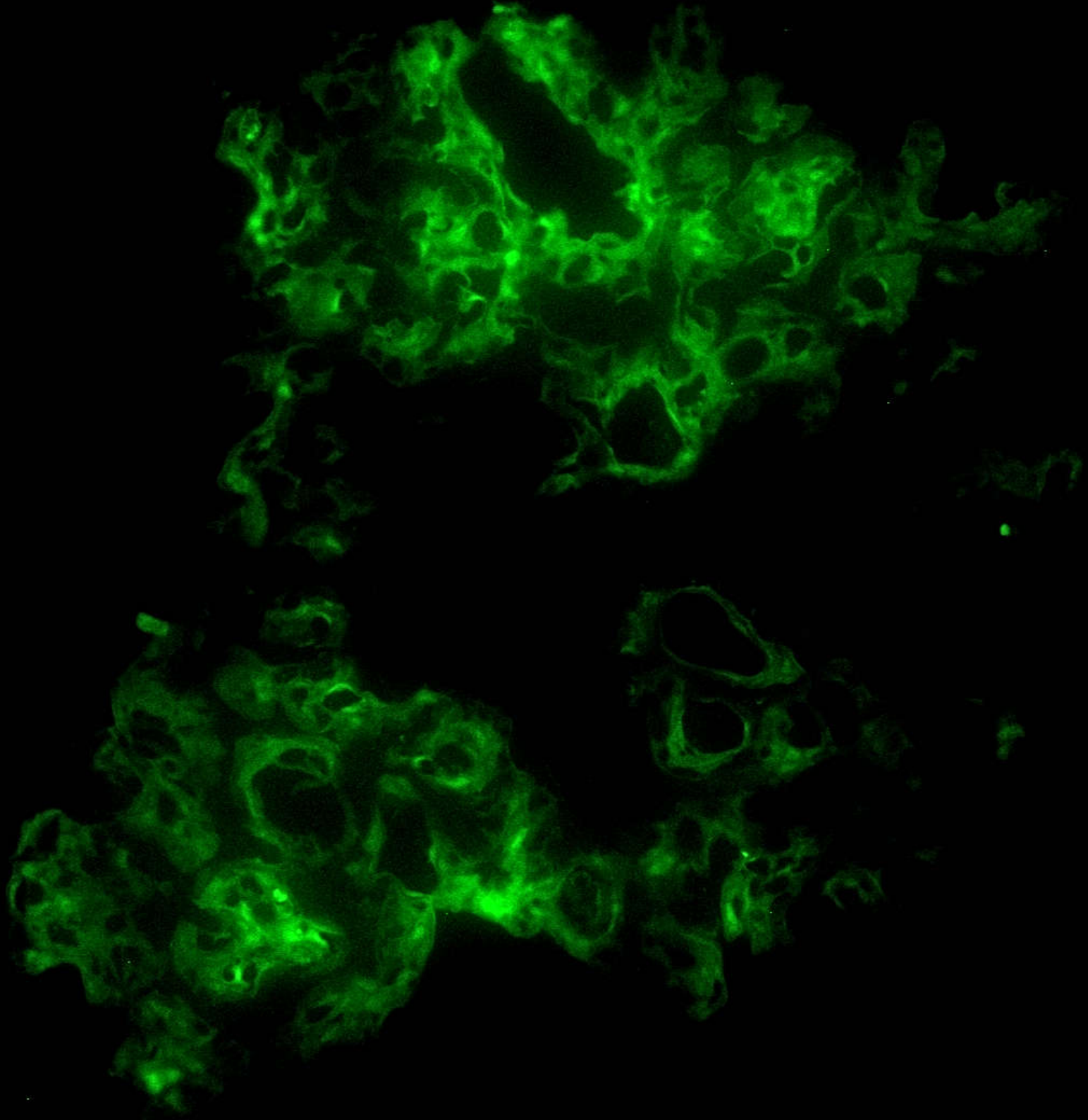

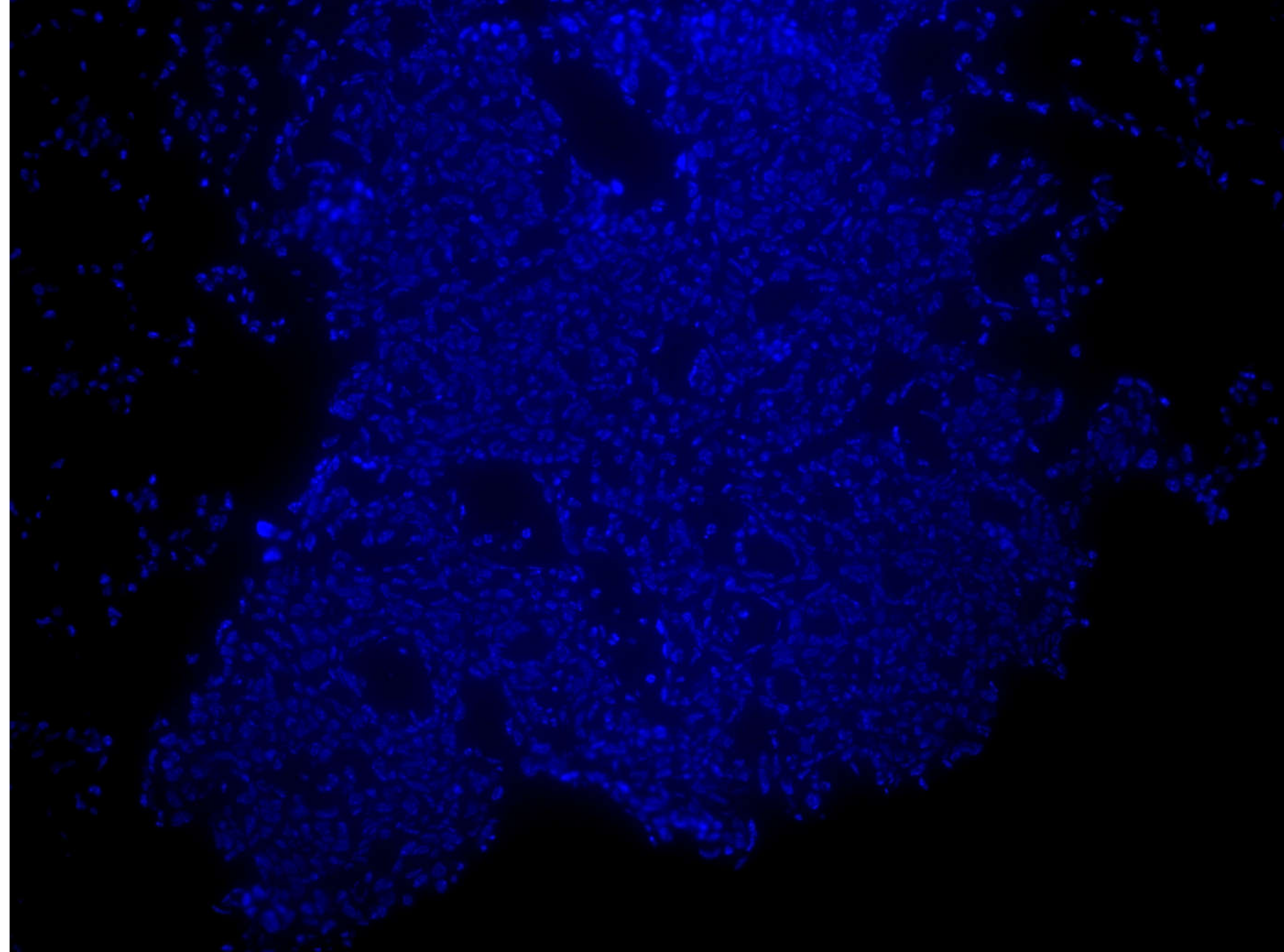

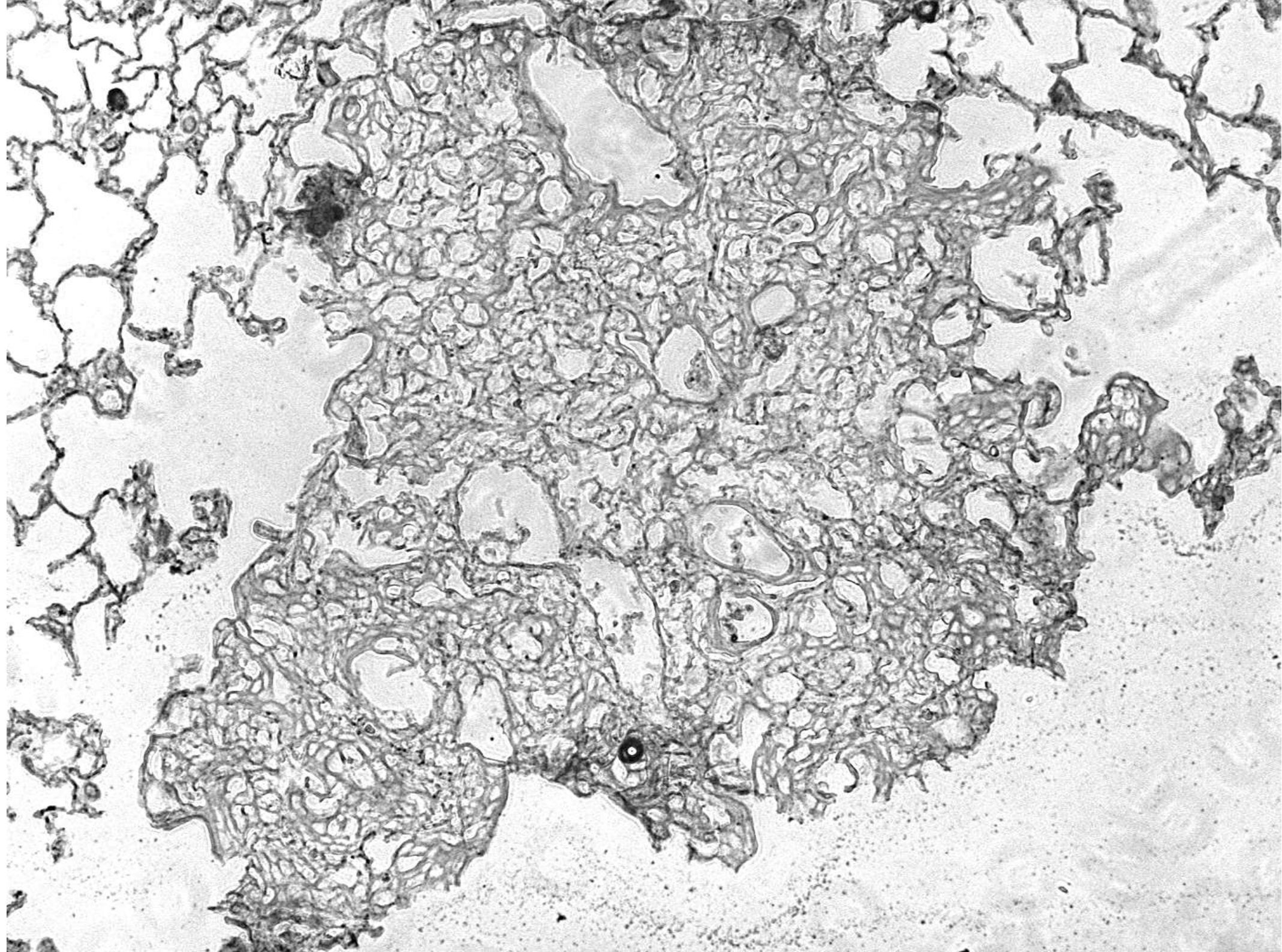

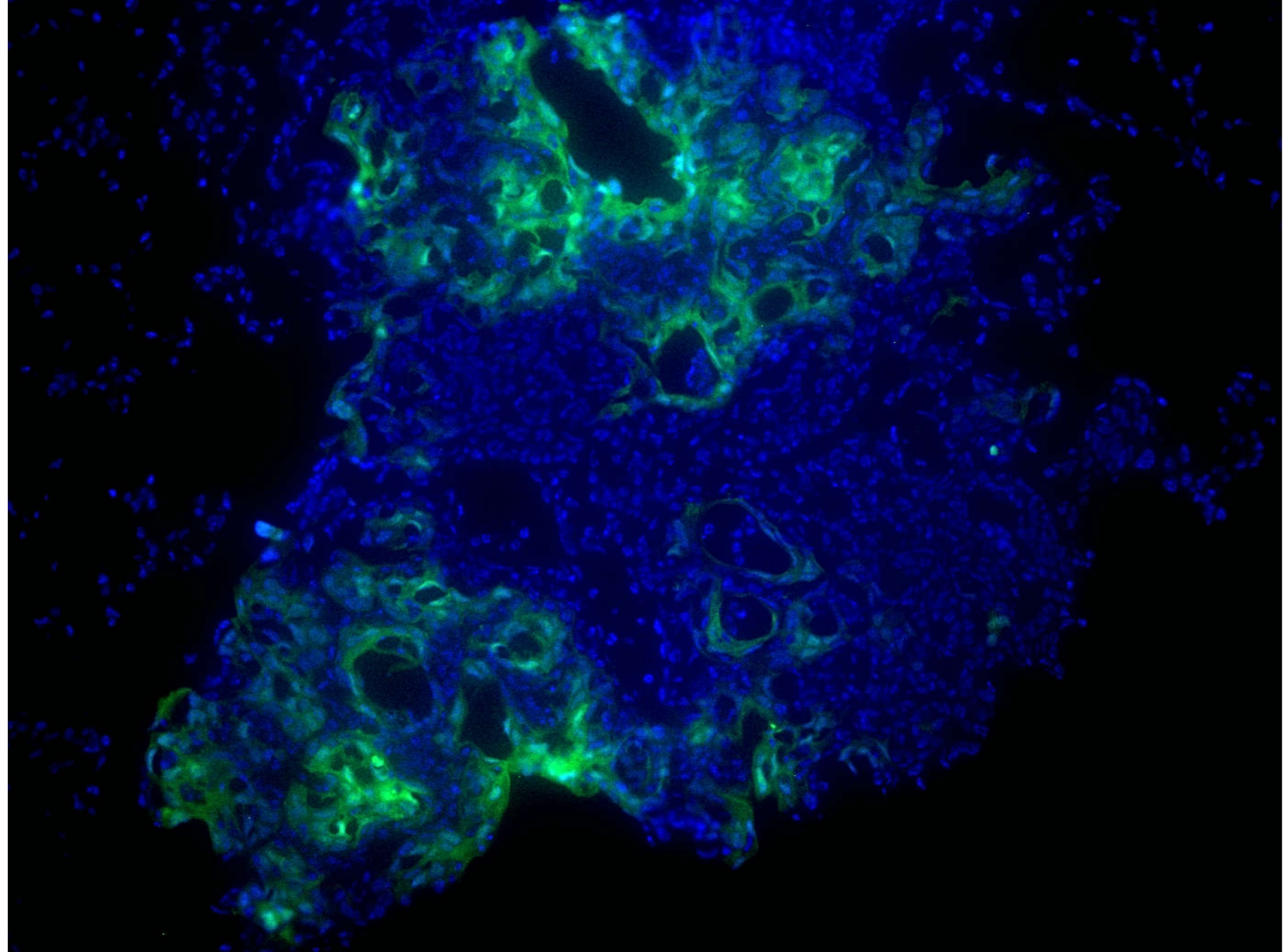

Supplement: Supplementary file 8 — Source Data for Figure 4 [file EMMM-12-e10233-s007.zip › Figure_4C_WT-lung_14days.pdf]

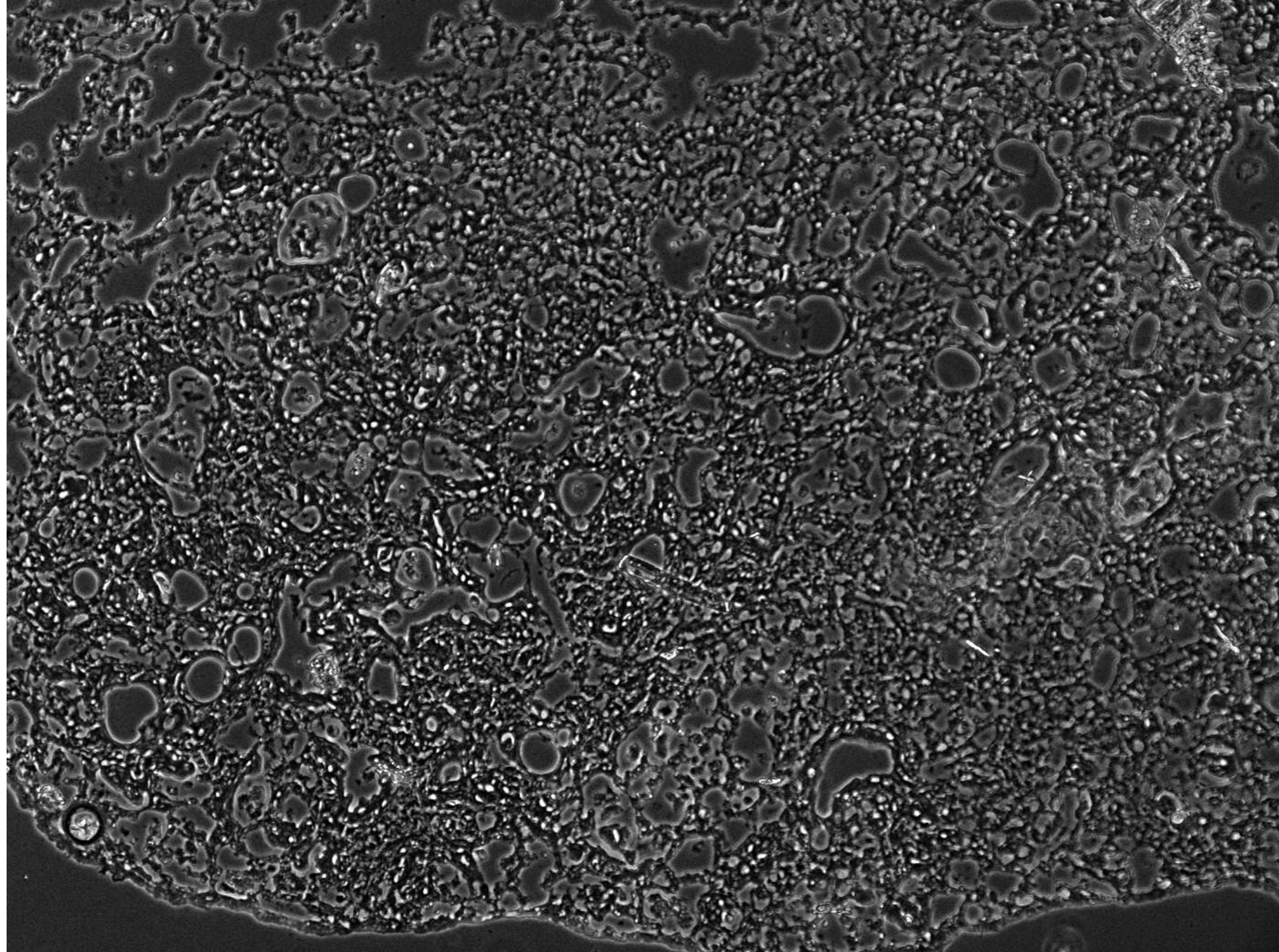

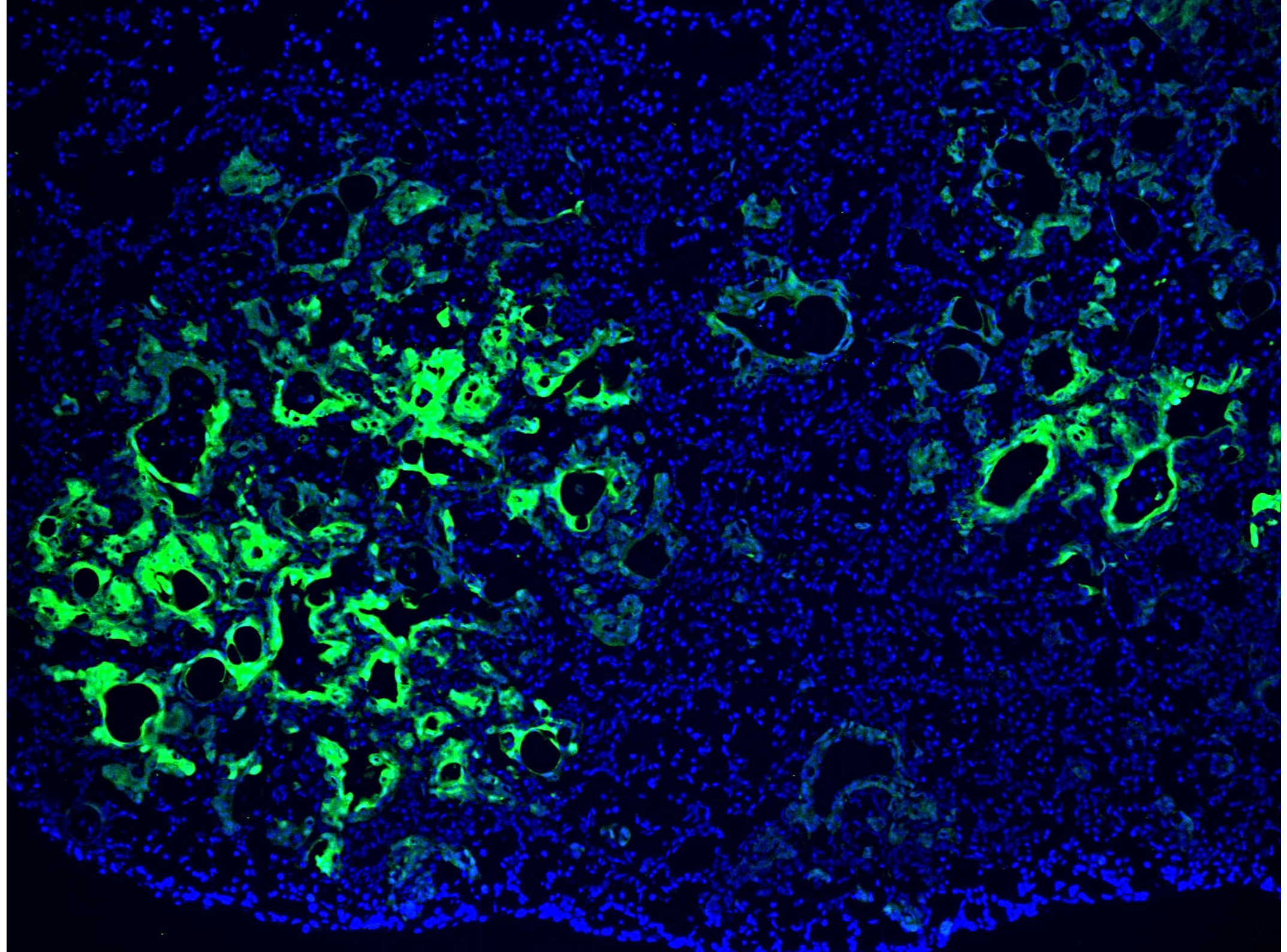

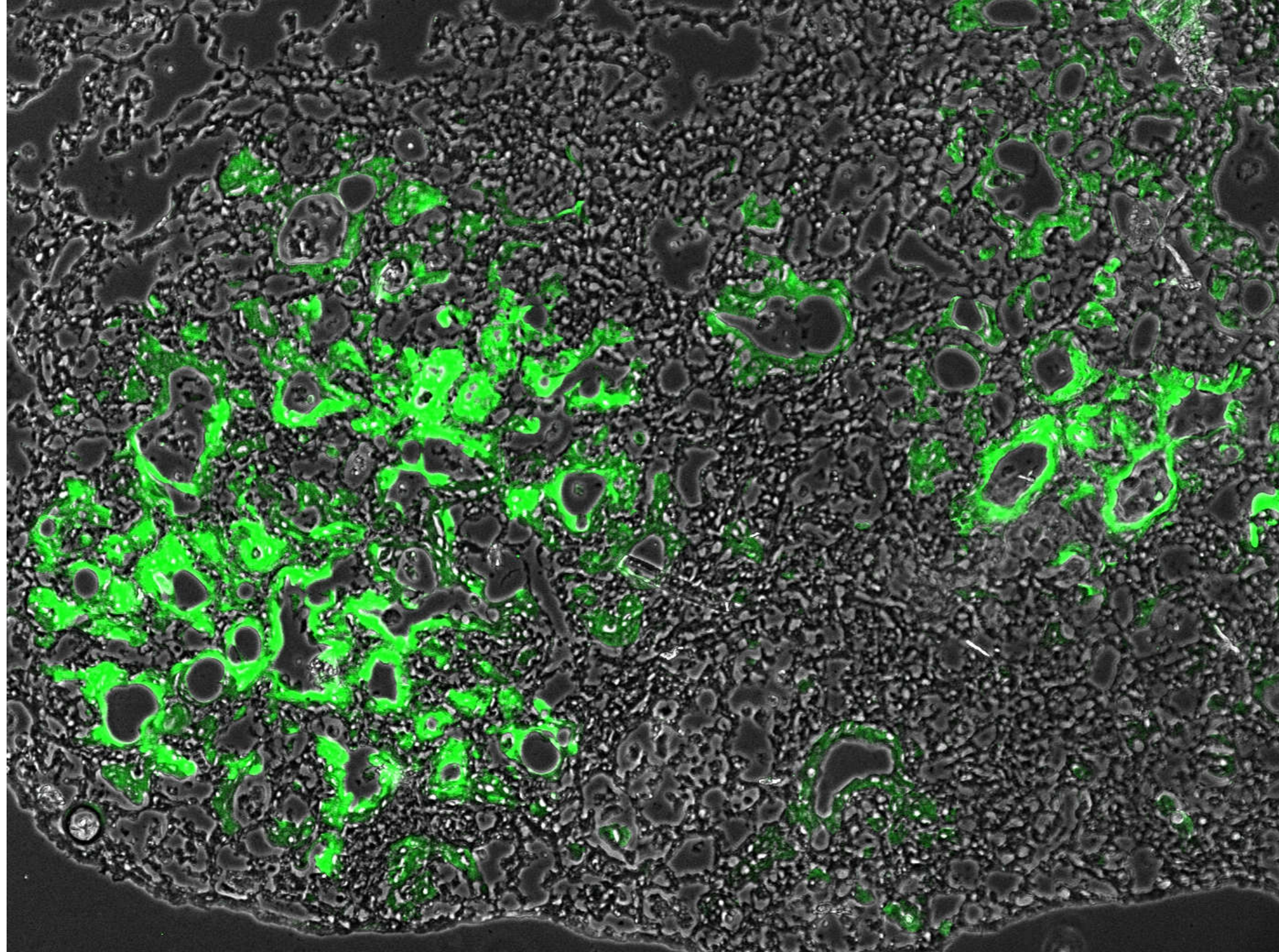

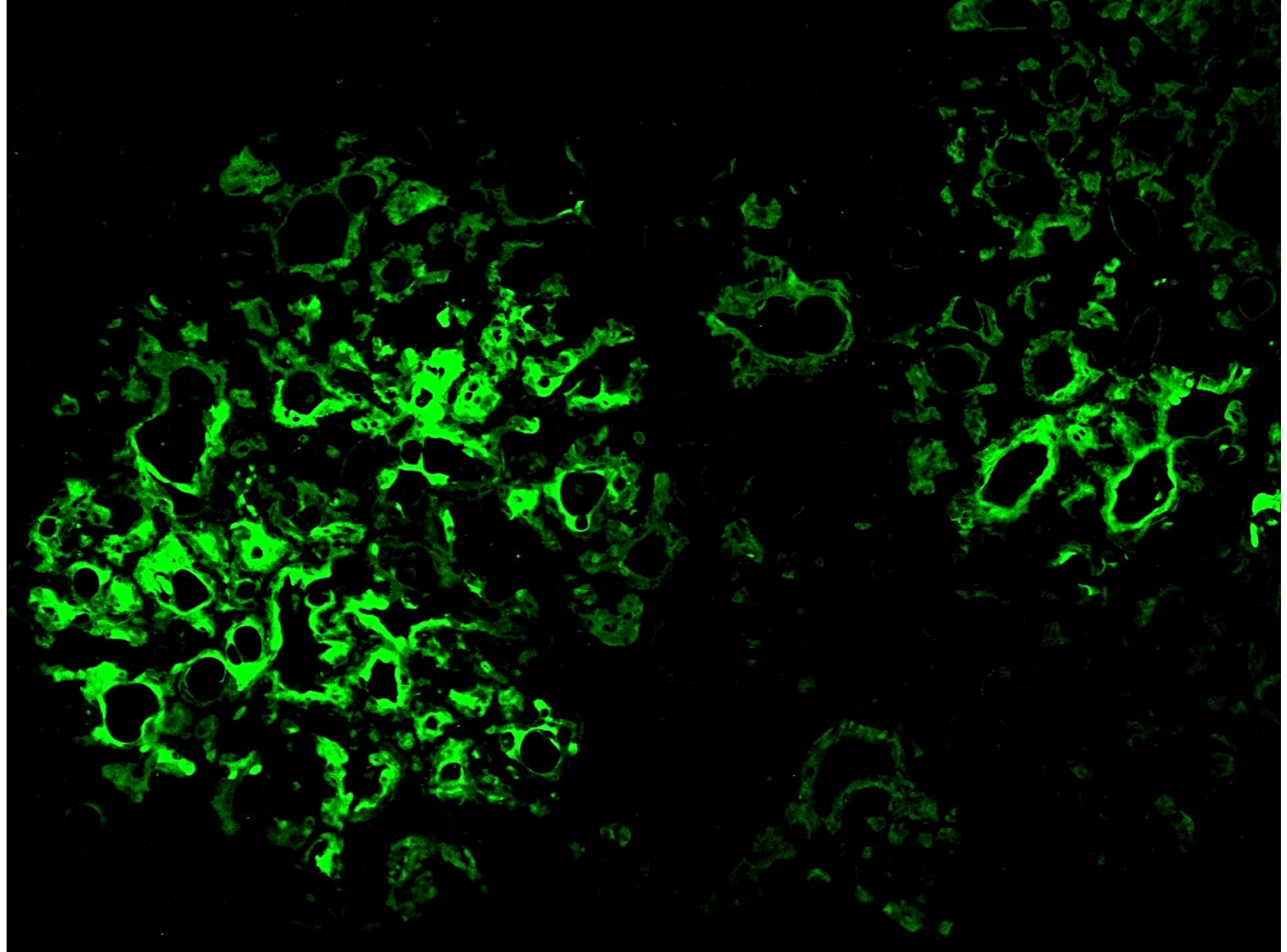

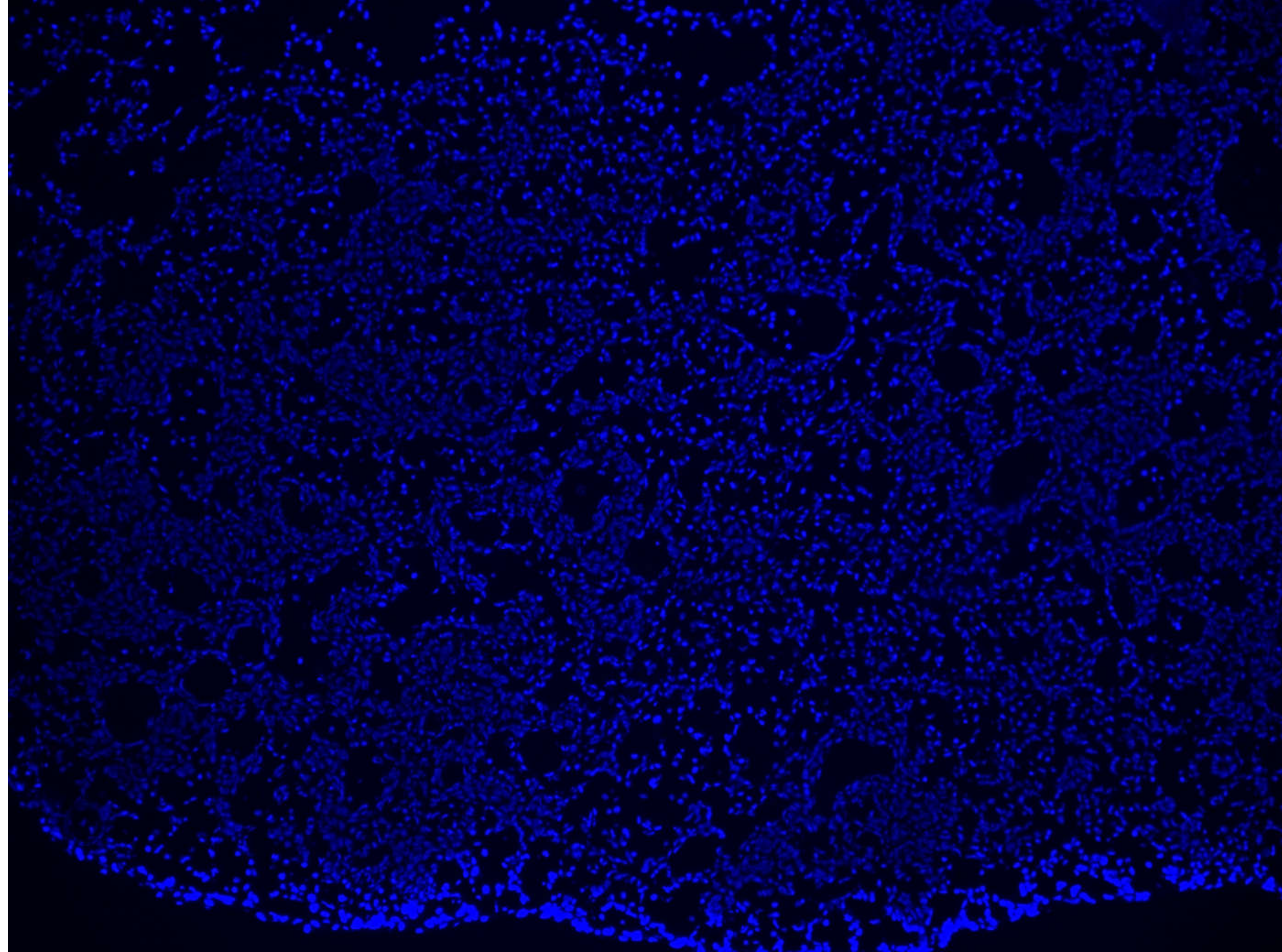

Supplement: Supplementary file 8 — Source Data for Figure 4 [file EMMM-12-e10233-s007.zip › Figure_4C_WT-lung_21days.pdf]

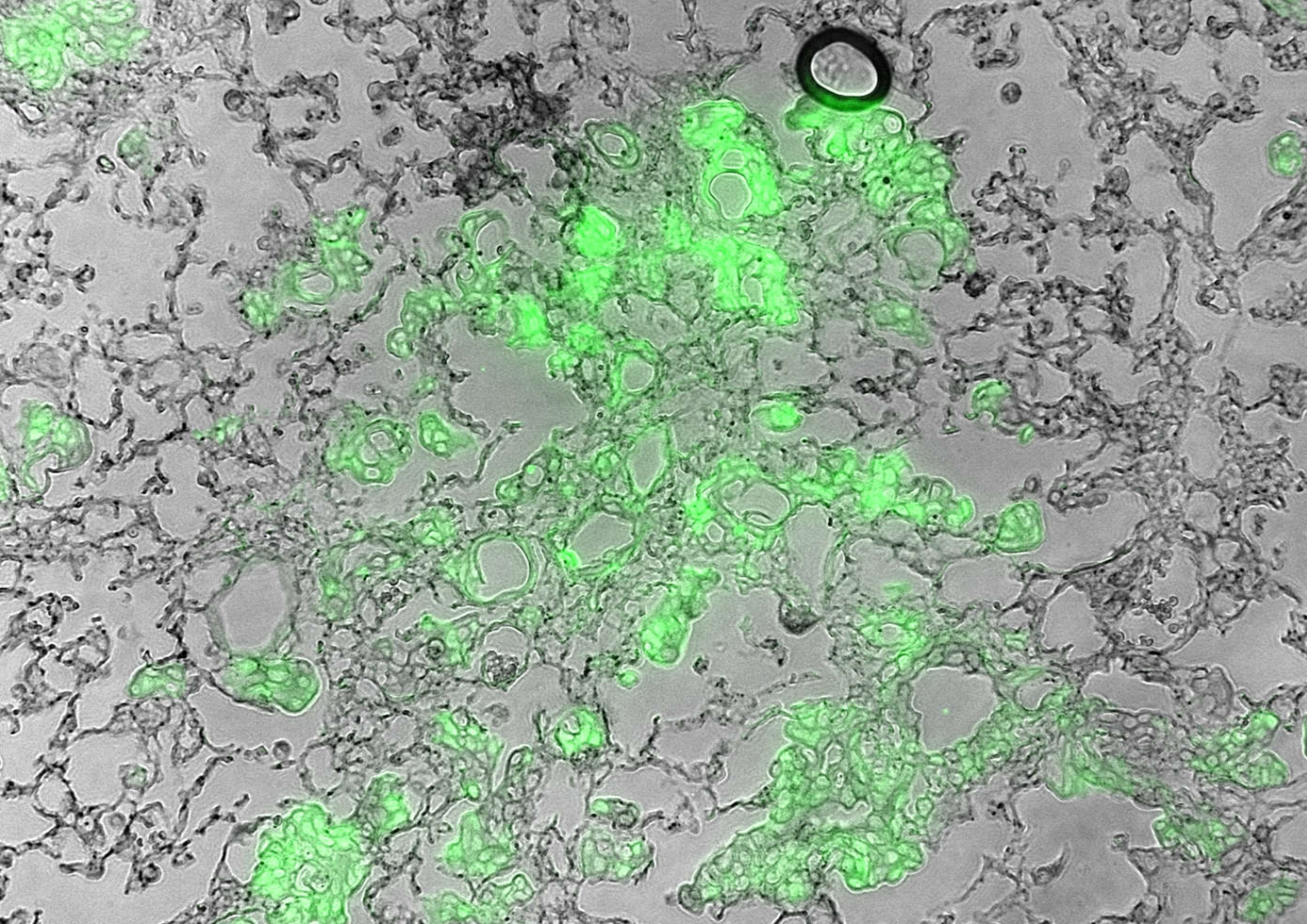

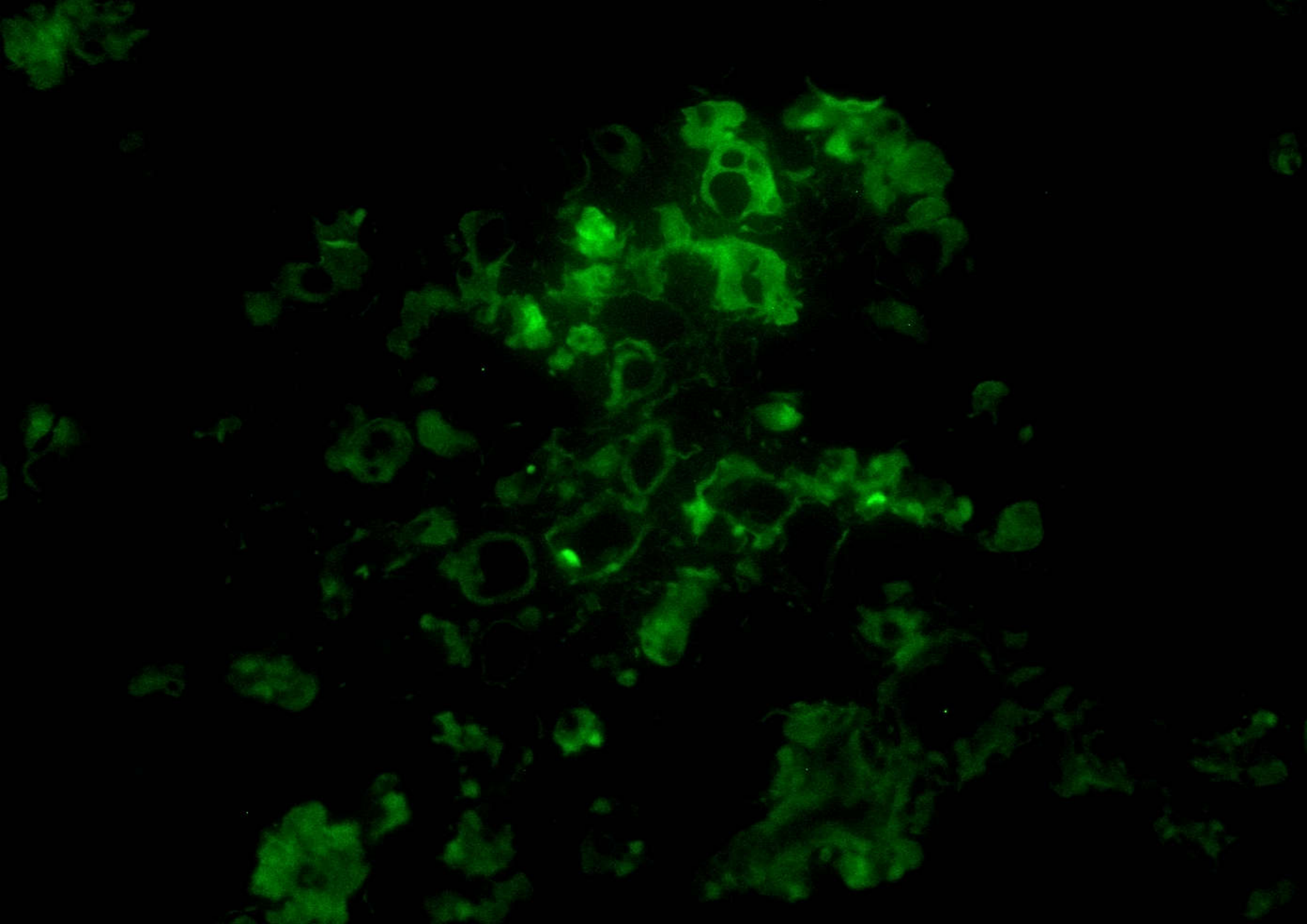



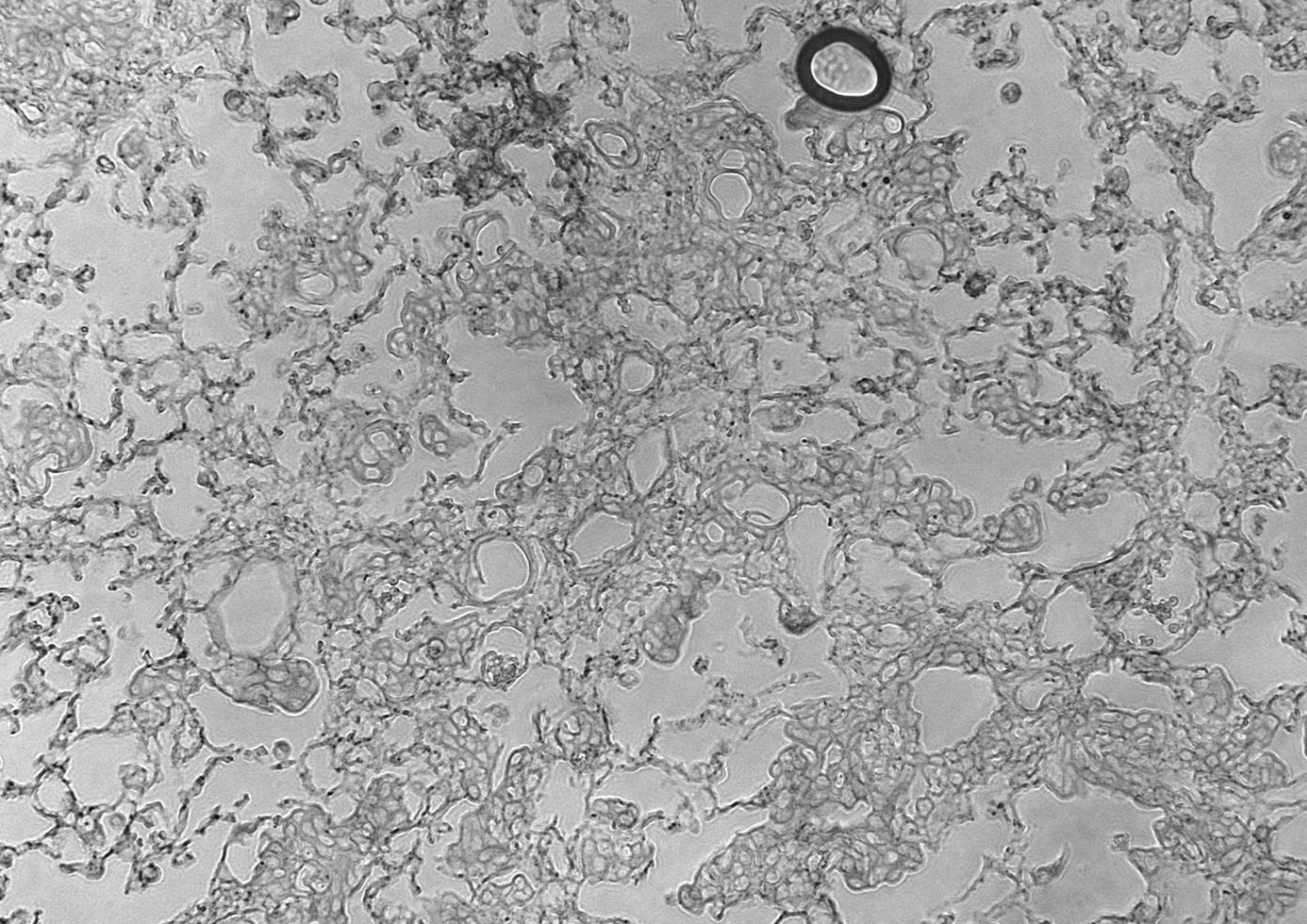

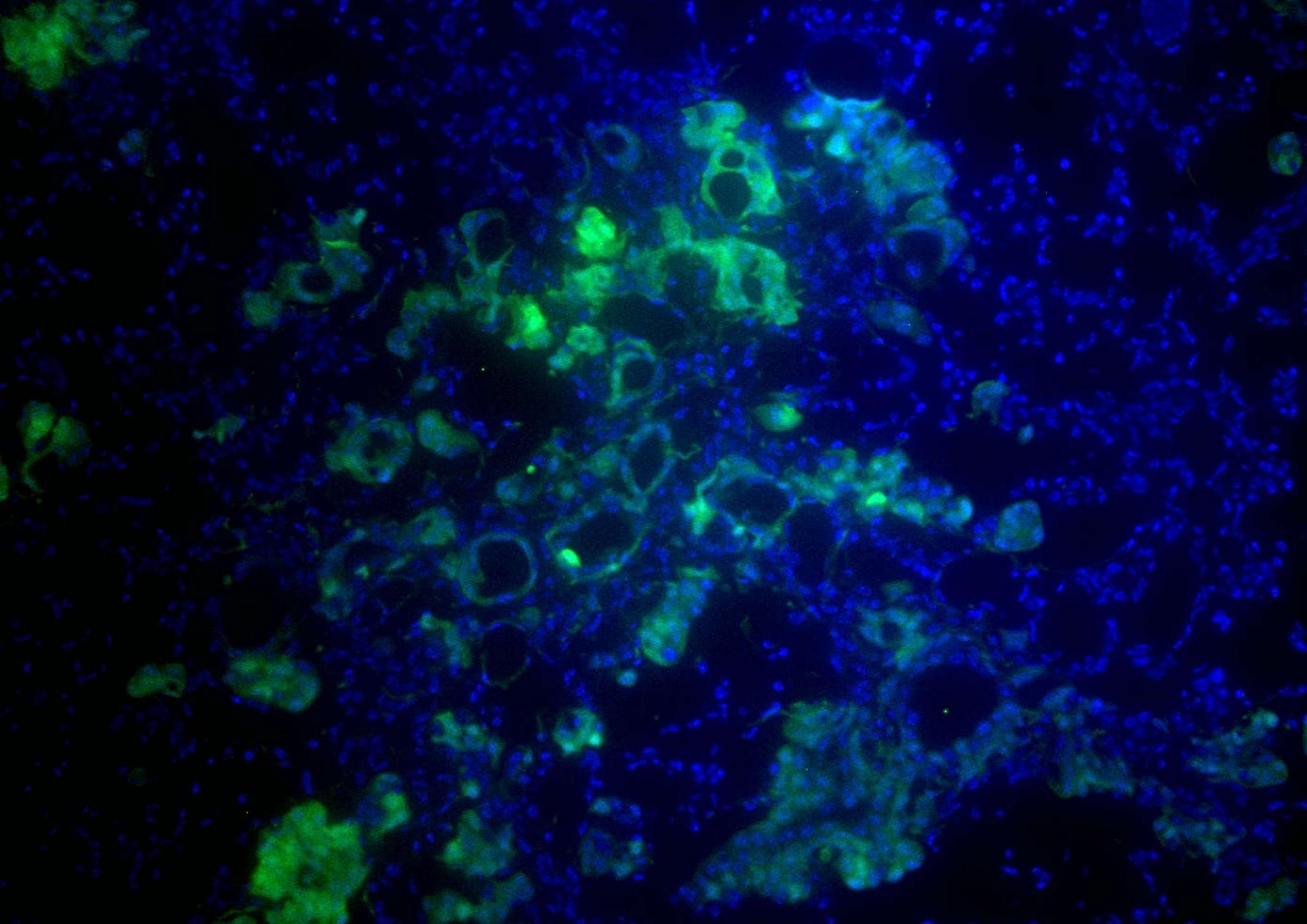

Supplement: Supplementary file 8 — Source Data for Figure 4 [file EMMM-12-e10233-s007.zip › Figure_4C_WT-lung_28days.pdf]

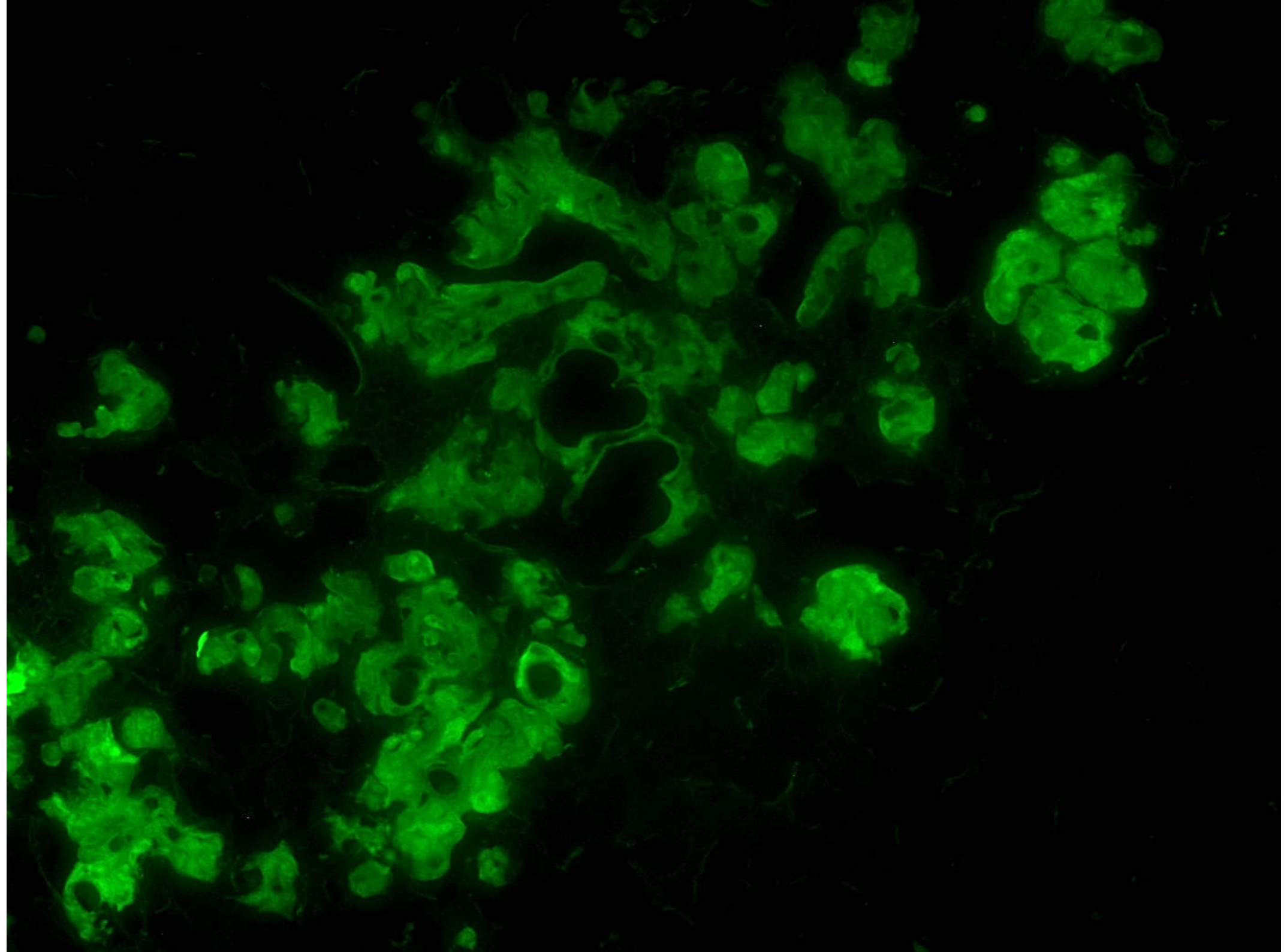

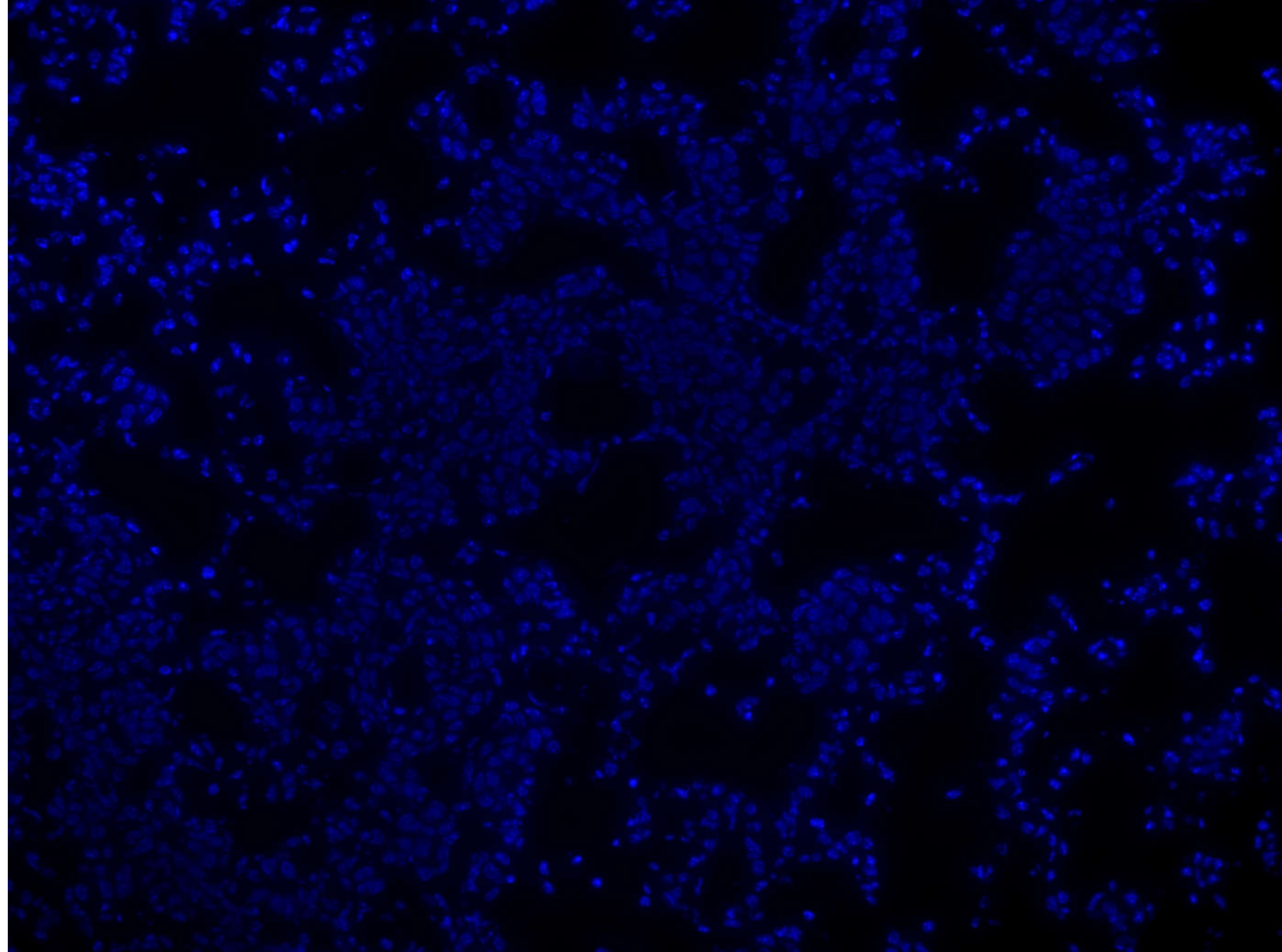

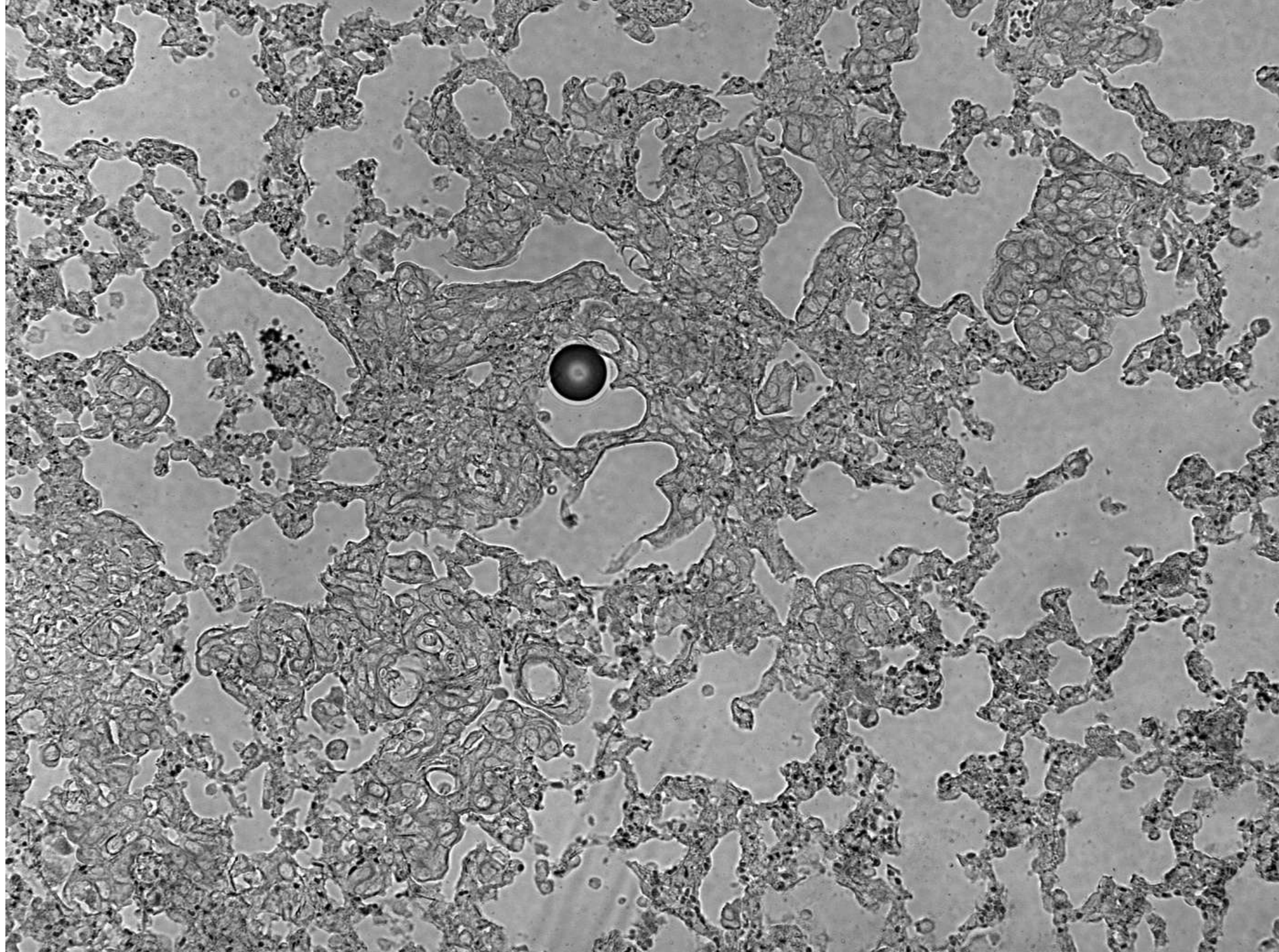

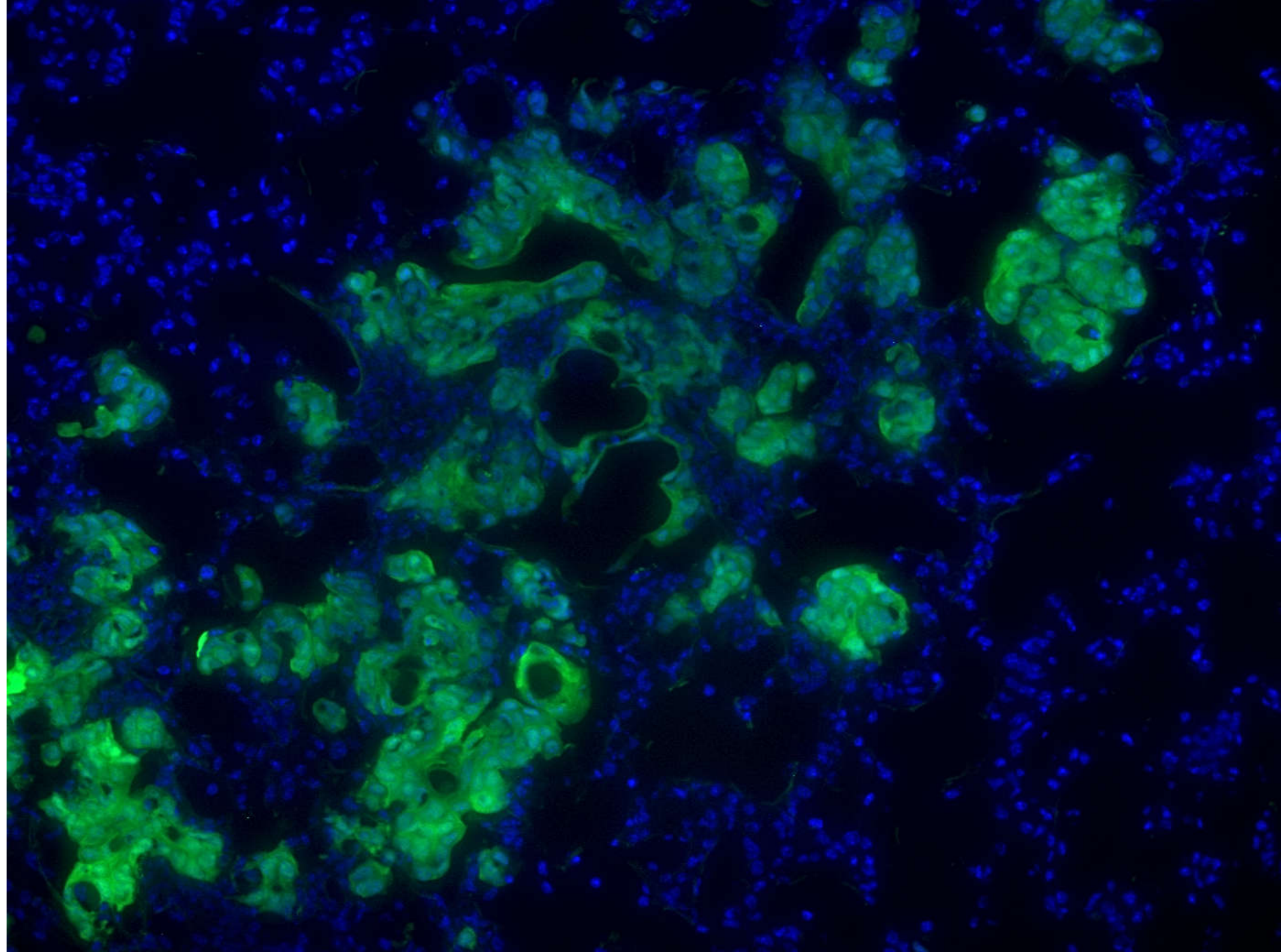

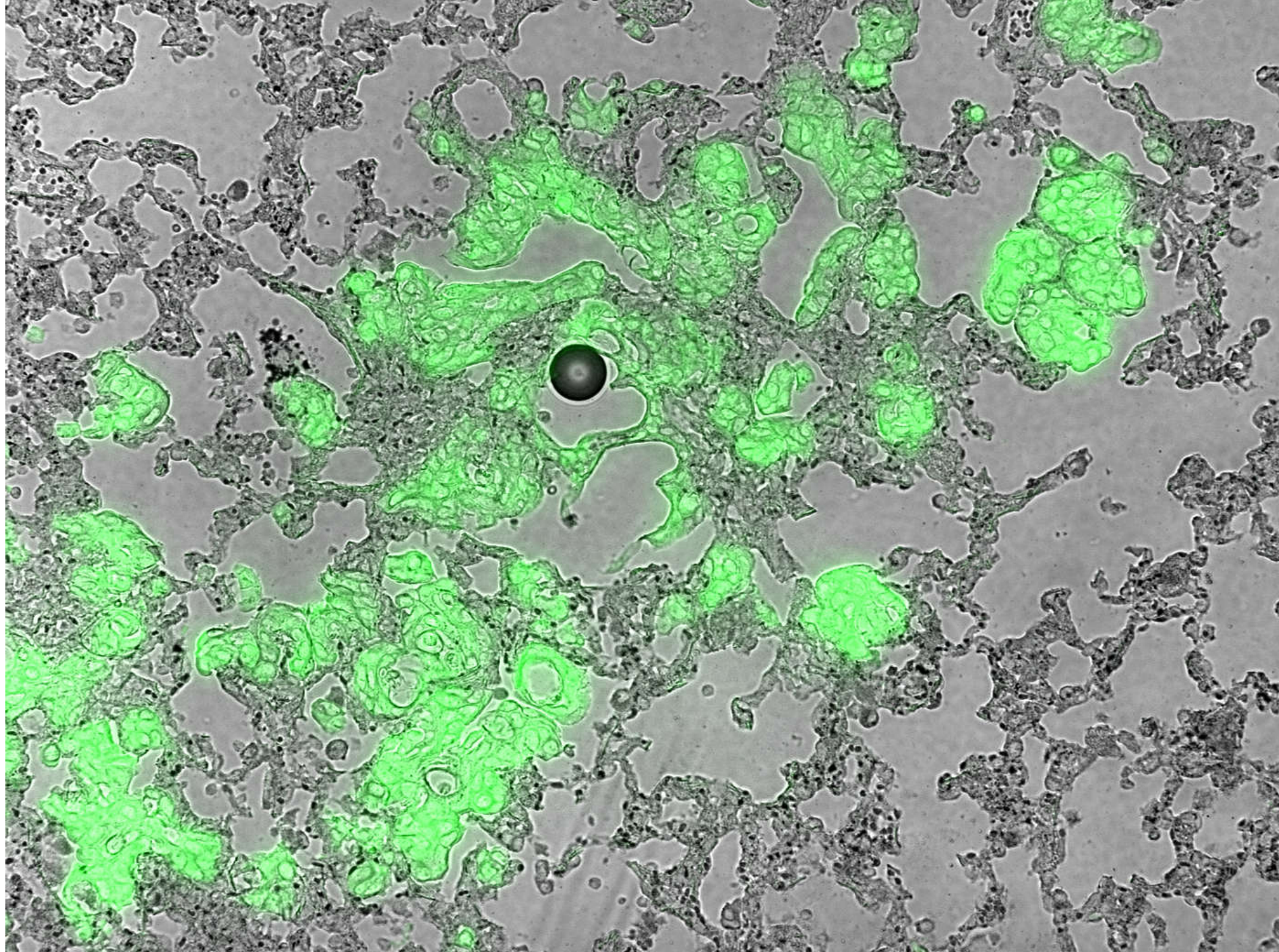

Supplement: Supplementary file 8 — Source Data for Figure 4 [file EMMM-12-e10233-s007.zip › Figure_4C_WT-lung_7days.pdf]

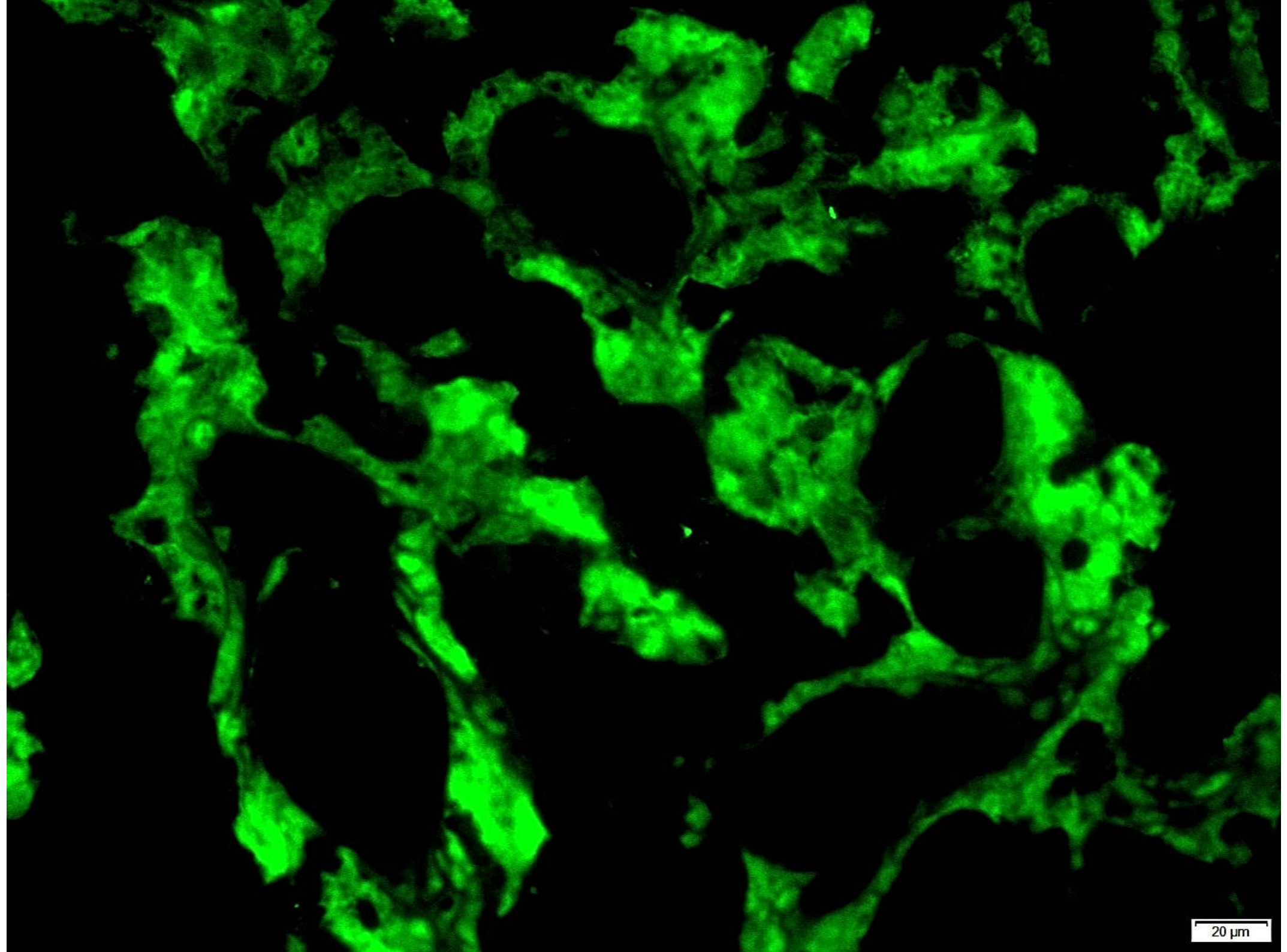

20  $\mu\text{m}$

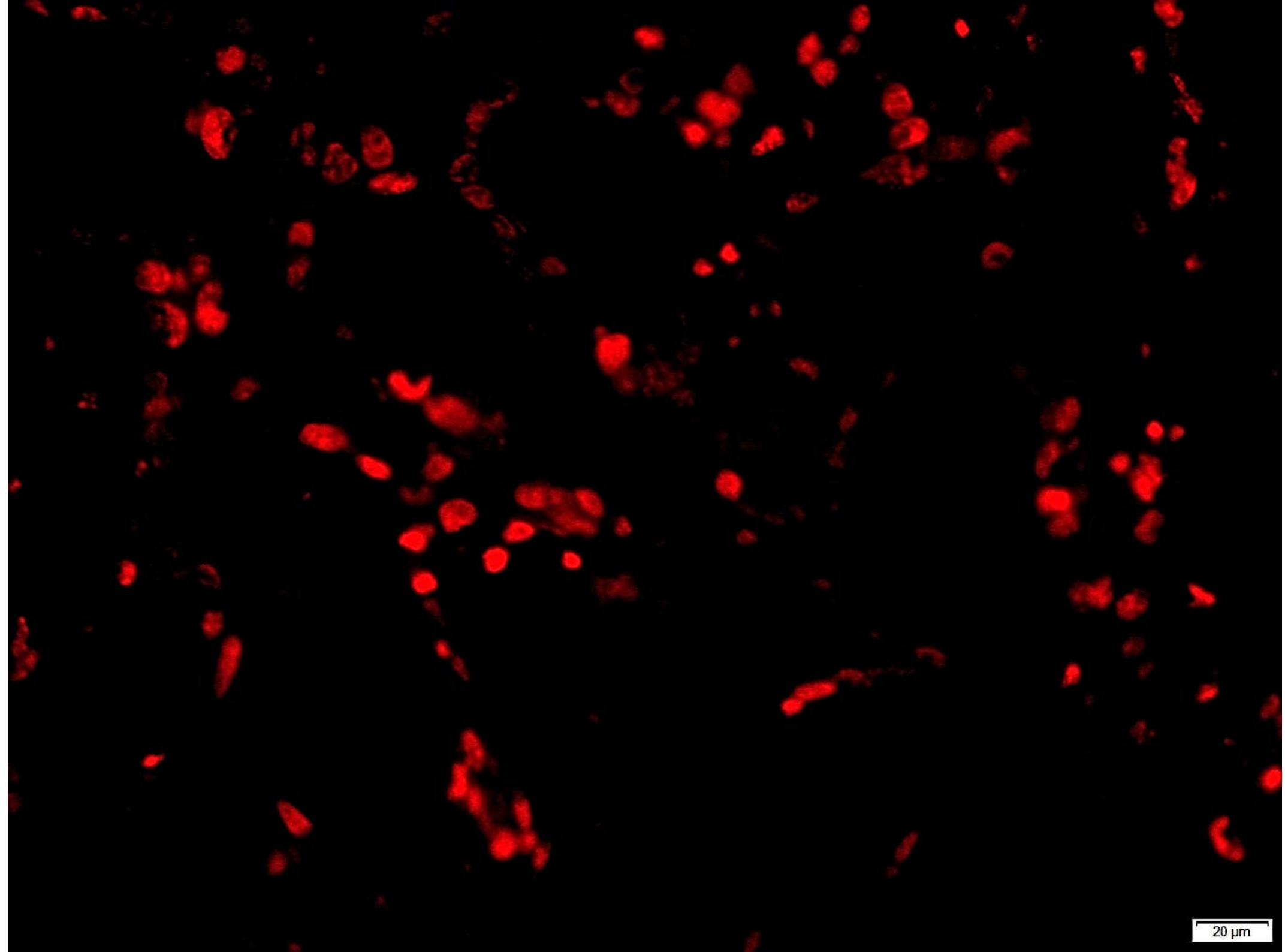

20  $\mu\text{m}$

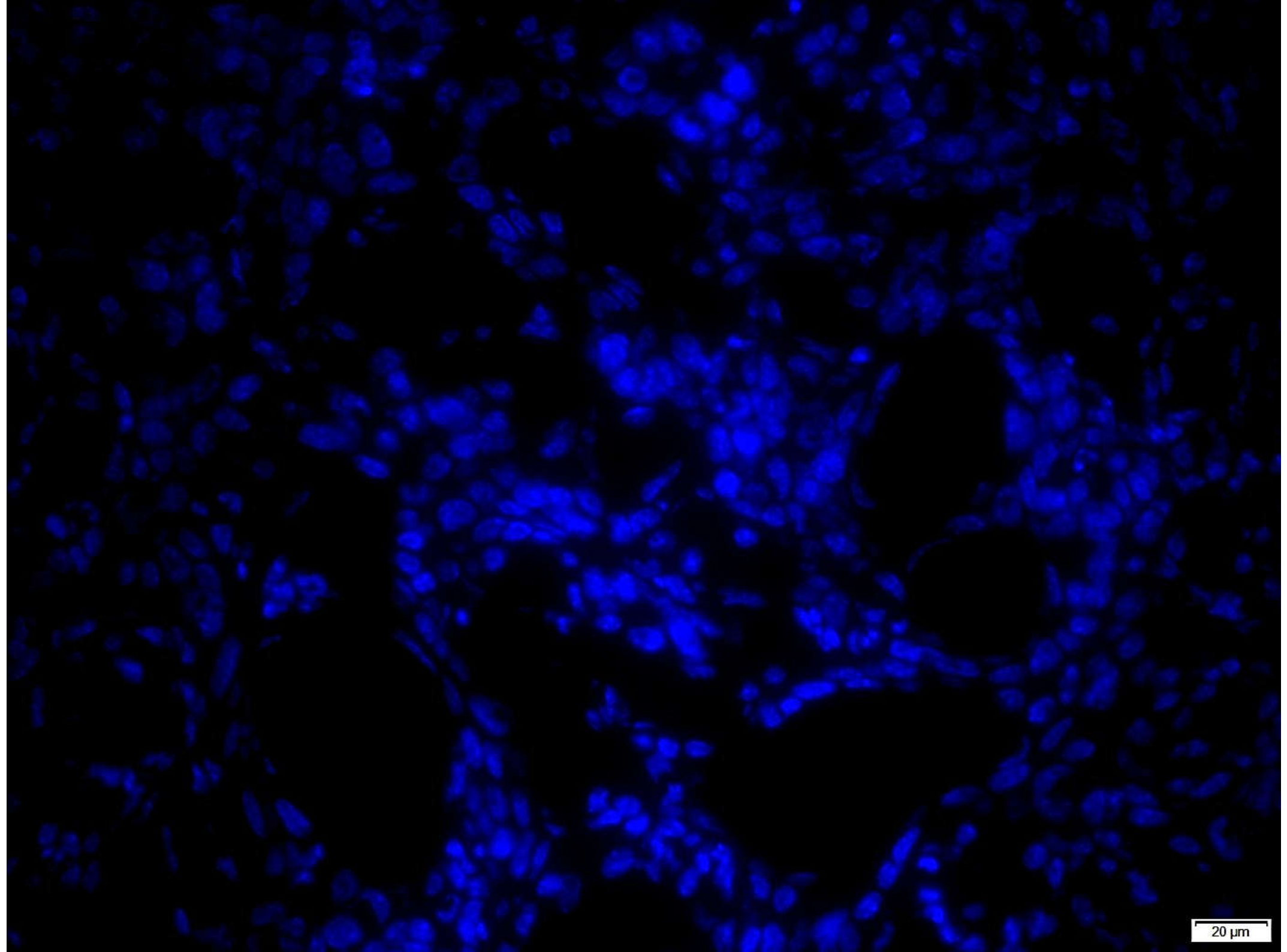

20 μm

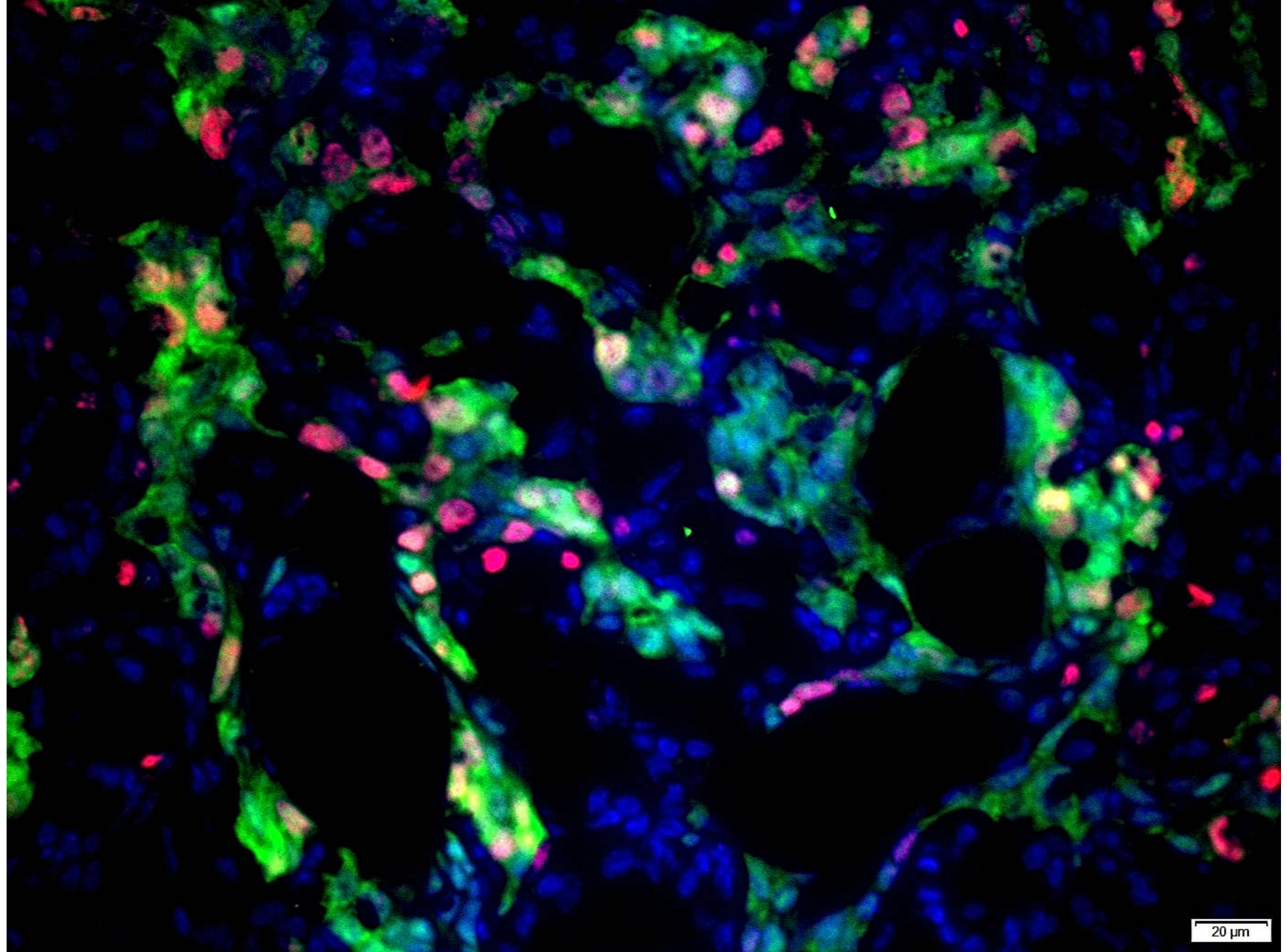

20  $\mu\text{m}$

Supplement: Supplementary file 8 — Source Data for Figure 4 [file EMMM-12-e10233-s007.zip › Figure_4D_LL-37-lung_.pdf]

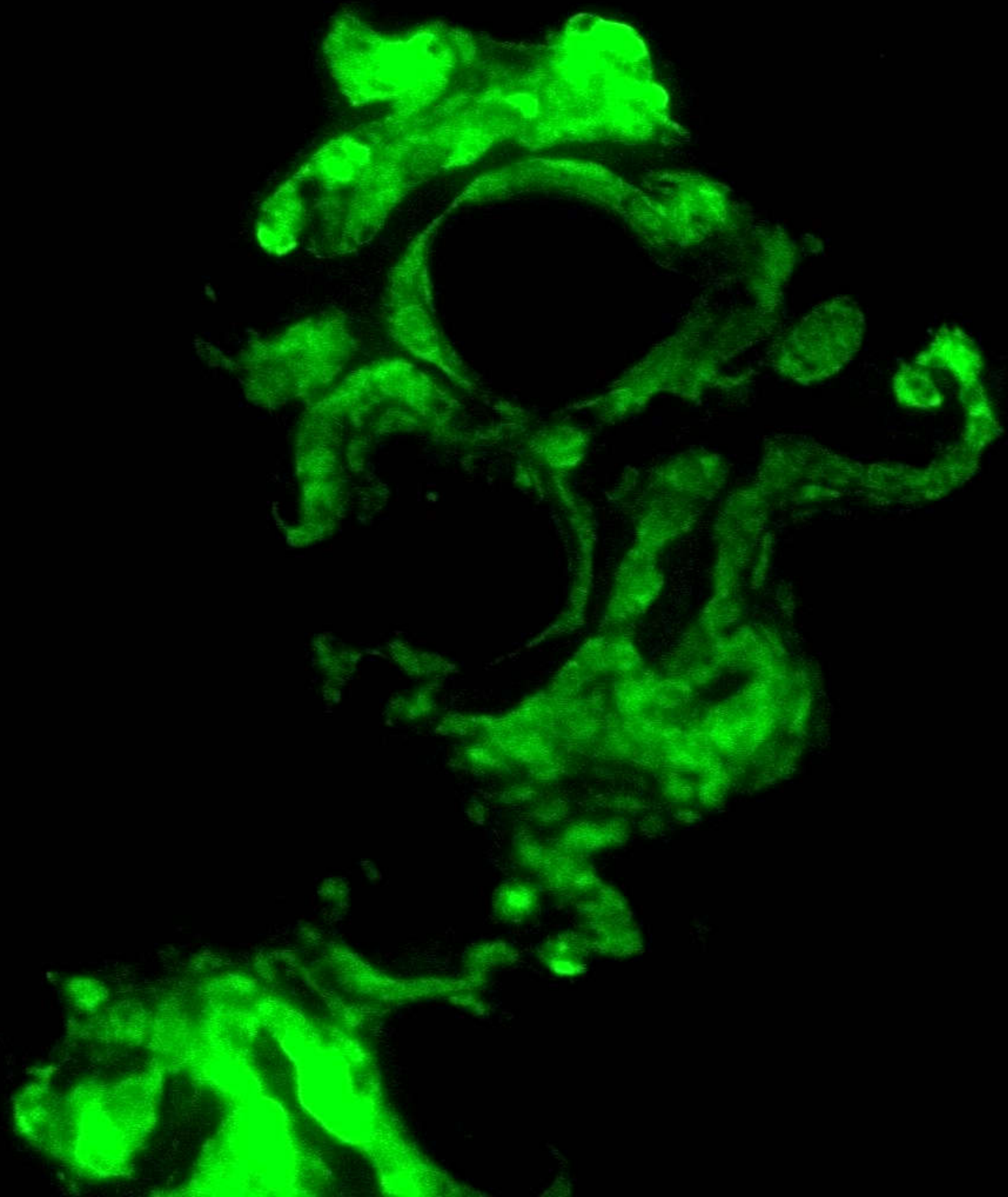

20  $\mu\text{m}$

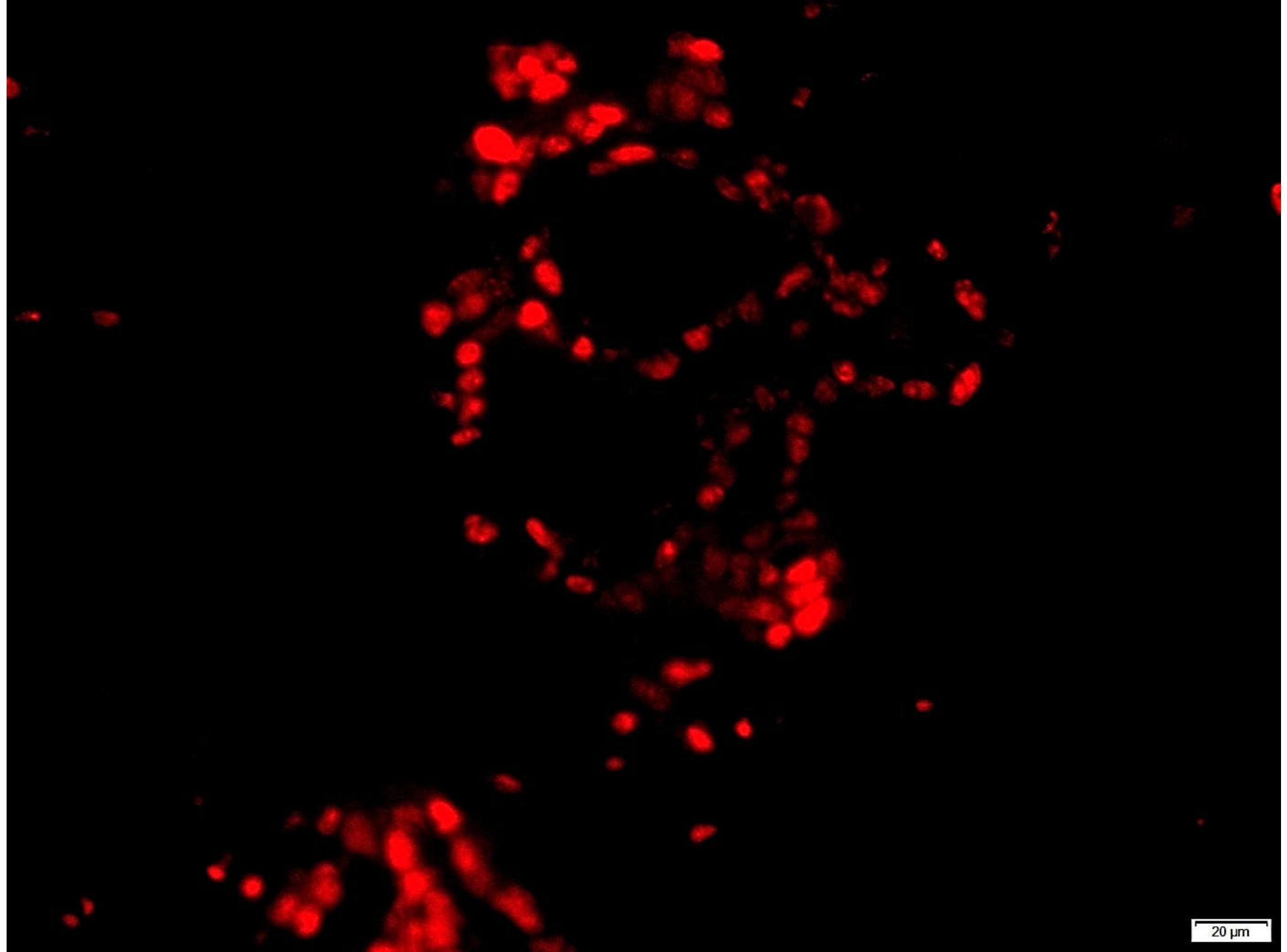

20  $\mu\text{m}$

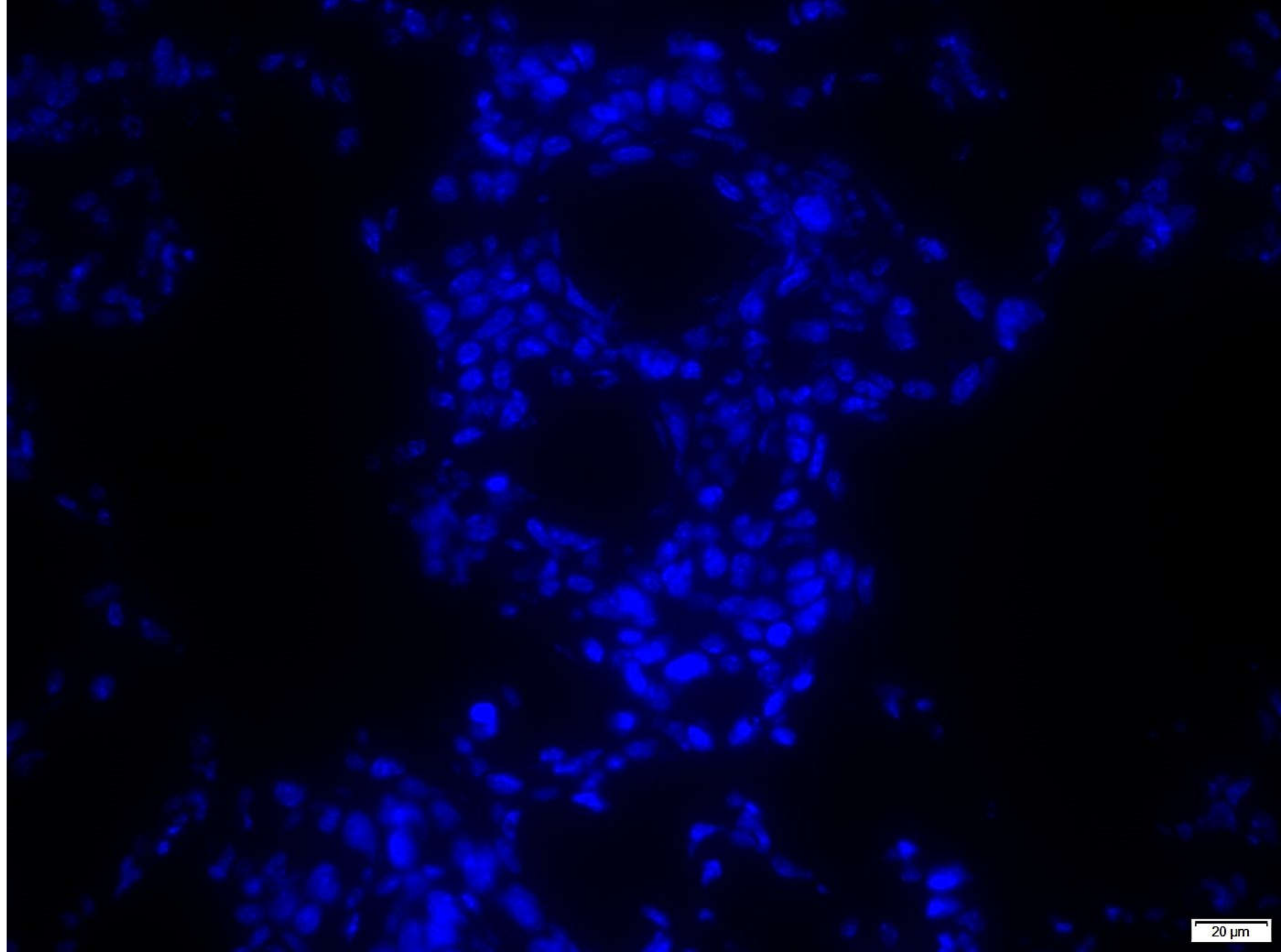

20 μm

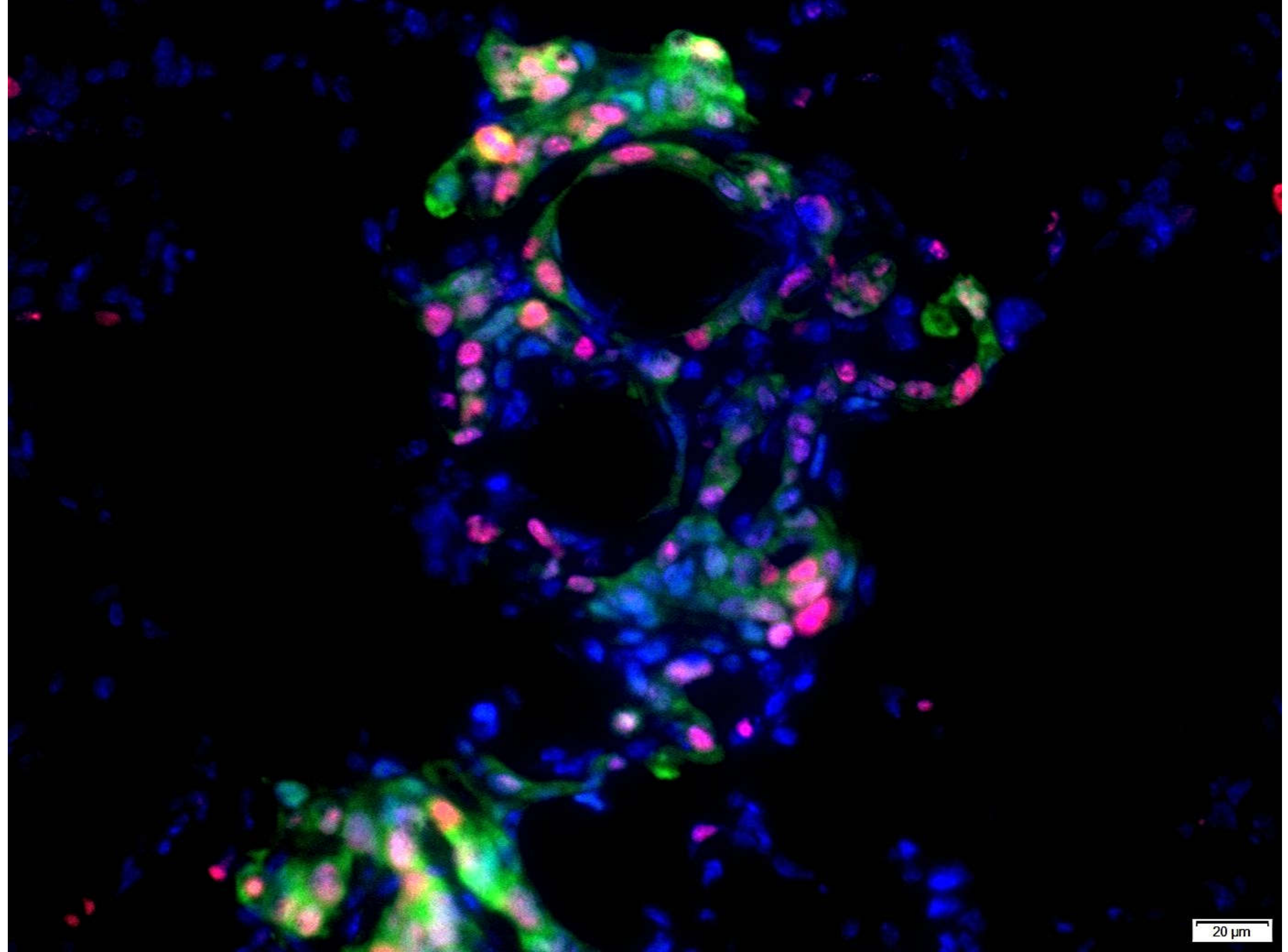

20  $\mu\text{m}$

Supplement: Supplementary file 8 — Source Data for Figure 4 [file EMMM-12-e10233-s007.zip › Figure_4D_WT-lung_.pdf]

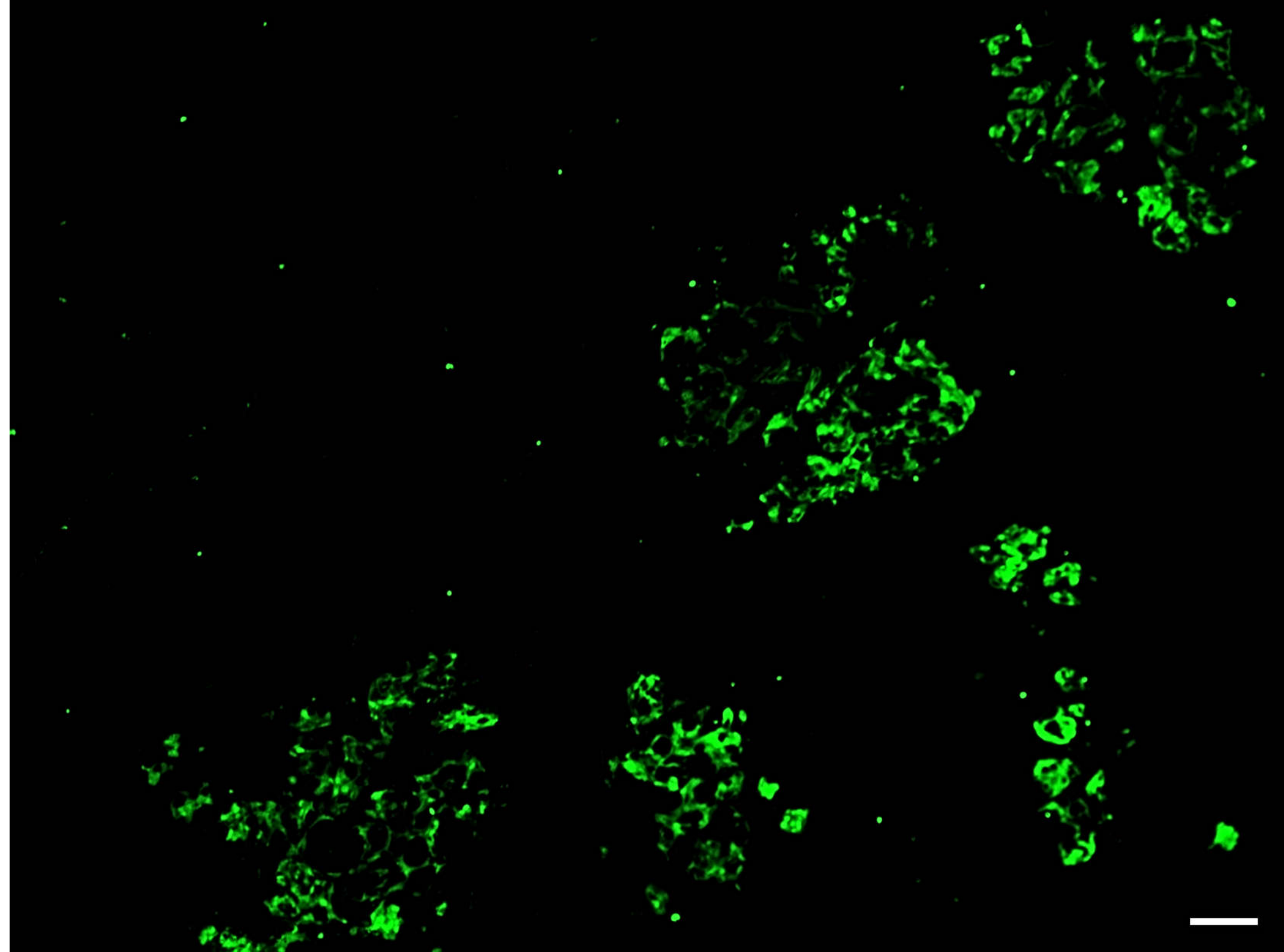

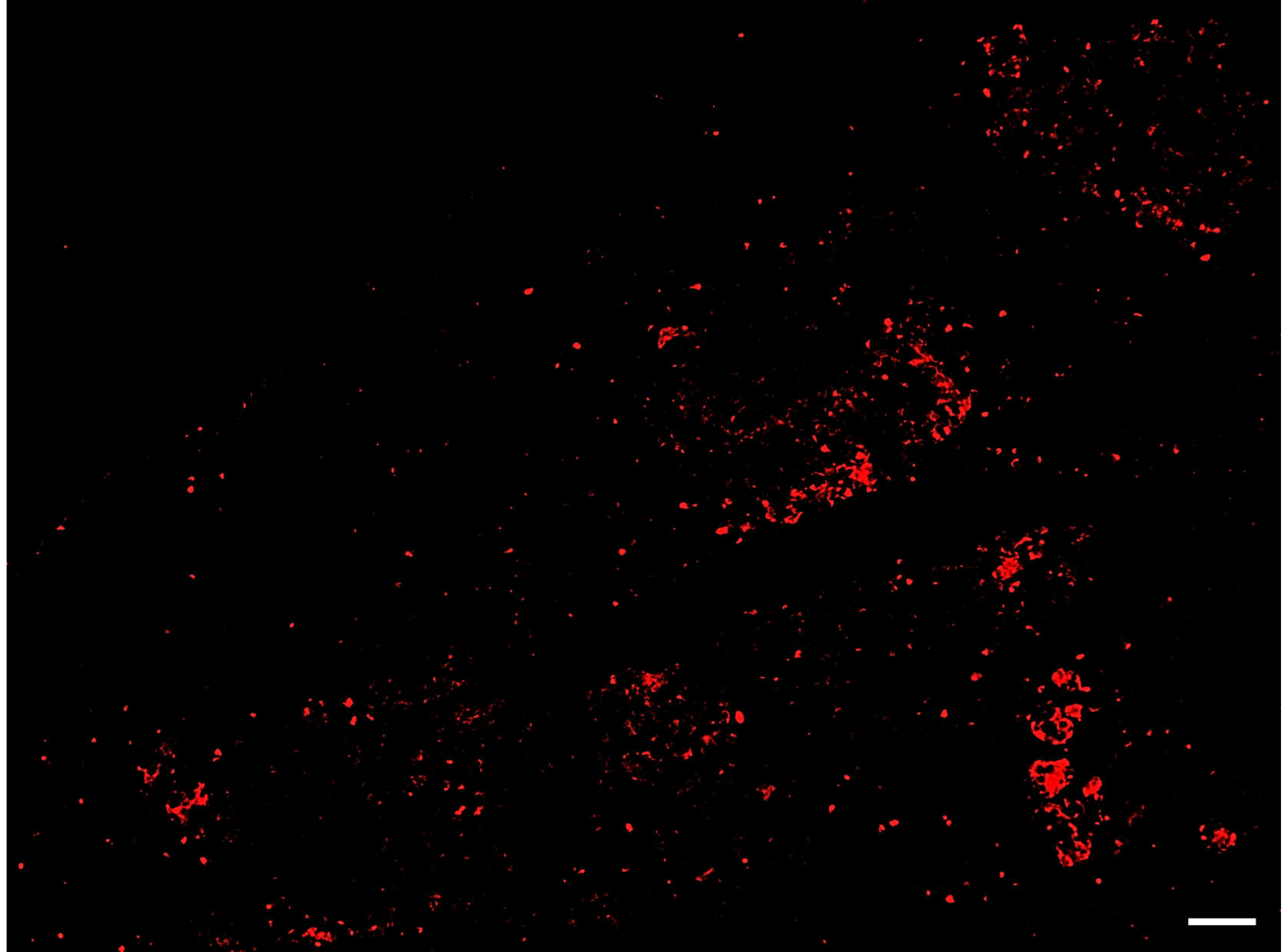

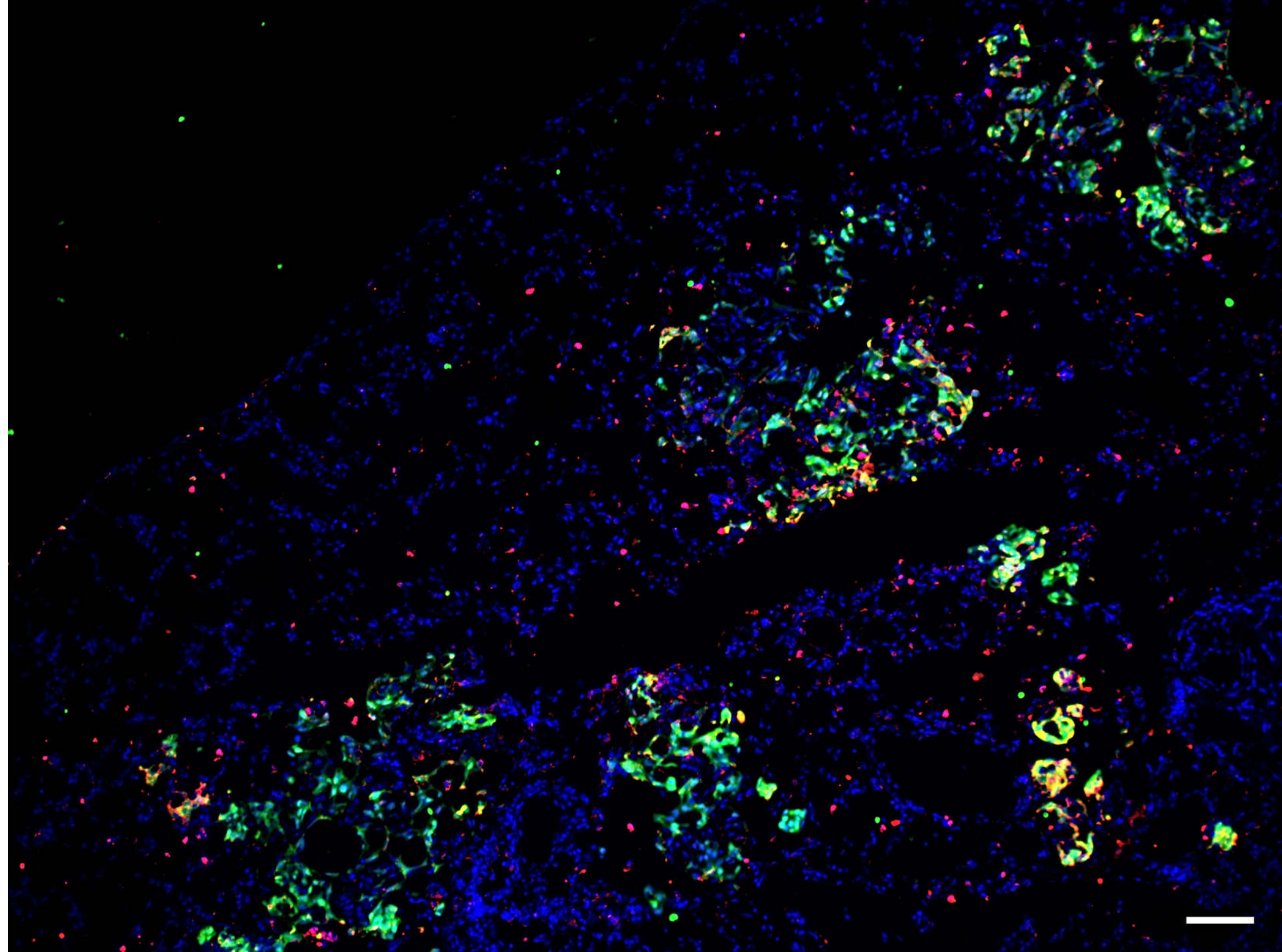

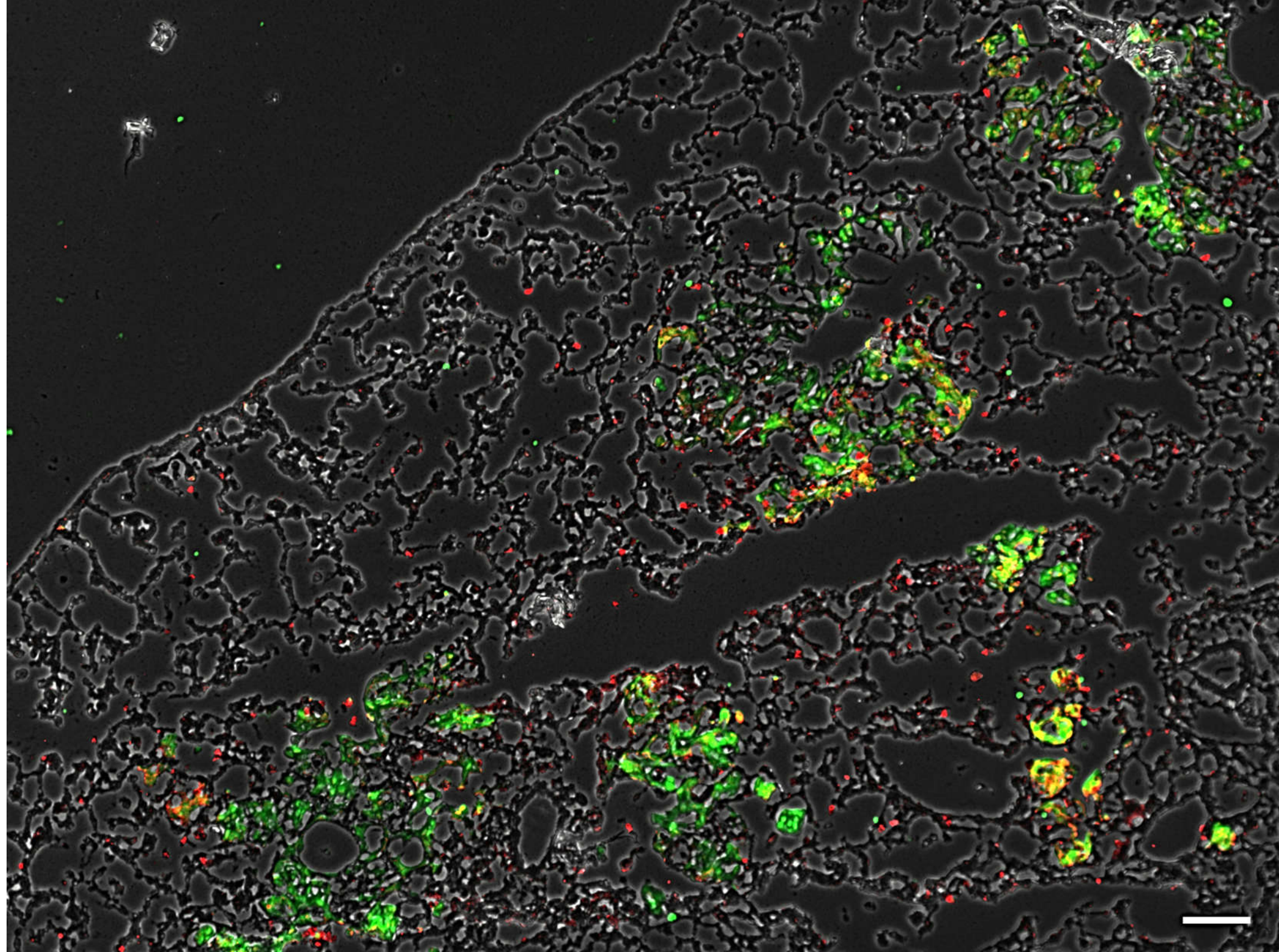

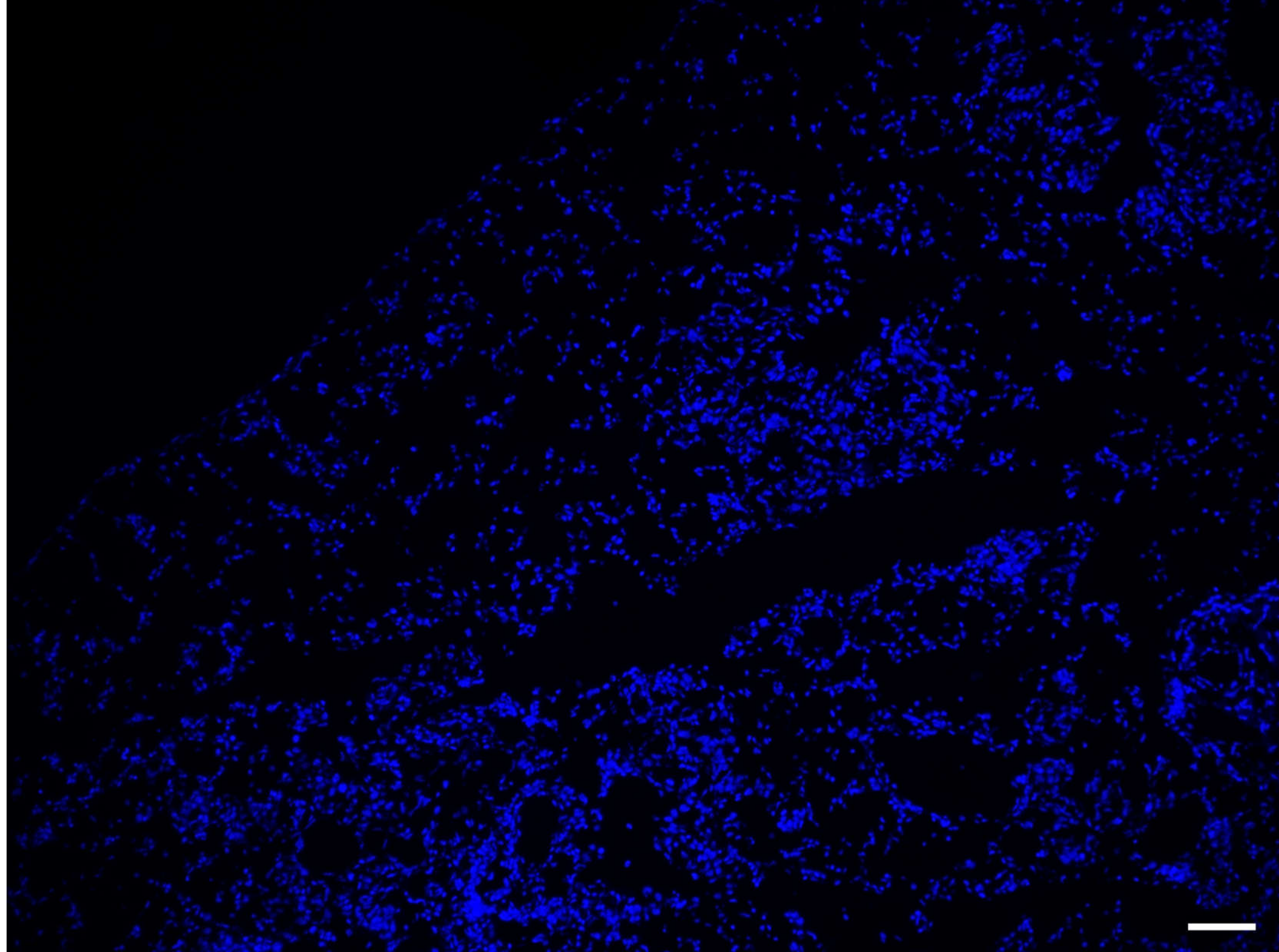

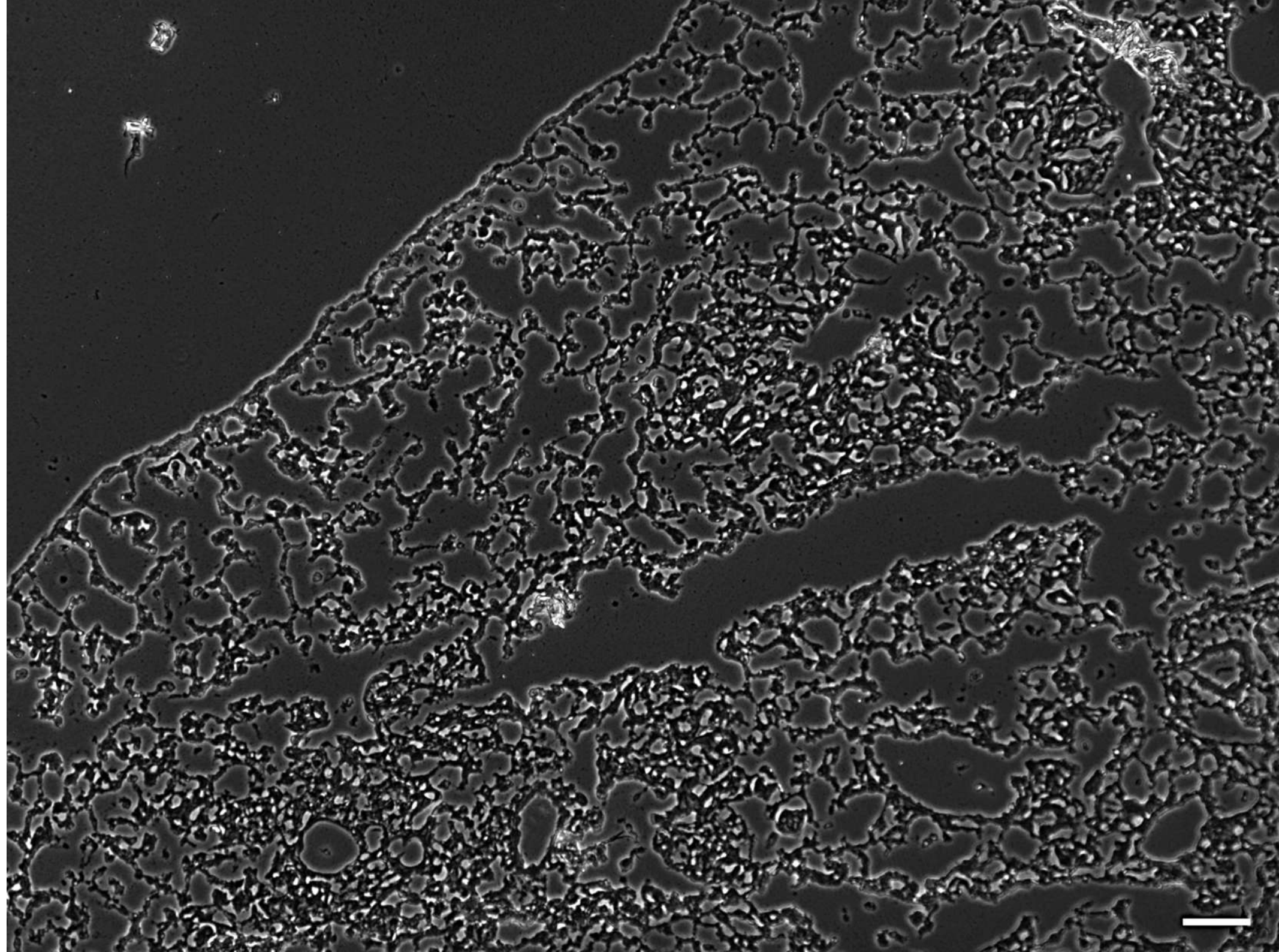

Supplement: Supplementary file 9 — Source Data for Figure 5 [file EMMM-12-e10233-s008.zip › Figure_5A_LL-37-lung_.pdf]

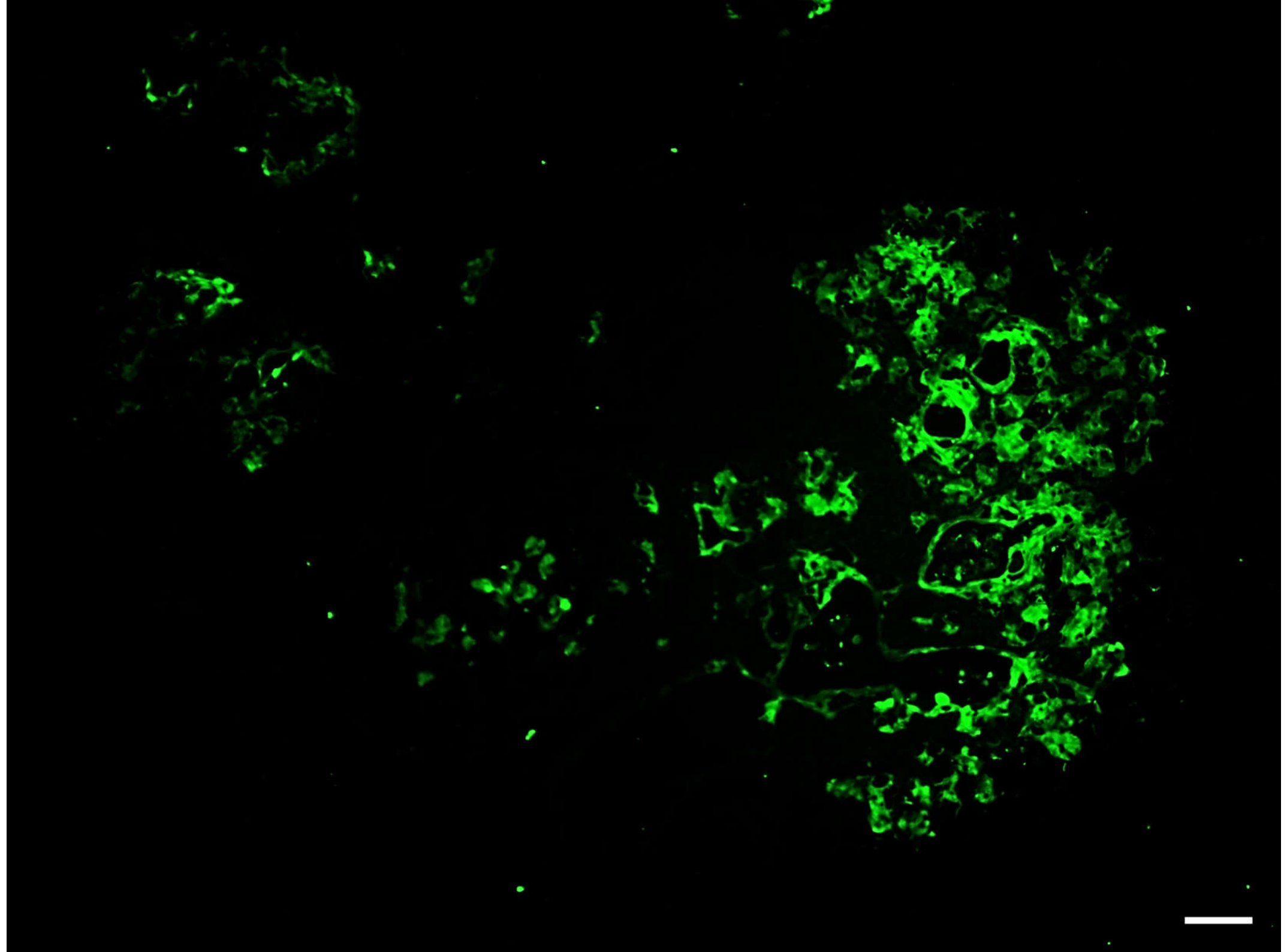

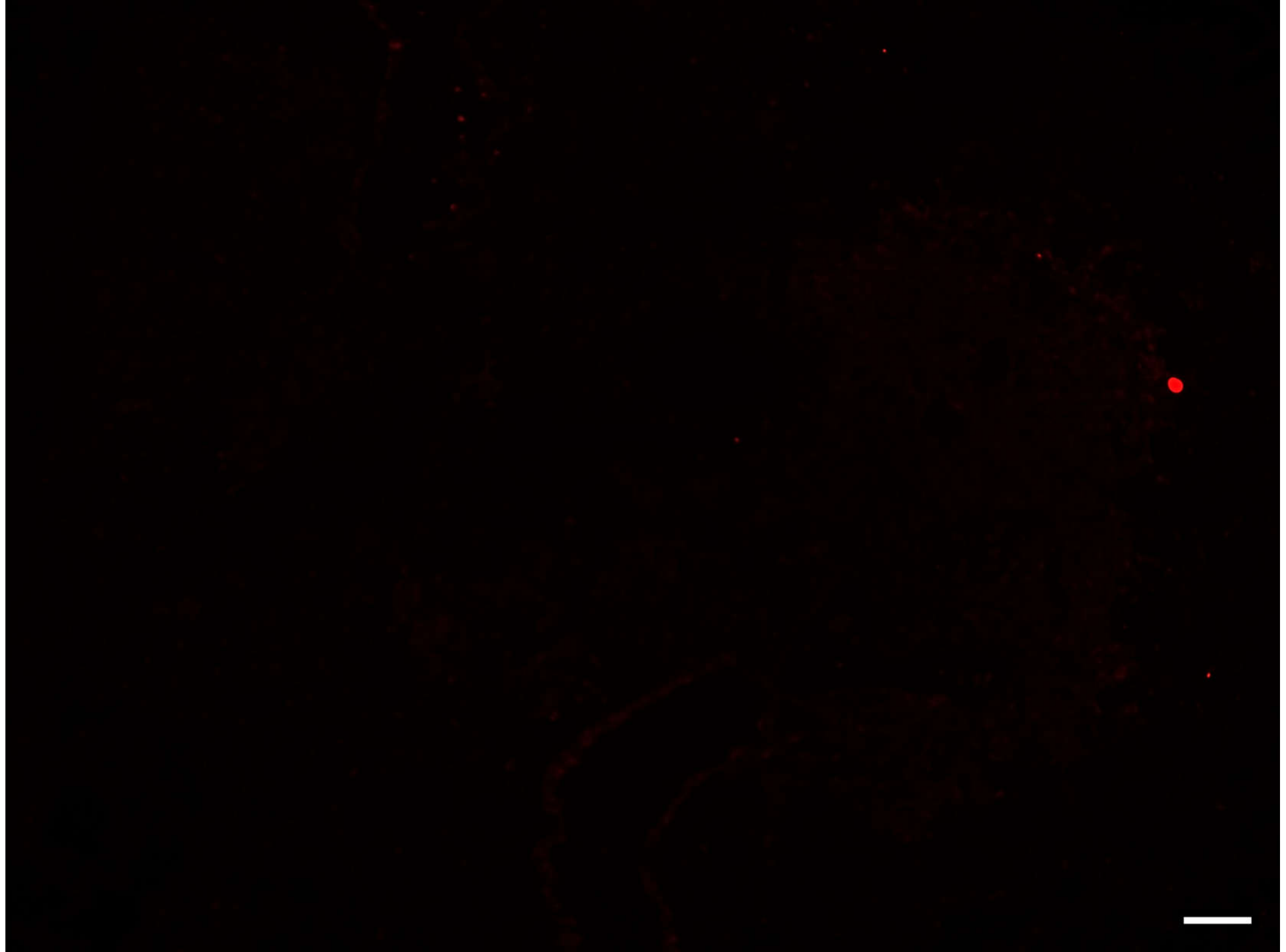

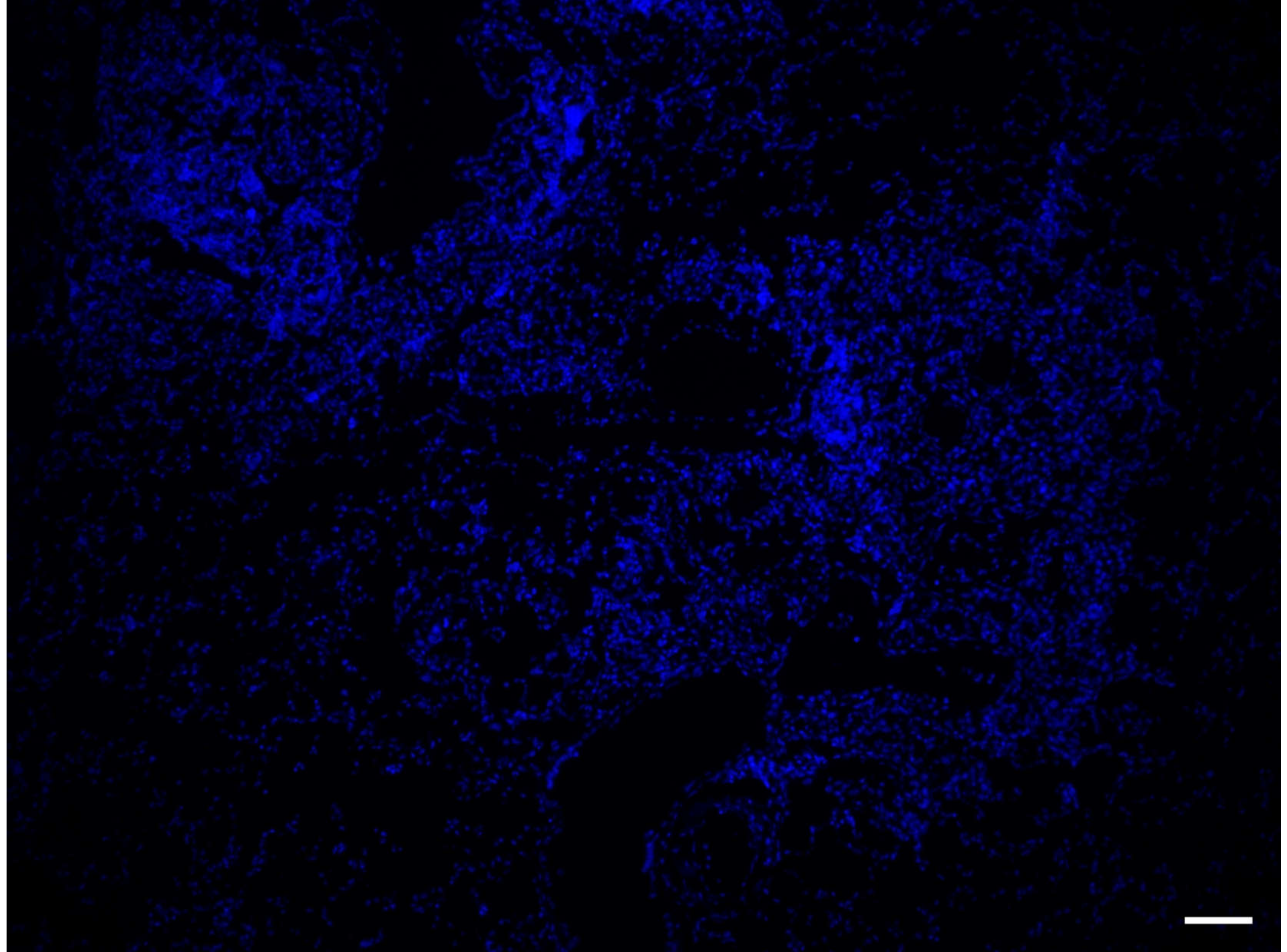

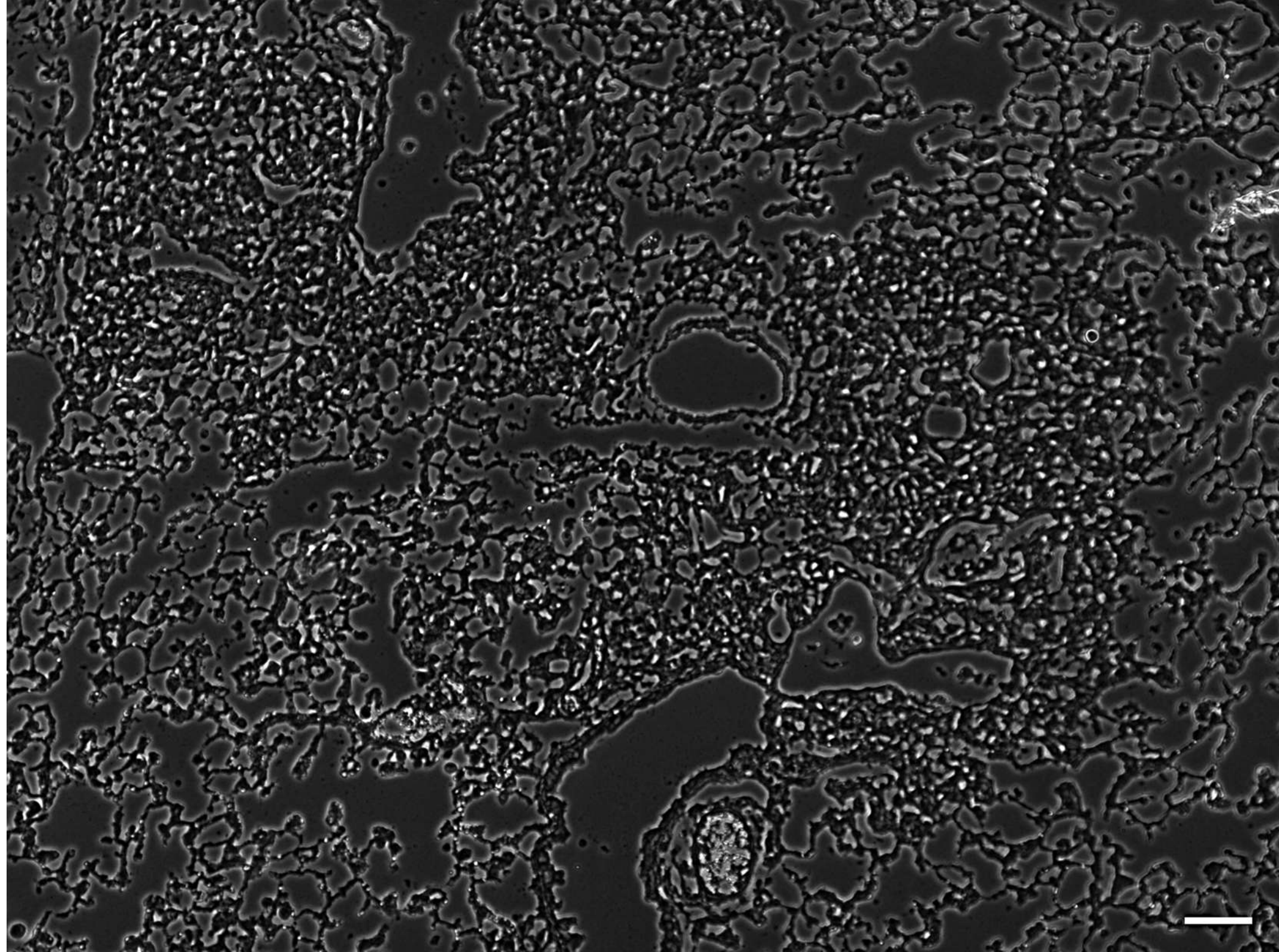

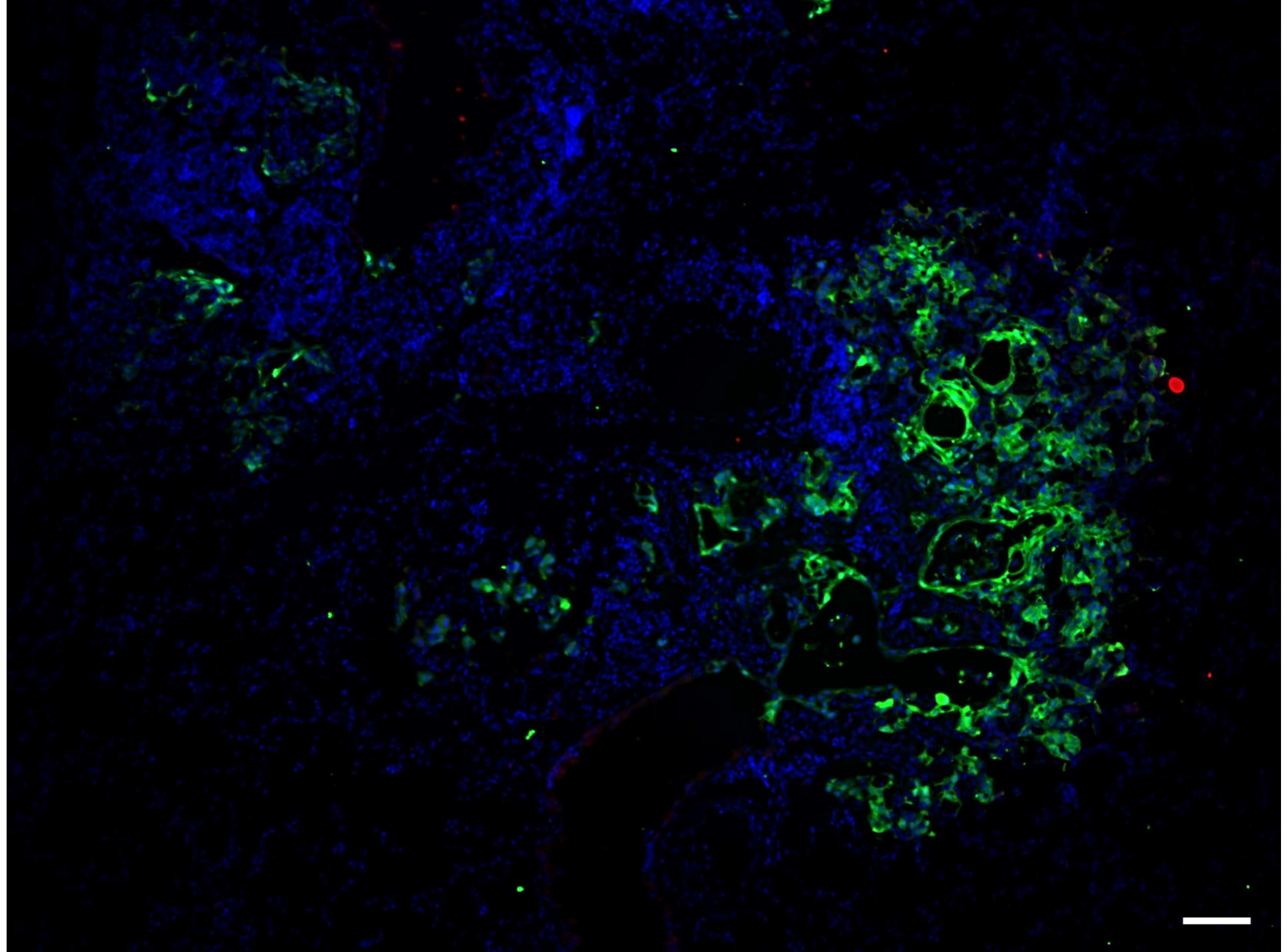

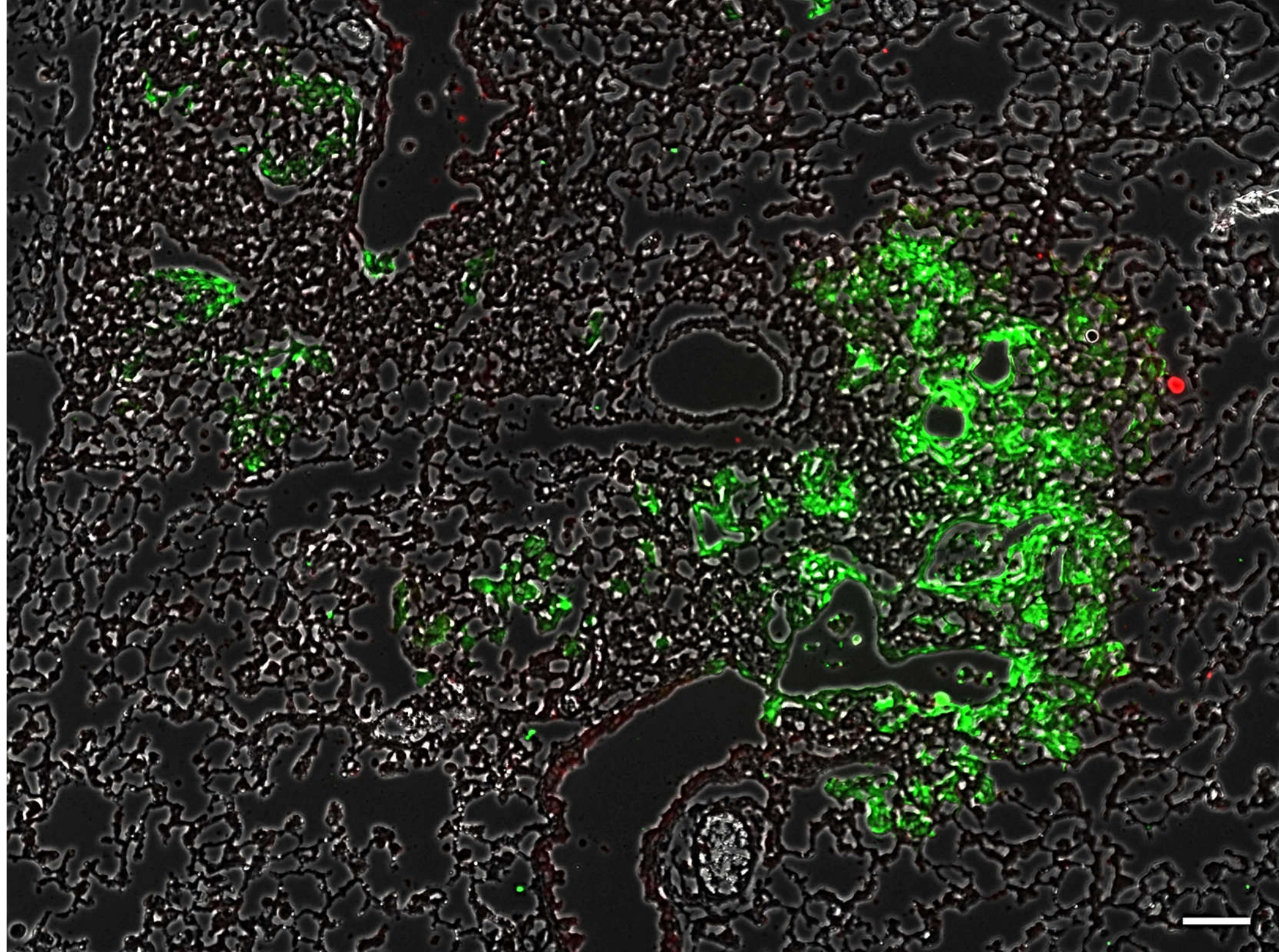

Supplement: Supplementary file 9 — Source Data for Figure 5 [file EMMM-12-e10233-s008.zip › Figure_5A_WT-lung_.pdf]

Group2

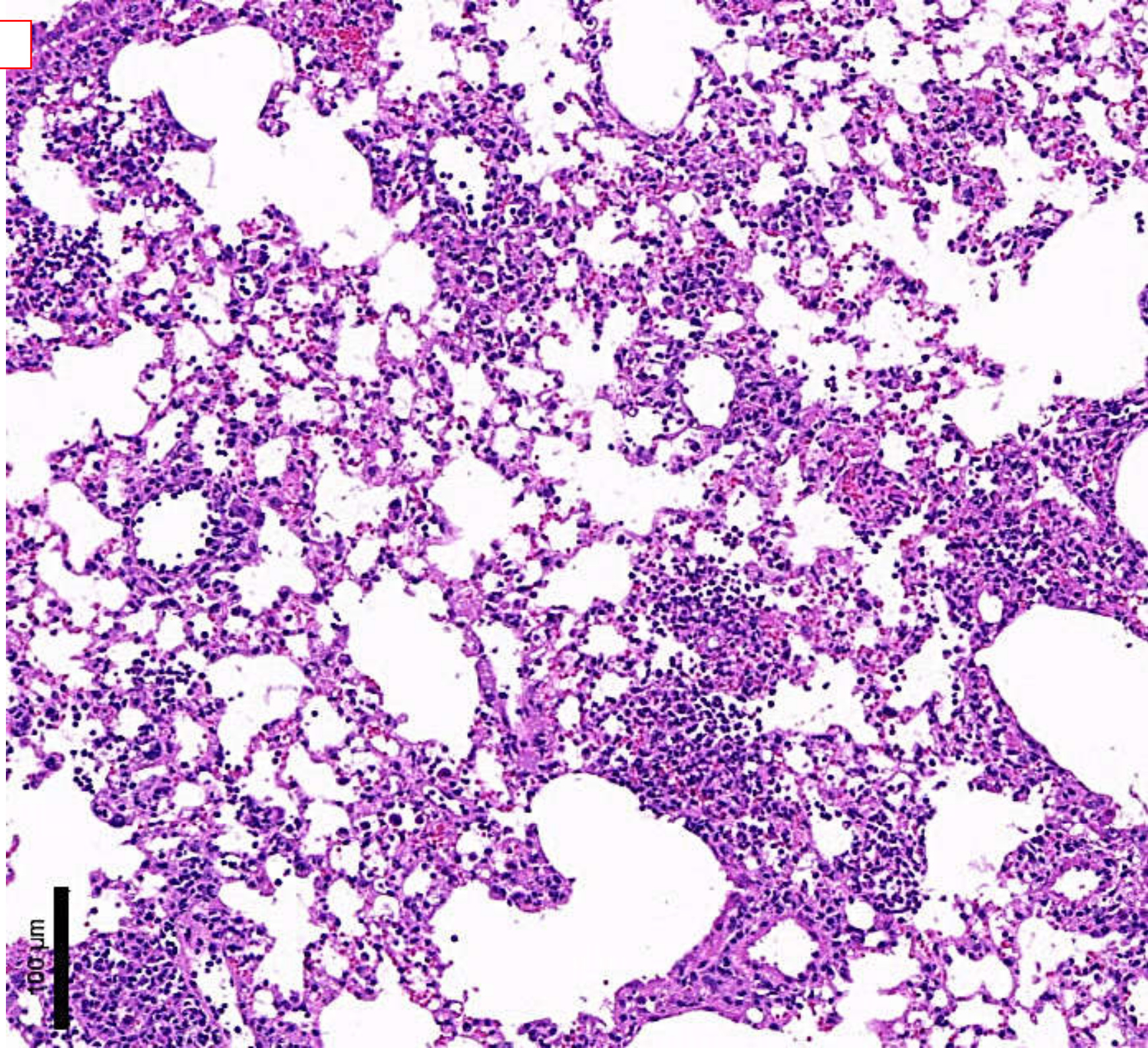

Group1

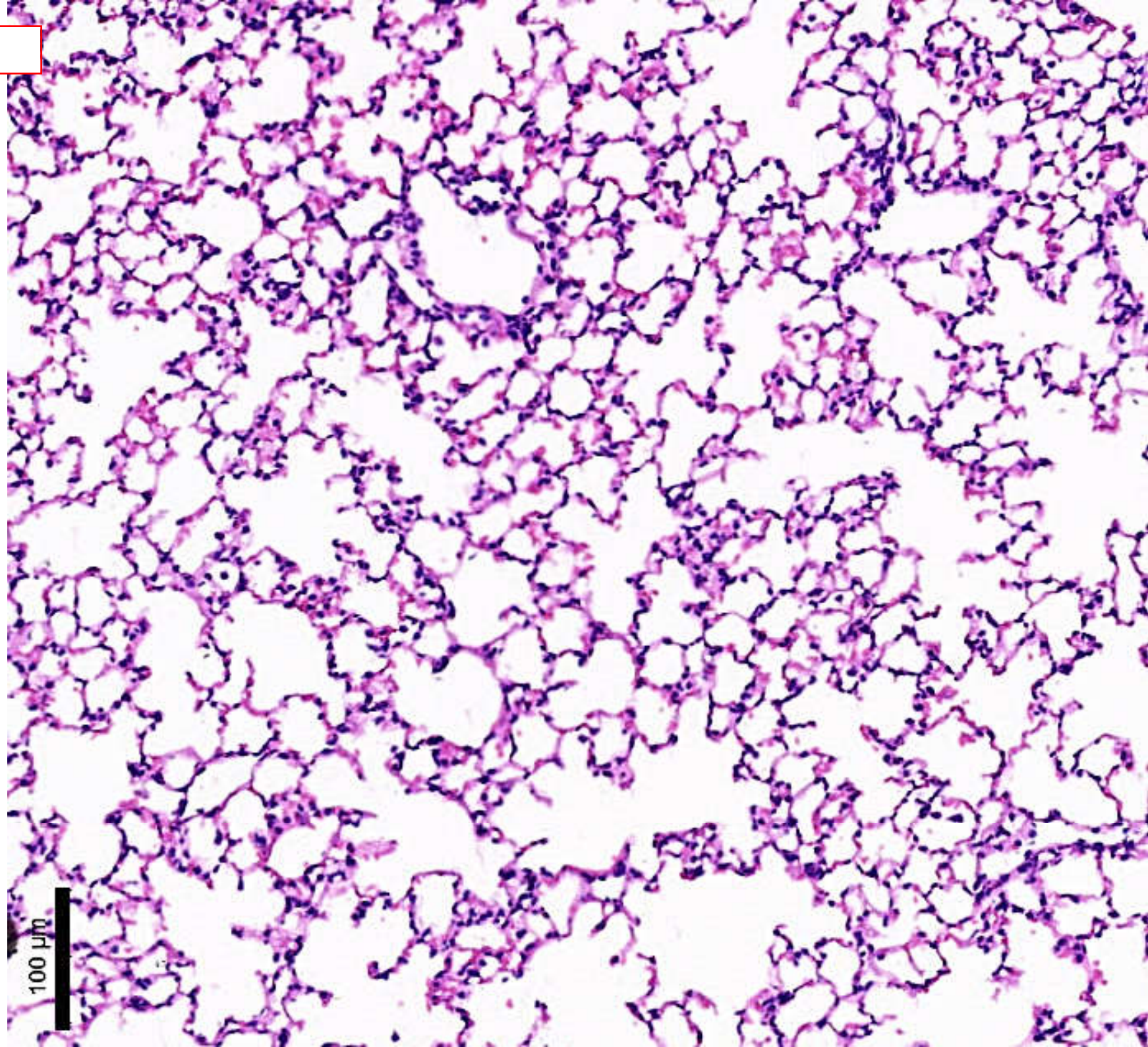

Group3

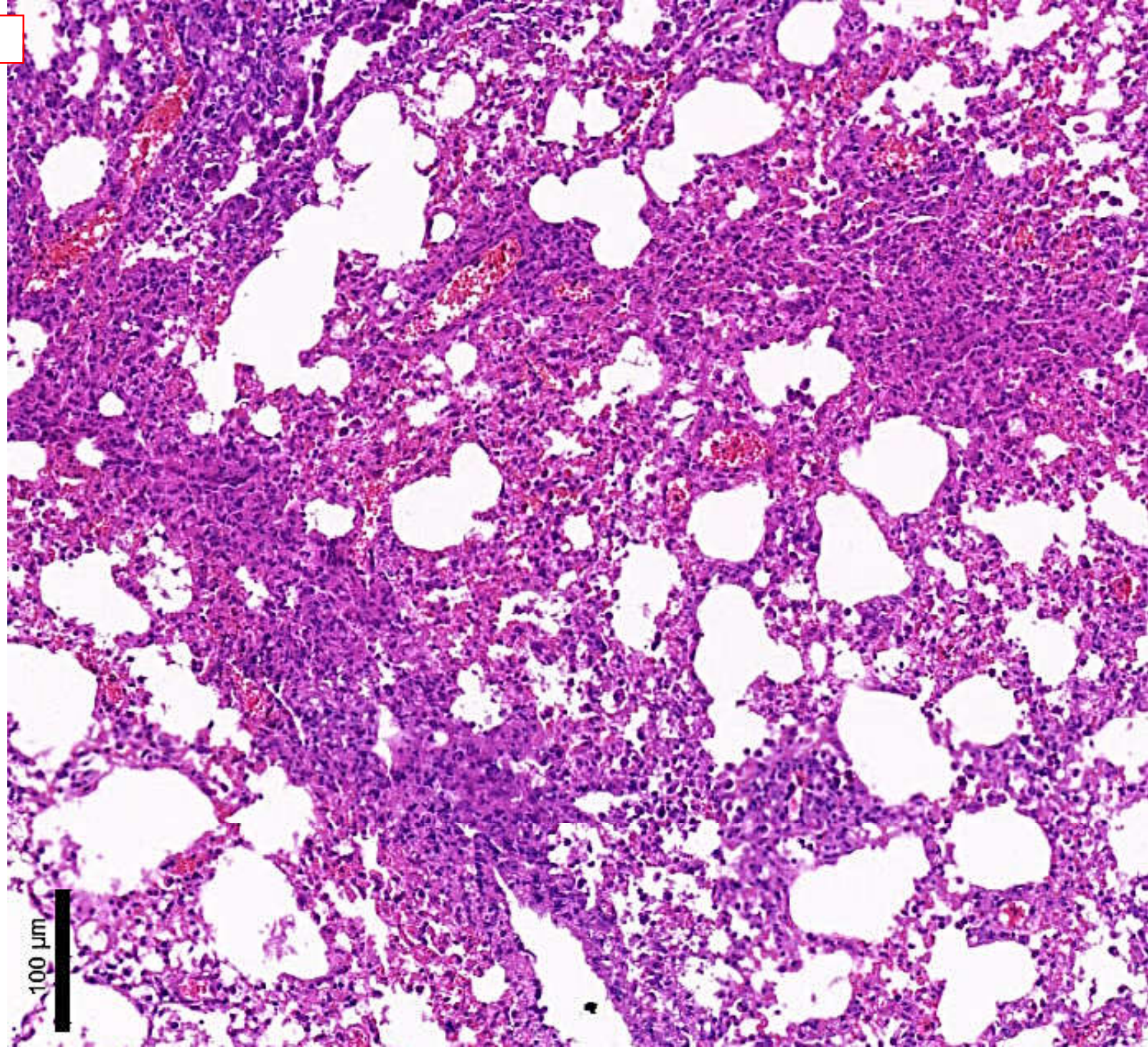

Group5

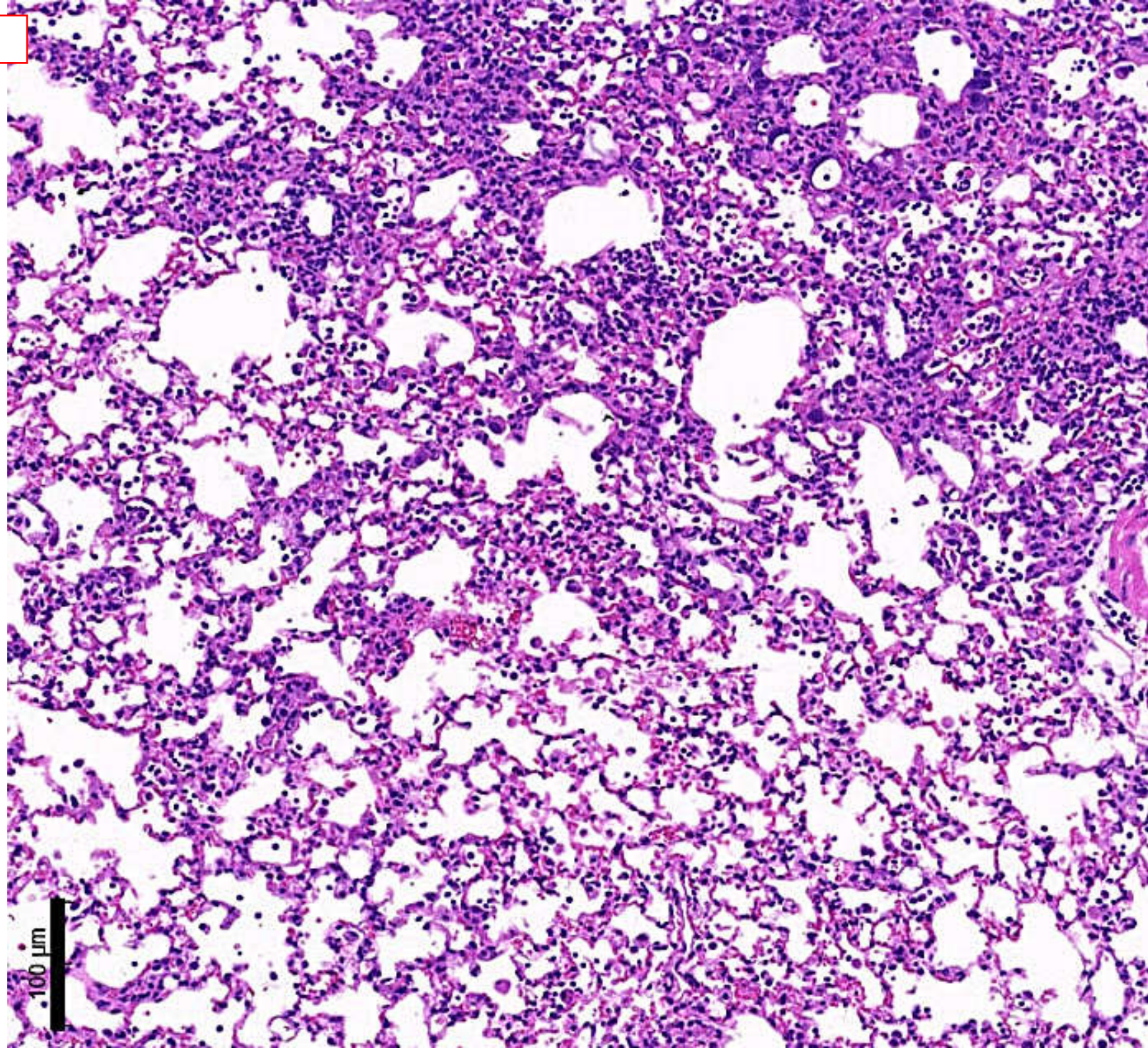

Group4

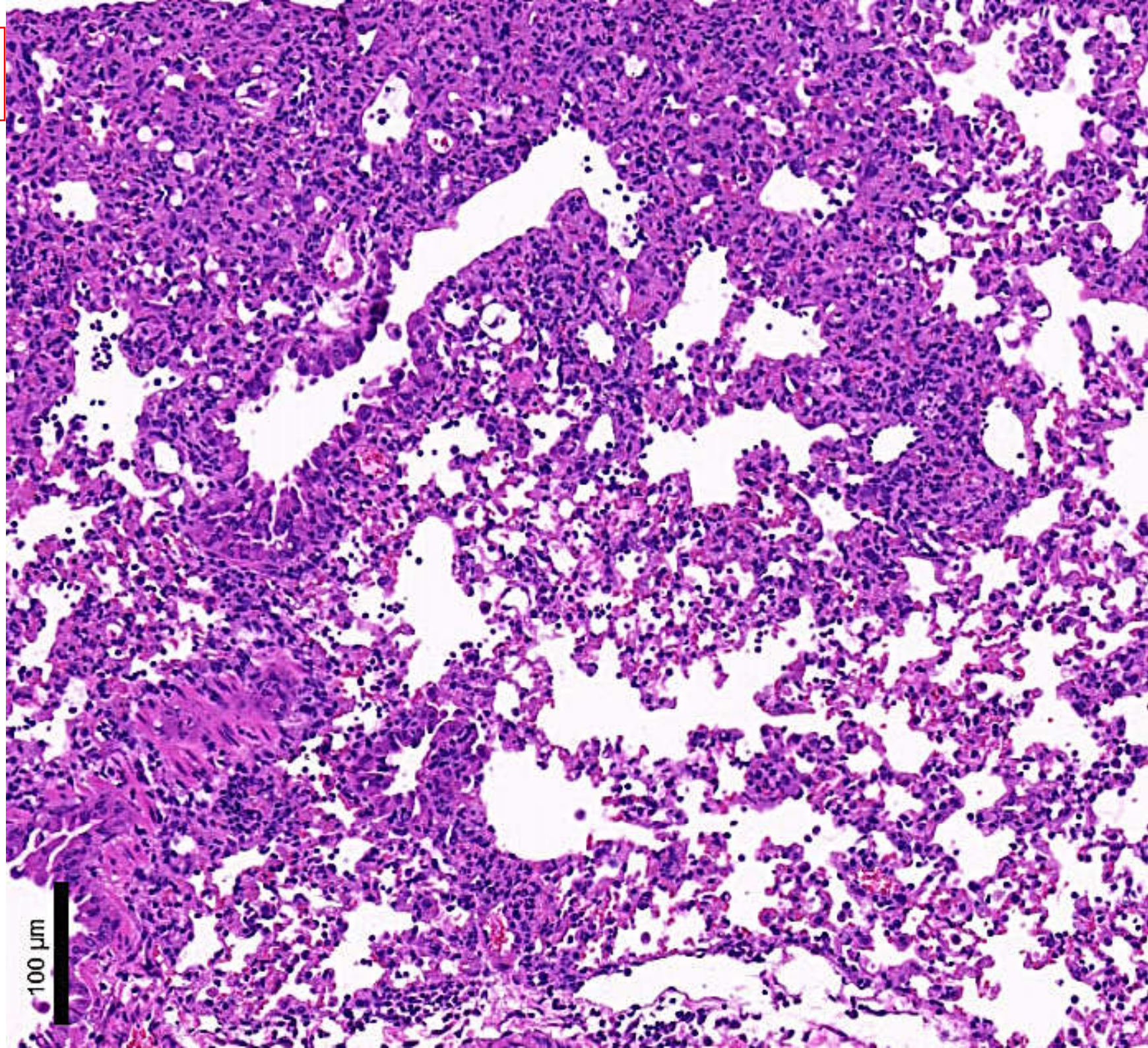

Supplement: Supplementary file 10 — Source Data for Figure 6 [file EMMM-12-e10233-s009.zip › Figure_6A_.pdf]

Group2

50  $\mu$ m

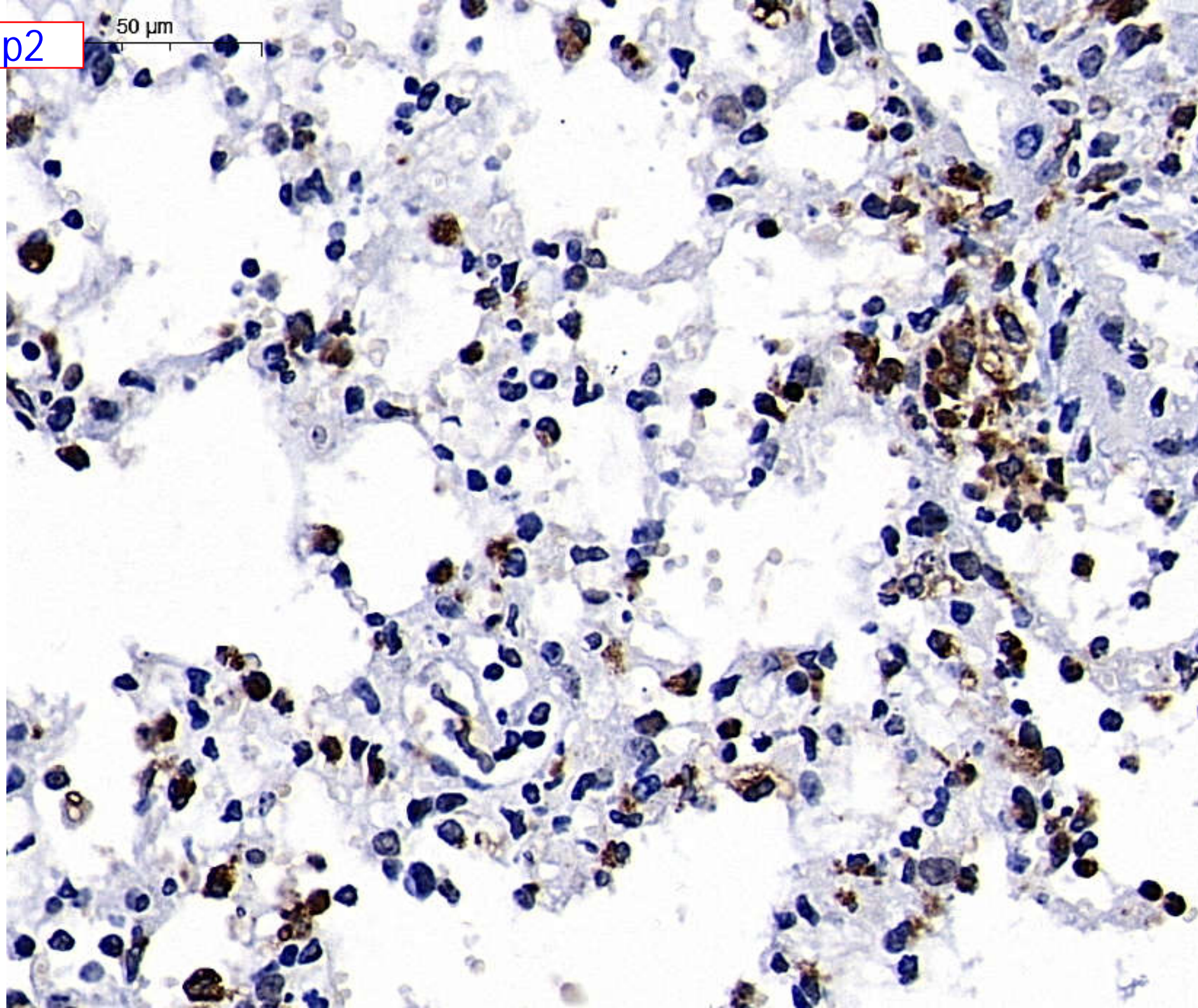

Group1

50  $\mu$ m

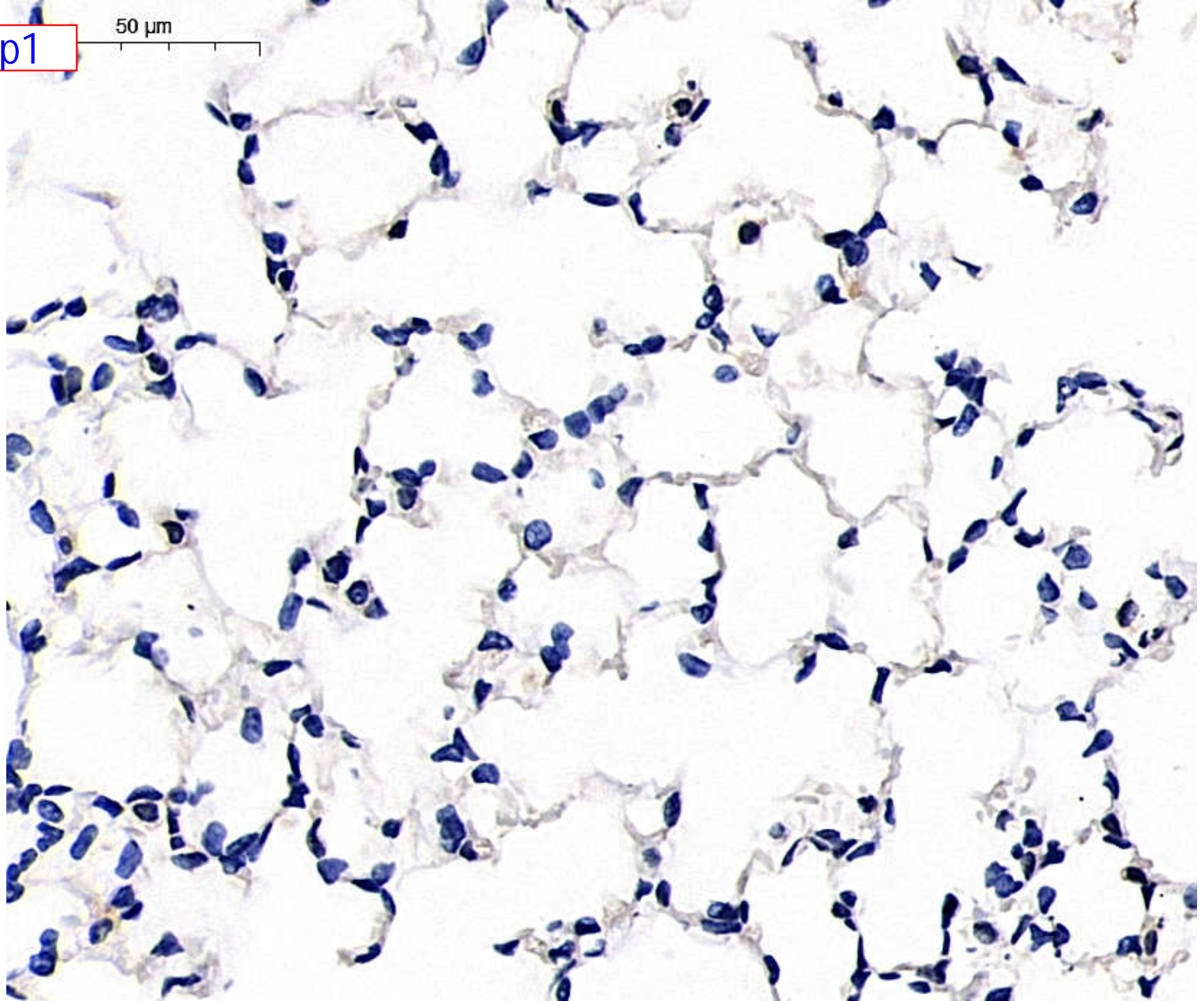

Group3

50  $\mu$ m

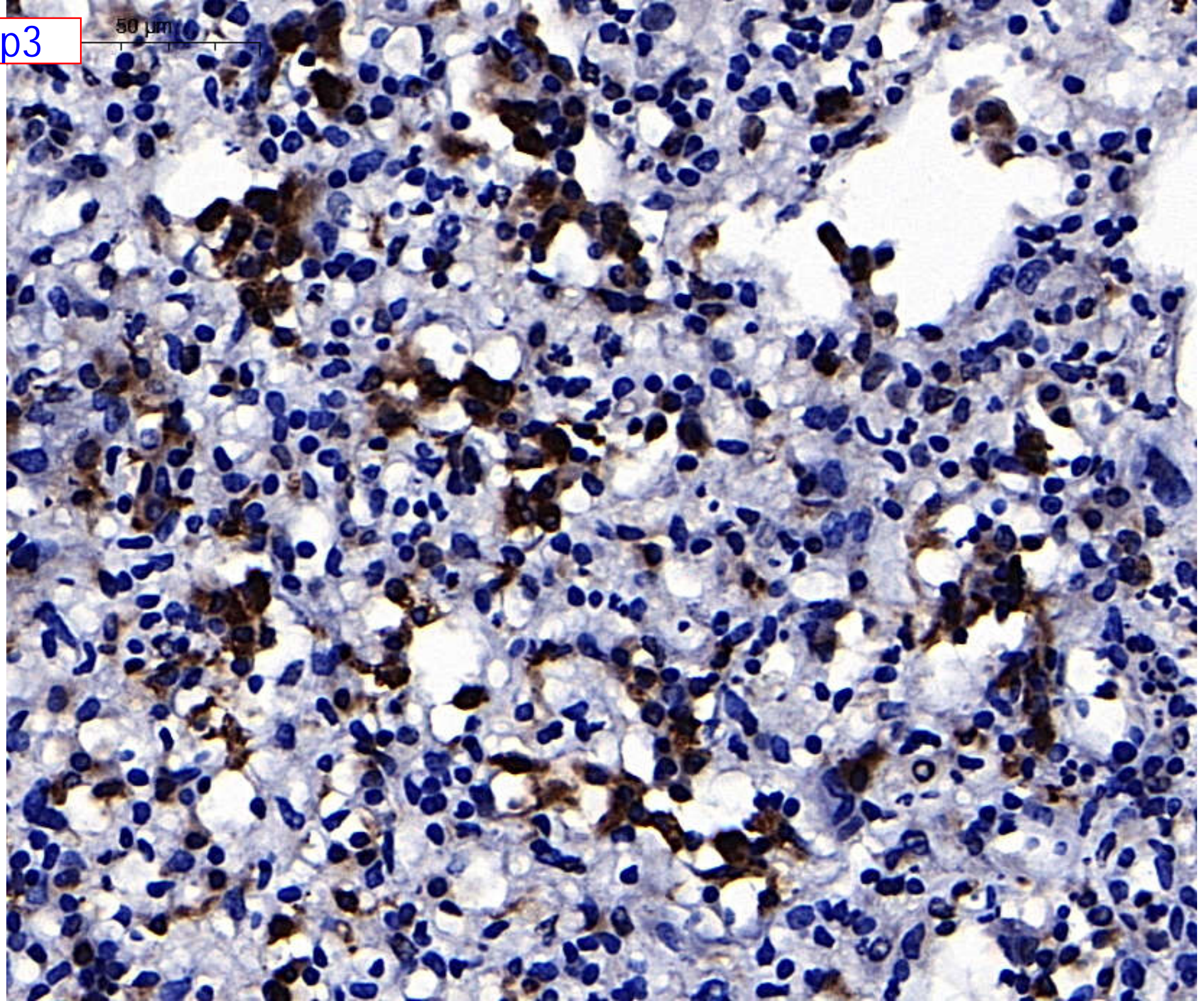

Group5

50  $\mu$ m

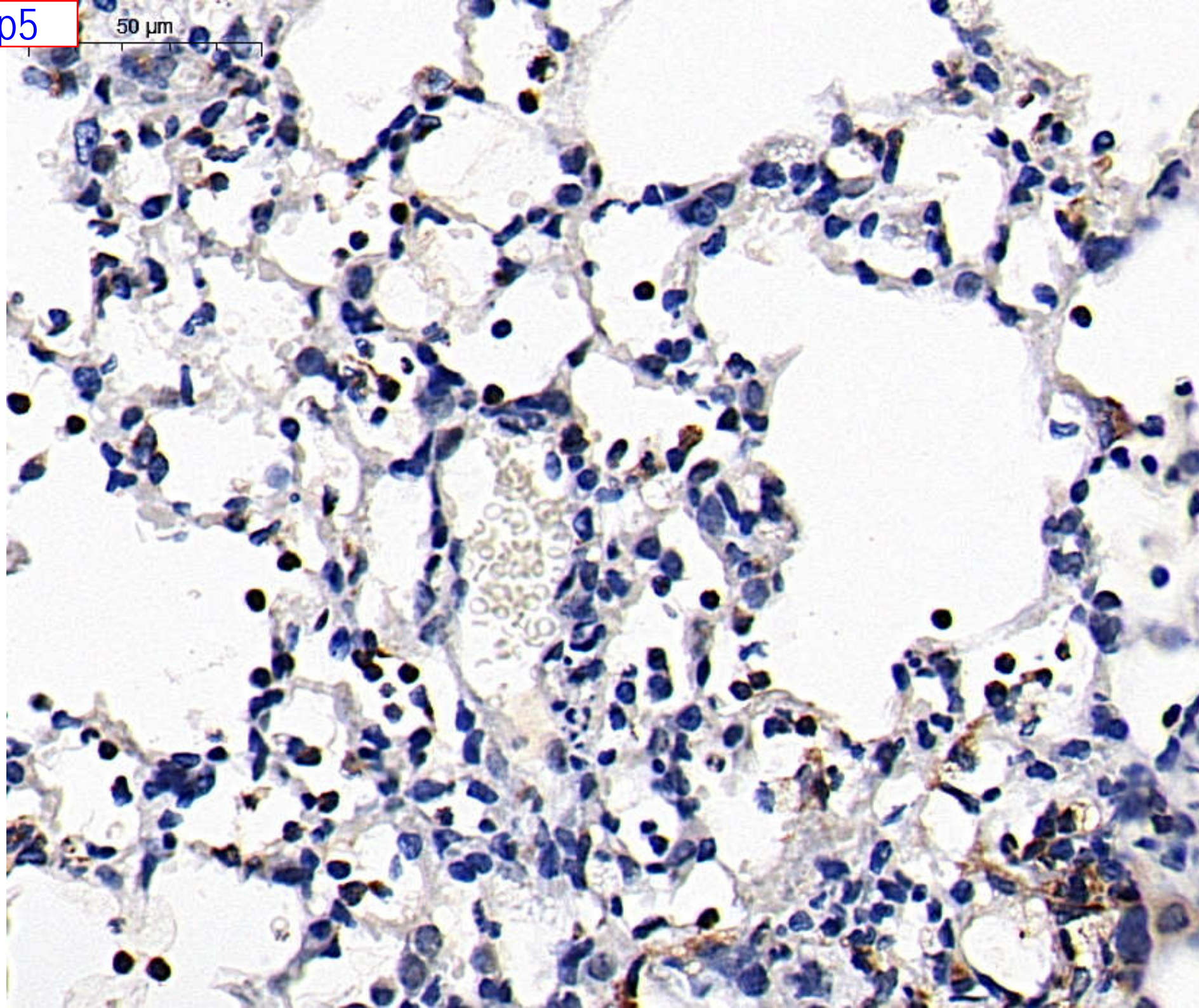

Group4

50  $\mu$ m

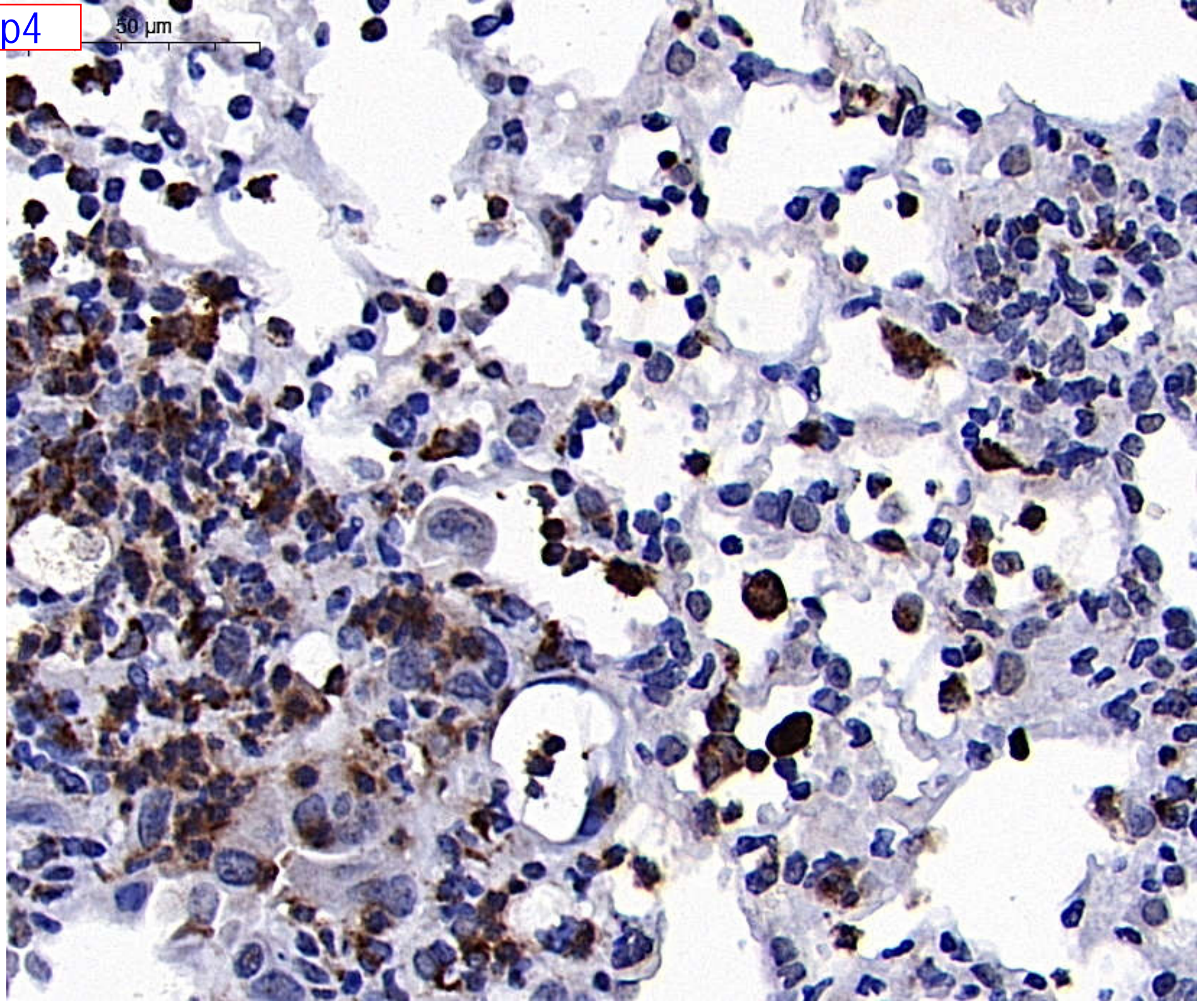

Supplement: Supplementary file 10 — Source Data for Figure 6 [file EMMM-12-e10233-s009.zip › Figure_6C_.pdf]

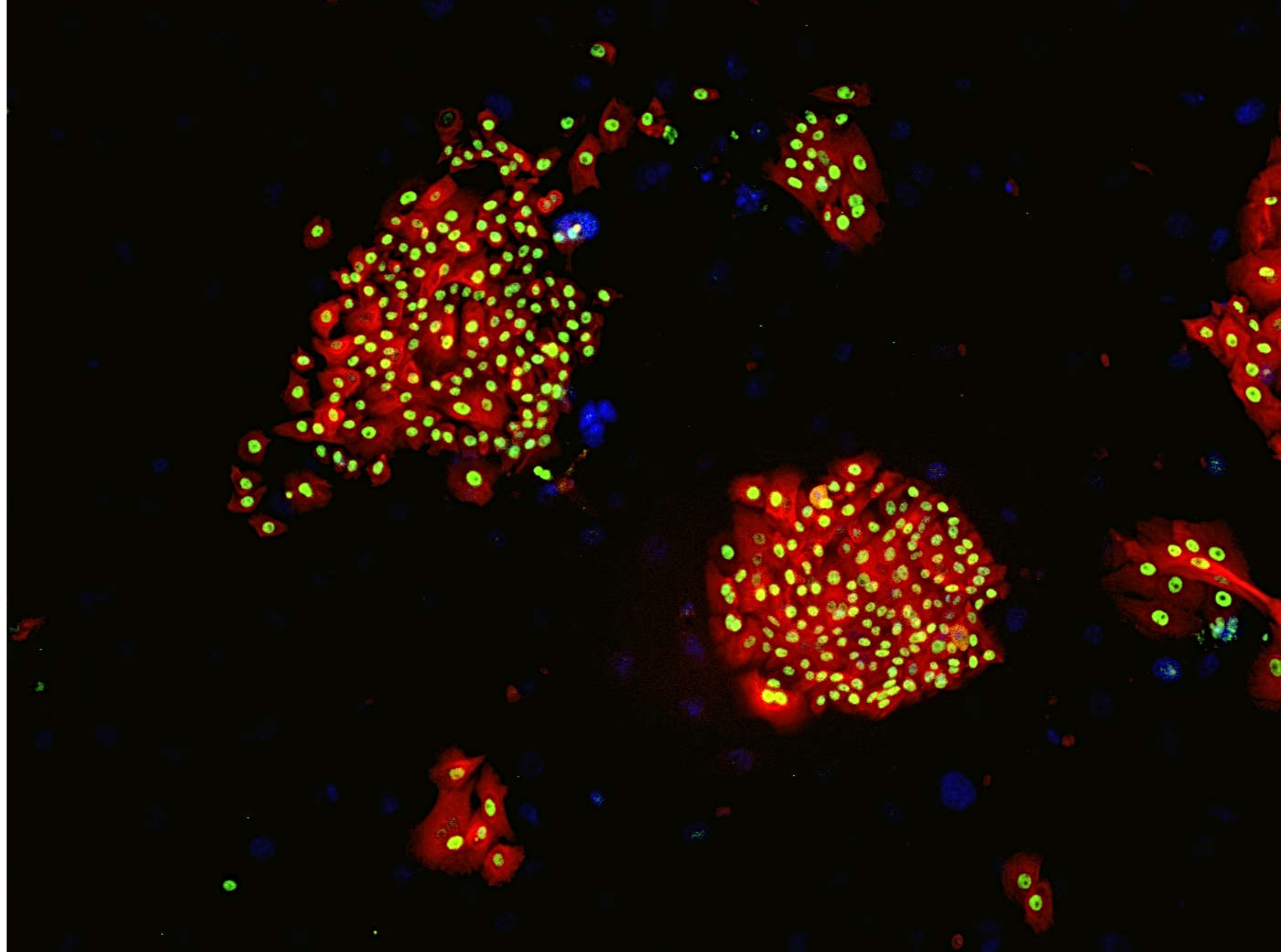

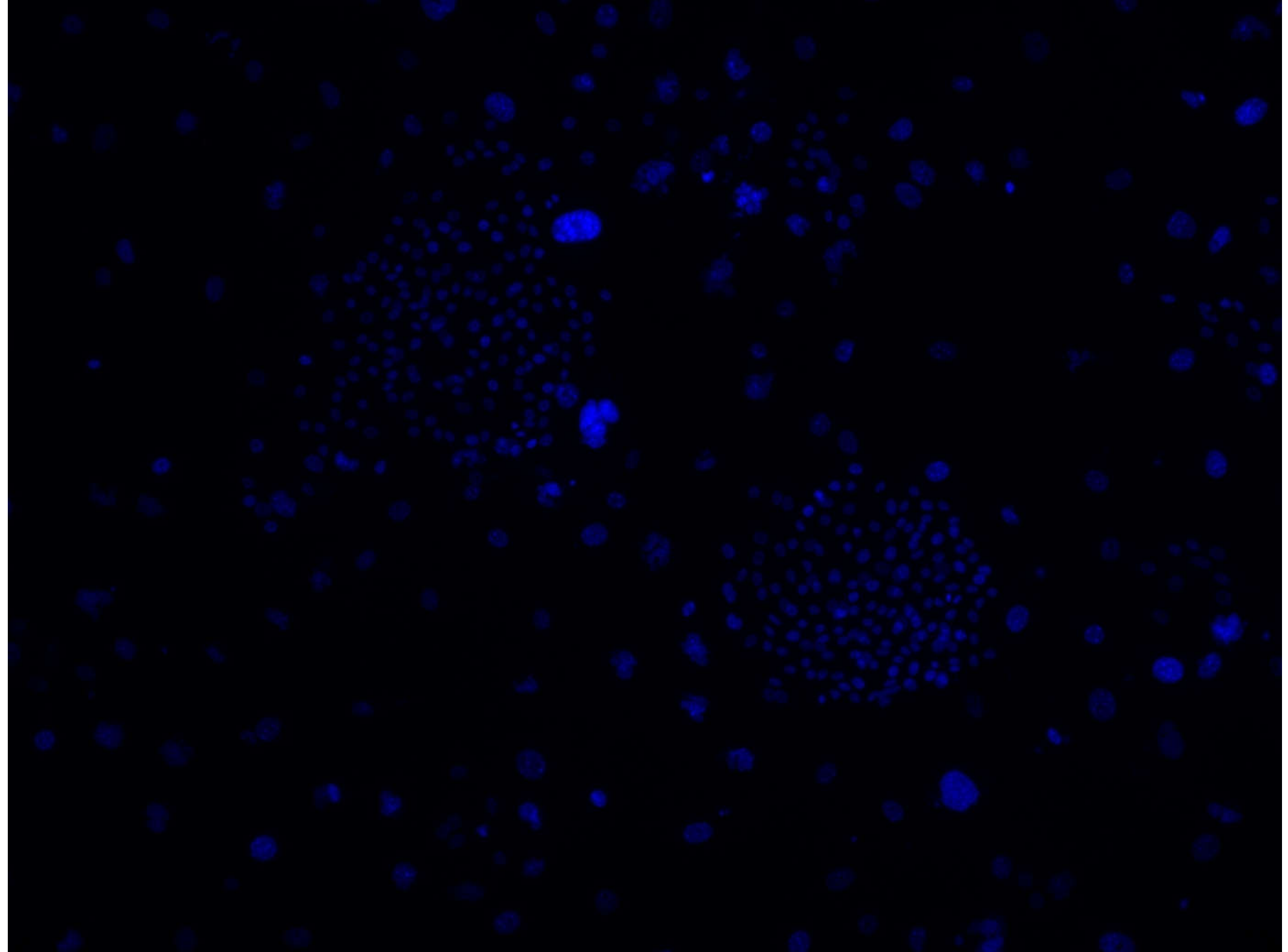

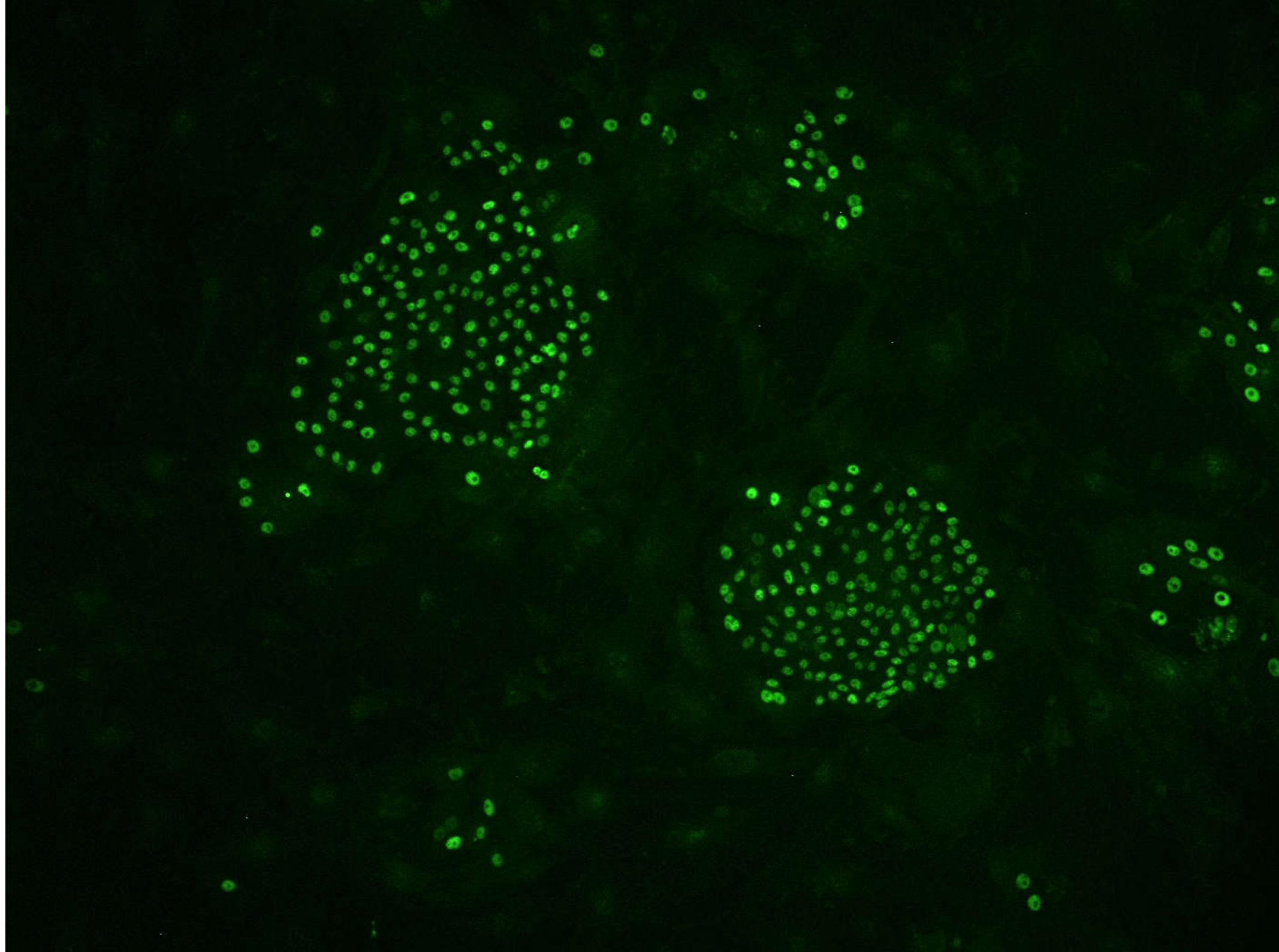

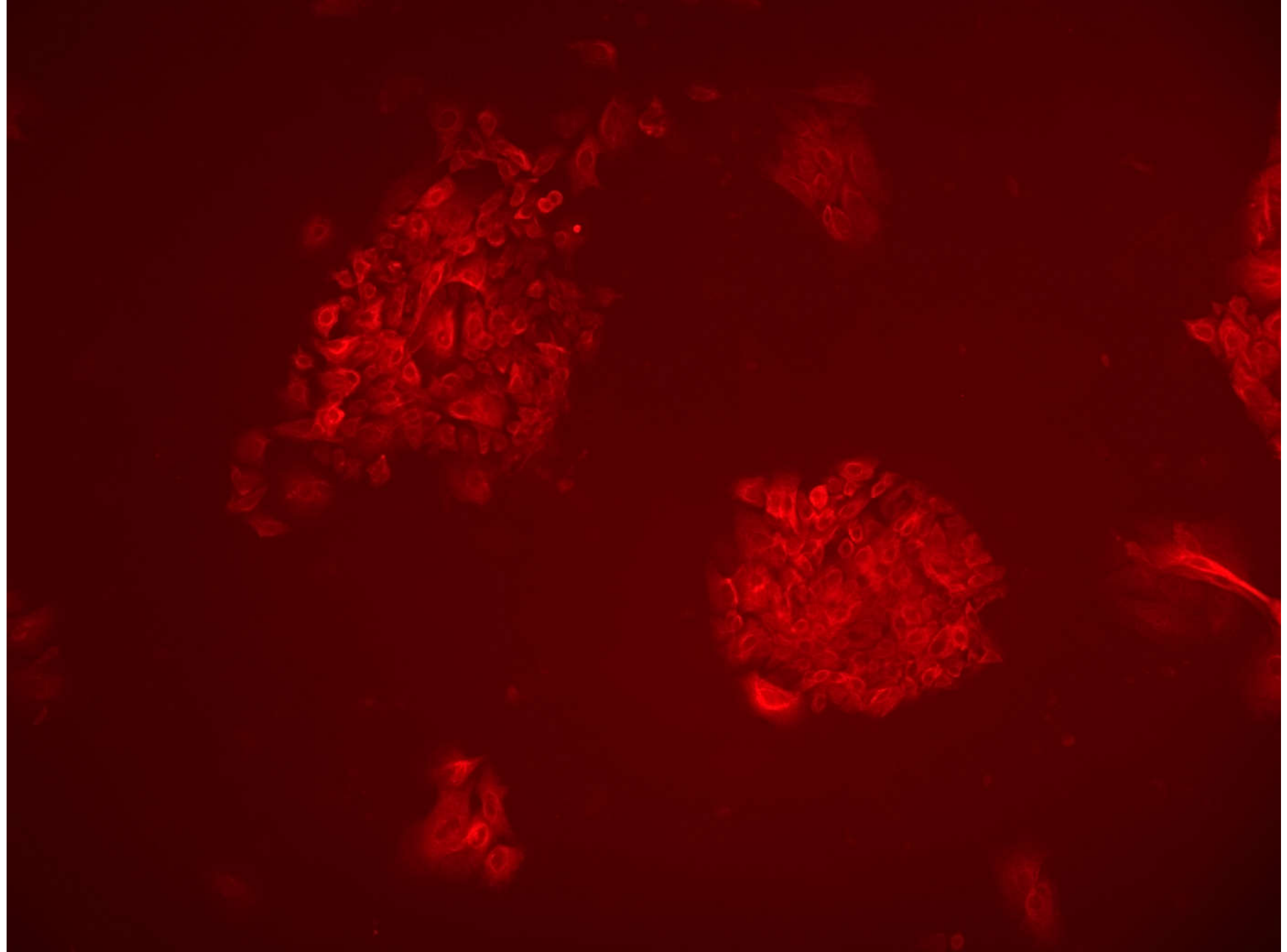

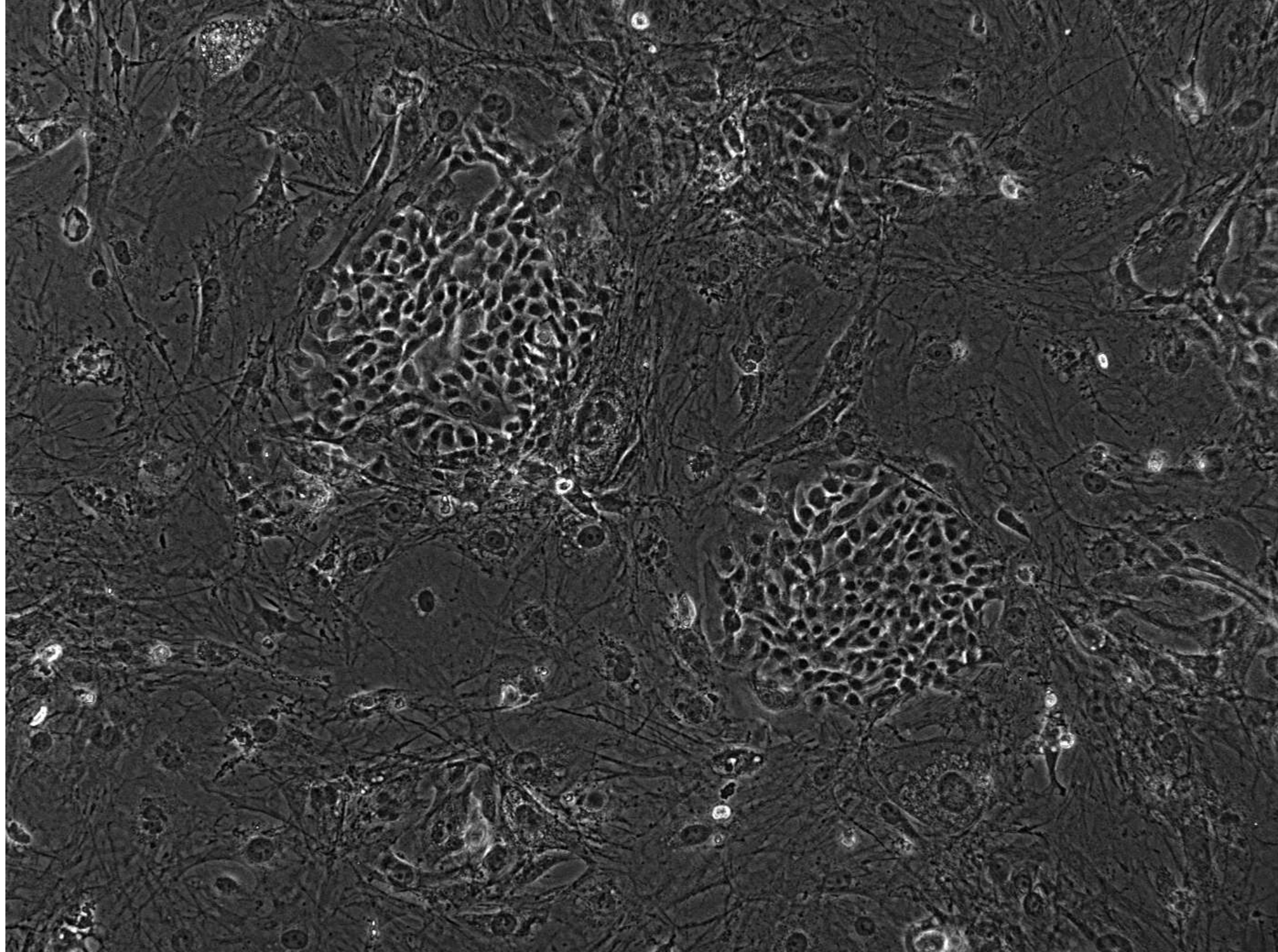

Supplement: Supplementary file 11 — Source Data for Figure 7 [file EMMM-12-e10233-s010.zip › Figure_7A.pdf]

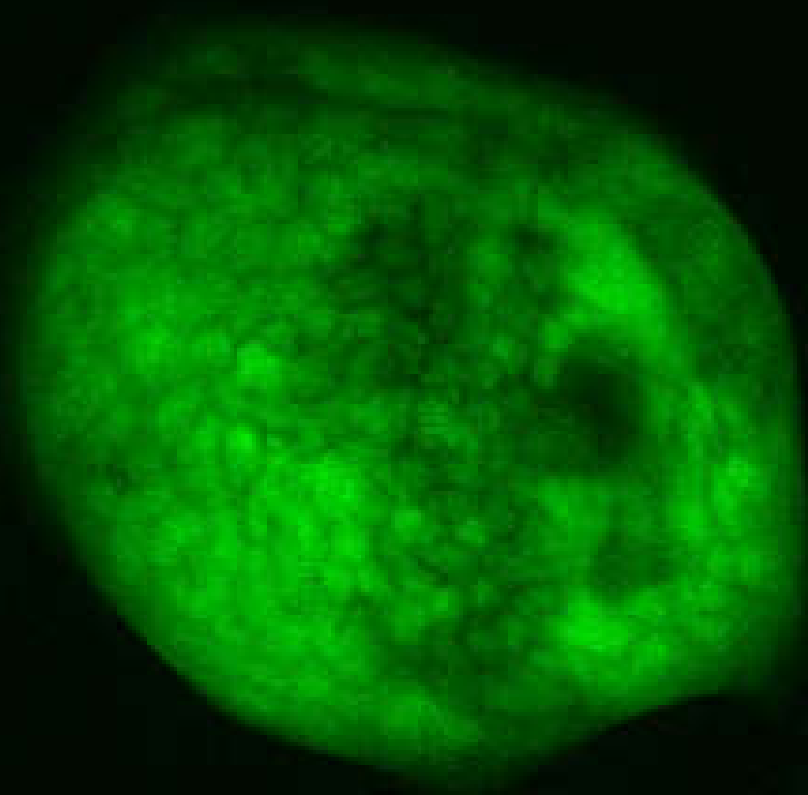

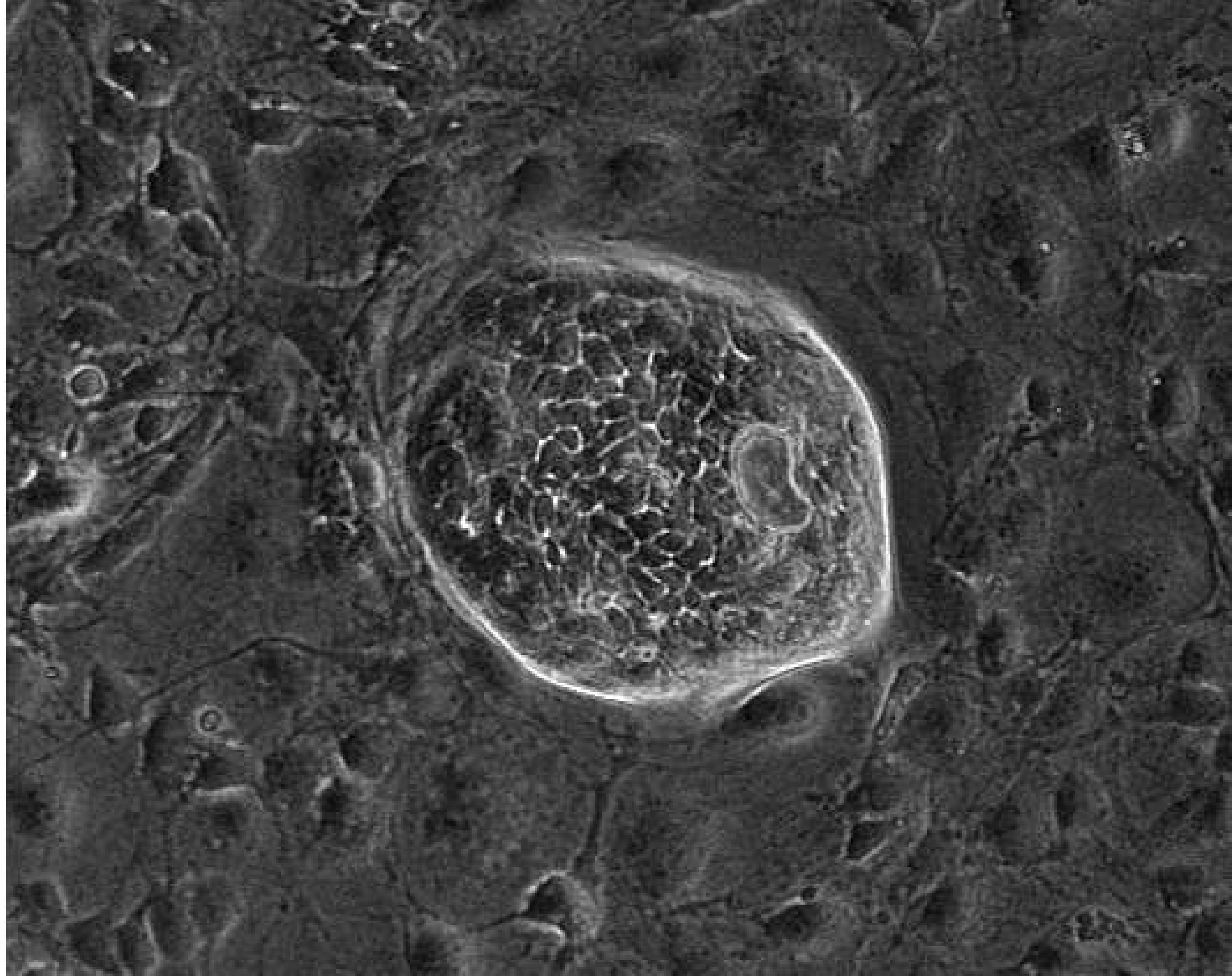

Supplement: Supplementary file 11 — Source Data for Figure 7 [file EMMM-12-e10233-s010.zip › Figure_7E.pdf]

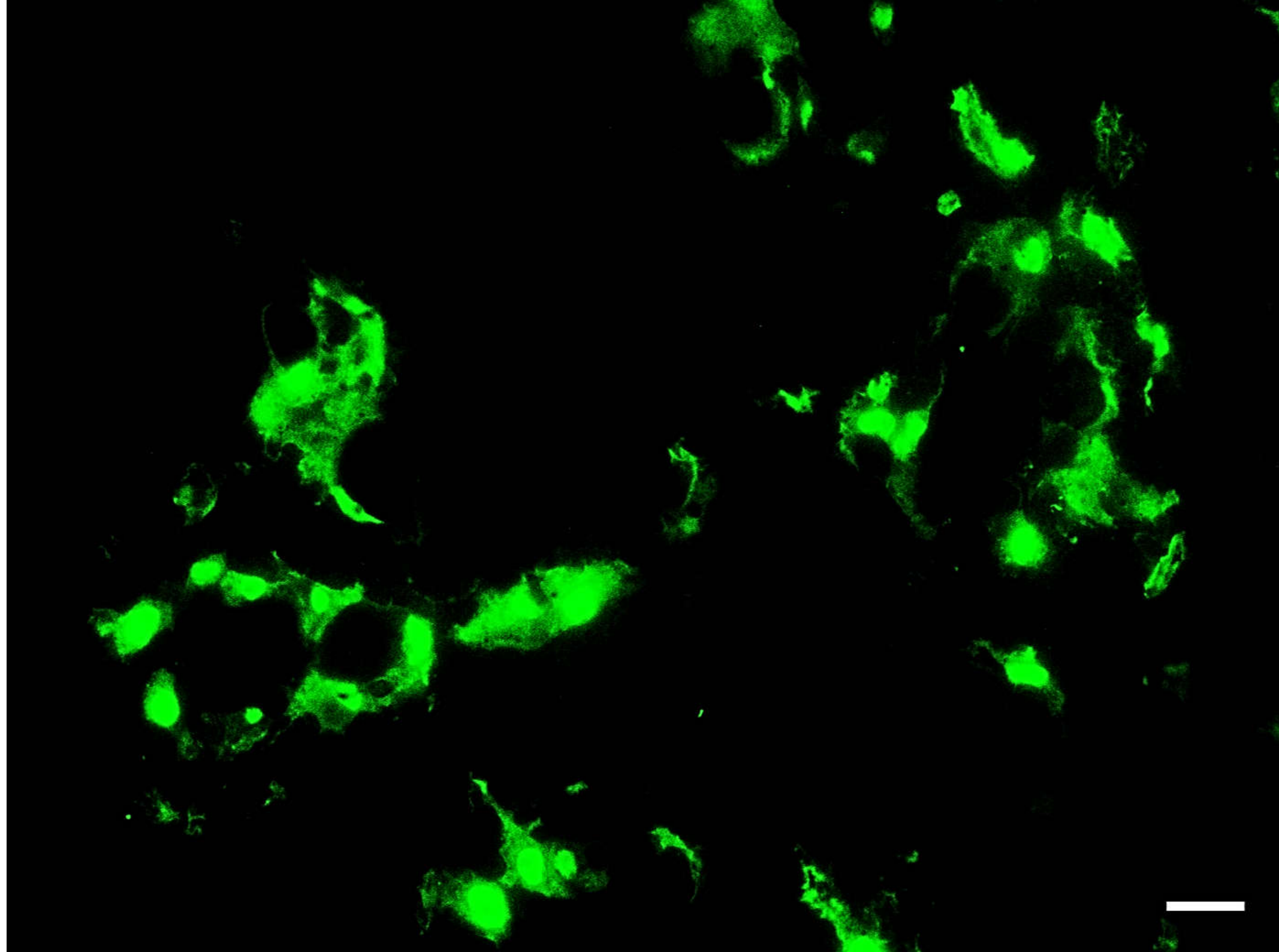

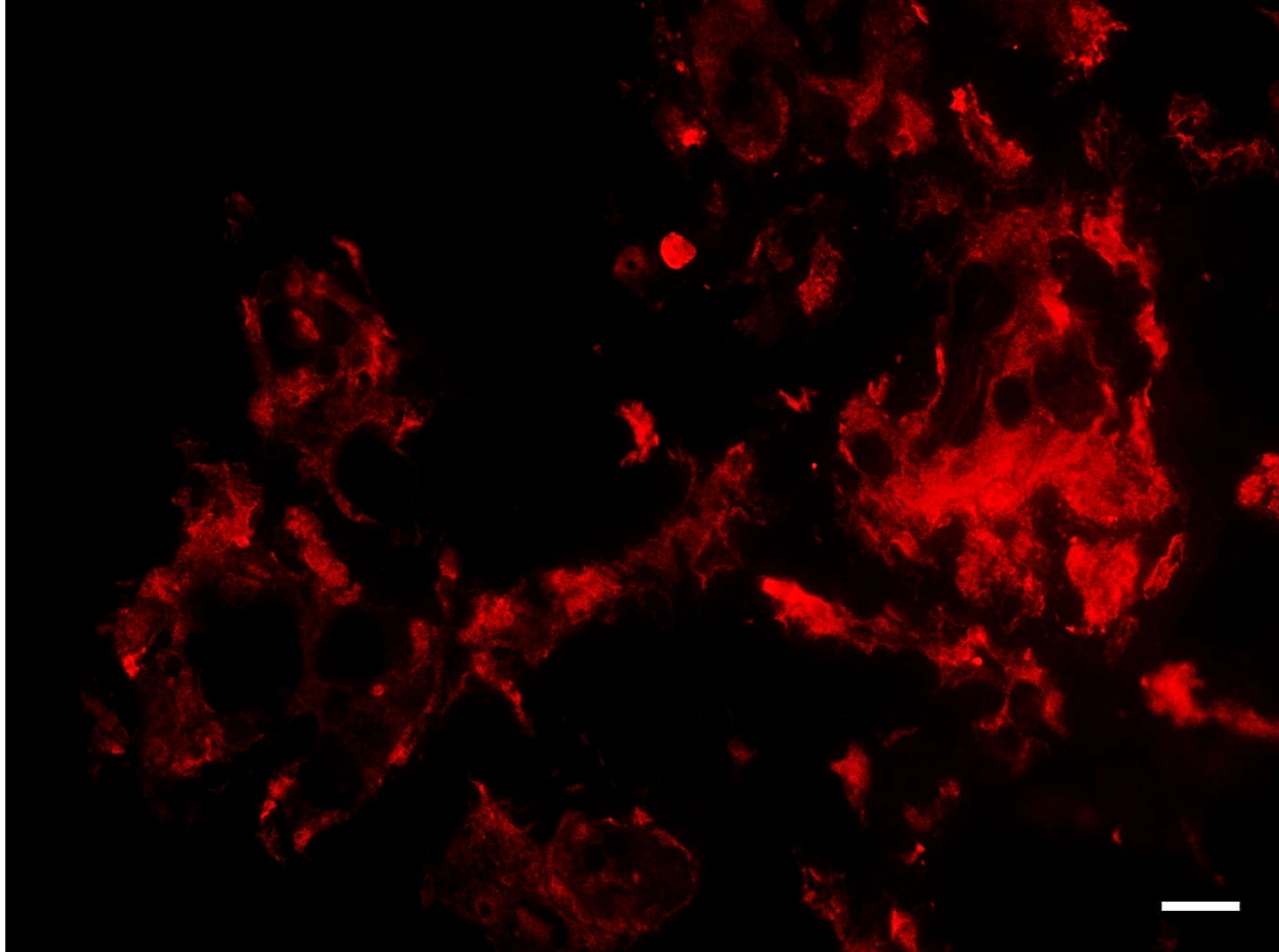

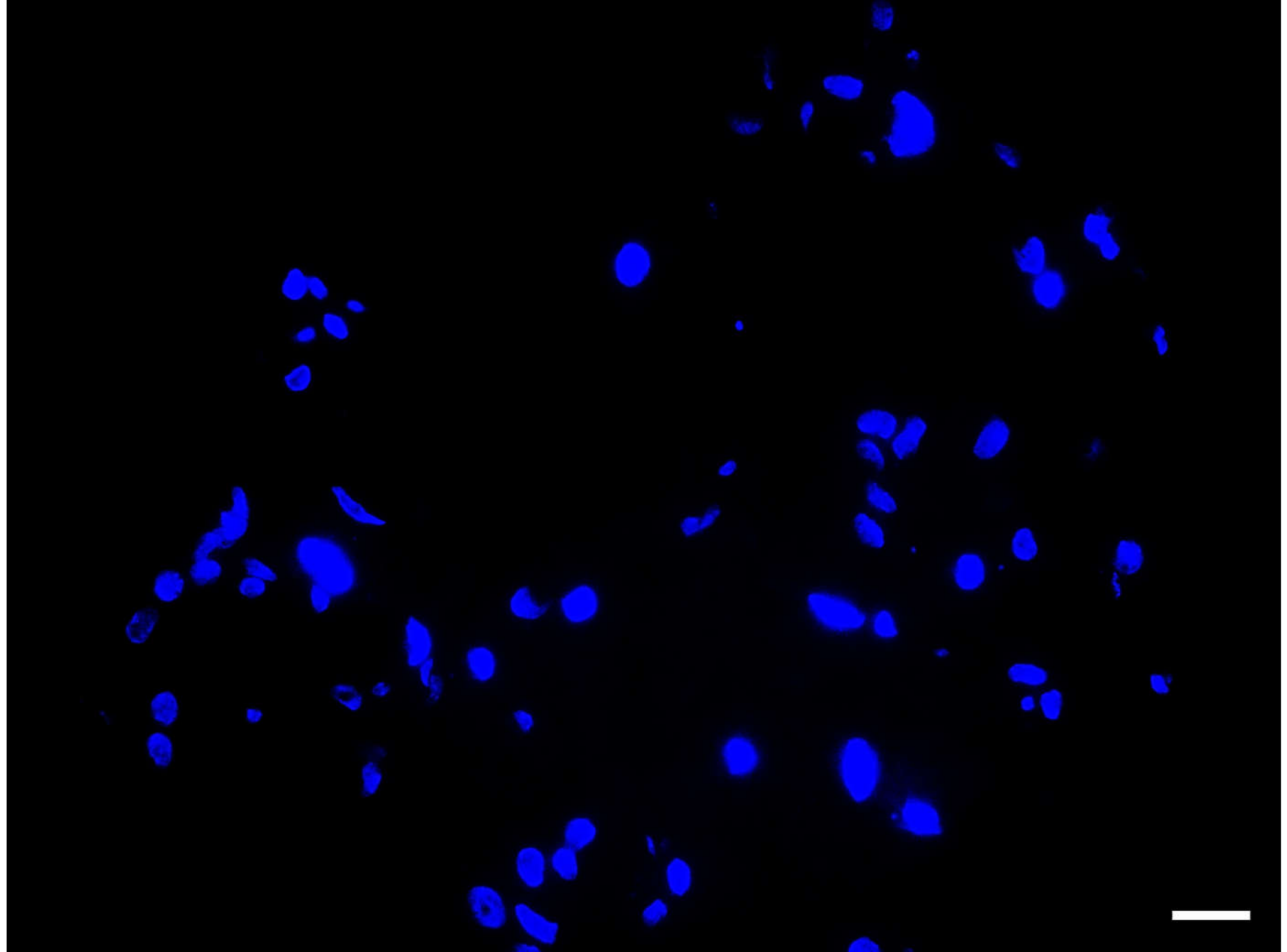

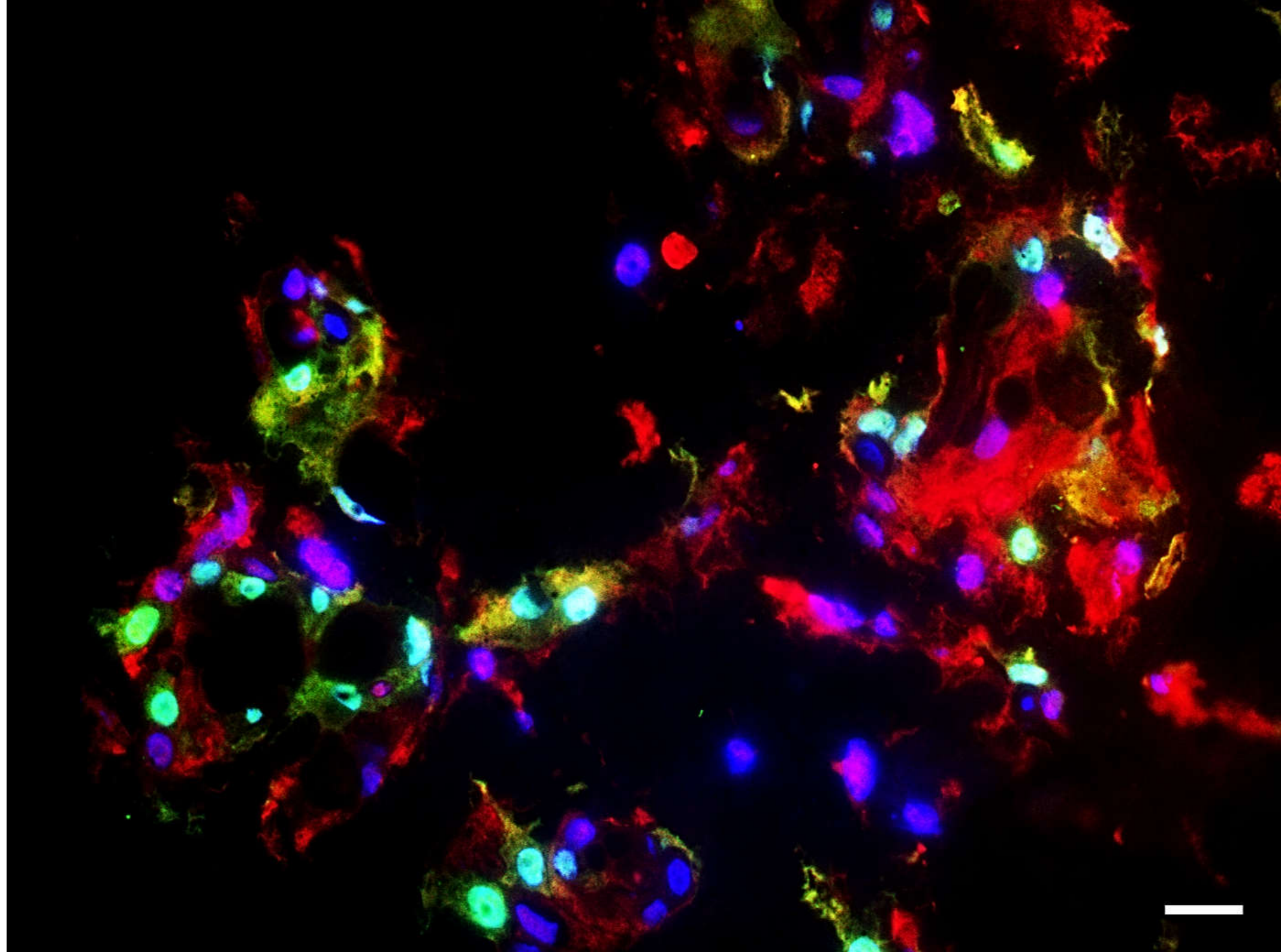

Supplement: Supplementary file 11 — Source Data for Figure 7 [file EMMM-12-e10233-s010.zip › Figure_7F_LL-37-hDASC.pdf]

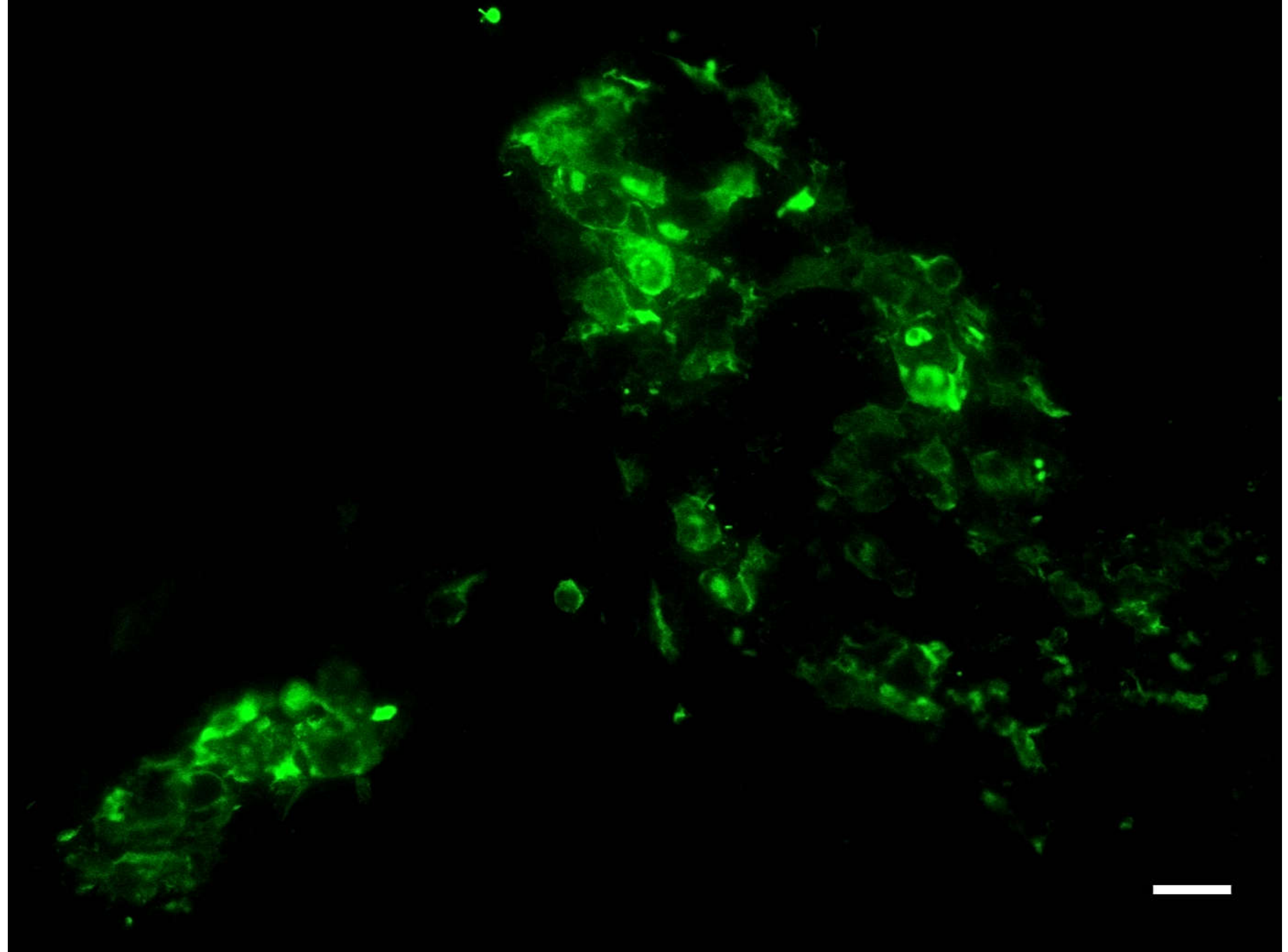

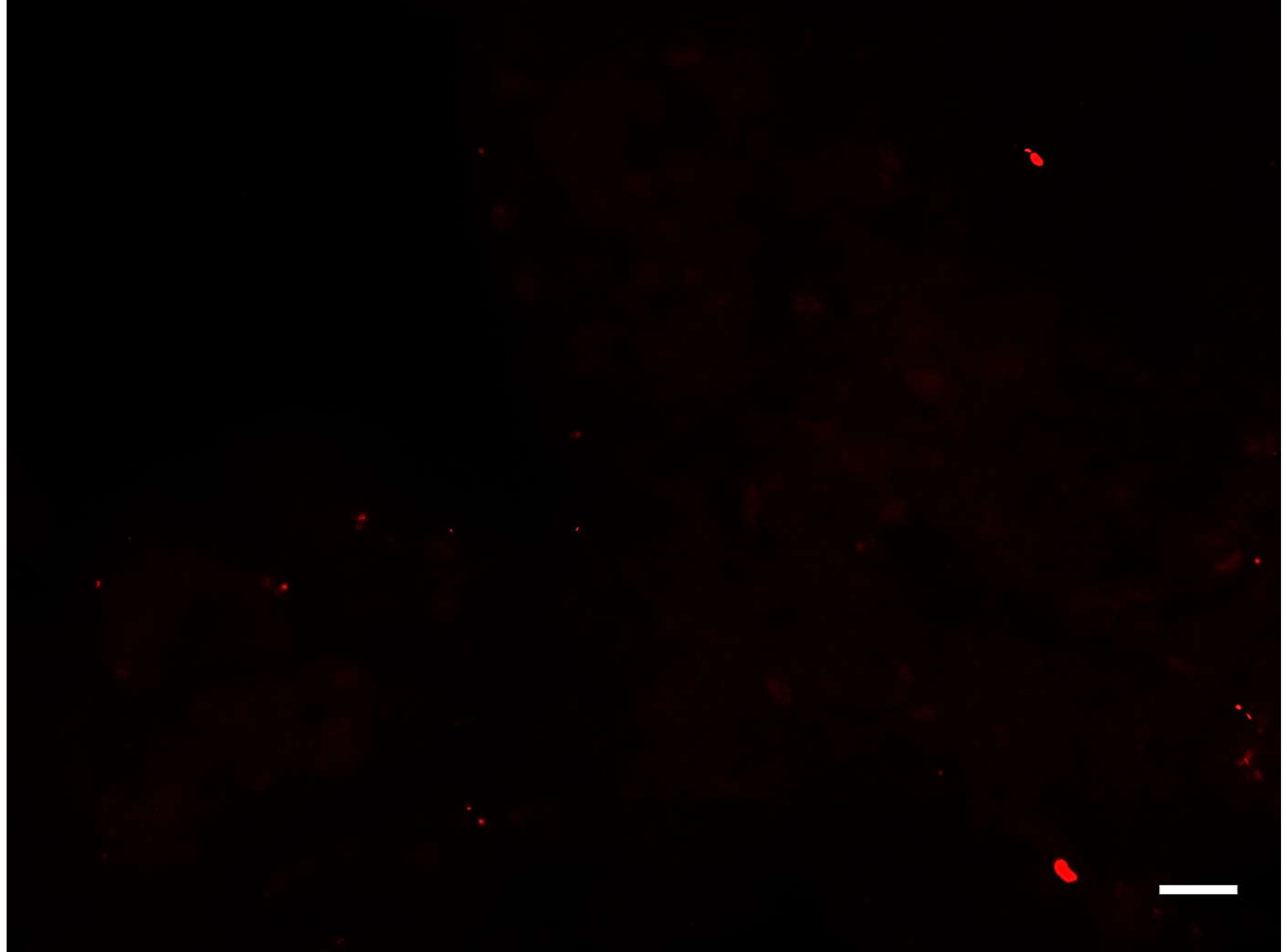

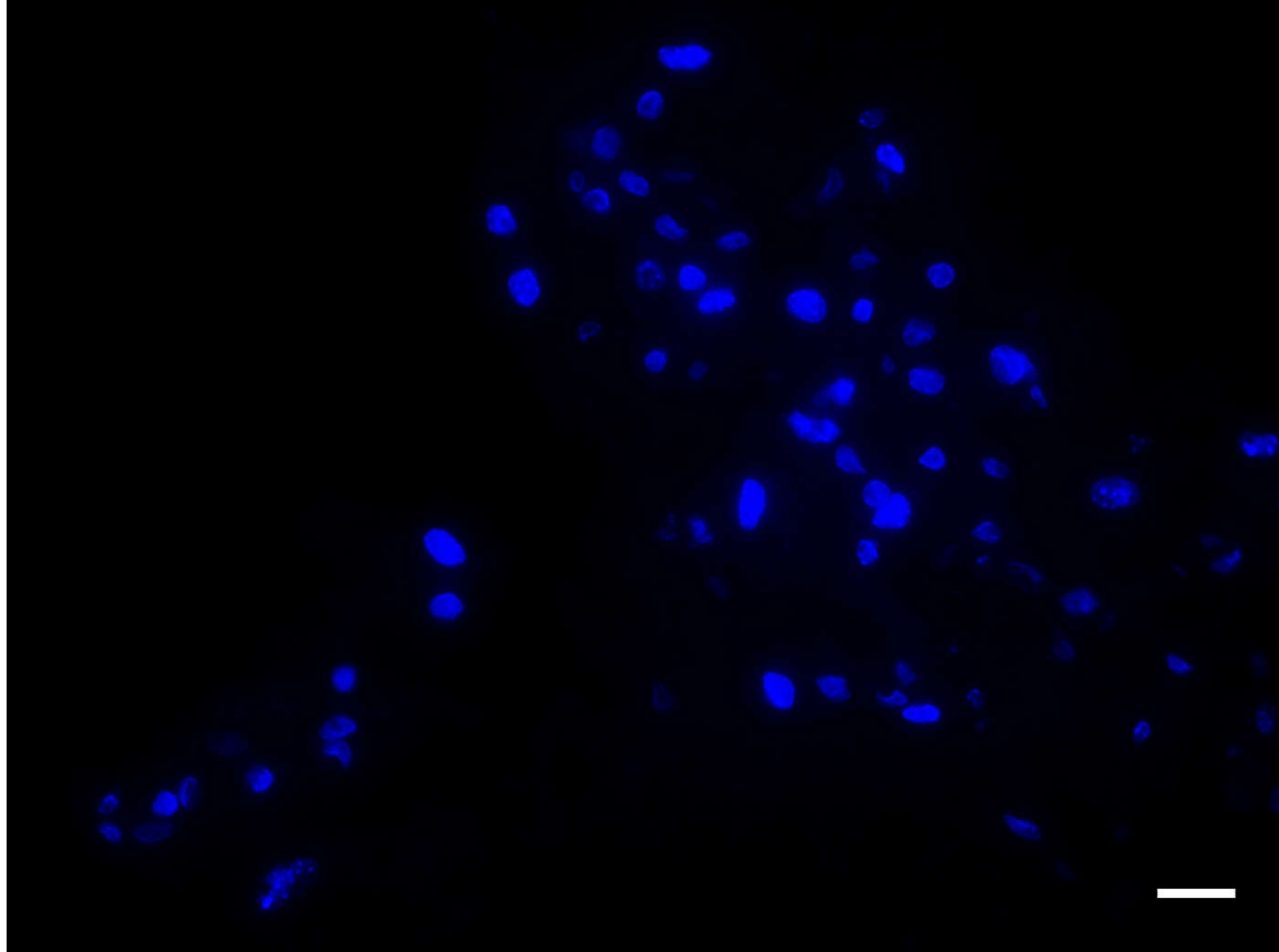

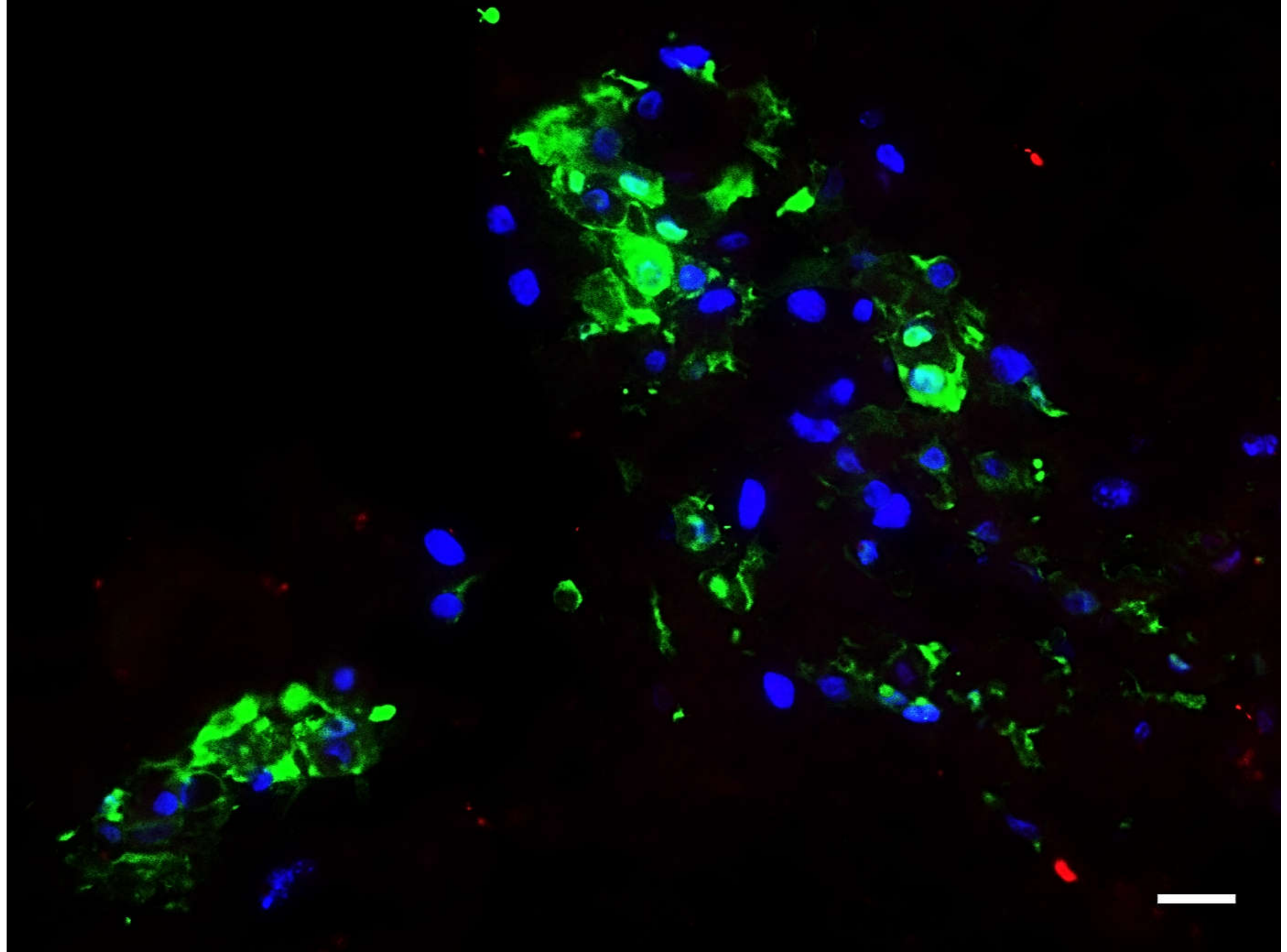

Supplement: Supplementary file 11 — Source Data for Figure 7 [file EMMM-12-e10233-s010.zip › Figure_7F_WT-hDASC.pdf]
