# Supplementary material for: Genetically engineered distal airway stem cell transplantation protects mice from pulmonary infection
Source: EMBO Mol Med. 2019 Nov 29;12(1):e10233. doi: 10.15252/emmm.201810233 (PMC6949487; doi:10.15252/emmm.201810233)
Supplement: Supplementary file 4 — Review Process File [file EMMM-12-e10233-s011.pdf]

EMBO Molecular Medicine

Genetically engineered distal airway stem cell transplantation protects mice from pulmonary infection

Yue-qing Zhou, Yun Shi, Ling Yang, Yu-fen Sun, Yu-fei Han, Zi-xian Zhao, Yu-jia Wang, Ying Liu, Yu Ma, Ting Zhang, Tao Ren, Tina P. Dale, Nicholas R. Forsyth, Fa-guang Jin, Jie-ming Qu, Wei Zuo, Jin-fu Xu

Review timeline:

Submission date:	21 December 2018
Editorial Decision:	15 January 2019
Revision received:	21 May 2019
Editorial Decision:	5 June 2019
Revision received:	4 September 2019
Editorial Decision:	17 October 2019
Revision received:	26 October 2019
Accepted:	4 November 2019

Editor: Lise Roth

Transaction Report:

(Note: With the exception of the correction of typographical or spelling errors that could be a source of ambiguity, letters and reports are not edited. The original formatting of letters and referee reports may not be reflected in this compilation.)

1st Editorial Decision 15 January 2019

Thank you for the submission of your manuscript to our editorial office. We have now heard back from the two referees whom we asked to evaluate your manuscript.

As you will see, the referees acknowledge the novelty and interest of the study, however they also have serious and overlapping concerns that preclude further consideration of the article at this time. They realize that addressing these comments would require a lot of additional work, time and effort.

As clear and conclusive insight into a novel clinically relevant observation is key for publication in EMBO Molecular Medicine, and together with the fact that we only accept papers that receive enthusiastic support upon initial review, I am afraid that we cannot offer to consider the manuscript further.

Given the potential interest and novelty of the findings, we would, however, be willing to consider a new manuscript on the same topic if at some time in the near future you obtained data that would considerably strengthen the message of the study and address the referees concerns in full. To be completely clear, however, I would like to stress that if you were to send a new manuscript this would be treated as a new submission rather than a revision and would be reviewed afresh, in particular with respect to the literature and the novelty of your findings at the time of resubmission. If you decide to follow this route, please make sure you nevertheless upload a letter of response to the referees' comments.

I am sorry that I could not bring better news this time and hope that the referee comments are helpful in your continued work in this area.

***** Reviewer's comments *****

Referee #1 (Comments on Novelty/Model System for Author):

Manuscript Number: EMM-2018-10233
Title: Transplantation of genetically engineered distal airway stem cell protects mice from pulmonary infection.

Severe pulmonary infections pose a great threat to human health, and with the increase of drug-resistant strains, new challenges have been imposed to the treatment. In addition to the use of antibiotics to control infection, alleviating lung injury and promoting tissue repair may be alternative treatment strategy. The antimicrobial LL37 is nontoxic in nature with high antimicrobial activity and good selectivity. These properties make the antimicrobial peptides promising for applications such as antimicrobial agents in medical industries. In this study, the authors engineered antimicrobial peptides into distal airway stem cells, major regenerative cells after large-scale lung damage, and studied their therapeutic effects in pulmonary infection. The authors used multiple methods to evaluate therapeutic effect of LL37 engineered stem cells, including bacterial load, histological image changes, lung ventilation index and the ability of lung tissue scaffolds to repair after mDASC cell transplantation. In addition, the authors used sophisticated in vitro 3D Culture technology to probe the anti-infective effects of LL37 in human DASC. Overall, the experimental design is logical and novel with potential clinical application value in future.

Referee #1 (Remarks for Author):

This study is very significant with potential for alternative pneumonia therapy. Meanwhile, some of the experiment technologies are demanding and most of the data are convincing. However, authors have not done well in dissecting the underlying mechanisms. There were errors with data presentation and deficiency in infection models, which may get help from technical experts.

Main concerns:
1. 5x108 CFU of PAO1 is very high does that the mice should be dead in short time. The authors should present the survival data and refer to any publication for the method. There is a concern about the method and the mouse model. Same question for in vitro experiment, No MOI was stated and 16 h would kill all cells if the MOI was more than one.

2. After infection, CFU in control lung and LL37-lung homogenate, Figure 4d and Figure 1a are almost the same result, why twice?

3. The authors intend to confirm the protective effect of gene engineered distal airway stem cells expressing LL37 on pulmonary infection. LL37 cathelicidin is main effect factor, but how to exclude the effect of mDASC or hDASC cells itself on pulmonary infection. In Figure 6g, the statistical difference in inflammation levels between non-transplanted DASC and transplanted DASC should be compared.

4. In vitro infection experiments, no specific MOI was given. In Figure 5 inflammation level of control-lung group was severe in histopathology, but in Figure 5b, the expression of cytokine IL-1β and TNFα is low in control lungs than normal lungs, these results seem to be somewhat contradictory.

5. When comparing images (also arrows should be added to highlight some features) or gel data, authors may use quantitative methods to clearly tell the differences among the samples. It appears that several figures are missing controls. Sometime the study can be strengthened with time lines. The proinflammatory cytokines or related pathways may be measured to show some deeper mechanistic studies.

6. Statistics: some figures have not described the sample sizes (Fig. 1, ect.). Many comparisons should be done with non-parametric methods, and methods should be mentioned in the figures.

7. Material and method: No information for mice and rat. Age? Weight? Gender? Provider? Protocol? Bacterial strains?

8. Are the authors sure the left and the right panel is the same picture in figure 6a?

9. What the blue color dye was used in all fluorescence images? Figure 2d and figure 4a need some separation to indicate the merger.

10. Figure 2h, it is unclear which groups were infected. The line should begin from the second group.

11. Fig 5a is very poor quality and scale bar is needed.

12. Many grammatical errors in the manuscripts, i.e., missing "the" in the front of lung. Authors should take this seriously to improve the writing.

Minor concerns:
1. Partial writing is not standardized, e.g., il-1b, TNF-α.
2. Some full names are missing like "pcc".
3. Western blotting is poor quality.
4. More discussion can be added for figure 1e.

Referee #2 (Comments on Novelty/Model System for Author):

Unfortunately the single late time point (after almost complete bacterial clearance) makes it very difficult to assess biological significance. In additional there is a lack of sufficient characterisation of the transgenic mouse, distinction between hCAP18 and LL-37, quantification of LL-37 expression levels, characterisation of inflammatory responses, and mechanistic evaluation. These and other issues are detailed with suggestions below.

Referee #2 (Remarks for Author):

The manuscript by Zhou et al asks interesting questions about the impact of overexpression of the antimicrobial host defence peptide LL-37 in pulmonary infection, both using a transgenic mouse model and stem cell based therapeutic platform.
Overall the paper addresses interesting questions, but I unfortunately have multiple concerns about the approach to the models used, depth to which these are interrogated, the interpretation of the data that are provided, the lack of mechanistic evaluation, and the viability of such an approach as a future human therapeutic strategy. In addition, the manuscript is not sufficiently well referenced. The most important concerns are detailed below to try to help the authors to tackle this potentially interest project.

Comments
1) The introduction makes far too many important statements that are not backed up by references and/or uses references that are not directly relevant to the preceding statement. This needs to be addressed throughout. In particular, the manuscript does not refer to key publications relevant to this specific research (e.g. Bals et al 1999, JCI, 103(8): 1113-7; Beaumont et al 2014 PLOS One 9(6) e99029; Yu et al 2010 J. Immunol 185(2):1142-1149; Yang et al 2000 JExpMed 192(7):1069-74; Schaller Bals et al 2002 Am J Respir Crit Care Med 165(7): 992-995 and others). This is particularly important because these papers, together with Kovach et al 2012 J.Immunol, Bals 1999 I&I (both referenced but not fully discussed in context) and others, have led the field to view LL-37 (particularly in the context of Pseudomonas infection of the lung) to be an inducible, neutrophil chemotactic and inflammation enhancing, protective antimicrobial in Pseudmonas lung infection. It is critical that the data in this paper, which does not fit this concept, is properly discussed in that context.

2) The introduction is focused on the modulatory properties of LL-37, when the data presented (particularly in the later parts of the manuscript) probably relate more to the directly microbicidal properties of this peptide.

3) Throughout the manuscript, no clear attention is given to the distinction between hCAP-18 (the pre-propeptide product of the CAMP gene) and its active predominant cleavage product LL-37. This distinction is critical. It appears that the new transgenic mouse overexpresses hCAP-18 constitutively, but how much is cleaved to LL-37? Is it cleaved without inflammation (usually neutrophil Pr3 is required) or are neutrophils required? Is the level altered in inflammation? How much LL-37 is produced and precisely where and by which cells? How does it compare to levels of endogenous cathelicidin, does it affect this, and what might be the interplay between these. This information and more is absolutely necessary to understand the model.

4) The hCAP-18-overexpressing mice are an interesting novel line perhaps worthy of paper on their own, examining their phenotypes with and without inflammation and infection. All we are told is that they have "no noticeable phenotype". Given the existing literature, constitutive expression of LL-37 would be expected to have effects. As it stands, this is inadequate, and interpretation of the data presented requires detailed description of the model and what characterisation was performed. Given some published data it is possible that LL-37 may not have a large effect until inflammation is induced, but this needs to be characterised with a mechanistic evaluation.

4) The murine lung PAO1 challenge is not detailed with enough clarity in the methodology. It appears that the mice were given 5x10E8 cfu of PAO1 directly into the lung. This is a very large inoculum and it is surprising, in my experience, that the mice survived this. As far as I can tell the only timepoint assessed was 2 days later, by which time nearly the entire inoculum had been cleared in all the mice (only 500 - 1000 cfu / g of lung left in figure 1). It is very hard to determine the biological significance (albeit it is statistically significant) of having 500 rather than 1000 cfu of PAO1 left after 48 hours. Studying earlier timepoints will be essential to understand the dynamics of this effect, particularly when the histology shows such a dramatic difference that does not correlate well with the minimal cfu differences. Maybe this is because the LL-37 is having a predominantly modulatory effect on inflammation, but that needs to be clearly examined mechanistically. What are the difference in cellular responses and cytokines/chemokines over a time course in response to infection?

5) The histology needs to be backed up by full quantification and scoring of multiple animals throughout the paper, not just some representative images.

6) The transcriptomics is potentially fascinating but doesn't add much as presented. It shows very marked differences - unlike the cfu data. Why this discrepancy? It does present an opportunity to develop this into a mechanistic evaluation in the future though, together with immune effector cellular response profiling of these mice both with and without infection. However, it does not fit well with published data (as detailed above), which needs to be carefully examined and discussed.

7) At the end of the first results section the authors state "The data indicate that constitutive expression of LL-37 in mouse lung can protect the lung from bacterial infection and thereafter inflammation". This conclusion fits previously published data, but the data presented do not fit with existing knowledge and no alternative mechanism is presented. Just for example, could this be a rapid early microbicidal effect that minimises the inflammatory response required before there is much inflammation for LL-37 to enhance? Is the BALF itself directly microbicidal?

8) The genetic basis for the LL-37 expression by mDASC is not clear enough. Is this hCAP-18 expression (and if so, how is it cleaved in cellular systems that do not produce the required proteases), or just the LL-37? Is it constitutive and can levels be affected by inflammation? Where in the cells is it localised and how is it release if it is just LL-37 (not the full pre-propeptide hCAP-18 being expressed)? Fig 2b needs to state what size this band is (is it hCAP-18 or LL-37) and show any other bands. Figure 2a needs to show controls (e.g. no primary antibody and isotype control + secondary) to check specificity.

9) Is Fig 2h just looking at inhibition of proliferation (also Fig 6g)? What was the starting inoculum? Is there no bacterial killing? This needs to be much clearer. If so, how is this compatible with LL-37 being an effective antimicrobial? There is far more growth in the WT m-DASC than the controls - why is that? What statistical test was used? Is LL-37mDASC significantly different than control?

© European Molecular Biology Organization

Why was the anti-LL-37 antibody only used against E. coli when all the rest of the paper deals principally with PAO1?

10) In Fig 3 more detail on what the engraftment ratio is and how it is calculated is required, and the histology needs to make it clearer where these cells are engrafted. The statement that "there is no incorporation of GFP+ cells in uninjured lung" should be demonstrated.

11) Again the infection studies end up comparing tiny residual cfus (as little as 50 - 150 cfu /ml in panel e) at a single late time point, which is of questionable biological significance and does not provide mechanistic evaluation. In addition, the magnitude of difference for PAO1 in panel d is greater here than when the whole lung is overexpressing LL-37 in figure 1. How is that explained?

12) Although it is good to get some cytokine evaluation here, other time points should be looked at and also cellular responses. Also, surely the most important statistical evaluation to make is LL-37-lung vs control-lung. Is that significant? What is happening to native cathelicidin expression in this model and how does that impact?

13) In Fig 6b it is unclear whether this could distinguish between any native expression of hCAP-18/LL-37 and expression of the transgene. Might the former be upregulated in response to the infectious stimuli and if so, what impact might it have? In Fig 6h the LL-37 (red) does not seem to co-localise with the GFP. Why is this? What is the control is Fig 6g? I'm not entirely clear. The values of the studies in this figure are not totally evident.

14) More effort should be made to justify why an approach such as that presented here would be preferable to much simpler strategies, such as direct delivery of LL-37 (or related peptides) or the use of cathelicidin-inducing drugs (already in use in human trails), which could have much lesser long term consequences and risks. Given published data one might expect that damage areas having constitutive expression of LL-37 might have enhanced inflammation and neutrophil recruitment. This could be harmful if not controlled during resolution. Is this a viable therapeutic approach?

15) Statistical evaluation seems to be by ANOVA followed by T-test Is this the correct post-test approach for the one-way ANOVAs?

1st Revision - authors' response 21 May 2019

Response to Reviewer 1
(Reviewer's comments are paraphrased and appear in *Italic)*

1. 5×108 CFU of PAO1 is very high does that the mice should be dead in short time. The authors should present the survival data and refer to any publication for the method. There is a concern about the method and the mouse model. Same question for in vitro experiment, No MOI was stated and 16 h would kill all cells if the MOI was more than one.

Reply: We appreciate the reviewer's comments.
(1) In Fig. 1, we used 5×10^6 CFU of PAO1 for each mouse. The protocol for the animal infection model is the following: we prepared 5×10^8 CFU/ml of PAO1 as the initial concentration of bacteria solution. Before infecting mice, 10 ul of the initial bacteria solution was diluted to 30 ul with PBS that was instilled into each mouse.

(2) We added the survival data as below Figure R1. Mice began to die at 15 h after PAO1 (5×10^8 CFU) administration, and all mice died at 30 h. Mice did not survive to 45 h post-infection when mice were injured by PAO1 (5×10^7 CFU). Mice were alive after 72 h post-infection of PAO1 administration with doses of 5×10^6 CFU or 5×10^5 CFU per mouse. In this study we chose the dose of 5×10^6 CFU for each mouse for the infection model. Since the Beaumont study demonstrated the capacity of LL-37 to enhance pulmonary bacterial clearance at 6 h and 24 h (Beaumont, P. E. et al. Cathelicidin host defense peptide augments clearance of pulmonary Pseudomonas aeruginosa infection by its influence on neutrophil function in vivo. PloS one 2014. 9, e99029, doi: 10.1371/journal.pone.0099029), we observed the bacterial clearance capacity of LL-37 at 48 h. Our mouse model is supported in several papers, such as 'Yu FS et. al. 2010 J Immunol. 185(2):1142-9;

Torres IM, et al 2017, Am J Physiol Lung Cell Mol Physiol. 314 (2):L225-L235' and 'David Nobuhiro Douda et. al. 2011 J Immunol. 187 (4) 1856-1865'.

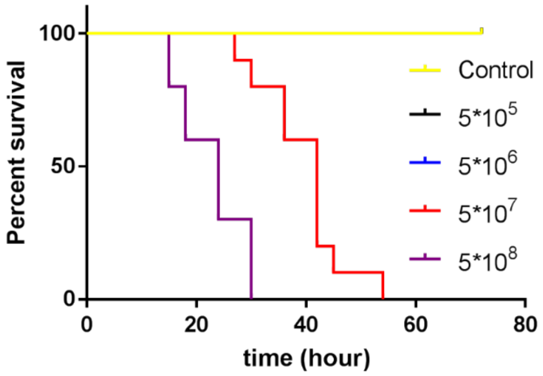

Figure R1. Survival rate for the mice pulmonary infection model using different dilutions of PAO1 *Pseudomonas aeruginosa*.

(3) To better present the mouse model, we have newly provided the schedule for the mice experiments in Fig. 4C in the new submission. The bacterial colonies were seeded from agar-plates kept at 4°C and placed in a shaking incubator overnight at 37℃ in liquid Luria-Bertani (LB) medium; bacterial concentrations were validated by plating on LB agar and counting colony-forming units (CFU). Before each experiment, the bacterial cells were washed twice and resuspended in PBS. Mice were anesthetized with isoflurane, and the lung was injured by intratracheal instillation with 3 U/kg body weight bleomycin (Selleckchem, USA) in 30 µl volume on day −7. Then, mice were anesthetized, and *P. aeruginosa* or *E. coli* (5×10^6 CFU) with LL-37-mDASCs (1×10^6 cells) in 30 µl volume were instilled into the lung on day 0, while control mice received bacteria with mDASCs. Intratracheal aspiration was performed by instilling the bacteria and cells into the trachea via the mouth, as described in our previous publications. Two days post-infection, mice were sacrificed, and the lung samples and BALF were collected for analysis.

(4) We repeated the experiment *in vitro*, and the MOI has been reduced. Details involved in the MOI are presented in the new submission.

2. After infection, CFU in control lung and LL-37-lung homogenate, Figure 4d and Figure 1a are almost the same result, why twice?

Reply: Sorry for the confusion. They are not the same experiments. Figure 1A(old version) used transgenic mice expressing LL-37 compared to WT mice. Figure 4D shows mice transplanted with LL-37-mDASCs compared to mice transplanted with WT-mDASCs.

3. The authors intend to confirm the protective effect of gene engineered distal airway stem cells expressing LL-37 on pulmonary infection. LL-37 cathelicidin is main effect factor, but how to exclude the effect of mDASC or hDASC cells itself on pulmonary infection. In Figure 6g, the statistical difference in inflammation levels between non-transplanted DASC and transplanted DASC should be compared.

Reply:
(1) DASCs were proven to repair the damage induced by H1N1 influenza virus and promote regeneration (Zuo, W. *et al. Nature* **517**, 616-620 (2015).). In this study, the engineered DASCs possess both regenerative and antimicrobial capacity—the highlight of this research. Here, the difference in the inflammation levels between non-transplanted DASCs and transplanted DASCs is now compared in Fig. 5E of the new submission. These data show that mDASCs have protective effects on pulmonary infection, while LL-37-DASCs have a stronger resistance to bacterial infection than WT-DASC.

(2) In Fig. 6G, cells were cultured *ex vivo* and were not involved in inflammation; therefore, we could statistically analyze this. Yet, in the new submission, we performed statistical comparison between the blank and WT-mDASC groups.

4. In vitro infection experiments, no specific MOI was given. In Figure 5 inflammation level of control-lung group was severe in histopathology, but in Figure 5b, the expression of cytokine IL-1β and TNFα is low in control lungs than normal lungs, these results seem to be somewhat contradictory.

Reply: Thank you for the reminder.

(1) Details involved in the MOI are present in new submission Fig. 2.

(2) It is our fault for causing confusion regarding the group naming, and we thank you for the reminder. In our previous draft, 'Control-Lung' represented the lung that was challenged with bleomycin and then treated with WT-mDASCs, and 'Normal-Lung' represented the lung that was just challenged with bleomycin and then treated with PBS; accordingly, WT-mDASC treatment reduced the inflammatory responses compared with non-treatment. To clarify this, we have rearranged the images in Fig. 5 in the new submission to make it easier to distinguish.

5. When comparing images (also arrows should be added to highlight some features) or gel data, authors may use quantitative methods to clearly tell the differences among the samples. It appears that several figures are missing controls. Sometime the study can be strengthened with time lines. The proinflammatory cytokines or related pathways may be measured to show some deeper mechanistic studies.

Reply: We appreciate your kind suggestion.

(1) We added quantitative analysis for the lung injury from HE stain sections, as shown in the newly added Fig. 1D and Fig. 5B of the new submission.

(2) We added control groups in Fig. 5A and 5C for the new submission.

(3) We added animal experiments with time lines and measured the proinflammatory cytokines in Fig. 1E.

(4) We completed additional experiments showing that LL-37 could inhibit the production and release of macrophage inflammatory factors induced by LPS and, thus, have anti-inflammatory effects by down-regulating the expression of inflammatory-associated NF-κB pathway signaling molecules. In this article, we intended to observe the direct anti-bacteria activity of LL-37.

6. Statistics: some figures have not described the sample sizes (Fig. 1, etc.). Many comparisons should be done with non-parametric methods, and methods should be mentioned in the figures.

Reply: Thank you for the correction, and we apologize for our carelessness. The methods for t-tests and ANOVAs are universal in our research article; therefore, we continued to utilize these methods. The Method section was modified and presented as below:

All statistical analyses were performed using GraphPad Prism 7 software. An unpaired t-test (two-tailed) was used for the comparison between two experimental groups. For experiments with more than two groups, one-way ANOVA was performed and, when required, followed by Turkey's test. A significance threshold was set at $p < 0.05$.

Two-way ANOVA was used in the grouped tables that were indicated in the legend. A significance threshold was set at $p < 0.05$.

7. Material and method: No information for mice and rat. Age? Weight? Gender? Provider? Protocol? Bacterial strains?

Reply: We apologize for not providing detailed information about mice.

(1) Animal: Female C57/B6 mice and wild type FVB mice (6-8 weeks) weighing 16-18 g were purchased from Shanghai SLAC laboratory Animal Co., Ltd. (China). LL-$37^{+/+}$ mice (FVB

background) were produced by the company of Cyagen Biosciences Inc. (China). Male Sprague-Dawley rats weighing 180–220 g each were purchased from Shanghai SLAC laboratory Animal Co., Ltd. (China). All mouse strains were housed in specific pathogen-free conditions within an animal care facility (Center of Laboratory Animal, Tongji University, Shanghai, China). All animal experiments were performed under the guidance and with approval from the Institutional Animal Care and Use Committee of Tongji University.

(2) *P. aeruginosa* (ATCC-BAA-47; strain HER-1018) and the *E. coli* strain DH5-α (ATCC-98489) were used in these experiments.

8. Are the authors sure the left and the right panel is the same picture in figure 6a?

Reply: We apologize for our carelessness. We have changed the image in the new submission.

9. What the blue color dye was used in all fluorescence images? Figure 2d and figure 4a need some separation to indicate the merger.

Reply: Thank you for the suggestion. The blue color dye was DAPI, as mentioned in the Method section. Fig. 4A was separated for the new submission. Figure 2D—changed and separated to indicate the merger—is shown below (Figure R2).

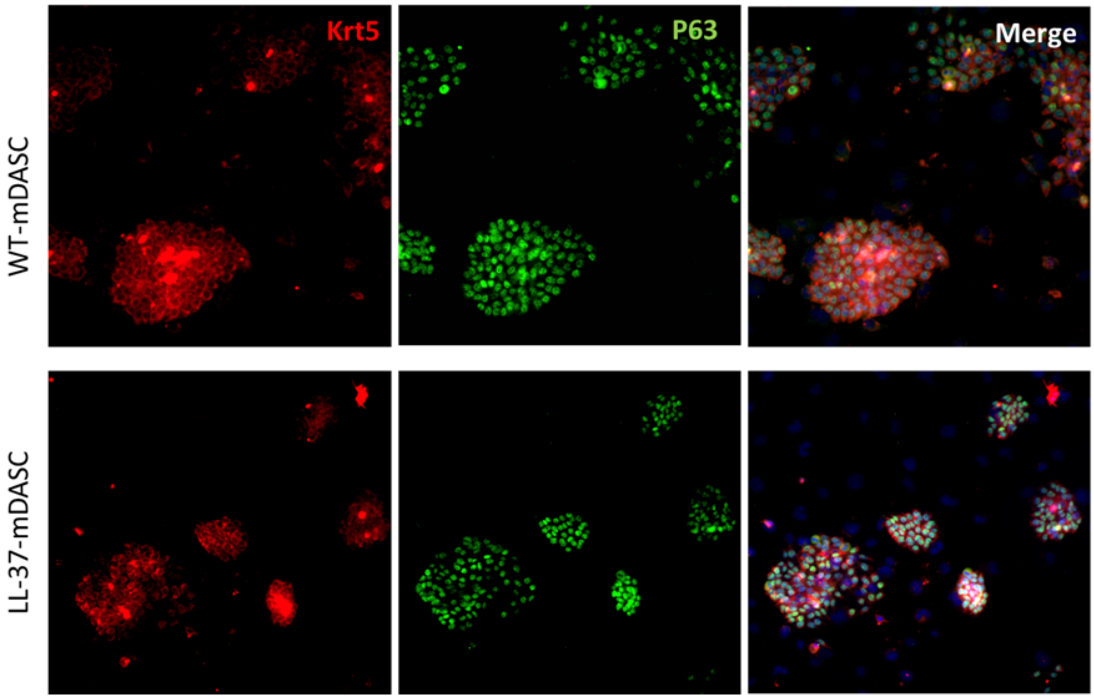

Figure R2. Anti-Krt5 (red) and anti-P63 (green) immunostaining of WT- and LL37-mDASC colonies. Scale bar, 40 µm.

10. Figure 2h, it is unclear which groups were infected. The line should begin from the second group.

Reply: Thank you for the proposal. We have relabeled the groups that were infected in the new submission.

11. Fig 5a is very poor quality and scale bar is needed.

Reply: Thanks for your suggestion. According to your comment, we changed the pictures and added some groups with scale bars in the revised manuscript.

12. Many grammatical errors in the manuscripts, i.e., missing "the" in the front of lung. Authors should take this seriously to improve the writing.

Reply: We thank the reviewer for the kind correction, and we apologize for the poor writing. Indeed, this was a grammatical error, and we have corrected it according to your suggestion.

Minor concerns:
1. Partial writing is not standardized, e.g., il-1β, TNF-α.
2. Some full names are missing like "pcc".
3. Western blotting is poor quality.
4. More discussion can be added for figure 1e.

Reply: We greatly appreciate your professional review work on our manuscript. Minor concerns mentioned above were corrected in our new submission. The manuscript has certainly benefited from these insightful revision suggestions. We look forward to working with you to move this manuscript closer to publication in EMBO Molecular Medicine.

Response to Reviewer 2
(Reviewer's comments are paraphrased and appear in *Italic Calibri Light*)

The manuscript by Zhou et al asks interesting questions about the impact of overexpression of the antimicrobial host defence peptide LL-37 in pulmonary infection, both using a transgenic mouse model and stem cell based therapeutic platform.

Overall the paper addresses interesting questions, but I unfortunately have multiple concerns about the approach to the models used, depth to which these are interrogated, the interpretation of the data that are provided, the lack of mechanistic evaluation, and the viability of such an approach as a future human therapeutic strategy. In addition, the manuscript is not sufficiently well referenced. The most important concerns are detailed below to try to help the authors to tackle this potentially interest project.

1. The introduction makes far too many important statements that are not backed up by references and/or uses references that are not directly relevant to the preceding statement. This needs to be addressed throughout. In particular, the manuscript does not refer to key publications relevant to this specific research (e.g. Bals et al 1999, JCI, 103(8): 1113-7; Beaumont et al 2014 PLOS One 9(6) e99029; Yu et al 2010 J. Immunol 185(2):1142-1149; Yang et al 2000 JExpMed 192(7):1069-74; Schaller Bals et al 2002 Am J Respir Crit Care Med 165(7): 992-995 and others). This is particularly important because these papers, together with Kovach et al 2012 J.Immunol, Bals 1999 I&I (both referenced but not fully discussed in context) and others, have led the field to view LL-37 (particularly in the context of Pseudomonas infection of the lung) to be an inducible, neutrophil chemotactic and inflammation enhancing, protective antimicrobial in Pseudmonas lung infection. It is critical that the data in this paper, which does not fit this concept, is properly discussed in that context.

Reply: We thank you very much for your kind suggestion and the introduction of new the submission was corrected to make it more understanding and convincing. hCAP-18/LL-37 (LL-37) is currently the only antimicrobial peptide of the cathelicidin family identified in humans that is an indispensable innate immune system component and represents the first line of defense against many invading pathogens. LL-37 plays various immunomodulatory roles in response to different kinds of pathogens invasion. LL-37 can directly kill bacterial, which was proven in previous studies (D. Vandamme, Cell Immunol 280, 22-35 (2012)). On the one hand, LL-37 plays a key role in inhibiting the formation of PAO1 biofilm at lower concentrations and directly degrades biofilms at higher concentrations (S. N. Dean, Front Microbiol 2, 128 (2011)); conversely, one study (Sadek M. Alalwani, et al. The antimicrobial peptide LL-37 modulates the inflammatory and host defense response of human neutrophils. Eur J Immunol. 2010 Apr;40(4):1118-26,) reported that LL-37 could reduce the secretion of TNF-α, IL-6, and IL-1βafter the stimulation of LPS. It is reported that LL-37 was highly expressed and had potent anti-infective and anti-inflammatory potential (Currie. Journal of immunology 196, 2699-2710, Nijnik, Current opinion in hematology 16, 41-47 (2009)). Previously, LL-37 treatment was shown to reduce LPS-induced nitric oxide release from the rat aorta (Ciornei, C. D., A. Egesten, and M. Bodelsson. 2003. Effects of human cathelicidin antimicrobial peptide LL-37 on lipopolysaccharide-induced nitric oxide release from rat aorta in vitro. Acta Anaesthesiol. Scand. 47:213–220) and protect mice from LPS lethality (Larrick, J. W.,

M. Hirata, R. F. Balint, J. Lee, J. Zhong, and S. C. Wright. 1995. Human CAP18: a novel antimicrobial lipopolysaccharide-binding protein. Infect. Immun. 63:1291–1297). In our study, LL-37-DASC was important in resisting PAO1 infection and reducing the secretion of cytokines including TNF-α and IL-6, which is consistent with former studies.

2. *The introduction is focused on the modulatory properties of LL-37, when the data presented (particularly in the later parts of the manuscript) probably relate more to the directly microbicidal properties of this peptide.*

Reply: Thank you for the reminder. In the introduction, we first explained that bacterial infection of the lungs is a major threat to human health and that cationic antimicrobial peptides have anti-infection and anti-inflammatory effects. However, the application of LL37 in clinical settings has been limited by different elements. For the entirety of the article, we have focused on the direct microbicidal properties of LL-37. In accordance with your reminder, we have updated sentences in the introduction and hope to explore the modulatory properties of LL-37 in our future work.

3. *Throughout the manuscript, no clear attention is given to the distinction between hCAP-18 (the pre-propeptide product of the CAMP gene) and its active predominant cleavage product LL-37. This distinction is critical. It appears that the new transgenic mouse overexpresses hCAP-18 constitutively, but how much is cleaved to LL-37? Is it cleaved without inflammation (usually neutrophil Pr3 is required) or are neutrophils required? Is the level altered in inflammation? How much LL-37 is produced and precisely where and by which cells? How does it compare to levels of endogenous cathelicidin, does it affect this, and what might be the interplay between these. This information and more is absolutely necessary to understand the model.*

Reply: We apologize for the improper wording. The human cationic antimicrobial protein of 18 kDa (hCAP-18) belongs to the cathelicidins; the precursor to LL37 has no antibacterial activity and is released mainly from activated neutrophil granulocytes (Larrick, J. W., M. Hirata, R. F. Balint, J. Lee, J. Zhong, and S. C. Wright. 1995. Human CAP18: a novel antimicrobial lipopolysaccharide-binding protein. Infect. Immun. 63:1291–1297. Sørensen, O., K. Arnljots, J. B. Cowland, D. F. Bainton, and N. Borregaard.1997. The human antibacterial cathelicidin, hCAP-18, is synthesized in myelocytes and metamyelocytes and localized to specific granules in neutrophils. Blood 90:2796–2803). After release, the 37-amino-acid α-helical C-terminal end is cleaved off, forming the functional antimicrobial peptide LL-37 (Gudmundsson, G. H., B. Agerberth, J. Odeberg, T. Bergman, B. Olsson, and R. Salcedo. 1996. The human gene FALL39 and processing of the cathelin precursor to the antibacterial peptide LL-37 in granulocytes. Eur. J. Biochem. 238:325–332). The process of cleaving hCAP18 is needed to activate neutrophils.

We infected FVB mice (control) and LL37$^{+/+}$ mice with PAO1 (5×10^6 CFU) for 24 h, and lung tissues were collected for Western blot (results shown below). LL37 and hCAP-18 were not detected in the lung of FVB mice, while LL37 was expressed weakly compared to hCAP-18 (Figure R3), which is likely caused by technical difficulties stemming from the small molecular weight of LL37. We hope to explore the relationship of LL37 production with inflammation in future work.

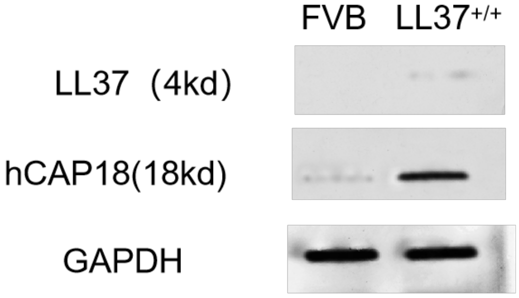

Figure R3. Immunoblotting for LL37 (4kd) and hCAP18 (18kd) in mouse lung.

The RNA-Seq data shows increased endogenous cathelicidian (CAMP) in FVB mice post-infection that did not occur in LL37$^{+/+}$ mice (Figure R4), suggesting that LL-37 expression could play an essential role in antibacterial clearance for CAMP replacement in early stages.

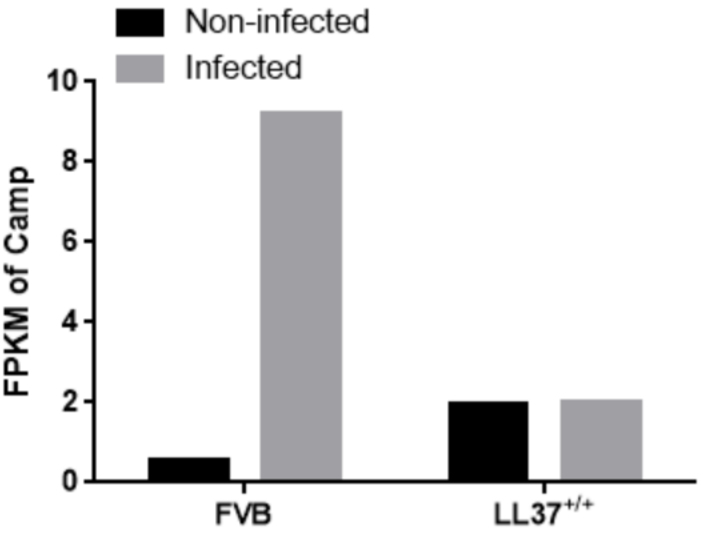

Figure R4. Relative expression of Camp in RNA-seq data.

4. *The hCAP-18-overexpressing mice are an interesting novel line perhaps worthy of paper on their own, examining their phenotypes with and without inflammation and infection. All we are told is that they have "no noticeable phenotype". Given the existing literature, constitutive expression of LL-37 would be expected to have effects. As it stands, this is inadequate, and interpretation of the data presented requires detailed description of the model and what characterisation was performed. Given some published data it is possible that LL-37 may not have a large effect until inflammation is induced, but this needs to be characterised with a mechanistic evaluation.*

Reply: Thanks for your positive comments. We apologize for not providing enough information about the phenotype. We have performed RNA-Seq on entire lung tissues from uninfected FVB and $LL37^{+/+}$ mice for mechanistic evaluation, as shown in Fig. 1H. Accordingly, the figures have been updated in new submission.

5. *The murine lung PAO1 challenge is not detailed with enough clarity in the methodology. It appears that the mice were given 5×10E8 cfu of PAO1 directly into the lung. This is a very large inoculum and it is surprising, in my experience, that the mice survived this. As far as I can tell the only timepoint assessed was 2 days later, by which time nearly the entire inoculum had been cleared in all the mice (only 500 - 1000 cfu / g of lung left in figure 1). It is very hard to determine the biological significance (albeit it is statistically significant) of having 500 rather than 1000 cfu of PAO1 left after 48 hours. Studying earlier timepoints will be essential to understand the dynamics of this effect, particularly when the histology shows such a dramatic difference that does not correlate well with the minimal cfu differences. Maybe this is because the LL-37 is having a predominantly modulatory effect on inflammation, but that needs to be clearly examined mechanistically. What are the difference in cellular responses and cytokines/chemokines over a time course in response to infection?*

Reply: We appreciate your careful checks, and we apologize for our carelessness. We used 5×10^6 CFU of PAO1 per mouse. The protocol for the animal infection model is the following: we prepared 5×10^8 CFU/ml of PAO1 as the initial concentration of bacteria solution. Before infecting mice, 10 ul of the initial bacteria solution was diluted to 30 ul with PBS that was instilled into mouse. We added the survival data as below Figure R1. Mice began to die at 15 h after PAO1 (5×10^8 CFU) administration, and all mice died at 30 h. Mice did not survive to 45 h post-infection when mice were injured by PAO1 (5×10^7 CFU). Mice were alive after 72 h post-infection of PAO1 administration with doses of 5×10^6 CFU or 5×10^5 CFU per mouse. In this study we chose the dose of 5×10^6 CFU for each mouse for the infection model. Since the Beaumont study demonstrated the capacity of LL-37 to enhance pulmonary bacterial clearance at 6 h and 24 h (Beaumont, P. E. et al. Cathelicidin host defence peptide augments clearance of pulmonary Pseudomonas aeruginosa infection by its influence on neutrophil function in vivo. PloS one 2014. 9, e99029, doi:10.1371/journal.pone.0099029), we observed the bacterial clearance capacity of LL-37 at 48 h.

© European Molecular Biology Organization

Our mouse model is supported in several papers, such as Yu FS et al 2010 J Immunol. 185(2):1142-9 ; Torres IM, et al 2017, Am J Physiol Lung Cell Mol Physiol. 314(2):L225-L235 and David Nobuhiro Douda et al 2011 J Immunol 187 (4) 1856-1865.

We have added animal experiments with time lines to study the effect of LL-37 at earlier time points, and the chemokines in response to infection have been examined over a time course in Fig. 1E of the new submission. Accordingly, the figures have been updated in the new submission. The secretion of TNF-α, IL-6, and IL-1βafter infection were reduced in LL-37$^{+/+}$ mice.

6. The histology needs to be backed up by full quantification and scoring of multiple animals throughout the paper, not just some representative images.

Reply: Your suggestion is important to us. Indeed, quantitative analyses would provide better understanding, and, thus, we added quantitative analysis of lung injury from the HE stain sections. Accordingly, the figures have been updated in the new submission (Figure 1D and Figure5).

7. The transcriptomics is potentially fascinating but doesn't add much as presented. It shows very marked differences - unlike the cfu data. Why this discrepancy? It does present an opportunity to develop this into a mechanistic evaluation in the future though, together with immune effector cellular response profiling of these mice both with and without infection. However, it does not fit well with published data (as detailed above), which needs to be carefully examined and discussed.

Reply: Thank you for the positive comments. In accordance with the reviewer's reminder, we supplemented more RNA-Seq in Fig. 1 of the resubmission, which we think is consistent with the published data mention above.

8. At the end of the first results section the authors state "The data indicate that constitutive expression of LL-37 in mouse lung can protect the lung from bacterial infection and thereafter inflammation". This conclusion fits previously published data, but the data presented do not fit with existing knowledge and no alternative mechanism is presented. Just for example, could this be a rapid early microbicidal effect that minimises the inflammatory response required before there is much inflammation for LL-37 to enhance? Is the BALF itself directly microbicidal?

Reply: Thank you for the suggestion. In the newly added Fig. 1E of the new submission, the expression of IL-6 and IL-1β was lower in LL37$^{+/+}$ mice than in FVB mice after PAO1 infection for 6 h, indicating that LL37 significantly reduced inflammation levels. In accordance with your suggestion, mechanistic evaluation can interpret this microbicidal effect—a significant concept for further research.

9. The genetic basis for the LL-37 expression by mDASC is not clear enough. Is this hCAP-18 expression (and if so, how is it cleaved in cellular systems that do not produce the required proteases), or just the LL-37? Is it constitutive and can levels be affected by inflammation? Where in the cells is it localised and how is it release if it is just LL-37 (not the full pre-propeptide hCAP-18 being expressed)? Fig 2b needs to state what size this band is (is it hCAP-18 or LL-37) and show any other bands. Figure 2a needs to show controls (e.g. no primary antibody and isotype control + secondary) to check specificity.

Reply: We apologize for the improper wording. The gene engineered for expression in either transgenic mice or mDASCs was hCAP-18. However, cleaved LL-37 was detected in the transgenic mice, as shown above.

In our study, we assessed the bacterial clearance abilities of cells and corresponding cellular supernatant. PAO1 growth was significantly inhibited by the supernatant from LL37-mDASCs but not wild-type ones. Although the detailed mechanism remains unclear, all our data show that hCAP-18/LL-37 possess bio-activity in cellular systems and inhibit PAO1 growth. The control (no primary antibody plus secondary) in Fig. 2A is shown below (Figure R5).

© European Molecular Biology Organization

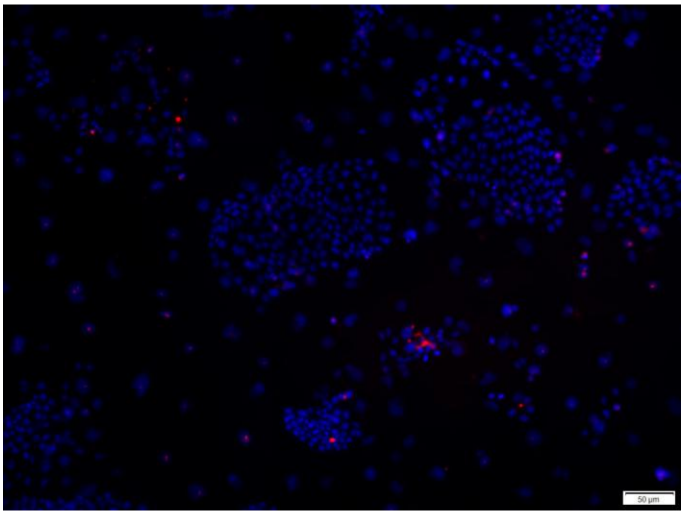

Figure R5. Immunofluorescence staining of mDASCs without primary antibody.
(Corresponds to Fig. 2A in the new submission)

10. Is Fig 2h just looking at inhibition of proliferation (also Fig 6g)? What was the starting inoculum? Is there no bacterial killing? This needs to be much clearer. If so, how is this compatible with LL-37 being an effective antimicrobial? There is far more growth in the WT m-DASC than the controls - why is that? What statistical test was used? Is LL-37mDASC significantly different than control? Why was the anti-LL-37 antibody only used against E. coli when all the rest of the paper deals principally with PAO1?

Reply: Thank you for the kind comments. Many metabolites were created in the conditioned medium during cell culture including carbohydrates, amino acids, and lipids that can promote bacterial proliferation. In the controls for Fig. 2H and 6G, there was only PAO1 and medium (starting inoculum: culture medium without FBS and antibiotics) without any cells. So, this explains the CFU increase of other groups compared to control group.

A one-way ANOVA was performed followed by Turkey's test, when required. A significance threshold was set at $p < 0.05$. In Fig. 2G, LL-37-mDASCs are not significantly different than control. In addition, we have added the data for the anti-LL-37 antibody in the PAO1 experiment; the result is shown in the newly added Fig. 2I of the new submission.

11. In Fig 3 more detail on what the engraftment ratio is and how it is calculated is required, and the histology needs to make it clearer where these cells are engrafted. The statement that "there is no incorporation of GFP+ cells in uninjured lung" should be demonstrated.

Reply: (1) The lungs were assumed to be a cube—if the area ratio of GFP fluorescence in the lung tissue was A, the transplantation volume ratio was calculated as $R=(\sqrt{A})^3$.

(2) Thank you for the reminder. The corresponding data for the statement "there is no incorporation of GFP+ cells in uninjured lung" is shown in Fig. R6 below.

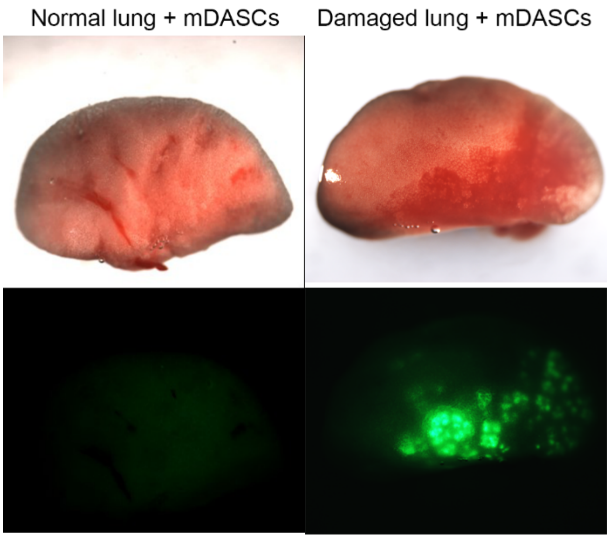

Figure R6. The incorporation of GFP+ cells in normal or bleomycin-damaged lungs 7 days post-transplantation.

12. Again the infection studies end up comparing tiny residual cfus (as little as 50 - 150 cfu /ml in panel e) at a single late time point, which is of questionable biological significance and does not provide mechanistic evaluation. In addition, the magnitude of difference for PAO1 in panel d is greater here than when the whole lung is over expressing LL-37 in figure 1. How is that explained?

Reply: Thanks for your suggestion.

(3) (1) Since the Beaumont study demonstrated the capacity of LL-37 to enhance pulmonary bacterial clearance at 6 h and 24 h (Beaumont, P. E. et al. Cathelicidin host defence peptide augments clearance of pulmonary Pseudomonas aeruginosa infection by its influence on neutrophil function in vivo. PloS one 2014. 9, e99029, doi:10.1371/journal.pone.0099029), we observed the bacterial clearance capacity of LL-37 at 48 h. Also, we agree that mechanistic evaluation is an important area, which will be significant concept of future research.

(2) In Fig. 4D, we selected the lobes with GFP+ where the mixtures (i.e. PAO1 with cells) were deemed to distribute; so, the CFU of these lobes was higher than the average of the whole lung.

13. Although it is good to get some cytokine evaluation here, other time points should be looked at and also cellular responses. Also, surely the most important statistical evaluation to make is LL-37-lung vs control-lung. Is that significant? What is happening to native cathelicidin expression in this model and how does that impact?

Reply: (1) We have supplemented the animal experiments with time lines and measured the proinflammatory cytokines, as shown in Fig. 1E of the new submission. The statistical results and details are shown in the figures and legends, respectively.

(2) The RNA-Seq data showed that native cathelicidian (CAMP) of LL-37-Lung was less than that of WT-Lung after PAO1 infection (Figure R7), which is likely caused by LL-37 playing a key role in inhibiting the bacterial growth; thus, the effect of endogenous CAMP is replaced by LL-37.

© European Molecular Biology Organization

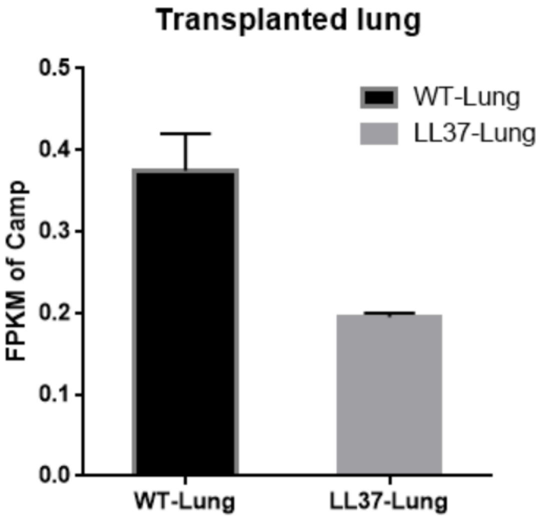

Figure R7. Relative CAMP RNA-Seq expression from transplanted and infected mice.

14. In Fig 6b it is unclear whether this could distinguish between any native expression of hCAP-18/LL-37 and expression of the transgene. Might the former be upregulated in response to the infectious stimuli and if so, what impact might it have? In Fig 6f the LL-37 (red) does not seem to co-localise with the GFP. Why is this? What is the control is Fig 6g? I'm not entirely clear. The values of the studies in this figure are not totally evident.

Reply: Thanks for your suggestions.

(1) In Fig. 6B, we tested the relative-expression of hCAP-18/LL-37 in LL37-hDASCs compared with WT-hDASCs, which was 1000 fold for both groups. Compared to this difference, the native expression is essentially negligible. Besides, the CAMP expression in mice increased only 10 times when infected, as mentioned above (Figure R7). Based on this, we deduce that the impact of native hCAP-18/LL-37 expression does not play a key role in our results.

(2) GFP may be lost in the process of cell proliferation.

(3) We have re-labelled the group in Fig. 6G. The first control was just medium (cultured medium without antibiotics and FBS) without bacteria, and the second control was medium cultured with bacteria.

15. More effort should be made to justify why an approach such as that presented here would be preferable to much simpler strategies, such as direct delivery of LL-37 (or related peptides) or the use of cathelicidin-inducing drugs (already in use in human trails), which could have much lesser long-term consequences and risks. Given published data one might expect that damage areas having constitutive expression of LL-37 might have enhanced inflammation and neutrophil recruitment. This could be harmful if not controlled during resolution. Is this a viable therapeutic approach?

Reply: (1) Research has reported the potent anti-infective potential of LL37. However, the direct application of LL37 in clinical settings has been limited by many disadvantages, including a short *in vivo* half-life, a high cost of synthesis *in vitro*, and hemolytic toxicity by intravenous injection. Therefore, the development of a system to achieve local, long-term LL37 release is highly desirable for pulmonary infection therapy. The aim of our research is to discover a carrier with LL-37 to the sites of pulmonary infection for treatment and regeneration.

(2) Indeed, it has been reported that LL37 enhances inflammation and neutrophil recruitment and is beneficial to clearance of bacteria, virus, and fungi (R. I. Lehrer, Curr Opin Hematol 9, 18-22 2002, Ooi EH, Am J Rhinol. 2007 21:367–72). We acknowledge the reviewer's points above; however, we hold the view that there is a balance between the anti-inflammatory and proinflammatory effects of LL-37. When pathogens invaded, the early pathogen infection was eliminated by LL37, and the

inflammatory response caused by infection decreased. Besides, we have newly provided CD68—a protein highly expressed by macrophages—immune-histochemical staining of lungs with different treatments in Fig. 5C and 5D of the new submission. These figures show that administration of LL37-mDASCs significantly decreased the amount of CD68 after PAO1 infection. We hope to explore the anti-inflammatory and pro-inflammatory effects of LL37 in our future work.

16. Statistical evaluation seems to be by ANOVA followed by T-test Is this the correct post-test approach for the one-way ANOVAs?

Reply: Thank you for the correction. The updated Method section for statistical analysis is shown as below and has been simultaneously corrected in the new submission.

All statistical analyses were performed using GraphPad Prism 7 software. An unpaired t-test (two-tailed) was used for the comparison between two experimental groups. For experiments with more than two groups, one-way ANOVA was performed and, when required, followed by Turkey's or Sidark's test. A significance threshold was set at $p < 0.05$.
Two-way ANOVA was used in the grouped tables that were indicated in the legend. A significance threshold was set at $p < 0.05$.

We really appreciate the editors' and reviewers' help and patience concerning our study. We have revised the manuscript extensively according to the reviewers' comments. We appreciate your immense help, and we welcome further suggestions for the study. We hope to have our article considered for publication in your journal. Should there been any other corrections we could make, please feel free to contact us.

2nd Editorial Decision 5 June 2019

Thank you for the resubmission of your manuscript to EMBO Molecular Medicine. We have now received feedback from the two reviewers who had initially reviewed the first version of your manuscript. As you will see from the reports below, while referee #1 is satisfied with the revisions and supports publication of the manuscript, referee #2 still has serious concerns (as we do) regarding the adequacy of the model and the clinical relevance of the data. This referee also notes that several of the points that were raised in the first review of the manuscript were not satisfactorily addressed.

As EMBO Press encourages a single round of revisions only, and given the extent of referee #2's concerns, we would normally reject the manuscript at this stage. However, after discussion with our editor in chief Philippe Sansonetti, we decided to exceptionally allow a second round of revisions. Please be aware that this will be the last chance for you to address all the points raised by the referee, and that acceptance or rejection of the manuscript will depend on the completeness of your responses included in the next, final version of the manuscript. For this reason, and to save you from any frustrations in the end, I would strongly advise against returning an incomplete revision.

I look forward to receiving your revised manuscript.

***** Reviewer's comments *****

Referee #1 (Comments on Novelty/Model System for Author):

Very nice paper.

Referee #1 (Remarks for Author):

Have addressed the concerns.

Referee #2 (Comments on Novelty/Model System for Author):

The transgenic mouse is not well enough characterised, and earlier timepoints in in vivo models must be studied for bacterial clearance to understand the mechanisms at play. Clarity in hCAP-18 to LL-37 processing are required and discrepancies exist around growth inhibition being shown in vitro vs microbicidal roles proposed in vivo.

Referee #2 (Remarks for Author):

The authors have addressed some of the questions raised and have improved the manuscript somewhat. However, many of the reviewers' comments have not been fully addressed; in some cases important responses have been responded to in the rebuttal but not addressed in the revised manuscript, in other instances the responses are insufficient (e.g. not showing critical early timepoint bacterial clearance data because another published study using a totally different model had shown that LL-37 could clear at those timepoints), in still others parts the points raised are really not tackled in a meaningful way at all. Thus, even although parts of this research do remain interesting, in my opinion the data simply do not provide strong enough mechanistic evaluation and do not support the conclusions made to explain the interesting observations, where they exist. I have detailed some of my remaining main concerns, but please note that these are not exhaustive, and there are additional points made in my initial review that remain outstanding. I also remain unconvinced as to why an approach such as that presented here would be preferable to much simpler strategies, such as direct delivery of LL-37 (or related peptides) or the use of cathelicidin-inducing drugs (already in use in human trails), which could have much lesser long-term consequences re the toxicity issues the authors themselves mention and consequent risks.

Page 5: Lines 8 -10 and Fig 1: The authors now state that a lower, more appropriate dose of PAO1 was used and explain that the prior stated concentration was an error. They also state that lung CFU were examined at 6, 24 and 48h, but they still only provide data for 48 hours, which does nothing to address my previously stated concern, namely that it is very hard to determine the biological significance (albeit it is statistically significant) of having 500 rather than 1000 cfu of PAO1 left after 48 hours. This is not strong enough to demonstrate altered clearance dynamics. Although new figures C-E are useful to show lower levels of inflammation at early timepoints, bacterial clearance is crucial to understand what is happening, irrespective of what other studies (e.g. Beaumont et al) have shown in a very different model system (this applies later in the study too).

Fig 1: I still find Figure 1 inadequate as a characterisation of this new transgenic mouse. Given the existing literature, constitutive expression of LL-37 would be expected to have many effects. Whereas the authors previous stated that these mice have "no noticeable phenotype", their RNA seq data shows lots of changes prior to infection. As a proof of principle that constitutive expression of LL-37 might be a good therapeutic idea, this is not sufficient. The data do not mechanistically determine what is happening to explain possible increase bacterial clearance and lower inflammation after infection. Despite my previous comments, the paper still does not even demonstrate whether this mouse actually has functional LL-37 present - the data in the response letter is not in the paper and doesn't convincingly show LL-37 is being processed at a meaningful level from hCAP-18, making the whole figure hard to interpret.

Fig 2: The authors say that LL-37 expression is shown, but the band in Fig 2c is labelled as 18kDa, so it must be the precursor hCAP-18, not LL-37. RTPCR in Fig 2b detects CAMP RNA for hCAP-18, and it is not clear whether the antibody can distinguish between LL-37 and hCAP-18 in the immunofluorescence (this applies to later figures too). This means that this whole system has been evaluated in the presence of the inactive precursor hCAP-18 (unless a suitable protease is present to cleave this to LL-37 - and that would need to be demonstrated), and therefore, I think that all the questions apparently being addressed, about what the functional consequences of LL-37 exposure are, are actually untested.

Fig 2 i & j: as per my previous comments, it seems that this is only a growth inhibition effect of the cathelicidin expression, but the authors claim this shows "a broad antimicrobial spectrum" (top P.7). That is misleading and does not therefore explain the claim of enhanced PAO1 killing in the murine models. Furthermore, the effect on PAO1 appears to be unrelated to LL-37 (with no significant

impact of the anti-LL-37 antibody for the relevant bacteria). But then, there is not definitive data to show that the hCAP-18 has been processed to LL-37 in this system anyway.

Fig 4: My concerns here have not been addressed - again the infection studies end up comparing tiny residual cfus (as little as 50 - 150 cfu /ml) at a single late time point, which is of questionable biological significance and does not provide mechanistic evaluation. In addition, the magnitude of difference for PAO1 in panel d is greater here than when the whole lung is over expressing LL-37 in figure 1. How is that explained?

Additional minor points:
Page 4: Lines 22 - 23: The statement "However, the LL-37 peptide has a short half-life in vivo due to lability to proteases, limiting its clinical application." should be referenced
Page 4: Lines 23 - 24: The statement "Furthermore, the peptide needs to be delivered topically, rather than systemically, to avoid potential toxicity." should be referenced to specific toxicity concerns. Also, how does the kind of therapy proposed here avoid that potential toxicity? I would have thought that constitute pulmonary LL-37 expression would be very harmful. This should be discussed in the manuscript.
Page 5: Lines 3 - 4: The authors should also reference Schaller Bals et al 2002 Am J Respir Crit Care Med 165(7):992-995, and in introducing the concept that LL-37 expression can protect against Pseudomonas lung infection the authors should reference the prior literature early e.g. Bals et al 1999, JCI, 103(8): 1113-7; Beaumont et al 2014 PLOS One 9(6) e99029; Yu et al 2010 J. Immunol 185(2):1142-1149; Kovach et al 2012 J.Immunol; Bals 1999 I&I

2nd Revision - authors' response 4 September 2019

The following is our point-to-point response to the comments of referees. The reviewer' comments are italicized, whereas our replies are in normal type.

Response to Reviewer #1

Referee #1:

Very nice paper.

Have addressed the concerns.

We thank the reviewer #1 for his/her kindness.

Response to Reviewer #2

The transgenic mouse is not well enough characterised, and earlier timepoints in in vivo models must be studied for bacterial clearance to understand the mechanisms at play. Clarity in hCAP-18 to LL-37 processing are required and discrepancies exist around growth inhibition being shown in vitro vs microbicidal roles proposed in vivo.

1. *The authors have addressed some of the questions raised and have improved the manuscript somewhat. However, many of the reviewers' comments have not been fully addressed; in some case important responses have been responded to in the rebuttal but not addressed in the revised manuscript, in other instances the responses are insufficient (e.g. not showing critical early timepoint bacterial clearance data because another published study using a totally different model had shown that LL-37 could clear at those timepoints), in still others parts the points raised are really not tackled in a meaningful way at all. Thus, even although parts of this research do remain interesting, in my opinion the data simply do not provide strong enough mechanistic evaluation and do not support the conclusions made to explain the interesting observations, where they exist. I have detailed some of my remaining main concerns, but please note that these are not exhaustive, and there are additional points made in my initial review that remain outstanding. I also remain unconvinced as to why an approach such as that presented here would be preferable to much simpler strategies, such as direct delivery of LL-37 (or related*

peptides) or the use of cathelicidin-inducing drugs (already in use in human trails), which could have much lesser long-term consequences re the toxicity issues the authors themselves mention and consequent risks.

Reply：

First of all, we would apologize for the previous inadequate revision, and would like to express our sincere respect to the reviewer for his/her rigorous scientific attitude to research.

Secondly, as the reviewer suggested, in the revised manuscript, we further characterized the transgenic mice from multiple aspects and expanded one figures to two. We analyzed their lung morphology, transcriptomic profile and response to bacterial infection. Also we completed the study of early time point bacterial clearance data, which was shown in Fig.1D in the revision manuscript (lists as follow named Fig. R1 for your convenience), including the data of 6h, 24h, 48h. After the infection with PAO1 for different time points, LL-37 transgenic mice showed significantly lower levels of bacteria in the lung homogenates, compared to WT mice.

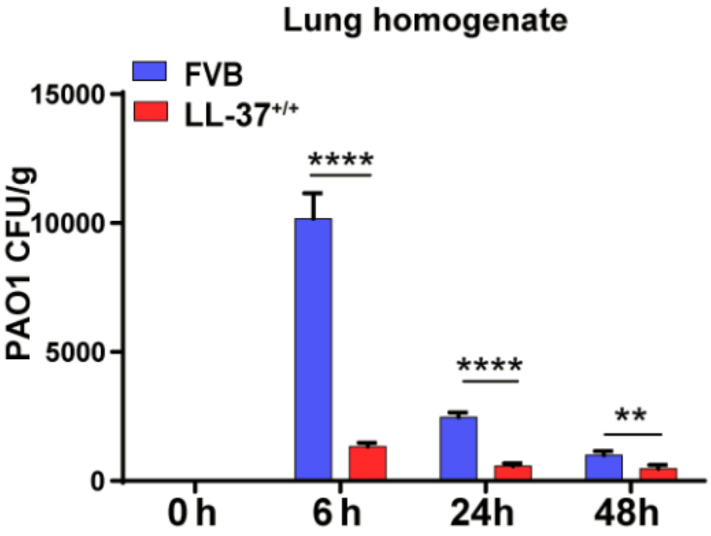

Fig R1. The CFU of PAO1 in lungs from indicated mice after PAO1 infection

Thirdly, the anti-microbial and immune modulatory mechanism of LL37 is very complicated. We agreed that dissecting the mechanism is important and could be our future project for the next several years to aim at. Now our major target of current work is to developing a new technology that hope to be an ideal candidate for the potential therapy for multidrug resistant bacteria infection and infection-related lung injury. For this reason, we could hardly address too much detailed mechanism in this manuscript. We hope we can exchange the progress about this in the future.

Fourthly, we agreed with the reviewer that other conventional strategies such as direct delivery of LL-37 or the use of cathelicidin-inducing drugs should be effective as well. However, here we are proposing an alternative novel strategy which has its own advantages: 1) The directly delivered LL37 could go to the circulation or anywhere in lung but the DASC distribution is limited to injured foci, which gives high local LL37 concentration while have minimal influence on healthy lobes. This is important to avoid side-effects; 2) The directly delivered LL37 could be cleaved or degraded by bacteria, and the constitutive expression of LL37 by DASC could supplement the lost peptide in time; 3) The DASCs were not only a carrying vehicle of LL37 but also regenerating new epithelium barrier to protect lung from further infection; 4) The long-term expression of LL37 does not seem to have vicious consequence as at least the LL37 transgenic mice were healthy in general. Altogether, considering all existing methods nowadays cannot efficiently control pulmonary infection-related death, a novel technology roadmap could give people new hope and of course all strategies have to be strictly tested on large animals first before they really go to clinical application.

2. *Page 5: Lines 8 -10 and Fig 1: The authors now state that a lower, more appropriate dose of PAO1 was used and explain that the prior stated concentration was an error. They also state that lung CFU were examined at 6, 24 and 48h, but they still only provide data for 48 hours, which does nothing to address my previously stated concern, namely that it is very hard to determine the biological significance (albeit it is statistically significant) of having 500 rather than 1000 cfu of PAO1 left after 48 hours. This is not strong enough to demonstrate altered clearance dynamics. Although new figures C-E are useful to show lower levels of inflammation at early timepoints, bacterial clearance is crucial to understand what is happening, irrespective*

of what other studies (e.g. Beaumont et al) have shown in a very different model system (this applies later in the study too).

Reply:

We thank the reviewer for the kind reminding and it was our mistake not to present the lung bacteria CFU data of all the time point in the previous version. To demonstrate altered clearance dynamics of LL37, we examined the lung CFU at 6, 24 and 48h, we found that LL-37 transgenic mice showed significantly lower levels of bacteria in the lung homogenates compared to WT mice at all time points. (Fig.R1 as above showed). These data demonstrate the capacity of LL-37 to enhance pulmonary bacterial clearance at early timepoints, which was consistent with lower levels of inflammation in lungs.

We also find Beaumont et.al.'s studies very interesting. They showed that expression of LL-37 lead to activation of neutrophil prior to infection, which was consistent with our new RNA-Seq analysis data in Figure 2B (same as Fig R2 below).

3. *Fig 1: I still find Figure 1 inadequate as a characterisation of this new transgenic mouse. Given the existing literature, constitutive expression of LL-37 would be expected to have many effects. Whereas the authors previous stated that these mice have "no noticeable phenotype," their RNA seq data shows lots of changes prior to infection. As a proof of principle that constitutive expression of LL-37 might be a good therapeutic idea, this is not sufficient. The data do not mechanistically determine what is happening to explain possible increase bacterial clearance and lower inflammation after infection. Despite my previous comments, the paper still does not even demonstrate whether this mouse actually has functional LL-37 present - the data in the response letter is not in the paper and doesn't convincingly show LL-37 is being processed at a meaningful level from hCAP-18, making the whole figure hard to interpret.*

Reply;

The reviewer mentioned three points in this paragraph. One is about the statement that the transgenic mice have "no noticeable phenotype". We apologize that these words we used were not accurate. What we would like to state was that the transgenic mice could grow and development normally as the wild type mice with no noticeable difference in general appearance and behavior. However, when we analyzed their transcriptomic profile of lung, even prior to infection, we did detect lots of changes that LL-37$^{+/+}$ gave rise to upregulation of multiple immune response related gene (Fig 2B, lists as follow named Fig. R2 for your convenience). In revised manuscript, we deleted the ambiguous statement.

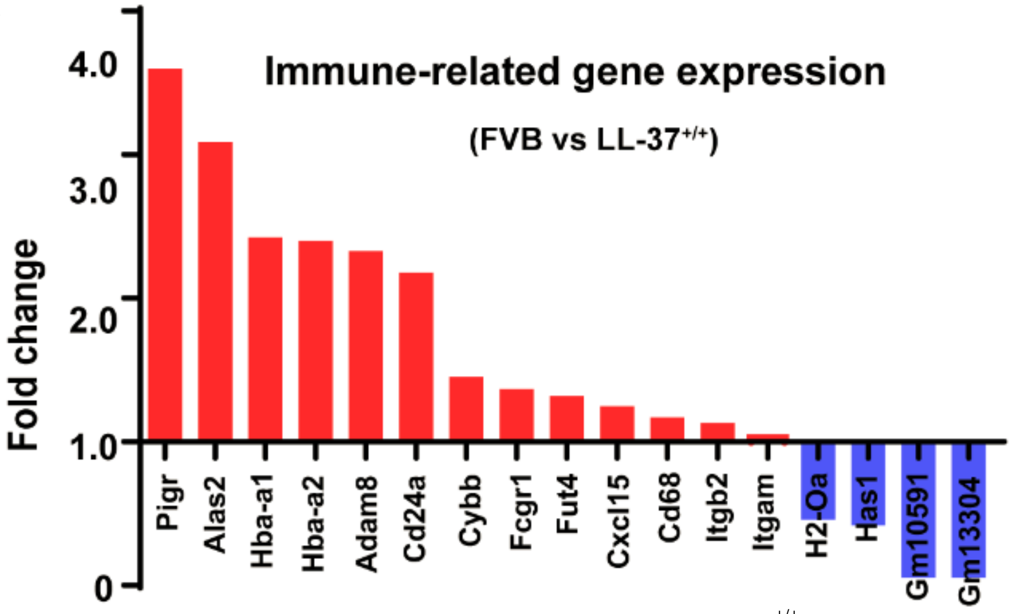

Fig R2. Histogram of selected differentially expressed genes of LL-37$^{+/+}$ mouse lung versus wild type FVB mouse lung prior to infection. Blue indicated genes up-regulated in wild type FVB mouse lungs, red indicated genes up-regulated in LL-37$^{+/+}$ mouse lungs.

The second point is about mechanistic evaluation. As we discussed above, the key point we want to highlight in our study is the new "proof-of-concept" technology and method, which has potential in clinical practice for pulmonary infection and injury treatment. Therefore, the mechanistic evaluation occupies little space in our manuscript. However, we are very grateful for the reviewer's suggestion, and the research on mechanism will be our focus in the future. We also look forward to sending to our future research to EMBO MM for review.

The third point is detection of functional LL37. Normally the expressed 18-KD hCAP-18 precursor needs to be processed into the 4-KD LL-37 to have biological functions. So we collected the BALF from wild-type FVB and LL-37 transgenic mice and passed them through the 10KD-centrifugal filter devices to concentrate the proteins with low molecular weight. The expression of 4KD LL-37 in transgenic mice was confirmed in the low-molecular-weight ultrafiltrate (Fig.1B, same as Fig.R3). When the BALF ultrafiltrate from LL-37 transgenic mice were used to grow PAO1 in vitro, it demonstrated much stronger bacteriostatic function than wild-type FVB ones. In contrast, the high-molecular-weight retentate had little bacteria inhibitory effect (Fig 1C, same as Fig.R4). This is the evidence showing that LL37 can be processed to be functional.

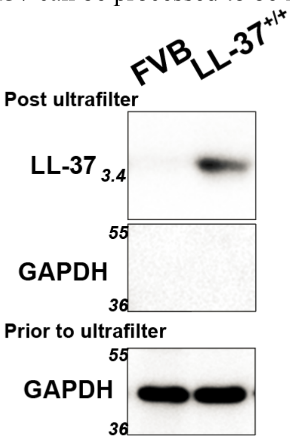

Figure R3. Detection of LL-37 (4KDa) expression in the indicated mouse by Western blotting. Prior to loading, samples were centrifuged to pass through 10 kD ultrafiltration membranes and the equal amount of ultrafiltrate (19 µg/lane) were subject to immunoblotting. High-molecular-weight proteins (GAPDH) were not detected in ultrafiltrate.

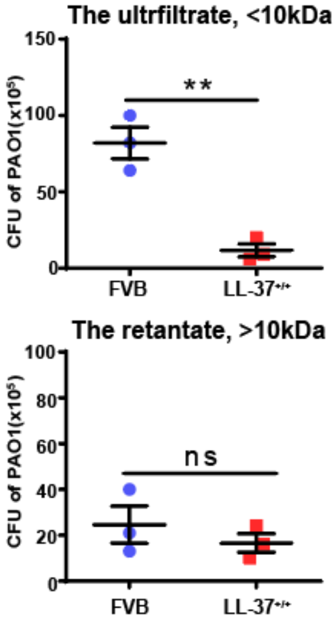

Figure R4. The growth of PAO1 was inhibited by less than 10 kD compositions of LL-37$^{+/+}$ mice BALF. BALF samples were separated into two different compositions, the ultrafiltrate (top panel) and the retentate (bottom panel).

4. *Fig 2: The authors say that LL-37 expression is shown, but the band in Fig 2c is labelled as 18kDa, so it must be the precursor hCAP-18, not LL-37. RTPCR in Fig 2b detects CAMP RNA for hCAP-18, and it is not clear whether the antibody can distinguish between LL-37 and hCAP-18 in the immunofluorescence (this applies to later figures too). This means that this whole system has been evaluated in the presence of the inactive precursor hCAP-18 (unless a suitable protease is present to cleave this to LL-37 - and that would need to be demonstrated), and therefore, I think that all the questions apparently being addressed, about what the functional consequences of LL-37 exposure are, are actually untested.*

Reply:
We thank the reviewer for the kind suggestion. It was previously reported that hCAP-18 is processed to the antimicrobial peptide LL-37 by extracellular cleavage with proteinase 3 encoded by PRTN3 gene (Sorensen, Blood, 2001). The PRTN3 gene is conserved in Rhesus monkey, cow, mouse, rat, mosquito, and frog, and its expression is detected in adult mouse lung (https://www.ncbi.nlm.nih.gov/gene/19152). Therefore, the 18 kD hCAP-18 precusor should be processed to 4 kD LL37 in mouse as well. Indeed, although the very small 4 kD protein is difficult to be detected by routine Western blotting, here we successfully detected the bands in our LL37-mDASC samples using an improved protocol (Fig. 3C in revised manuscript, also listed below as Fig. R5).

All commercial antibodies targeting LL-37 can recognize both the cleaved product and the precursor. Therefore, immunofluorescence cannot help us distinguish hCAP-18(18kD) and LL-37(4kD).

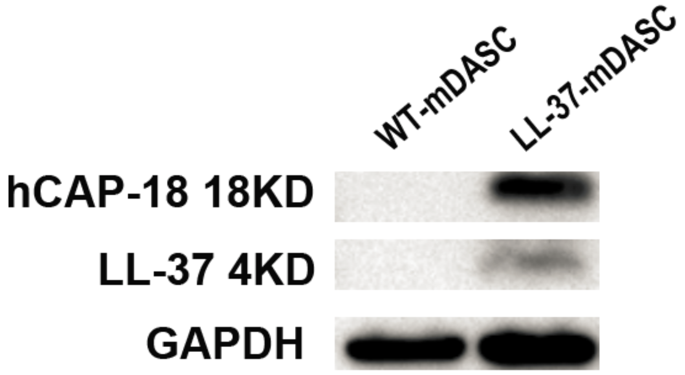

Figure R5. Western blot detection of hCAP18 (18kD) and LL-37 (4kD) in lysates of WT-mDASC and LL-37-mDASC Lane 1, WT-mDASC; lane 2, LL-37-mDASC.

5. *Fig 2 i & j: as per my previous comments, it seems that this is only a growth inhibition effect of the cathelicidin expression, but the authors claim this shows ă broad antimicrobial spectrum" (top P.7). That is misleading and does not therefore explain the claim of enhanced PAO1 killing in the murine models. Furthermore, the effect on PAO1 appears to be unrelated to LL-37 (with no significant impact of the anti-LL-37 antibody for the relevant bacteria). But then, there is not definitive data to show that the hCAP-18 has been processed to LL-37 in this system anyway*

Reply:
We thank the reviewer for reminding. We have changed the claim of "a broad antimicrobial spectrum" to the description of "a growth inhibition effect".

To confirm that the antimicrobial effect of engineered cells was attributed to LL-37 peptide production, we used anti-LL-37 antibody to neutralize the secreted peptide and increased the sample size of the experiment, and we found that anti-LL-37 antibody significantly compromised the inhibiting bacterial growth effect of LL-37-mDASCs on PAO1 (Fig.3I in the revised manuscript, lists as follow named Fig. R6 for your convenience).

As stated above, in Fig. 3C of revised manuscript, we detected the expression of LL-37 (4KDa) in LL-37-mDASC, so we confirmed that the hCAP-18 could be processed to LL-37 in this system.

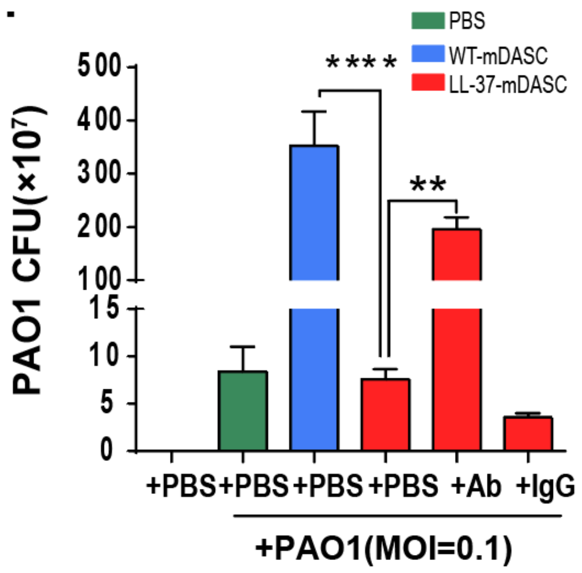

Figure R6. Preincubation of cells with anti-LL-37 antibody, but not mouse IgG, significantly reduced the targeting PAO1 growth inhibition effect of LL-37-mDASC. Initial addition of microbial was 10^3 CFU. n=3. Error bars, S.E.M.

6. *Fig 4: My concerns here have not been addressed - again the infection studies end up comparing tiny residual cfus (as little as 50 - 150 cfu /ml) at a single late time point, which is of questionable biological significance and does not provide mechanistic evaluation. In addition, the magnitude of difference for PAO1 in panel d is greater here than when the whole lung is over expressing LL-37 in figure 1. How is that explained?*

Reply:
We appreciate the reviewer's comments. The original time point is 2 days. Now we added experiment to compare the CFUs in lung homogenate and bronchoalveolar lavage fluid (BALF) 6 hours, 24 hours and 48 hours after PAO1 infection (Fig.5B and 5C in the revised manuscript, lists as follow named Fig. R7 for your convenience). As for the further mechanistic evaluation, we thanked the review for this suggestion, it will be the key point in our future research. Both transgenic LL37 expression and stem cell-based LL37 expression had statistically significant effect on PAO1 inhibition. We think it is kind of difficult to compare their "inhibition magnitude" in two totally different experimental system, and we did not rule out the possibility the transplanted stem cells could work in synergistic with LL37.

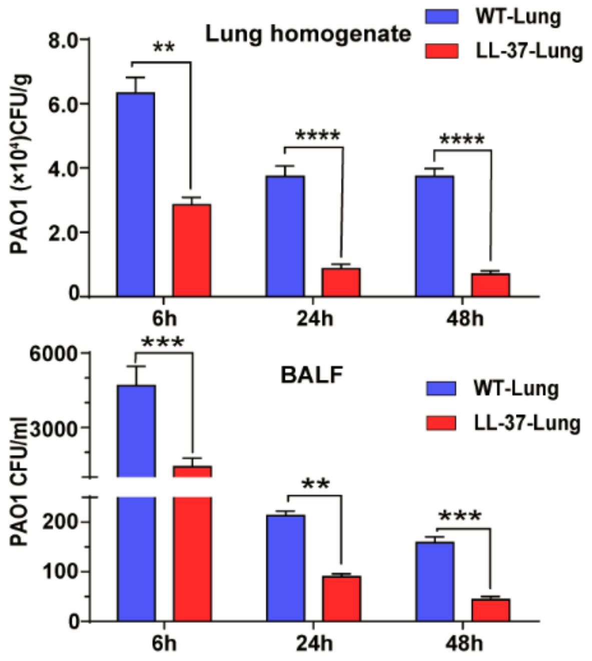

Figure R7. Intratracheal instillation of equal amount of PAO1 into WT-Lung (WT-mDASC engrafted) or LL-37-Lung (LL-37-mDASC engrafted) followed by bacterial CFU analysis in whole lung homogenates (upper panel) and BALF (down panel) 6, 24 and 48 hours after infection. n=3. Error bars, S.E.M.

Additional minor points:

7. *Page 4: Lines 22 - 23: The statement "However, the LL-37 peptide has a short half-life in vivo due to lability to proteases, limiting its clinical application." should be referenced*

Reply:
Bacteria in vivo could secrete proteolytic enzymes to cleave or degrade LL37. The statement had been referenced in the revised manuscript (introduction part):

Vandamme D, Landuyt B, Luyten W, Schoofs L (2012) A comprehensive summary of LL-37, the factotum human cathelicidin peptide. Cellular immunology 280: 22-35

8. *Page 4: Lines 23 - 24: The statement "Furthermore, the peptide needs to be delivered topically, rather than systemically, to avoid potential toxicity." should be referenced to specific toxicity concerns. Also, how does the kind of therapy proposed here avoid that potential toxicity? I would have thought that constitute pulmonary LL-37 expression would be very harmful. This should be discussed in the manuscript.*

Reply:
Thanks for reviewer's suggestion. The statement had been referenced in the revised manuscript and the references of the statement were listed below:

Johansson J, Gudmundsson GH, Rottenberg ME, Berndt KD, Agerberth B (1998) Conformation-dependent antibacterial activity of the naturally occurring human peptide LL-37. *The Journal of biological chemistry* **273:** 3718-3724.

According to the previous report, the minimal inhibitory concentration of LL-37 against *E. coli* is 5 μM, and at 13–25 μM the peptide is cytotoxic against several eukaryotic cells. Therefore, to avoid potential LL37 toxicity, it is critical to limit local LL37 concentration. Here using DASC as a carrier of LL37 has two advantages. Firstly, transplanted DASC distributed only in damaged lung instead of healthy lung, therefore the LL37 concentration in healthy lobes or areas would be low. Secondly, even if the local accumulated LL37 concentration was too high to be toxic to lung cells, the LL37-producing DASC itself should be killed first, which will then cut the source of the peptide and makes it a "self-limit" system to avoid constitute pulmonary LL-37 expression to a hazardous level. Hence, DASC could be an ideal carrying vehicle for LL-37 antimicrobial peptide to achieve improved delivery and function.

9. *Page 5: Lines 3 - 4: The authors should also reference Schaller Bals et al 2002 Am J Respir Crit Care Med 165(7):992-995, and in introducing the concept that LL-37 expression can protect against Pseudomonas lung infection the authors should reference the prior literature early e.g. Bals et al 1999, JCI, 103(8): 1113-7; Beaumont et al 2014 PLOS One 9(6) e99029; Yu et al 2010 J. Immunol 185(2):1142-1149; Kovach et al 2012 J.Immunol; Bals 1999 I&I*

Reply:
We thank reviewer for the suggestion and have referenced the following literatures in the revised manuscript:

Schaller-Bals S, Schulze A, Bals R (2002) Increased levels of antimicrobial peptides in tracheal aspirates of newborn infants during infection. American Journal of Respiratory and Critical Care Medicine, 165: 992-995

Bals R, Weiner DJ, Meegalla RL, Wilson JM (1999a) Transfer of a cathelicidin peptide antibiotic gene restores bacterial killing in a cystic fibrosis xenograft model. The Journal of Clinical Investigation, 103: 1113-7

© European Molecular Biology Organization

Bals R, Weiner DJ, Moscioni AD, Meegalla RL, Wilson JM (1999b) Augmentation of innate host defense by expression of a cathelicidin antimicrobial peptide. Infection and Immunity, 67: 6084-9

Yu FS, Cornicelli MD, Kovach MA, Newstead MW, Zeng X, Kumar A, Gao N, Yoon SG, Gallo RL, Standiford TJ (2010) Flagellin stimulates protective lung mucosal immunity: role of cathelicidin-related antimicrobial peptide. Journal of Immunology, 185: 1142-9

Kovach MA, Ballinger MN, Newstead MW, Zeng X, Bhan U, Yu FS, Moore BB, Gallo RL, Standiford TJ (2012) Cathelicidin-Related Antimicrobial Peptide Is Required for Effective Lung Mucosal Immunity in Gram-Negative Bacterial Pneumonia. J Immunol, 2012, 189 (1) 304-311

Beaumont PE, McHugh B, Gwyer Findlay E, Mackellar A, Mackenzie KJ, Gallo RL, Govan JR, Simpson AJ, Davidson DJ (2014) Cathelicidin host defence peptide augments clearance of pulmonary Pseudomonas Aeruginosa infection by its influence on neutrophil function in vivo. PloS One, 9: e99029

3rd Editorial Decision 17 October 2019

Thank you for the submission of your revised manuscript to EMBO Molecular Medicine, and please accept my apologies for the delay in getting back to you, which is due to the fact referee #2 needed more time to complete his/her evaluation. As you will see from the report below, this referee is now supportive of publication pending minor revisions. Moreover, we would like you to address minor editorial amendments before we can accept your manuscript for publication.

I look forward to reading a new revised version of your manuscript as soon as possible.

***** Reviewer's comments *****

Referee #2 (Remarks for Author):

I am delighted to see that this latest iteration of the manuscript from Zhou et al is so greatly improved. The new data have really significantly strengthened the manuscript and the authors are to be congratulated. While most of my concerns have now been addressed, there are a few that are not totally resolved.
1) In Fig 1b we now clearly see BALF 4kDa LL-37. This was really important, but there is still not enough clarity about the construct used to make the mouse in M&M P. 13, so some confusion remains. This transgenic mouse has not been reported before, so more detailed information is required in this paper, including a supplementary figure with the sequence of the construct used and a statement that clearly explains what part of the hCAP18 gene is included and what is not. I think perhaps this is simply the exonic sequence of LL-37, meaning it does not have the natural promoter region, processing sequence or the Cathelin domain. That may explain why the authors only looked for the 4 kDa and not 18 kDa peptide here. However, in the response to reviewers they write "Normally the expressed 18-KD hCAP-18 precursor need to be processed into the 4-KD LL-37 to have biological functions. So we collected the BALF from wild-type FVB and LL-37 transgenic mice and passed them through the 10KD-centrifugal filter devices" which makes me think they thought they could have detected the 18 kDa form. This needs to be clarified carefully.

2) Similar to the point above, the same is true on p17 for the pHIV-LL-37-EGFP. In the response to the reviewers, the authors show both 18 kDa and 4 kDa hCAP-18/LL-37 bands, so in this case they must be expressing the whole hCAP18 gene. This needs clarity and sequence for the construct. I can't see that figure (which is included on p.9 of the response to reviewer letter) in the manuscript - it should be added and the issue of cleavage/activation discussed clearly.

3) Legend for 1c needs more detail added - how many cfu PAO1 were inoculated at the start in this experiment. The effect is described at bacteriostatic, suggesting that, similar to the later figures in 3G, this may be a modest growth inhibition. If true, that needs to be fairly represented in the text and not over stated.

4) Page 5, line 15 - 16, rephrase "When the BALF ultrafiltrate from LL-37 transgenic mice were used to grow PAO1 in vitro,..." to properly describe what has been done here.

5) Fig 3G (and H-I): Although this has a clear growth inhibition effect, this still shows a lot of bacterial growth. The way the results text is written creates an impression about this that is a bit misleading. For example, the last column is growing from 1 x 10E4 to ~350 x 10E4 cfu, yet P.7 line 15 change says "potent growth inhibitory". Consider changing "potent growth inhibitory" to "impaired bacterial growth of PA01 at different infection dose and time points, although substantial bacterial proliferation was observed under both conditions (Fig 3G-I)".

6) Given what is observed as minor growth inhibition (point 5 above) in vitro, translates to clearly improved clearance in vivo (with really great new time course data), the authors should reflect on the discussion about whether it is possible that the mechanism in vivo is likely to be more complex than simple direct microbicidal effects. There is lots of literature on this in the field and ought to be reflected in discussion. I was interested that in the response to the reviewers' letter, the authors wrote that Beaumont et.al. " showed that expression of LL-37 lead to activation of neutrophil prior to infection, which was consistent with our new RNA-Seq analysis data in Figure 2B(same as Fig R2 below)." It is not clear to me what specific data from Fig 2B they mean. This comment could be one option to reflect on and expanded in the manuscript in the context of mechanism.

7) Fig EV2C - presumably should say "+PAO1" on x axis, not "+ E.coli"

8) P.7 Line 18 - what does "inhibiting germ growth effect" mean? Rephrase.

9) P.7, line 23 "extraordinary anti-microbial functions" is not appropriate in this context.

10) Fig 5: state cfu of PAO1 added in the figure legend

11) Fig 5: Some estimate of the quantity of LL-37 expressed in the in these lungs/BALF would really help.

12) Fig EV3 E-G - what time points after infection are being shown and what was the initial infectious inoculum of E.coli. Add details to legend.

13) It is worth reflecting in the discussion that changes in gene expression in the presence or absence of LL-37 in vivo after to infection (Fig 5) are not taking place in a simple lung infection model, but with the added complication of effects on bleomycin injury and cell engraftment. In figure 6, the difference between untreated Bleomycin+infection mice and WT-mDASC treated are most more profound than difference between WT-mDASC treated and LL-37-mDASC treated mice. Brief reflection should be added.

14) Fig 7G - what time points after infection are being shown and what were the initial infectious inoculum used. Add information to legend.

15) I think that brief statement in the discussion on the possible long-term effects of constitutive LL-37 expression must be included, and in the future I hope that the authors will characterise older transgenic mice.

16) A little English language editing assistance would help from the journal.

3rd Revision - authors' response 26 October 2019

Referee #2 (Remarks for Author):

I am delighted to see that this latest iteration of the manuscript from Zhou et al is so greatly improved. The new data have really significantly strengthened the manuscript and the authors are to be congratulated. While most of my concerns have now been addressed, there are a few that are not totally resolved.

The authors would like to thank the reviewer for his/her time and comments. Those comments are all valuable and very helpful for revising and improving our paper, as well as the important guiding significance to our researches. We have studied comments carefully and made correction at the following, which we hope, meet with approval.

The authors would like to thank the reviewer for his/her time and comments. Those comments are all valuable and very helpful for further improving our manuscript. We have worked on the comments carefully and made revision accordingly.

1) In Fig 1b we now clearly see BALF 4kDa LL-37. This was really important, but there is still not enough clarity about the construct used to make the mouse in M&M P. 13, so some confusion remains. This transgenic mouse has not been reported before, so more detailed information is required in this paper, including a supplementary figure with the sequence of the construct used and a statement that clearly explains what part of the hCAP18 gene is included and what is not. I think perhaps this is simply the exonic sequence of LL-37, meaning it does not have the natural promoter region, processing sequence or the Cathelin domain. That may explain why the authors only looked for the 4 kDa and not 18 kDa peptide here. However, in the response to reviewers they write "Normally the expressed 18-KD hCAP-18 precursor need to be processed into the 4-KD LL-37 to have biological functions. So we collected the BALF from wild-type FVB and LL-37 transgenic mice and passed them through the 10KD-centrifugal filter devices" which makes me think they thought they could have detected the 18 kDa form. This needs to be clarified carefully.

Re: We are grateful for the reviewer's comments. For the construction of LL-37 transgenic mouse, we have provided detailed information in the method in our manuscript and we have Figure 1A to summarize it. Actually we constructed a transgenic mouse, which expressed the full-length human CAMP gene. Indeed, we have detected the 18kDa precursor too but in this figure we only emphasized the functional 4-KD LL-37 we detected.

2) Similar to the point above, the same is true on p17 for the pHIV-LL-37-EGFP. In the response to the reviewers, the authors show both 18 kDa and 4 kDa hCAP-18/LL-37 bands, so in this case they must be expressing the whole hCAP18 gene. This needs clarity and sequence for the construct. I can't see that figure (which is included on p.9 of the response to reviewer letter) in the manuscript - it should be added and the issue of cleavage/activation discussed clearly.

Re: Full-length human CAMP cDNA (NCBI: NM_004345.4) was inserted into a commercial lentivirus vector pHIV-GFP (Addgene) under a constitutively active EF-1α promoter. The figure included on the p.9 response in last revision was the same one presented in Fig 3C of manuscript. We have added the information and discussed the cleavage/activation issue.

3) Legend for 1c needs more detail added - how many cfu PAO1 were inoculated at the start in this experiment. The effect is described at bacteriostatic, suggesting that, similar to the later figures in 3G, this may be a modest growth inhibition. If true, that needs to be fairly represented in the text and not over stated.

Re: We are grateful for the reviewer's suggestion. We have added more details in the legend for Figure 1C and the description of “bacteriostatic” in Figure 1C has been rephrased.

4) Page 5, line 15 - 16, rephrase "When the BALF ultrafiltrate from LL-37 transgenic mice were used to grow PAO1 in vitro,..." to properly describe what has been done here.

Re: We have rephrased this description.

5) Fig 3G (and H-I): Although this has a clear growth inhibition effect, this still shows a lot of bacterial growth. The way the results text is written creates an impression about this that is a bit misleading. For example, the last column is growing from 1 x 10E4 to ~350 x 10E4 cfu, yet P.7 line 15 change says "potent growth inhibitory". Consider changing "potent growth inhibitory" to "impaired bacterial growth of PA01 at different infection dose and time points, although substantial bacterial proliferation was observed under both conditions (Fig 3G-I)".

Re: We are grateful for the reviewer's reminds. We were sorry for that our previous description made a bit misleading. In the new manuscript, we have corrected our description.

6) Given what is observed as minor growth inhibition (point 5 above) in vitro, translates to clearly improved clearance in vivo (with really great new time course data), the authors should reflect on the discussion about whether it is possible that the mechanism in vivo is likely to be more complex than simple direct microbicidal effects. There is lots of literature on this in the field and ought to be reflected in discussion. I was interested that in the response to the reviewers' letter, the authors wrote that Beaumont et.al. " showed that expression of LL-37 lead to activation of neutrophil prior to infection, which was consistent with our new RNA-Seq analysis data in Figure 2B(same as Fig R2 below)." It is not clear to me what specific data from Fig 2B they mean. This comment could be one option to reflect on and expanded in the manuscript in the context of mechanism.

Re: We are grateful for the reviewer's comments. We agreed with the reviewer and added some discussion of complex mechanisms. Beaumont et.al found LL-37 simulated neutrophil activation. RNA-Seq data indicated that GO term of neutrophil migration is enriched between WT and LL-37+/+ mouse. We will further investigate the complex mechanism in future.

7) Fig EV2C - presumably should say "+PAO1" on x axis, not "+ E.coli"
Re: We apologize for our carelessness. We have corrected it in the updated figure.

8) P.7 Line 18 - what does "inhibiting germ growth effect" mean? Rephrase.
Re: We are grateful for the reviewer's reminds. We have changed "inhibiting germ growth effect" to "inhibitory effect".

9) P.7, line 23 "extraordinary anti-microbial functions" is not appropriate in this context.
We are grateful for the reviewer's reminds. We have modified the sentence.

10) Fig 5: state cfu of PAO1 added in the figure legend
This has been supplemented in the legend.

11) Fig 5: Some estimate of the quantity of LL-37 expressed in the in these lungs/BALF would really help.
We appreciate your kind suggestion. What we cared about here were the expressions of LL-37 in the lungs or not, not the quantity. Besides that, there is no technique that can accurately quantity the expression of LL-37 so far. For example, western-bloting only could be semi-quantitative, Elisa kit could not recognize LL-37 from hCAP-18. So it is a big challenge for us to quantify the expression of LL-37.

12) Fig EV3 E-G - what time points after infection are being shown and what was the initial infectious inoculum of E.coli. Add details to legend.

Re: These have been supplemented in the legend.

13) It is worth reflecting in the discussion that changes in gene expression in the presence or absence of LL-37 in vivo after to infection (Fig 5) are not taking place in a simple lung infection model, but with the added complication of effects on bleomycin injury and cell engraftment. In figure 6, the difference between untreated Bleomycin+infection mice and WT-mDASC treated are most more profound than difference between WT-mDASC treated and LL-37-mDASC treated mice. Brief reflection should be added.

Re: We have added discussion as suggested by the reviewer. The transplanted DASC itself could rapidly established the epithelium barrier which seems have protective effects on pulmonary infection, while the LL-37-DASCs have even stronger protective effects than WT-DASC.

14) Fig 7G - what time points after infection are being shown and what were the initial infectious inoculum used. Add information to legend.

Re: The information has been added into legend.

15) I think that brief statement in the discussion on the possible long-term effects of constitutive LL-37 expression must be included, and in the future I hope that the authors will characterise older transgenic mice.

Re: It has been discussed in the manuscript.

16) A little English language editing assistance would help from the journal.
Thanks for your suggestion that is really valuable. The manuscript has been polished by a native speaker.

Re: This manuscript has been polished by Prof. Nicholas Forsyth, who is a native English speaker from UK.

We would like to thank you again for all comments of our manuscript. We tried our best to improve the manuscript, and hope that correction will meet with approval. Once again, we really appreciate your help and patience.

© European Molecular Biology Organization

EMBO PRESS

YOU MUST COMPLETE ALL CELLS WITH A PINK BACKGROUND ➔

PLEASE NOTE THAT THIS CHECKLIST WILL BE PUBLISHED ALONGSIDE YOUR PAPER

USEFUL LINKS FOR COMPLETING THIS FORM

http://www.antibodypedia.com
http://1degreebio.org
http://www.equator-network.org/reporting-guidelines/improving-bioscience-research-repo
http://grants.nih.gov/grants/olaw/olaw.htm
http://www.mrc.ac.uk/Ourresearch/Ethicsresearchguidance/Useofanimals/index.htm
http://ClinicalTrials.gov
http://www.consort-statement.org
http://www.consort-statement.org/checklists/view/32-consort/66-title
http://www.equator-network.org/reporting-guidelines/reporting-recommendations-for-tun
http://datadryad.org
http://figshare.com
http://www.ncbi.nlm.nih.gov/gap
http://www.ebi.ac.uk/ega
http://biomodels.net/
http://biomodels.net/miriam/
http://jjj.biochem.sun.ac.za
http://oba.od.nih.gov/biosecurity/biosecurity_documents.html
http://www.selectagents.gov/

Corresponding Author Name: Jie-ming Qu, Wei Zuo, Jin-fu Xu

Journal Submitted to: EMBO Molecular Medicine
Manuscript Number: EMM-2018-10233-V4

Reporting Checklist For Life Sciences Articles (Rev. June 2017)

This checklist is used to ensure good reporting standards and to improve the reproducibility of published results. These guidelines are consistent with the Principles and Guidelines for Reporting Preclinical Research issued by the NIH in 2014. Please follow the journal's authorship guidelines in preparing your manuscript.

A- Figures

1. Data

The data shown in figures should satisfy the following conditions:

- the data were obtained and processed according to the field's best practice and are presented to reflect the results of the experiments in an accurate and unbiased manner.
- figure panels include only data points, measurements or observations that can be compared to each other in a scientifically meaningful way.
- graphs include clearly labeled error bars for independent experiments and sample sizes. Unless justified, error bars should not be shown for technical replicates.
- if n< 5, the individual data points from each experiment should be plotted and any statistical test employed should be justified
- Source Data should be included to report the data underlying graphs. Please follow the guidelines set out in the author ship guidelines on Data Presentation.

2. Captions

Each figure caption should contain the following information, for each panel where they are relevant:

- a specification of the experimental system investigated (eg cell line, species name).
- the assay(s) and method(s) used to carry out the reported observations and measurements
- an explicit mention of the biological and chemical entity(ies) that are being measured.
- an explicit mention of the biological and chemical entity(ies) that are altered/varied/perturbed in a controlled manner.

- the exact sample size (n) for each experimental group/condition, given as a number, not a range;
- a description of the sample collection allowing the reader to understand whether the samples represent technical or biological replicates (including how many animals, litters, cultures, etc.).
- a statement of how many times the experiment shown was independently replicated in the laboratory.
- definitions of statistical methods and measures:
  - common tests, such as t-test (please specify whether paired vs. unpaired), simple χ2 tests, Wilcoxon and Mann-Whitney tests, can be unambiguously identified by name only, but more complex techniques should be described in the methods section;
  - are tests one-sided or two-sided?
  - are there adjustments for multiple comparisons?
  - exact statistical test results, e.g., P values = x but not P values < x;
  - definition of 'center values' as median or average;
  - definition of error bars as s.d. or s.e.m.

Any descriptions too long for the figure legend should be included in the methods section and/or with the source data.

In the pink boxes below, please ensure that the answers to the following questions are reported in the manuscript itself. Every question should be answered. If the question is not relevant to your research, please write NA (non applicable). We encourage you to include a specific subsection in the methods section for statistics, reagents, animal models and human subjects.

B- Statistics and general methods

Please fill out these boxes ➔ (Do not worry if you cannot see all your text once you press return)

1.a. How was the sample size chosen to ensure adequate power to detect a pre-specified effect size?	No statistical methods were used to predetermine sample sizes. Sample sizes were selected empirically from previous experimental experience with similar assays, and/or from sizes generally employed in the field.
1.b. For animal studies, include a statement about sample size estimate even if no statistical methods were used.	NA
2. Describe inclusion/exclusion criteria if samples or animals were excluded from the analysis. Were the criteria pre-established?	No samples were excluded from the analysis.
3. Were any steps taken to minimize the effects of subjective bias when allocating animals/samples to treatment (e.g. randomization procedure)? If yes, please describe.	To minimize the effects of subjective bias in our research, the mice we selected were gender-consistent, similar in age, and close in weight.
For animal studies, include a statement about randomization even if no randomization was used.	NA
4.a. Were any steps taken to minimize the effects of subjective bias during group allocation or/and when assessing results (e.g. blinding of the investigator)? If yes please describe.	The investigators were blinded during data collection and analysis when calculating the lung injury scores.
4.b. For animal studies, include a statement about blinding even if no blinding was done	NA
5. For every figure, are statistical tests justified as appropriate?	YES
Do the data meet the assumptions of the tests (e.g., normal distribution)? Describe any methods used to assess it.	Kolmogorov-Smirnov test was used for assessing whether the data meet normal distribution with GraphPad Prism 7 Software
Is there an estimate of variation within each group of data?	YES

Is the variance similar between the groups that are being statistically compared?	YES

C- Reagents

6. To show that antibodies were profiled for use in the system under study (assay and species), provide a citation, catalog number and/or clone number, supplementary information or reference to an antibody validation profile. e.g., Antibodypedia (see link list at top right), 1DegreeBio (see link list at top right).	KRT5 (EP1601Y, Thermo), P63 (4A4, Abcam),AQP5 (1:200, EPR3747, Abcam), GFP (B-2, Santa Cruz), GFP (ab5450, Abcam), GFP (ab290, Abcam), LL-37/cathelicidin (ab69484, ab80895 Abcam ,D-7 Santa Cruz and HM2070, Hycult biotech),GAPDH (ab8245, Abcam) . Alexa Fluor-conjugated Donkey 488/594 (1:200, Life Technologies, USA) and HRP-conjugated anti-mouse IgG(H+L)(800151, Vazyme) were used as secondary antibodies. The commercial antibodies are well used and reported in lots of previous publications.
7. Identify the source of cell lines and report if they were recently authenticated (e.g., by STR profiling) and tested for mycoplasma contamination.	No commonly misidentified cell lines were used in this study. All the cell lines used in this study were recently authenticated. And cells were tested negative for mycoplasma contamination by Vero cell infection assay.

* for all hyperlinks, please see the table at the top right of the document

D- Animal Models

8. Report species, strain, gender, age of animals and genetic modification status where applicable. Please detail housing and husbandry conditions and the source of animals.	The information has been included in the Materials and Methods Section of the manuscript. Female C57/B6 mice and wild type FVB mice (6-8 weeks), weighing 16-18 g were purchased from Shanghai SLAC Laboratory Animal Co., Ltd. (China). LL-37+/+ mice (Fvb background) were produced by Cyagen Biosciences Inc. (China). Male Sprague-Dawley rats, weighing 180–220 g each, were purchased from shanghai SLAC laboratory animal Co., Ltd. (China). All mice were housed in specific pathogen-free conditions within an animal care facility (Center of Laboratory Animal, Tongji University, Shanghai, China).All animals were housed in a pathogen-free facility with 12 hr light/dark cycle and 24 hr access to food and water. Animal experiments in this study were approved by and performed in accordance with the institutional animal care and use committee at the Tongji University at Shanghai.
9. For experiments involving live vertebrates, include a statement of compliance with ethical regulations and identify the committee(s) approving the experiments.	All animal experiments were performed under the guidance of, and with approval from, the Institutional Animal Care and Use Committee of Tongji University.
10. We recommend consulting the ARRIVE guidelines (see link list at top right) (PLoS Biol. 8(6), e1000412, 2010) to ensure that other relevant aspects of animal studies are adequately reported. See author guidelines, under 'Reporting Guidelines'. See also: NIH (see link list at top right) and MRC (see link list at top right) recommendations. Please confirm compliance.	All animal experiments conformed to these guidelines.

E- Human Subjects

11. Identify the committee(s) approving the study protocol.	All the experiments about human subjects were approved by Shanghai East Hospital ethics committee(2016-001).
12. Include a statement confirming that informed consent was obtained from all subjects and that the experiments conformed to the principles set out in the WMA Declaration of Helsinki and the Department of Health and Human Services Belmont Report.	The informed consent was obtained from all subjects and all the experiments conformed to the principles set out in the WMA Declaration of Helsinki and the Department of Health and Human Services Belmont Report.
13. For publication of patient photos, include a statement confirming that consent to publish was obtained.	NA
14. Report any restrictions on the availability (and/or on the use) of human data or samples.	NA
15. Report the clinical trial registration number (at ClinicalTrials.gov or equivalent), where applicable.	NA
16. For phase II and III randomized controlled trials, please refer to the CONSORT flow diagram (see link list at top right) and submit the CONSORT checklist (see link list at top right) with your submission. See author guidelines, under 'Reporting Guidelines'. Please confirm you have submitted this list.	NA
17. For tumor marker prognostic studies, we recommend that you follow the REMARK reporting guidelines (see link list at top right). See author guidelines, under 'Reporting Guidelines'. Please confirm you have followed these guidelines.	NA

F- Data Accessibility

18: Provide a "Data Availability" section at the end of the Materials & Methods, listing the accession codes for data generated in this study and deposited in a public database (e.g. RNA-Seq data: Gene Expression Omnibus GSE39462, Proteomics data: PRIDE PXD000208 etc.) Please refer to our author guidelines for 'Data Deposition'.<br><br>Data deposition in a public repository is mandatory for:<br>a. Protein, DNA and RNA sequences<br>b. Macromolecular structures<br>c. Crystallographic data for small molecules<br>d. Functional genomics data<br>e. Proteomics and molecular interactions	We have provided a Data Availability section at the end of the Materials and Methods.<br>Data availability<br>The RNA-Seq data are available at the Sequence Read Archive(SRA) ((https://www.ncbi.nlm.nih.gov/sra),) database with accession numbers PRJNA559986 and PRJNA559606. (Reviewer link： https://dataview.ncbi.nlm.nih.gov/object/PRJNA559986?reviewer=a8i3eej3e50p12j80j5nkkhh95 and https://dataview.ncbi.nlm.nih.gov/object/PRJNA559606?reviewer=3tvmqjes8qqtoq1erd4tllmjod).
19. Deposition is strongly recommended for any datasets that are central and integral to the study; please consider the journal's data policy. If no structured public repository exists for a given data type, we encourage the provision of datasets in the manuscript as a Supplementary Document (see author guidelines under 'Expanded View' or in unstructured repositories such as Dryad (see link list at top right) or Figshare (see link list at top right).	NA
20. Access to human clinical and genomic datasets should be provided with as few restrictions as possible while respecting ethical obligations to the patients and relevant medical and legal issues. If practically possible and compatible with the individual consent agreement used in the study, such data should be deposited in one of the major public access-controlled repositories such as dbGAP (see link list at top right) or EGA (see link list at top right).	NA
21. Computational models that are central and integral to a study should be shared without restrictions and provided in a machine-readable form. The relevant accession numbers or links should be provided. When possible, standardized format (SBML, CellML) should be used instead of scripts (e.g. MATLAB). Authors are strongly encouraged to follow the MIRIAM guidelines (see link list at top right) and deposit their model in a public database such as Biomodels (see link list at top right) or JWS Online (see link list at top right). If computer source code is provided with the paper, it should be deposited in a public repository or included in supplementary information.	NA

G- Dual use research of concern

22. Could your study fall under dual use research restrictions? Please check biosecurity documents (see link list at top right) and list of select agents and toxins (APHIS/CDC) (see link list at top right). According to our biosecurity guidelines, provide a statement only if it could.	NA
